# Supplementary material for: Stereoselective oxidative glycosylation of anomeric nucleophiles with alcohols and carboxylic acids
Source: Nat Commun. 2018 Sep 7;9:3650. doi: 10.1038/s41467-018-06016-4 (PMC6128909; doi:10.1038/s41467-018-06016-4)
Supplement: Supplementary file 1 — Supplementary Information [file 41467_2018_6016_MOESM1_ESM.pdf]

SUPPLEMENTARY INFORMATION

Stereoselective Oxidative Glycosylation of Anomeric Nucleophiles with Alcohols and Carboxylic Acids

Tianyi Yang, Feng Zhu, and Maciej A. Walczak*

Department of Chemistry and Biochemistry, University of Colorado, Boulder, Colorado 80309, United States

Email: maciej.walczak@colorado.edu

Supplementary Notes

All chemicals were purchased as reagent grade and used without further purification unless otherwise noted. Solvents were filtered through a column of activated alumina prior to use. All reactions were carried out under anhydrous N_2 in oven-dried glassware. $Zn(OTf)_2$ was purchased from Sigma-Aldrich. Iodosobenzene and hydroxy(tosyloxy)iodobenzene was purchased from TCI Chemicals, Inc. Visualizations were performed with UV light and/or Hanessian stain and/or sulfuric acid stain (5% H_2SO_4 in MeOH). Column chromatography was performed on silica gel (230-400 mesh). ^{1}H and ^{13}C NMR spectra were recorded on Bruker/Varian 300/400/500 MHz instruments are reported as follows: chemical shift (δ), multiplicity (s = singlet, d = doublet, t = triplet, q =quartet, br = broad, m = multiplet), coupling constants (Hz), and integration. The residual solvent reference peaks were used from published literature. 2D NMR experiments were performed using standard parameters (*200 and More NMR Experiments*, S. Berger, S. Braun, Wiley-VCH, **2004**). IR measurements were performed on Agilent Cary 630 FT/IR instrument and optical rotations were measured on JASCO P-1030 and are reported as average of five data points. The ESI-MS experiments were performed on Synapt G2 HDMS™ quadrupole/ToF mass spectrometer (Waters) equipped with a standard ESI ion source. Data acquisition and analysis were carried out with the MassLynx software package.

Supplementary Methods

General Procedure A for Anomeric Acylation

Under N_2, a one-dram vial was charged with anomeric stannane (0.100 mmol, 1 equiv), phenyliodonium biscarboxylate (0.200 mmol, 2 equiv), CuCl (0.200 mmol, 2 equiv), KF (0.400 mmol, 4 equiv), freshly activated powdered 4Å MS (ca. 100 mg), anh. toluene (1.00 mL) and anh. 1,4-dioxane (1.00 mL) and was heated up to 110 °C. The reaction was stirred for 12 h, and filtered through Celite®. The filtrate was concentrated and purified by column chromatography on SiO_2.

General Procedure B for the Glycosylation with Iodosobenzene

Under N_2, anomeric stannane (0.100 mmol - 0.200 mmol), the corresponding alcohol (0.100 - 0.200 mmol), $Zn(OTf)_2$ (0.010 - 0.100 mmol), iodosobenzene (0.200 - 0.300 mmol), freshly activated 4Å MS and anh. $CHCl_3$ (0.50 - 2.00 ml) were successively added into a vial. The reaction mixture was stirred at room temperature for the indicated period of time, filtered through a pad of silica gel, and concentrated. ^1H NMR spectra were recorded using this mixture to evaluate diastereoselectivity. The crude material was purified by column chromatography on SiO_2.

General Procedure C for the Glycosylation with Hydroxy(tosyloxy)iodobenzene (Koser's Reagent)

Under N_2, anomeric stannane (0.100 mmol), the corresponding alcohol (0.100 mmol), $Zn(OTf)_2$ (0.005 mmol), Hydroxy(tosyloxy)iodobenzene (0.100 mmol), freshly activated 4Å MS and anh. CH_2Cl_2 (4.00 ml) were successively added into a vial. After stirring at room temperature for 12 h, anomeric stannane (0.100 mmol) and hydroxy(tosyloxy)iodobenzene (0.100 mmol) were added and stirred for additional 12 h. Anomeric stannane (0.100 mmol) and hydroxy(tosyloxy)iodobenzene (0.100 mmol) were added and stirred for additional 12 h. The reaction mixture was filtered through a pad of silica gel, and concentrated. ^1H NMR spectra were recorded using this mixture to evaluate diastereoselectivity. The crude material was purified by column chromatography on SiO_2.

General Procedure for Low-Temperature NMR Experiment

A suspension of anomeric stannane (36.0 mg, 0.050 mmol, 1.0 equiv), Koser reagent (21.2 mg, 0.050 mmol, 1.0 equiv) and $Zn(OTf)_2$ (18.1 mg, 0.050 mmol, 1.0 equiv) in 1.00 mL of CD_2Cl_2 in an NMR tube was cooled to -78 °C under nitrogen. The reaction was maintained at -78 °C for 10 min, and promptly inserted into the NMR instrument probe precooled to -80 °C for data acquisition. The probe temperature was maintained at each temperature for 10 min, then warmed by 10 °C over 2 min period. At each 10-min interval, the ^1H NMR spectrum was recorded.

General Procedure for the Study of Reactions by ESI-MS

A volume of 2 μL of the reaction solution from General Procedure C at a certain reaction time was diluted with 98 μL of acetonitrile, and the diluted reaction solutions was injected into the ESI source at a flow rate of 50 μL/min.

Detailed Experimental Procedures for Compounds 11 - 54

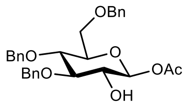

11

3,4,6-Tri-*O*-benzyl-*β*-D-glucopyranosyl acetate (**11**). According to the general protocol *A*, (3,4,6-tri-*O*-benzyl–*β*–D-glucopyranosyl)tri-*n*-butylstannane[1] **10** (72.4 mg, 0.100 mmol), freshly activated 4Å MS, PIDA (64.4 mg, 0.200 mmol), CuCl (19.8 mg, 0.200 mmol), KF (23.2 mg, 0.400 mmol), anh. toluene (1.00 mL), and anh. 1,4-dioxane (1.00 mL) were successively added into a vial. The reaction mixture was stirred at 110 °C for 12 h and afforded after chromatographic purification on SiO_2 (Hexanes:EtOAc, 3:1) **11** (37.9 mg, 77%) as a colorless oil: $[\alpha]_D^{24}$ = +24.0 (c = 1.00, $CHCl_3$); IR (ATR) ν = 3471, 3030, 2925, 1741, 1454, 1365, 1279, 1075, 735, 698 cm^{-1}; ^1H NMR (400 MHz, $CDCl_3$) δ 7.37 - 7.27 (m, 13H), 7.18 - 7.14 (m, 2H), 5.50 (d, *J* = 8.0 Hz, 1H, C1H), 4.91 (d, *J* = 11.4 Hz, 1H), 4.84 - 4.78 (m, 2H), 4.64 (d, *J* = 12.1 Hz, 1H), 4.54 (d, *J* = 10.8 Hz, 1H), 4.48 (d, *J* = 12.1 Hz, 1H), 3.80 - 3.69 (m, 3H), 3.65 (td, *J* = 8.5, 8.1, 2.5 Hz, 1H), 3.63 - 3.55 (m, 2H), 2.14 (s, 3H); ^{13}C NMR (101 MHz, $CDCl_3$) δ 169.5, 138.3, 137.8, 128.6, 128.4, 128.4, 128.0, 127.9, 127.8, 127.7, 94.0, 84.5, 77.1, 75.6, 75.3, 74.9, 73.5, 72.9, 68.0, 21.1; HRMS (ESI) *m/z* calcd for $C_{29}H_{32}O_7$ $[M + Na]^+$ 515.2046, found 515.2050.

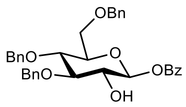

15

3,4,6-Tri-*O*-benzyl–*β*–D-glucopyranosyl benzoate (**15**). According to the general protocol *A*, (3,4,6-tri-*O*-benzyl–*β*–D-glucopyranosyl)tri-*n*-butylstannane[1] **10** (72.4 mg, 0.100 mmol), freshly activated 4Å MS, phenyliodonium dibenzoate[2] (83.0 mg, 0.200 mmol), CuCl (19.8 mg, 0.200 mmol), KF (23.2 mg, 0.400 mmol), anh. toluene (1.00 mL), and anh. 1,4-dioxane (1.00 mL) were successively added into a vial. The reaction mixture was stirred at 110 °C for 12 h and afforded after chromatographic purification on SiO_2 (Hexanes:EtOAc, 4:1) **15** (25.0 mg, 45%) as a colorless oil: $[\alpha]_D^{24}$ = -197.0 (c = 1.00, $CHCl_3$); IR (ATR) ν = 3463, 3029, 2929, 2855, 1728, 1495, 1451, 1360, 1107, 1049, 732 cm^{-1}; ^1H NMR (500 MHz, $CDCl_3$) δ 8.11 (dd, *J* = 8.3, 1.4 Hz, 1H), 7.60 - 7.56 (m, 1H), 7.44 (t, *J* = 7.8 Hz, 1H), 7.40 - 7.29 (m, 15H), 5.78 (d, *J* = 8.1 Hz, 1H, C1H), 4.91 (d, *J* = 11.5 Hz, 1H), 4.77 (d, *J* = 11.7 Hz, 1H), 4.67 (d, *J* = 11.5 Hz, 1H), 4.61 (d, *J* = 11.7 Hz, 1H), 4.50 (d, *J* = 11.7 Hz, 1H), 4.46 (d, *J* = 11.8 Hz, 1H), 4.26 (ddd, *J* = 10.2, 8.1, 2.5 Hz, 1H), 4.10 - 4.07 (m, 1H), 3.87 - 3.83 (m, 1H), 3.74 - 3.67 (m, 1H), 3.64 (dd, *J* = 9.1, 5.3 Hz, 1H), 3.57 (dd, *J* = 9.7, 2.8 Hz, 1H), 2.31 (d, *J* = 2.6 Hz, 1H); ^{13}C NMR (101 MHz, $CDCl_3$) δ 165.3, 137.8, 133.6, 130.3, 128.8, 128.6, 128.5, 128.4, 128.4, 128.2, 128.0, 127.9, 127.9, 95.1, 82.4, 74.9, 74.5, 73.7, 72.4, 72.3, 70.1, 67.9; HRMS (ESI) *m/z* calcd for $C_{34}H_{34}O_7$ $[M + Na]^+$ 577.2202, found 577.2216.

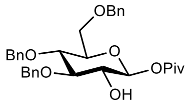

16

3,4,6-Tri-*O*-benzyl–*β*–D-glucopyranosyl pivalate (**16**). According to the general protocol *A*, (3,4,6-tri-*O*-benzyl–*β*–D-glucopyranosyl)tri-*n*-butylstannane[1] **10** (72.4 mg, 0.100 mmol), freshly activated 4Å MS, phenyliodonium dipivalate **S1** (75.0 mg, 0.202 mmol), CuCl (19.8 mg, 0.200 mmol), KF (23.2 mg, 0.400 mmol), anh. toluene (1.00 mL), and anh. 1,4-dioxane (1.00 mL) were successively added into a vial. The reaction mixture was stirred at 110 °C for 12 h and afforded after chromatographic purification on SiO_2 (Hexanes:EtOAc, 5:1) **16** (25.0 mg, 64%) as a colorless oil: $[\alpha]_D^{24}$ = +27.8 (c = 1.00, $CHCl_3$); IR (ATR) ν = 3490, 3080, 2924, 2889, 1736, 1603, 1453, 1267, 1072, 737 cm^{-1}; ^1H NMR (500 MHz, $CDCl_3$) δ 7.39 - 7.29 (m, 15H), 5.49 (d, *J* = 8.1 Hz, 1H, C1H), 4.88 (d, *J* = 11.5 Hz, 1H), 4.74 (d, *J* = 11.8 Hz, 1H), 4.66 (d, *J* = 11.5 Hz, 1H), 4.57 (d, *J* = 11.8 Hz, 1H), 4.50 (d, *J* = 11.7 Hz, 1H), 4.46 (d, *J* = 11.7 Hz, 1H), 4.08 (dd, *J* = 9.7, 8.1 Hz, 1H), 4.04 (dd, *J* = 3.0, 1.1 Hz, 1H), 3.76 (ddd, *J* = 7.9, 5.2, 1.1 Hz, 1H), 3.68 (t, *J* = 8.5 Hz, 1H), 3.62 (dd, *J* = 9.0, 5.2 Hz, 1H), 3.49 (dd, *J* = 9.8, 2.9 Hz, 1H), 2.22 (s, 1H), 1.24 (s, 9H); ^{13}C NMR (101 MHz, $CDCl_3$) δ 177.1, 138.3, 137.7, 128.6, 128.4, 128.3, 128.3, 128.0, 128.0, 127.9, 127.7, 127.7, 94.4, 82.2, 74.7, 74.3, 73.5, 72.2, 72.2, 69.9, 67.7, 38.8, 26.9; HRMS (ESI) *m/z* calcd for $C_{32}H_{38}O_7$ $[M + Na]^+$ 557.2515, found 557.2517.

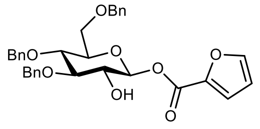

17

3,4,6-Tri-*O*-benzyl–*β*–D-glucopyranosyl furoate (**17**). According to the general protocol *A*, (3,4,6-tri-*O*-benzyl–*β*–D-glucopyranosyl)tri-*n*-butylstannane[1] **10** (72.4 mg, 0.100 mmol), freshly activated 4Å MS, phenyliodonium difuroate **S2** (79.2 mg, 0.200 mmol), CuCl (19.8 mg, 0.200 mmol), KF (23.2 mg, 0.400 mmol), anh. toluene (1.00 mL), and anh. 1,4-dioxane (1.00 mL) were successively added into a vial. The reaction mixture was stirred at 110 °C for 12 h and afforded after chromatographic purification on SiO_2 (Hexanes:EtOAc, 5:1) **17** (25.0 mg, 64%) as a colorless oil: $[\alpha]_D^{24}$ = +12.4 (c = 1.00, $CHCl_3$); IR (ATR) ν = 3466, 3029, 2921, 1730, 1576, 1471, 1360, 1293, 1178, 1067, 927 cm^{-1}; ^1H NMR (500 MHz, $CDCl_3$) δ 7.61 (d, *J* = 1.7 Hz, 1H), 7.37 - 7.27 (m, 14H), 7.20 - 7.15 (m, 2H), 6.52 (dd, *J* = 3.5, 1.7 Hz, 1H), 5.73 (d, *J* = 8.0 Hz, 1H, C1H), 4.92 (d, *J* = 11.4 Hz, 1H), 4.88 - 4.79 (m, 2H), 4.62 (d, *J* = 12.1 Hz, 1H), 4.57 (d, *J* = 10.8 Hz, 1H), 4.49 (d, *J* = 12.1 Hz, 1H), 3.85 - 3.72 (m, 4H), 3.69 - 3.61 (m, 2H), 2.26 (d, *J* = 3.2 Hz, 1H); ^{13}C NMR (101 MHz, $CDCl_3$) δ 157.1, 147.2, 143.8, 138.5, 138.0, 137.9, 128.8, 128.6, 128.5, 128.1, 128.1, 128.1, 128.0, 127.9, 119.7, 112.2, 94.5, 84.5, 77.4, 75.9, 75.5, 75.1, 73.7, 73.1, 68.2; HRMS (ESI) *m/z* calcd for $C_{32}H_{32}O_8$ $[M + Na]^+$ 567.1995, found 567.2001.

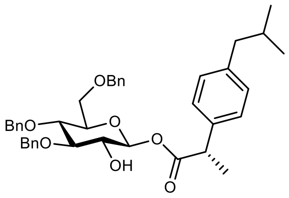

18

3,4,6-Tri-*O*-benzyl–*β*–D-glucopyranosyl *S*-(4-isobutylphenyl)propionate (**18**). According to the general protocol *A*, (3,4,6-tri-*O*-benzyl–*β*–D-glucopyranosyl)tri-n-butylstannane[1] **10** (72.4 mg, 0.100 mmol), freshly activated 4Å MS, phenyliodonium bis(ibuprofen) **S3** (116 mg, 0.200 mmol), CuCl (19.8 mg, 0.200 mmol), KF (23.2 mg, 0.400 mmol), anh. toluene (1.00 mL), and anh. 1,4-dioxane (1.00 mL) were successively added into a vial. The reaction mixture was stirred at 110 °C for 12 h and afforded after chromatographic purification on SiO_2 (Hexanes:EtOAc, 10:1) **18** (49.8 mg, 78%) as a colorless oil: $[\alpha]_D^{24}$ = +34.8 (c = 1.00, $CHCl_3$); IR (ATR) ν = 3458, 3030, 2922, 1751, 1453, 1363, 1269, 1069, 737 cm^{-1}; ^1H NMR (500 MHz, $CDCl_3$) δ 7.38 - 7.29 (m, 13H), 7.24 -7.21 (d, *J* = 8.2 Hz, 2H), 7.19 (dd, *J* = 7.4, 2.0 Hz, 2H), 7.10 - 7.07 (d, *J* = 8.2 Hz, 2H), 5.51 (d, *J* = 7.8 Hz, 1H, C1H), 4.89 (d, *J* = 11.4 Hz, 1H), 4.83 - 4.78 (m, 2H), 4.60 - 4.54 (m, 2H), 4.46 (d, *J* = 12.1 Hz, 1H), 3.80 (q, *J* = 7.2 Hz, 1H), 3.75 - 3.69 (m, 3H), 3.65 (td, *J* = 8.3, 7.9, 2.3 Hz, 1H), 3.62 - 3.56 (m, 2H), 2.43 (d, *J* = 7.2 Hz, 2H), 2.04 (d, *J* = 3.2 Hz, 1H), 1.83 (dp, *J* = 13.5, 6.7 Hz, 1H), 1.54 (d, *J* = 7.2 Hz, 3H), 0.89 (d, *J* = 6.6 Hz, 6H); ^{13}C NMR (101 MHz, $CDCl_3$) δ 173.3, 140.7, 138.3, 138.0, 137.9, 136.9, 129.3, 128.6, 128.4, 128.3, 127.9, 127.9, 127.8, 127.6, 127.2, 94.3, 84.2, 76.7, 75.9, 75.2, 74.8, 73.5, 73.0, 68.0, 45.1, 45.0, 30.1, 22.4, 18.5; HRMS (ESI) *m/z* calcd for $C_{40}H_{46}O_7$ $[M + Na]^+$ 641.3141, found 641.3144.

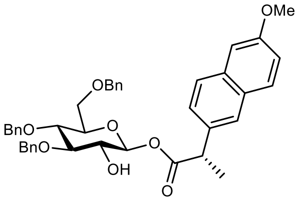

19

3,4,6-Tri-*O*-benzyl-β-D-glucopyranosyl *S*-(6-methoxynaphthyl)propionate (19). According to the general protocol A, (3,4,6-tri-O-benzyl-β-D-glucopyranosyl)tri-*n*-butylstannane[1] **10** (72.4 mg, 0.100 mmol), freshly activated 4Å MS, phenyliodonium bis(naproxen) (126 mg, 0.200 mmol), CuCl (19.8 mg, 0.200 mmol), KF (23.2 mg, 0.400 mmol), anh. toluene (1.00 mL), and anh. 1,4-dioxane (1.00 mL) were successively added into a vial. The reaction mixture was stirred at 110 °C for 12 h and afforded after chromatographic purification on SiO_2 (Hexanes:EtOAc, 7:1) **19** (49.8 mg, 78%) as a light yellow oil: $[\alpha]_D^{24}$ = +46.8 (c = 1.00, $CHCl_3$); IR (ATR) ν = 3410, 3030, 2925, 1731, 1605, 1453, 1360, 1266, 1065, 739 cm^{-1}; ^1H NMR (400 MHz, CDCl3) δ 7.69 - 7.64 (m, 3H), 7.40 (dd, *J* = 8.5, 1.8 Hz, 1H), 7.36 - 7.27 (m, 8H), 7.26 - 7.19 (m, 3H), 7.21 - 7.13 (m, 4H), 7.14 - 7.05 (m, 2H), 5.54 (d, *J* = 7.5 Hz, 1H, C1H), 4.87 (d, *J* = 11.4 Hz, 1H), 4.81 - 4.73 (m, 2H), 4.55 (d, *J* = 10.9 Hz, 1H), 4.43 (d, *J* = 12.1 Hz, 1H), 4.33 (d, *J* = 12.1 Hz, 1H), 3.98 - 3.87 (m, 4H), 3.71 - 3.52 (m, 6H), 2.10 (s, 1H), 1.61

(d, J = 7.2 Hz, 3H); ^{13}C NMR (101 MHz, $CDCl_3$) δ 173.3, 157.6, 138.3, 137.9, 137.8, 134.8, 133.7, 129.3, 128.9, 128.6, 128.4, 128.2, 127.9, 127.9, 127.9, 127.8, 127.8, 127.5, 127.1, 126.2, 126.1, 118.9, 105.5, 94.2, 84.1, 75.9, 75.1, 74.7, 73.4, 72.7, 68.0, 55.3, 45.4, 29.7, 18.6; HRMS (ESI) *m/z* calcd for $C_{41}H_{42}O_8$ [M + Na]+ 685.2777, found 685.2785.

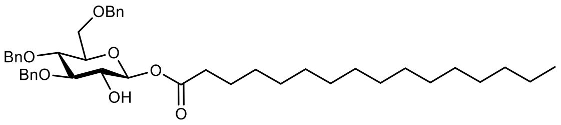

20

3,4,6-Tri-*O*-benzyl–*β*–D-glucopyranosyl palmitate (20). According to the general protocol *A*, (3,4,6-tri-*O*-benzyl–*β*–D-glucopyranosyl)tri-*n*-butylstannane[1] **10** (72.4 mg, 0.100 mmol), freshly activated 4Å MS, phenyliodonium dipalmate **S4** (137 mg, 0.200 mmol), CuCl (19.8 mg, 0.200 mmol), KF (23.2 mg, 0.400 mmol), anh. toluene (1.00 mL), and anh. 1,4-dioxane (1.00 mL) were successively added into a vial. The reaction mixture was stirred at 110 °C for 12 h and afforded after chromatographic purification on SiO_2 (Hexanes:EtOAc, 10:1) **20** (56.6 mg, 82%) as a colorless oil: $[\alpha]_D^{24}$ = -14.8 (c = 1.00, $CHCl_3$); IR (ATR) ν = 3467, 2923, 2853, 1729, 1616, 1454, 1361, 1272, 1070, 711 cm^{-1}; ^{1}H NMR (500 MHz, $CDCl_3$) δ 7.39 - 7.28 (m, 13H), 7.20 - 7.15 (m, 2H), 5.53 (d, J = 8.0 Hz, 1H, C1H), 4.92 (d, J = 11.4 Hz, 1H), 4.87 - 4.80 (m, 2H), 4.66 (d, J = 12.1 Hz, 1H), 4.57 (d, J = 10.8 Hz, 1H), 4.51 (d, J = 12.1 Hz, 1H), 3.80 - 3.72 (m, 3H), 3.69 - 3.65 (m, 1H), 3.64 - 3.57 (m, 2H), 2.41 (t, J = 7.6 Hz, 2H), 1.65 (p, J = 7.5, 2H), 1.35 - 1.19 (m, 24H), 0.90 (t, J = 6.9 Hz, 3H); ^{13}C NMR (101 MHz, $CDCl_3$) δ 172.6, 138.5, 138.0, 138.0, 128.8, 128.6, 128.5, 128.1, 128.1, 128.1, 128.1, 128.0, 127.9, 94.0, 84.7, 77.3, 75.8, 75.5, 75.1, 73.7, 73.2, 68.2, 34.3, 32.1, 29.8, 29.8, 29.8, 29.6, 29.5, 29.4, 29.2, 24.7, 22.9, 14.3; HRMS (ESI) *m/z* calcd for $C_{43}H_{60}O_7$ $[M + Na]^+$ 711.4236, found 711.4233.

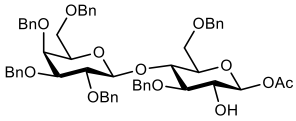

21

(2,3,4,6-tetra-*O*-benzyl–*β*–D-galactopyranosyl-(1→4)-3,6-di-*O*-benzyl–*β*–D-glucopyanosyl acetate (21). According to the general protocol *A*, [2,3,4,6-tetra-*O*-benzyl–*β*–D-galactopyranosyl-(1→4)-3,6-di-*O*-benzyl-*β*-D-glucopyranosyl]tri-*n*-butylstannane[1] (115.6 mg, 0.100 mmol), freshly activated 4Å MS, PIDA (64.4 mg, 0.200 mmol), CuCl (19.8 mg, 0.200 mmol), KF (23.2 mg, 0.400 mmol), anh. toluene (1.00 mL), and anh. 1,4-dioxane (1.00 mL) were successively added into a vial. The reaction mixture was stirred at 110 °C for 12 h and afforded after chromatographic purification on SiO_2 (Hexanes:EtOAc, 1:1) **21** (51.8 mg, 56%) as a colorless oil: $[\alpha]_D^{24}$ = +24.6 (c = 1.00, $CHCl_3$); IR (ATR) ν = 3460, 3029, 2919, 2869, 1754, 1500, 1453, 1225, 1071, 736 cm^{-1}; ^{1}H NMR (500 MHz, $CDCl_3$) δ 7.37 - 7.27 (m, 20H), 7.24 - 7.19 (m, 10H), 5.52 (d, J = 8.1 Hz, 1H, C1H), 5.14 (d, J = 11.1 Hz, 1H), 4.96 (d, J = 11.6

Hz, 1H), 4.80 (d, J = 11.0 Hz, 1H), 4.76 - 4.70 (m, 2H), 4.69 (d, J = 11.9 Hz, 1H), 4.62 (d, J = 11.1 Hz, 1H), 4.59 - 4.50 (m, 2H), 4.40 - 4.35 (m, 2H), 4.33 (d, J = 12.0 Hz, 1H), 4.29 (d, J = 11.8 Hz, 1H), 4.06 (t, J = 9.3 Hz, 1H), 3.90 (d, J = 3.0 Hz, 1H), 3.84 (dd, J = 11.0, 3.1 Hz, 1H), 3.74 (dd, J = 9.7, 7.7 Hz, 1H), 3.63 - 3.56 (m, 2H), 3.54 (t, J = 8.6 Hz, 1H), 3.51 - 3.46 (m, 2H), 3.43 (dd, J = 9.1, 5.2 Hz, 1H), 3.37 - 3.30 (m, 2H), 2.36 (d, J = 2.4 Hz, 1H), 2.13 (s, 3H); ^{13}C NMR (101 MHz, $CDCl_3$) δ 169.8, 139.1, 138.8, 138.7, 138.6, 138.1, 128.5, 128.5, 128.4, 128.4, 128.4, 128.3, 128.1, 128.0, 128.0, 127.9, 127.7, 127.7, 127.5, 127.5, 102.8, 93.9, 82.9, 82.4, 79.9, 76.1, 75.5, 75.4, 75.0, 74.7, 73.7, 73.6, 73.3, 73.1, 72.7, 72.0, 68.3, 67.6, 21.3; HRMS (ESI) *m/z* calcd for $C_{56}H_{60}O_{12}$ $[M + Na]^+$ 947.3983, found 947.3972.

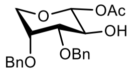

22

3,4-Di-*O*-benzyl–*β*–D-arabinopyranosyl acetate (22). According to the general protocol *A*, (3,4-di-*O*-benzyl-*β*-D-arabinosyl)tri-*n*-butylstannane **S5** (60.3 mg, 0.100 mmol), freshly activated 4Å MS, PIDA (64.4 mg, 0.200 mmol), CuCl (19.8 mg, 0.200 mmol), KF (23.2 mg, 0.400 mmol), anh. toluene (1.00 mL), and anh. 1,4-dioxane (1.00 mL) were successively added into a vial. The reaction mixture was stirred at 110 °C for 12 h and afforded after chromatographic purification on SiO_2 (Hexanes:EtOAc, 5:1) **22** (22.7 mg, 61%) as a colorless oil: $[\alpha]_D^{24}$ = -34.8 (c = 1.00, $CHCl_3$); IR (ATR) ν = 3462, 3029, 2923, 1743, 1602, 1453, 1366, 1228, 1085, 1050, 738 cm^{-1}; ^{1}H NMR (500 MHz, $CDCl_3$) δ 7.45 - 7.29 (m, 10H), 5.47 (d, J = 7.7 Hz, 1H, C1H), 4.77 (d, J = 12.3 Hz, 1H), 4.67 (d, J = 12.2 Hz, 1H), 4.62 (d, J = 12.2 Hz, 1H), 4.52 (d, J = 11.8 Hz, 1H), 4.16 - 4.07 (m, 2H), 3.78 (s, 1H), 3.51 - 3.46 (m, 2H), 2.35 (br, 1H), 2.17 (s, 3H); ^{13}C NMR (101 MHz, $CDCl_3$) δ 169.8, 137.7, 137.5, 128.6, 128.4, 128.4, 128.0, 128.0, 127.8, 127.8, 127.8, 94.6, 80.2, 71.7, 71.3, 71.1, 69.3, 64.1, 29.7; HRMS (ESI) *m/z* calcd for $C_{21}H_{24}O_6$ $[M + Na]^+$ 395.1471, found 395.1465.

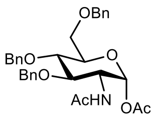

23

2-Acetamido-3,4,6-tri-*O*-benzyl-2-deoxy-*α*-D-glucopyranosyl acetate (23). According to the general protocol *A*, (2-acetamido-3,4,6-tri-*O*-benzyl-2-deoxy-*α*-D-glucosyl)tri-*n*-butylstannane[1] (76.5 mg, 0.100 mmol), freshly activated 4Å MS, PIDA (64.4 mg, 0.200 mmol), CuCl (19.8 mg, 0.200 mmol), KF (23.2 mg, 0.400 mmol), anh. toluene (1.00 mL), and anh. 1,4-dioxane (1.00 mL) were successively added into a vial. The reaction mixture was stirred at 110 °C for 12 h and afforded after chromatographic purification on SiO_2 (Hexanes:EtOAc, 2:1) **23** (22.7 mg, 73%) as a colorless oil: $[\alpha]_D^{24}$ = +75.7 (c = 1.00, $CHCl_3$); IR (ATR) ν = 3297, 3030, 2918, 1751, 1654, 1544, 1452, 1372, 1273, 1224, 1126, 1027, 941 cm^{-1}; ^{1}H NMR (400 MHz, $CDCl_3$) δ 7.39 - 7.25 (m, 13H), 7.21 - 7.15 (m, 2H), 6.12 (d, J = 3.6 Hz, 1H, C1H), 4.91 - 4.84 (m, 2H), 4.80 (d,

J = 10.6 Hz, 1H), 4.67 - 4.59 (m, 2H), 4.57 (d, J = 10.5 Hz, 1H), 4.49 (d, J = 12.1 Hz, 1H), 4.31 (ddd, J = 10.7, 8.6, 3.5 Hz, 1H), 3.86 (dd, J = 9.9, 8.6 Hz, 1H), 3.82 - 3.74 (m, 2H), 3.73 - 3.61 (m, 2H), 2.05 (s, 3H), 1.77 (s, 3H); ^{13}C NMR (101 MHz, $CDCl_3$) δ 170.0, 169.1, 138.3, 138.0, 137.9, 128.8, 128.7, 128.5, 128.4, 128.3, 128.2, 128.1, 128.0, 127.8, 91.8, 79.1, 76.8, 75.3, 74.7, 73.7, 73.5, 68.2, 51.5, 23.3, 21.0; HRMS (ESI) *m/z* calcd for $C_{31}H_{35}NO_7$ [M + Na]$^+$ 556.2311, found 556.2323.

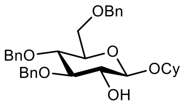

25

Cyclohexyl 3,4,6-tri-*O*-benzyl-*β*-D-glucopyranoside (25). According to the general protocol *B*, (3,4,6-tri-*O*-benzyl–*β*–D-glucopyranosyl)tri-*n*-butylstannane[1] **10** (72.4 mg, 0.100 mmol), cyclohexanol (20.8 μl, 0.200 mmol), $Zn(OTf)_2$ (7.30 mg, 0.020 mmol), iodosobenzene (44.0 mg, 0.200 mmol), freshly activated 4Å MS and anh. $CHCl_3$ (1.00 mL) were successively added into a vial. The reaction mixture was stirred at room temperature for 24 h and afforded after chromatographic purification on SiO_2 (Hexanes:EtOAc, 5:1) **25** (45.9 mg, 86%) as a colorless oil: $[\alpha]_D^{24}$ = -8.0 (c = 1.00, $CHCl_3$); IR (ATR) ν = 3463, 3029, 2929, 2855, 1728, 1495, 1451, 1360, 1107, 1049, 732 cm^{-1}; ^{1}H NMR (300 MHz, $CDCl_3$) δ 7.43 - 7.29 (m, 13H), 7.24 - 7.19 (m, 2H), 4.98 (d, J = 11.3 Hz, 1H), 4.87 (d, J = 10.9 Hz, 1H), 4.85 (d, J = 11.3 Hz, 1H), 4.64 (d, J = 12.2 Hz, 1H), 4.61 - 4.54 (m, 2H), 4.38 (d, J = 7.2 Hz, 1H, C1H), 3.81 - 3.65 (m, 3H), 3.64 - 3.47 (m, 4H), 2.36 (d, J = 1.9 Hz, 1H), 2.09 - 1.90 (m, 2H), 1.77 (m, 2H), 1.61 - 1.16 (m, 6H); ^{13}C NMR (75 MHz, $CDCl_3$) δ 138.9, 138.4, 138.3, 128.5 (2), 128.4, 128.1, 128.0, 127.9, 127.8, 127.7 (2), 101.2, 84.8, 77.8, 77.7, 75.3, 75.2, 75.1, 74.9, 73.5, 69.2, 33.8, 32.1, 25.7, 24.3, 24.2; HRMS (ESI) *m/z* calcd for $C_{33}H_{40}O_6$ [M + Na]$^+$ 555.2717, found 555.2722.

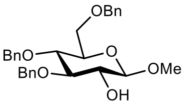

27

Methyl 3,4,6-tri-*O*-benzyl-*β*-D-glucopyranoside (27).[3] According to the general protocol *B*, (3,4,6-tri-*O*-benzyl–*β*–D-glucopyranosyl)tri-*n*-butylstannane[1] **10** (72.4 mg, 0.100 mmol), methanol (8.00 μl, 0.200 mmol), $Zn(OTf)_2$ (7.30 mg, 0.020 mmol), iodosobenzene (66.0 mg, 0.300 mmol), freshly activated 4Å MS and anh. $CHCl_3$ (1.00 mL) were successively added into a vial. The reaction mixture was stirred at room temperature for 24 h and afforded after chromatographic purification on SiO_2 (Hexanes:EtOAc, 3:1) **27** (40.0 mg, 86%) as a colorless oil: ^{1}H NMR (300 MHz, $CDCl_3$) δ 7.44 - 7.28 (m, 13H), 7.24 - 7.17 (m, 2H), 4.97 (d, J = 11.3 Hz, 1H), 4.89 (d, J = 11.3 Hz, 1H), 4.87 (d, J = 10.8 Hz, 1H), 4.67 (d, J = 12.2 Hz, 1H), 4.63 - 4.54 (m, 2H), 4.22 (d, J = 7.2 Hz, 1H, C1H), 3.88 -3.71 (m, 2H), 3.61 - 3.68 (m, 2H), 3.60 (s, 3H),3.58 - 3.46 (m, 2H), 2.58 (d, J = 1.9 Hz, 1H); ^{13}C NMR (75 MHz, $CDCl_3$) δ 138.7, 138.2,

138.1, 128.5 (2), 128.4, 128.0 (2), 127.9, 127.8 (2), 127.7, 103.8, 84.6, 77.7, 75.2, 75.1, 74.7, 73.6, 68.9, 57.2. Characterization data matched the literature report.[3]

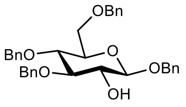

28

Benzyl 3,4,6-tri-*O*-benzyl-*β*-D-glucopyranoside (**28**). According to the general protocol *B*, (3,4,6-tri-*O*-benzyl–*β*–D-glucopyranosyl)tri-*n*-butylstannane[1] **10** (144.8 mg, 0.200 mmol), benzyl alcohol (10.8 mg, 0.100 mmol), $Zn(OTf)_2$ (7.30 mg, 0.020 mmol), iodosobenzene (66.0 mg,0.300 mmol), freshly activated 4Å MS and anh. $CHCl_3$ (1.00 mL) were successively added into a vial. The reaction mixture was stirred at room temperature for 24 h and afforded after chromatographic purification on SiO_2 (Hexanes:EtOAc, 5:1) **28** (33.0 mg, 61%) as a colorless oil: $[\alpha]_D^{24}$ = -20.3 (c = 0.50, $CHCl_3$); IR (ATR) = 3454, 3030, 2866, 2359, 1496, 1453, 1360, 1208, 1110, 1061, 735 cm^{-1}; ^1H NMR (300 MHz, $CDCl_3$) δ 7.42 - 7.27 (m, 18H), 7.19 (dt, *J* = 6.5, 2.5 Hz, 2H), 4.96 (d, *J* = 11.7 Hz, 1H), 4.93 (d, *J* = 11.2 Hz, 1H), 4.85 (d, *J* = 10.8 Hz, 1H), 4.83 (d, *J* = 11.3 Hz, 1H), 4.65 (d, *J* = 12.2 Hz, 1H), 4.64 (d, *J* = 11.8 Hz, 1H), 4.57 (d, *J* = 12.2 Hz, 1H), 4.56 (d, *J* = 10.8 Hz, 1H), 4.37 (d, *J* = 7.0 Hz, 1H, C1H), 3.86 - 3.69 (m, 2H), 3.69 - 3.55 (m, 3H), 3.52 - 3.42 (m, 1H), 2.32 (d, *J* = 1.9 Hz, 1H); ^{13}C NMR (75 MHz, $CDCl_3$) δ 138.8, 138.3, 138.3, 137.3, 128.6, 128.6, 128.5, 128.5, 128.3, 128.1, 128.1, 128.1, 127.9, 127.9, 127.8, 127.8, 101.8, 84.7, 77.7, 75.4, 75.3, 75.1, 74.9, 73.7, 71.2, 69.1; HRMS (ESI) *m/z* calcd for $C_{34}H_{36}O_6$ $[M + Na]^+$ 563.2404, found 563.2401.

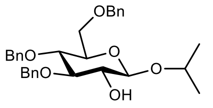

29

Isopropyl 3,4,6-tri-*O*-benzyl-*β*-D-glucopyranoside (29).[4] According to the general protocol *B*, (3,4,6-tri-*O*-benzyl-*β*-D-glucopyranosyl)tri-*n*-butylstannane[1] **10** (144.8 mg, 0.200 mmol), isopropanol (7.70 μl, 0.100 mmol), $Zn(OTf)_2$ (7.30 mg, 0.020 mmol), iodosobenzene (66.0 mg, 0.300 mmol), freshly activated 4Å MS and anh. $CHCl_3$ (1.00 mL) were successively added into a vial. The reaction mixture was stirred at room temperature for 24 h and afforded after chromatographic purification on SiO_2 (Hexanes:EtOAc, 5:1) **29** (37.4 mg, 76%) as a colorless oil: $[\alpha]_D^{24}$ = -12.3 (c = 1.00, $CHCl_3$); IR (ATR) ν = 3447, 3031, 2927, 2868, 1725, 1483, 1453, 1360, 1167, 1099, 738, 698 cm^{-1}; ^1H NMR (300 MHz, $CDCl_3$) δ 7.42 - 7.27 (m, 13H), 7.20 - 7.17 (m, 2H), 4.95 (d, *J* = 11.3 Hz, 1H), 4.84 (d, *J* = 10.8 Hz, 1H); 4.83 (d, *J* = 11.3 Hz, 1H), 4.62 (d, *J* = 12.3 Hz, 1H), 4.58 - 4.51 (m, 2H), 4.31 (d, *J* = 7.4 Hz, 1H, C1H), 4.02 (p, *J* = 6.2 Hz, 1H), 3.79 - 3.44 (m, 6H), 2.28 (d, *J* = 1.8 Hz, 1H), 1.29 (d, *J* = 6.2 Hz, 3H), 1.20 (d, *J* = 6.1 Hz, 3H); ^{13}C NMR (75 MHz, $CDCl_3$) δ 138.9, 138.4, 138.3, 128.6, 128.5, 128.5, 128.1, 128.1, 127.9, 127.8, 127.7, 101.3, 84.8, 77.8, 75.3, 75.2, 75.2, 74.9, 73.6, 72.1, 69.2, 23.7, 22.2. Characterization data matched the literature report.[4]

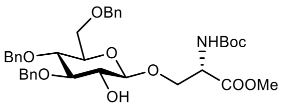

30

***N*-(*tert*-Butoxycarbonyl)-*O*-(3,4,6-tri-*O*-benzyl-*β*-D-glucopyranosyl)-L-serine methyl ester (30).** According to the general protocol *B*, (3,4,6-tri-*O*-benzyl-*β*-D-glucopyranosyl)tri-*n*-butylstannane[1] **10** (144.8 mg, 0.200 mmol), methyl (*tert*-butoxycarbonyl)-D-serine (21.9 mg, 0.100 mmol), $Zn(OTf)_2$ (7.30 mg, 0.020 mmol), iodosobenzene (66.0 mg, 0.300 mmol), freshly activated 4Å MS and anh. $CHCl_3$ (1.00 mL) were successively added into a vial. The reaction mixture was stirred at room temperature for 24 h and afforded after chromatographic purification on SiO_2 (Hexanes:EtOAc, 3:1) **30** (54.1 mg, 83%) as a colorless foam: $[\alpha]_D^{24}$ = +5.1 (c = 0.50, $CHCl_3$); IR (ATR) ν = 3419, 2868, 1713, 1497, 1453, 1364, 1211, 1161, 1058, 737, 698 cm^{-1}; ^1H NMR (300 MHz, $CDCl_3$) δ 7.38 - 7.27 (m, 13H), 7.20 - 7.14 (m, 2H), 5.56 - 5.48 (m, 1H), 4.96 (d, *J* = 11.3 Hz, 1H), 4.90 - 4.77 (m, 2H), 4.64 - 4.50 (m, 4H), 4.28 (d, *J* = 7.2 Hz, 1H, C1H), 4.20 (dd, *J* = 10.8, 5.0 Hz, 1H), 3.88 (dd, *J* = 10.8, 3.4 Hz, 1H), 3.75 (s, 3H), 3.71 - 3.41 (m, 6H), 2.96 (s, 1H), 1.45 (s, 9H); ^{13}C NMR (75 MHz, $CDCl_3$) δ 170.6, 155.9, 138.8, 138.2 (2), 128.6, 128.5 (2), 128.1 (2), 128.0, 127.9, 127.8 (2), 103.9, 84.6, 77.4, 75.3 (2), 75.2, 74.7, 73.7, 70.7, 68.9, 54.3, 52.8, 28.5; HRMS (ESI) *m/z* calcd for $C_{36}H_{45}NO_{10}$ $[M + Na]^+$ 674.2936, found 674.2947.

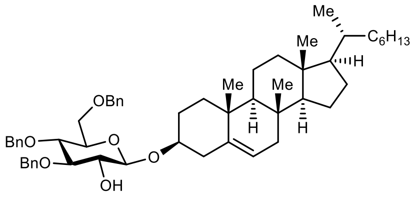

31

***O*-(3,4,6-Tri-*O*-benzyl-β-D-glucopyranosyl)cholesterol (31).**[5] According to the general protocol B, (3,4,6-tri-*O*-benzy-β-D-glucopyranosyl)tri-*n*-butylstannane **10** (144.8 mg, 0.200 mmol), cholesterol (38.7 mg, 0.100 mmol), $Zn(OTf)_2$ (36.5 mg, 0.100 mmol), iodosobenzene (66.0 mg, 0.300 mmol), freshly activated 4Å MS and anh. $CHCl_3$ (1.00 mL) were successively added into a vial. The reaction mixture was stirred at room temperature for 48 h and afforded after chromatographic purification on SiO_2 (Hexanes:EtOAc, 8:1) **31** (56.7 mg, 68%) as a colorless foam: $[\alpha]_D^{25}$ = -13.2 (c = 1.00, $CHCl_3$); IR (ATR) ν = 3460, 3029, 2933, 2863, 1453, 1362, 1110, 1060, 732, 696 cm^{-1}; ^1H NMR (300 MHz, $CDCl_3$) δ 7.42 - 7.27 (m, 13H), 7.23 - 7.17 (m, 2H), 5.36 (dt, *J* = 5.9, 1.7 Hz, 1H), 4.95 (d, *J* = 11.3 Hz, 1H), 4.85 (d, *J* = 10.9 Hz, 1H), 4.84 (d, *J* = 11.3 Hz, 1H), 4.61 (d, *J* = 12.2 Hz, 1H), 4.58 - 4.52 (m, 2H), 4.36 (d, *J* = 7.3 Hz, 1H, C1H), 3.79 - 3.64 (m, 2H), 3.64 - 3.44 (m, 5H), 2.41 - 2.32 (m, 1H), 2.30 (d, *J* = 1.9 Hz, 1H), 2.28 - 2.22 (m, 1H), 2.01 (tq, *J* = 9.5, 2.9, 2.5 Hz, 3H), 1.92 - 1.77 (m, 2H), 1.72 - 1.58 (m, 2H), 1.58 - 0.99 (m, 22H), 0.93 (d, *J* = 6.4 Hz, 3H), 0.89 (d, *J* = 1.3 Hz, 3H),

0.87 (d, J = 1.3 Hz, 3H), 0.69 (s, 3H); ^{13}C NMR (75 MHz, $CDCl_3$) δ 140.5, 138.9, 138.4, 138.3, 128.6, 128.5 (2), 128.1 (2), 127.9 (2), 127.8, 127.7, 122.2, 101.4, 84.8, 79.3, 77.8, 75.3, 75.2, 75.1, 74.9, 73.6, 69.2, 56.9, 56.3, 50.3, 42.5, 39.9, 39.7, 39.1, 37.4, 36.9, 36.3, 35.9, 32.1, 32.0, 29.9, 28.4, 28.2, 24.4, 24.0, 23.0, 22.7, 21.2, 19.5, 18.9, 12.0. Characterization data matched the literature report.[5]

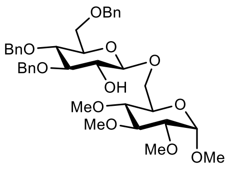

32

Methyl 3,4,6-tri-*O*-benzyl-*β*-D-glucopyranosyl-(1→6)-2,3,4-tri-O-methyl–*α*–D-glucopyranoside (32). According to the general protocol *C*, (3,4,6-tri-*O*-benzyl-*β*-D-glucopyranosyl)tri-n-butylstannane **10** (72.4 mg, 0.100 mmol), methyl 2,3,4-tri-*O*-methyl-*α*-D-glucopyranoside[6] (23.6 mg, 0.100 mmol), $Zn(OTf)_2$ (1.83 mg, 0.005 mmol), hydroxy(tosyloxy)iodobenzene (39.2 mg, 0.100 mmol), freshly activated 4Å MS and anh. CH_2Cl_2 (4.00 mL) were successively added into a vial. After stirring at room temperature for 12 h, (3,4,6-tri-*O*-benzyl-*β*-D-glucopyranosyl)tri-*n*-butylstannane[1] **10** (72.4 mg, 0.100 mmol) and hydroxy(tosyloxy)iodobenzene (39.2 mg, 0.100 mmol) were added and stirred for additional 12 h. (3,4,6-Tri-*O*-benzyl–*β*–D-glucopyranosyl)tri-*n*-butylstannane **10** (72.4 mg, 0.100 mmol) and hydroxy(tosyloxy)iodobenzene (39.2 mg, 0.100 mmol) were added and stirred at room temperature for 12 h and afforded after chromatographic purification on SiO_2 (Hexanes:EtOAc, 1:1.5) **32** (53.5 mg, 80%) as a colorless oil: $[\alpha]_D^{25}$ = +43.2 (c = 1.00, $CHCl_3$); IR (ATR) ν = 3461, 2904, 1494, 1452, 1359, 1081, 1045, 908, 734, 697 cm^{-1}; ^{1}H NMR (300 MHz, $CDCl_3$) δ 7.40 - 7.28 (m, 13H), 7.18 (dt, J = 6.5, 2.3 Hz, 2H), 4.96 (d, J = 11.2 Hz, 1H), 4.90 - 4.79 (m, 3H), 4.62 (d, J = 12.2 Hz, 1H), 4.55 (d, J = 12.2 Hz, 1H), 4.54 (d, J = 10.9 Hz, 1H), 4.38 - 4.30 (m, 1H, C1H), 4.22 - 4.13 (m, 1H), 3.78 - 3.67 (m, 4H), 3.63 (s, 3H), 3.61 - 3.57 (m, 3H), 3.55 (s, 3H), 3.53 - 3.49 (m, 2H), 3.51 (s, 3H), 3.42 (s, 3H), 3.21 (dd, J = 9.6, 3.6 Hz, 1H), 3.13 (t, J = 9.2 Hz, 1H), 2.68 (s, 1H); ^{13}C NMR (75 MHz, $CDCl_3$) δ 138.8, 138.3, 138.2, 128.5 (3), 128.1, 128.0, 127.9, 127.8 (2), 127.7, 103.7, 97.5, 84.7, 83.5, 81.8, 80.0, 75.5, 75.3, 75.2, 74.7, 73.6, 69.8, 69.1 (2), 61.0, 60.6, 59.1, 55.4; HRMS (ESI) *m/z* calcd for $C_{37}H_{48}O_{11}$ $[M + Na]^+$ 691.3089, found 591.3091.

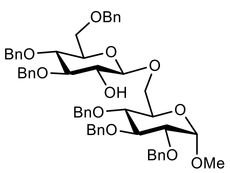

33

Methyl 3,4,6-tri-*O*-benzyl-*β*-D-glucopyranosyl-(1→6)-2,3,4-tri-*O*-benzyl-*β*-D -glucopyranoside (33). According to the general protocol C, (3,4,6-tri-O-benzyl-*β*-D-glucopyranosyl)tri-*n*-butylstannane **10** (72.4 mg, 0.100 mmol), methyl 2,3,4-tri-*O*-benzyl-α-D-glucopyranoside[7] (46.5 mg, 0.100 mmol), $Zn(OTf)_2$ (1.83 mg, 0.005 mmol), hydroxy(tosyloxy)iodobenzene (39.2 mg, 0.100 mmol), freshly activated 4Å MS and anh. CH_2Cl_2 (4.00 mL) were successively added into a vial. After stirring at room temperature for 12 h, (3,4,6-tri-O-benzyl-*β*-D-glucopyranosyl)tri-*n*-butylstannane **10** (72.4 mg, 0.100 mmol) and hydroxy(tosyloxy)iodobenzene (39.2 mg, 0.100 mmol) were added and stirred for additional 12 h. (3,4,6-Tri-*O*-benzyl-*β*-D-glucopyranosyl)tri-*n*-butylstannane **10** (72.4 mg, 0.100 mmol) and hydroxy(tosyloxy)iodobenzene (39.2 mg, 0.100 mmol) were added and stirred at room temperature for 12 h and afforded after chromatographic purification on SiO_2 (Hexanes:EtOAc, 3:1) **33** (76.3 mg, 85%) as a colorless oil: $[\alpha]_D^{24}$ = +12.8 (c = 0.10, $CHCl_3$); IR (ATR) ν = 3454, 3029, 2902, 1483, 1453, 1359, 1050, 911, 735, 697, 462 cm^{-1}; ^1H NMR (300 MHz, $CDCl_3$) δ 7.40 - 7.26 (m, 28H), 7.22 - 7.14 (m, 2H), 5.00 (d, *J* = 10.9 Hz, 1H), 4.93 (d, *J* = 11.2 Hz, 1H), 4.91 (d, *J* = 11.1 Hz, 1H), 4.86 - 4.77 (m, 4H), 4.72 - 4.50 (m, 6H), 4.29 - 4.21 (m, 1H), 4.16 (dd, *J* = 11.0, 2.2 Hz, 1H, C1H), 4.02 (t, *J* = 9.2 Hz, 1H), 3.84 (ddd, *J* = 10.1, 5.2, 2.1 Hz, 1H), 3.79 - 3.63 (m, 3H), 3.62 - 3.44 (m, 6H), 3.39 (s, 3H), 2.49 (d, *J* = 1.4 Hz, 1H); ^{13}C NMR (75 MHz, $CDCl_3$) δ 138.8 (2), 138.4, 138.3, 138.2, 128.6 (2), 128.5 (2), 128.3, 128.1 (2), 128.0, 127.9(3), 127.8(2), 127.7 (2), 103.6, 98.2, 84.6, 82.1, 79.9, 78.2, 77.6, 75.9, 75.5, 75.2, 75.1 (2), 74.6, 73.6, 73.5, 70.0, 69.1, 68.9, 55.4; HRMS (ESI) m/z calcd for $C_{55}H_{60}O_{11}$ [M + Na]+ 919.4028, found 919.4021.

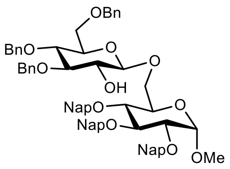

34

Methyl 3,4,6-tri-*O*-benzyl-*β*-D-glucopyranosyl-(1→6)-2,3,4-tri-*O*-(2-naphthylmethyl)-*α*-D-glucopyranoside (34). According to the general protocol *C*, (3,4,6-tri-*O*-benzyl-*b*-D-glucopyranosyl)tri-*n*-butylstannane **10** (72.4 mg, 0.100 mmol), methyl 2,3,4-tri-*O*-(2-naphthylmethyl)-*α*-D-glucopyranoside (61.5 mg, 0.100 mmol), $Zn(OTf)_2$ (1.83 mg, 0.005 mmol), hydroxy(tosyloxy)iodobenzene (39.2 mg, 0.100 mmol), freshly activated 4Å MS and anh. CH_2Cl_2 (4.00 mL) were successively added into a vial. After stirring at room temperature for 12 h, (3,4,6-tri-*O*-benzyl-*β*-D-glucopyranosyl)tri-*n*-butylstannane[1] **10** (72.4 mg, 0.100 mmol) and hydroxy(tosyloxy)iodobenzene (39.2 mg, 0.100 mmol) were added and stirred for additional 12 h. (3,4,6-Tri-*O*-benzyl-*β*-D-glucopyranosyl)tri-*n*-butylstannane **10** (72.4 mg, 0.100 mmol) and hydroxy(tosyloxy)iodobenzene (39.2 mg, 0.100 mmol) were added and stirred at room temperature for 12 h and afforded after chromatographic purification on SiO_2 (Hexanes:EtOAc, 3:1) **34** (81.7 mg, 78%) as a colorless oil:$[\alpha]_D^{24}$ = -33.5 (c = 0.065, $CHCl_3$); IR (ATR) ν = 3517, 3059, 3033, 2906, 1458, 1368, 1070,

821, 743, 702 cm^{-1}; ^{1}H NMR (300 MHz, $CDCl_3$) δ 7.90 - 7.59 (m, 12H), 7.57 - 7.27 (m, 20H), 7.25 - 7.05 (m, 4H), 5.20 (d, *J* = 11.2 Hz, 1H), 5.10 - 4.74 (m, 8H), 4.69 (d, *J* = 3.5 Hz, 1H), 4.62 - 4.43 (m, 3H), 4.25 - 4.07 (m, 3H), 3.94 - 3.83 (m, 1H), 3.77 - 3.45 (m, 8H), 3.42 (s, 4H), 2.43 (s, 1H);^{13}C NMR (75 MHz, $CDCl_3$) δ 138.8, 138.3 (2), 136.4, 135.9, 135.6, 133.5, 133.4 (2), 133.3, 133.1, 128.6, 128.5 (3), 128.3, 128.2, 128.1 (2), 127.9, 127.8 (2), 127.7, 127.2, 126.6 (2), 126.3, 126.2 (3), 126.1, 126.0, 125.9, 103.6, 98.2, 84.6, 82.2, 79.9, 78.2, 77.4, 76.0, 75.5, 75.2, 75.1, 74.6, 73.6, 70.1, 69.1, 68.9, 55.5.; HRMS (ESI) *m/z* calcd for $C_{67}H_{66}O_{11}$ $[M + Na]^+$ 1069.4497, found 1069.4498.

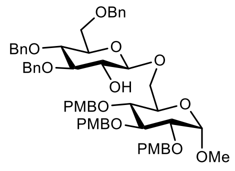

35

Methyl 3,4,6-tri-*O*-benzyl-*β*-D-glucopyranosyl-(1→6)-2,3,4-tri-O-(4-methoxybenzyl)-*α*-D-glucopyranoside (35). According to the general protocol *C*, (3,4,6-tri-*O*-benzyl-*β*-D-glucopyranosyl)tri-*n*-butylstannane **10** (72.4 mg, 0.100 mmol), methyl 2,3,4-tri-*O*-(4-methoxybenzyl)-*α*-D-glucopyranoside (55.5 mg, 0.100 mmol), $Zn(OTf)_2$ (1.83 mg, 0.005 mmol), hydroxy(tosyloxy)iodobenzene (39.2 mg, 0.100 mmol), freshly activated 4Å MS and anh. CH_2Cl_2 (4.00 mL) were successively added into a vial. After stirring at room temperature for 12 h, (3,4,6-tri-*O*-benzyl-*β*-D-glucopyranosyl)tri-*n*-butylstannane[1] **10** (72.4 mg, 0.100 mmol) and hydroxy(tosyloxy)iodobenzene (39.2 mg, 0.100 mmol) were added and stirred for additional 12 h. (3,4,6-Tri-*O*-benzyl-*β*-D-glucopyranosyl)tri-*n*-butylstannane **10** (72.4 mg, 0.100 mmol) and hydroxy(tosyloxy)iodobenzene (39.2 mg, 0.100 mmol) were added and stirred at room temperature for 12 h and afforded after chromatographic purification on SiO_2 (Hexanes:EtOAc, 1.5:1) **35** (52.3 mg, 53%) as a colorless oil: $[\alpha]_D^{24}$ = -8.40 (c = 0.100, $CHCl_3$); IR (ATR) ν = 3476, 2910, 2839, 1614, 1517, 1458, 1365, 1253, 1067, 825, 743,702 cm^{-1}; ^{1}H NMR (300 MHz, $CDCl_3$) δ 7.40 - 7.27 (m, 17H), 7.22 - 7.15 (m, 4H), 6.92 - 6.80 (m, 6H), 4.95 - 4.88 (m, 2H), 4.86 - 4.79 (m, 3H), 4.76 -4.71 (m, 2H), 4.62 - 4.50 (m, 7H), 4.23 (d, *J* = 5.6 Hz, 1H, C1H), 4.13 (dd, *J* = 11.0, 2.3 Hz, 1H), 3.95 (t, *J* = 9.2 Hz, 1H), 3.81 (m, 6H), 3.78 (s, 3H), 3.73 - 3.63 (m, 3H), 3.57 (dt, *J* = 4.9, 2.2 Hz, 3H), 3.52 - 3.41 (m, 3H), 3.37 (s, 3H), 2.48 (s, 1H); ^{13}C NMR (75 MHz, $CDCl_3$) δ 159.6, 159.4, 159.3, 138.8, 138.3 (2), 131.2, 130.6, 130.4, 129.9, 129.8, 129.6, 128.6, 128.5 (2), 128.1 (2), 127.9, 127.8 (2), 127.7, 114.0 (3), 103.7, 98.3, 84.6, 81.9, 79.6, 77.9, 75.6, 75.5, 75.2, 75.1, 74.8, 74.7, 73.6, 73.1, 70.0, 69.1, 69.0, 55.4; HRMS (ESI) *m/z* calcd for $C_{58}H_{66}O_{14}$ $[M + Na]^+$ 1009.4345, found 1009.4349.

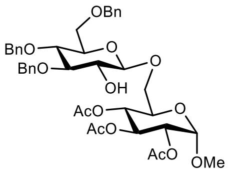

36

Methyl 3,4,6-tri-*O*-benzyl-*β*-D-glucopyranosyl-(1→6)-2,3,4-tri-O-acetyl-*α*-D-glucopyranoside (36). According to the general protocol *C*, (3,4,6-tri-*O*-benzyl–*β*–D-glucopyranosyl)tri-*n*-butylstannane **10** (72.4 mg, 0.100 mmol), methyl 2,3,4-tri-O-acetyl-α-D-glucopyranoside[8] (32.0 mg, 0.100 mmol), $Zn(OTf)_2$ (1.83 mg, 0.005 mmol), hydroxy(tosyloxy)iodobenzene (39.2 mg, 0.100 mmol), freshly activated 4Å MS and anh. CH_2Cl_2 (4.00 mL) were successively added into a vial. After stirring at room temperature for 12 h, (3,4,6-tri-*O*-benzyl–*β*–D-glucopyranosyl)tri-*n*-butylstannane **10** (72.4 mg, 0.100 mmol) and hydroxy(tosyloxy)iodobenzene (39.2 mg, 0.100 mmol) were added and stirred for additional 12 h. (3,4,6-Tri-*O*-benzyl–*β*–D-glucopyranosyl)tri-*n*-butylstannane **10** (72.4 mg, 0.100 mmol) and hydroxy(tosyloxy)iodobenzene (39.2 mg, 0.100 mmol) were added and stirred at room temperature for 12 h and afforded after chromatographic purification on SiO_2 (Hexanes:EtOAc, 1:1) **36** (67.0 mg, 89%) as a colorless foam: $[\alpha]_D^{24}$ = +24.7 (c = 1.00, $CHCl_3$); IR (ATR) ν = 3491, 3067, 3033, 2925, 2869, 1752, 1499, 1458, 1372, 1231, 1048, 743, 702 cm^{-1}; ^1H NMR (500 MHz, $CDCl_3$) δ 7.41 – 7.29 (m, 13H), 7.14 (dd, *J* = 7.4, 2.1 Hz, 2H), 5.15 (dd, *J* = 10.3, 9.3 Hz, 1H), 5.06 (d, *J* = 2.7 Hz, 1H), 4.97 (d, *J* = 3.6 Hz, 1H), 4.87 (d, *J* = 11.0 Hz, 1H), 4.84 – 4.79 (m, 3H), 4.62 (d, *J* = 12.0 Hz, 1H), 4.54 – 4.46 (m, 2H), 4.26 (d, *J* = 9.6 Hz, 2H), 4.23 (dd, *J* = 7.6, 4.8 Hz, 1H), 4.09 (dd, *J* = 12.4, 2.5 Hz, 1H), 3.94 – 3.85 (m, 2H), 3.76 (dd, *J* = 10.6, 2.9 Hz, 1H), 3.70 – 3.64 (m, 4H), 3.40 (s, 3H), 2.12 (s, 3H), 2.08 (s, 3H), 2.05 (s, 3H), 1.91 (d, *J* = 8.6 Hz, 1H); ^{13}C NMR (126 MHz, $CDCl_3$) δ 170.9, 170.4, 170.2, 138.7, 138.4, 138.0, 128.6, 128.5 (4), 128.1, 128.0, 127.9 (2), 127.8 (3), 100.2, 96.9, 82.8, 75.6 (2), 75.0, 73.8, 72.9, 72.2, 71.4, 70.4, 67.4, 55.5, 21.2, 21.1, 20.9; HRMS (ESI) *m/z* calcd for $C_{40}H_{48}O_{14}$ $[M + Na]^+$ 775.2942, found 775.2939.

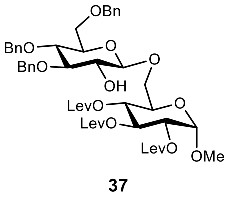

37

Methyl 3,4,6-tri-*O*-benzyl-*β*-D-glucopyranosyl-(1→6)-2,3,4-tri-*O*-levulinyl- *β*-D-glucopyranoside (37). According to the general protocol *C*, (3,4,6-tri-*O*-benzyl-*β*-D-glucopyranosyl)tri-*n*-butylstannane **10** (72.4 mg, 0.100 mmol), methyl 2,3,4-tri-*O*-levulinyl-*β*-D-glucopyranoside (48.9 mg, 0.100 mmol), $Zn(OTf)_2$ (1.83 mg, 0.005 mmol), hydroxy(tosyloxy)iodobenzene (39.2 mg, 0.100 mmol), freshly activated 4Å

MS and anh. CH_2Cl_2 (4.00 mL) were successively added into a vial. After stirring at room temperature for 12 h, (3,4,6-tri-*O*-benzyl-*β*-D-glucopyranosyl)tri-*n*-butylstannane[1] **10** (72.4 mg, 0.100 mmol) and hydroxy(tosyloxy)iodobenzene (39.2 mg, 0.100 mmol) were added and stirred for additional 12 h. (3,4,6-Tri-*O*-benzyl-*β*-D-glucopyranosyl)tri-*n*-butylstannane **10** (72.4 mg, 0.100 mmol) and hydroxy(tosyloxy)iodobenzene (39.2 mg, 0.100 mmol) were added and stirred at room temperature for 12 h and afforded after chromatographic purification on SiO_2 (Hexanes:EtOAc, 1:1) **37** (68.1 mg, 74%) as a colorless oil: $[\alpha]_D^{24}$= +27.2 (c = 1.1, $CHCl_3$); IR (ATR) ν = 3514, 3067, 3033, 2921, 1748, 1722, 1499, 1458, 1409, 1365, 1208, 1156, 1119, 1048 cm^{-1}; ^1H NMR (300 MHz, $CDCl_3$) δ 7.44 – 7.24 (m, 13H), 7.18 (dd, *J* = 7.0, 2.6 Hz, 2H), 5.56 (tt, *J* = 9.6, 1.9 Hz, 1H), 5.36 (t, *J* = 9.9 Hz, 1H), 5.05 (d, *J* = 11.2 Hz, 1H), 4.99 – 4.89 (m, 2H), 4.85 (d, *J* = 10.8 Hz, 1H), 4.79 (d, *J* = 11.2 Hz, 1H), 4.65 – 4.56 (m, 2H), 4.53 (d, *J* = 10.8 Hz, 1H), 4.24 (d, *J* = 7.1 Hz, 1H), 4.18 – 4.09 (m, 1H), 4.00 – 3.91 (m, 1H), 3.83 – 3.74 (m, 1H), 3.74 – 3.49 (m, 6H), 3.42 (s, 3H), 3.12 (d, *J* = 1.5 Hz, 1H), 2.82 – 2.51 (m, 12H), 2.20 – 2.14 (m, 9H); δ ^{13}C NMR (75 MHz, $CDCl_3$) δ 207.0, 206.6, 206.5, 172.7, 171.9(2), 139.1, 138.4(2), 128.5(2), 128.4, 128.2, 128.1, 127.8, 127.7, 103.1, 97.1, 85.0, 75.4, 75.2, 75.1(2), 73.5, 71.0, 69.9, 69.4, 68.2, 68.0, 67.3, 55.7, 38.0, 37.9, 29.9(2), 28.1, 28.0(2); HRMS (ESI) *m/z* calcd for $C_{49}H_{60}O_{17}$ $[M + Na]^+$ 943.3728, found 943.3726.

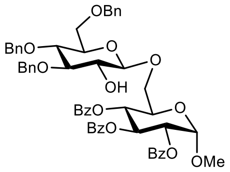

38

Methyl 3,4,6-tri-*O*-benzyl-*β*-D-glucopyranosyl-(1→6)-2,3,4-tri-O-benzoyl-*α*-D-glucopyranoside (38). According to the general protocol *C*, (3,4,6-tri-*O*-benzyl–*β*–D-glucopyranosyl)tri-*n*-butylstannane[1] **10** (72.4 mg, 0.100 mmol), methyl 2,3,4-tri-O-benzoyl-*α*-D-glucopyranoside[9] (50.7 mg, 0.100 mmol), $Zn(OTf)_2$ (1.83 mg, 0.005 mmol), hydroxy(tosyloxy)iodobenzene (39.2 mg, 0.100 mmol), freshly activated 4Å MS and anh. CH_2Cl_2 (4.00 mL) were successively added into a vial. After stirring at room temperature for 12 h, (3,4,6-tri-*O*-benzyl–*β*–D-glucopyranosyl)tri-*n*-butylstannane[1] **10** (72.4 mg, 0.100 mmol) and hydroxy(tosyloxy)iodobenzene (39.2 mg, 0.100 mmol) were added and stirred for additional 12 h. (3,4,6-Tri-*O*-benzyl–*β*–D-glucopyranosyl)tri-*n*-butylstannane **10** (72.4 mg, 0.100 mmol) and hydroxy(tosyloxy)iodobenzene (39.2 mg, 0.100 mmol) were added and stirred at room temperature for 12 h and afforded after chromatographic purification on SiO_2 (Hexanes:EtOAc, 2:1) **38** (56.3 mg, 60%) as a colorless foam: $[\alpha]_D^{24}$ = +34.4 (c = 0.20, $CHCl_3$); IR (ATR) ν = 3521, 3030, 2919, 1724, 1601, 1451, 1359, 1260, 1103, 1065, 707, 484 cm^{-1}; ^1H NMR (300 MHz, $CDCl_3$) δ 8.01 -7.76 (m, 4H), 7.90 - 7.81 (m, 2H), 7.59 - 7.48 (m, 2H), 7.47 - 7.27 (m, 20H), 7.17 (dt, *J* = 6.4, 2.3 Hz, 2H), 6.19 (ddd, *J* = 11.3, 9.7, 1.6 Hz, 1H), 5.82 (t, *J* = 9.9 Hz, 1H), 5.33 - 5.26 (m, 2H), 5.08 (d, *J* = 11.2

Hz, 1H), 4.85 (d, J = 10.8 Hz, 1H), 4.81 (d, J = 11.2 Hz, 1H), 4.68 - 4.44 (m, 4H), 4.30 - 4.16 (m, 3H), 3.79 - 3.53 (m, 6H), 3.48 (s, 3H), 3.27 (d, J = 1.7 Hz, 1H); ^{13}C NMR (75 MHz, $CDCl_3$) δ 166.1, 165.9 (2), 139.0, 138.3 (2), 133.8, 133.5, 133.2, 130.2, 130.1, 129.8, 129.3, 129.2, 128.8, 128.6 (2) , 128.5 (3), 128.4, 128.2, 128.1, 127.8, 127.7, 103.4, 97.3, 84.8, 77.4, 75.5, 75.3, 75.2 (2), 73.5, 72.2, 70.6, 69.3, 69.0, 68.4, 67.9, 55.9; HRMS (ESI) *m/z* calcd for $C_{55}H_{54}O_{14}$ [M + Na]$^+$ 961.3406, found 961.3408.

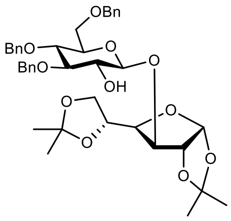

39

3,4,6-Tri-*O*-benzyl-*β*-D-glucopyranosyl-(1→3)-1,2:5,6-di-*O*-isopropylidene-*α*-D-glucofuranose (39). According to the general protocol *C*, (3,4,6-tri-*O*-benzyl-*β*-D-glucopyranosyl)tri-*n*-butylstannane **10** (72.4 mg, 0.100 mmol), 1,2:5,6-di-*O*-isopropylidene-*α*-D-glucofuranose (26.0 mg, 0.100 mmol), $Zn(OTf)_2$ (1.83 mg, 0.005 mmol), hydroxy(tosyloxy)iodobenzene (39.2 mg, 0.100 mmol), 2,4,6-tri-*tert*-butylpyrimidine (24.8 mg, 0.100 mmol), freshly activated 4Å MS and anh. CH_2Cl_2 (4.00 mL) were successively added into a vial. After stirring at room temperature for 12 h, (3,4,6-tri-*O*-benzyl-*β*-D-glucopyranosyl)tri-*n*-butylstannane[1] **10** (72.4 mg, 0.100 mmol), 2,4,6-tri-*tert*-butylpyrimidine (24.8 mg, 0.100 mmol), and hydroxy(tosyloxy)iodobenzene (39.2 mg, 0.100 mmol) were added and stirred for additional 12 h. (3,4,6-Tri-*O*-benzyl-*β*-D-glucopyranosyl)tri-*n*-butylstannane **10** (72.4 mg, 0.100 mmol), 2,4,6-tri-*tert*-butylpyrimidine (24.8 mg, 0.100 mmol), and hydroxy(tosyloxy)iodobenzene (39.2 mg, 0.100 mmol) were added and stirred at room temperature for 12 h and afforded after chromatographic purification on SiO_2 (Hexanes:EtOAc, 3:1) **39** (55.3 mg, 80%) as a colorless oil: $[\alpha]_D^{24}$= -15.3 (c = 0.3, $CHCl_3$); IR (ATR) ν = 3443, 3033, 2988, 2929, 1499, 1458, 1376, 1216, 1115, 1067, 1029, 851 cm^{-1}; ^{1}H NMR (300 MHz, $CDCl_3$) δ 7.49 – 7.12 (m, 15H), 5.91 (d, J = 3.6 Hz, 1H), 5.00 (d, J = 11.2 Hz, 1H), 4.91 – 4.79 (m, 2H), 4.73 (d, J = 3.7 Hz, 1H), 4.71 – 4.56 (m, 2H), 4.60 – 4.49 (m, 2H), 4.46 – 4.34 (m, 2H), 4.23 – 4.10 (m, 2H), 4.06 (dd, J = 8.8, 4.9 Hz, 1H), 3.97 (d, J = 3.0 Hz, 1H), 3.79 – 3.66 (m, 3H), 3.68 – 3.45 (m, 3H), 1.50 (s, 3H), 1.45 (s, 3H), 1.37 (s, 3H), 1.29 (s, 3H); ^{13}C NMR (75 MHz, $CDCl_3$) δ 139.0, 138.5, 138.3, 128.5(3), 128.2, 128.0, 127.9, 127.8, 127.7(2), 112.0, 109.6, 105.5, 101.7, 84.8, 84.6, 80.8, 77.3, 76.6, 76.3, 75.4, 75.2, 73.9, 73.1, 71.5, 69.2, 67.6, 27.0, 26.9, 26.4, 25.3; HRMS (ESI) *m/z* calcd for $C_{39}H_{48}O_{11}$ [M + Na]$^+$ 715.3094, found 715.3091.

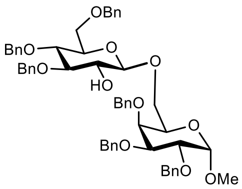

40

Methyl 3,4,6-tri-*O*-benzyl-*β*-D-glucopyranosyl-(1→6)-2,3,4-tri-O-benzyl-*α*-D-galactopyranoside (40). According to the general protocol *C*, (3,4,6-tri-*O*-benzyl–*β*–D-glucopyranosyl)tri-*n*-butylstannane[1] **10** (72.4 mg, 0.100 mmol), methyl 2,3,4-tri-O-benzoyl-α-D-galactopyranoside[8] (46.5 mg, 0.100 mmol), $Zn(OTf)_2$ (1.83 mg, 0.005 mmol), hydroxy(tosyloxy)iodobenzene (39.2 mg, 0.100 mmol), freshly activated 4Å MS and anh. CH_2Cl_2 (4.00 mL) were successively added into a vial. After stirring at room temperature for 12 h, (3,4,6-tri-*O*-benzyl–*β*–D-glucopyranosyl)tri-*n*-butylstannane **10** (72.4 mg, 0.100 mmol) and hydroxy(tosyloxy)iodobenzene (39.2 mg, 0.100 mmol) were added and stirred for additional 12 h. (3,4,6-Tri-*O*-benzyl–*β*–D-glucopyranosyl)tri-*n*-butylstannane **10** (72.4 mg, 0.100 mmol) and hydroxy(tosyloxy)iodobenzene (39.2 mg, 0.100 mmol) were added and stirred at room temperature for 12 h and afforded after chromatographic purification on SiO_2 (Hexanes:EtOAc, 2:1) **40** (73.6 mg, 82%) as a colorless foam: $[\alpha]_D^{24}$ = +18.1 (c = 1.00, $CHCl_3$); IR (ATR) ν = 3454, 3063, 3033, 2906, 1499, 1458, 1357, 1096, 1048, 739, 702 cm^{-1}; ^1H NMR (500 MHz, $CDCl_3$) δ 7.41 – 7.28 (m, 28H), 7.16 – 7.12 (m, 2H), 4.94 (t, *J* = 11.2 Hz, 2H), 4.88 – 4.81 (m, 4H), 4.73 (d, *J* = 11.8 Hz, 1H), 4.73 – 4.67 (m, 2H), 4.65 (d, *J* = 11.4 Hz, 1H), 4.57 (d, *J* = 12.1 Hz, 1H), 4.52 (d, *J* = 10.8 Hz, 1H), 4.47 (d, *J* = 12.1 Hz, 1H), 4.28 (d, *J* = 7.6 Hz, 1H, C1H), 4.04 (dd, *J* = 10.0, 3.6 Hz, 1H), 3.96 – 3.91 (m, 2H), 3.88 – 3.84 (m, 2H), 3.76 – 3.69 (m, 3H), 3.67 – 3.62 (m, 1H), 3.56 (t, *J* = 8.9 Hz, 1H), 3.50 (dd, *J* = 9.1, 7.6 Hz, 1H), 3.45 (ddd, *J* = 9.8, 4.0, 2.3 Hz, 1H), 3.39 (s, 3H); ^{13}C NMR (126 MHz, $CDCl_3$) δ 138.9, 138.7, 138.5, 138.2, 138.1, 128.6, 128.5 (2), 128.4 (2), 128.3, 128.2, 128.1, 128.0, 127.9 (3), 127.8 (2), 127.7 (2), 127.6, 103.5, 98.9, 84.5, 79.1, 76.4, 75.2 (2), 75.1, 75.0, 74.7, 73.7, 73.6, 73.5, 70.0, 69.7, 68.8, 55.6; HRMS (ESI) *m/z* calcd for $C_{55}H_{60}O_{11}$ $[M + Na]^+$ 919.4033, found 919.4026.

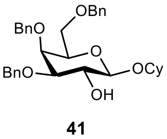

41

Cyclohexyl 3,4,6-tri-*O*-benzyl–*β*–D-galactopyranoside (41). According to the general protocol *B*, (3,4,6-tri-*O*-benzyl–*β*–D-galactopyranosyl)tri-*n*-butylstannane[1] (145 mg, 0.200 mmol), cyclohexanol (10.4 μl, 0.100 mmol), $Zn(OTf)_2$ (7.30 mg, 0.020 mmol), iodosobenzene (66.0 mg, 0.300 mmol), freshly activated 4Å MS, and anh. $CHCl_3$ (1.00 mL) were successively added into a vial. The reaction mixture was stirred at room temperature for 24 h and afforded after chromatographic purification on SiO_2 (Hexanes:EtOAc, 8:1) **41** (38.4 mg, 72%) as a colorless oil: $[\alpha]_D^{24}$ = -3.4 (c = 0.30,

$CHCl_3$); IR (ATR) ν = 3469, 3029, 2930, 2856, 1495, 1452, 1364, 1075, 1070, 735, 697 cm^{-1}; ^1H NMR (300 MHz, $CDCl_3$) δ 7.41 - 7.26 (m, 15H), 4.89 (d, *J* = 11.6 Hz, 1H), 4.72 (s, 2H), 4.62 (d, *J* = 11.7 Hz, 1H), 4.51 - 4.40 (m, 2H), 4.32 (d, *J* = 7.7 Hz, 1H, C1H), 3.99 - 3.88 (m, 2H), 3.69 - 3.52 (m, 4H), 3.44 (dd, *J* = 9.8, 2.9 Hz, 1H), 2.30 (d, *J* = 1.9 Hz, 1H), 1.94 (t, *J* = 11.9 Hz, 2H), 1.72 (t, *J* = 5.7 Hz, 2H), 1.53 - 1.12 (m, 6H); ^{13}C NMR (75 MHz, $CDCl_3$) δ 138.7, 138.4, 138.1, 128.6 (2), 128.5, 128.3, 128.0, 127.9, 127.8 (2), 127.7, 101.7, 82.2, 74.7, 74.0, 73.7, 73.3, 72.7, 71.6, 69.1, 33.7, 32.0, 25.7, 24.4, 24.2; HRMS (ESI) *m/z* calcd for $C_{33}H_{40}O_6$ [M + Na]$^+$ 555.2717, found 555.2717.

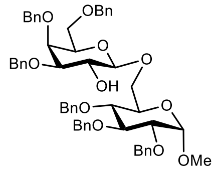

42

Methyl 3,4,6-tri-*O*-benzyl–*β*–D-galactopyranosyl-(1→6)-2,3,4-tri-*O*-benzyl–*α*-D-glucopyranoside (42). According to the general protocol *C*, (3,4,6-tri-*O*-benzyl–*β*–D-galactopyranosyl)tri-*n*-butylstannane[1] (72.4 mg, 0.100 mmol), methyl 2,3,4-tri-*O*-benzyl-α-D-glucopyranoside[7] (46.5 mg, 0.1 mmol), $Zn(OTf)_2$ (1.83 mg, 0.005 mmol), hydroxy(tosyloxy)iodobenzene (39.2 mg, 0.100 mmol), freshly activated 4Å MS, and anh. CH_2Cl_2 (4.00 mL) were successively added into a vial. After stirring at room temperature for 12 h, (3,4,6-tri-*O*-benzyl–*β*–D-galactopyranosyl)tri-*n*-butylstannane (72.4 mg, 0.100 mmol) and hydroxy(tosyloxy)iodobenzene (39.2 mg, 0.100 mmol) were added and stirred for additional 12 h. (3,4,6-Tri-*O*-benzyl–*β*–D-galactopyranosyl)tri-*n*-butylstannane (72.4 mg, 0.100 mmol) and hydroxy(tosyloxy)iodobenzene (39.2 mg, 0.100 mmol) were added and stirred at room temperature for 12 h and afforded after chromatographic purification on SiO_2 (Hexanes:EtOAc, 2:1) **42** (60.1 mg, 67%) as a colorless oil: $[\alpha]_D^{24}$ = +12.9 (c = 0.50, $CHCl_3$); IR (ATR) ν = 3458, 3029, 2912, 1495, 1453, 1360, 1067, 909, 733, 696, 461 cm^{-1}; ^1H NMR (300 MHz, $CDCl_3$) δ 7.43 - 7.19 (m, 30H), 4.98 (d, *J* = 10.9 Hz, 1H), 4.89 (d, *J* = 11.4 Hz, 1H), 4.88 (d, *J* = 11.0 Hz, 1H), 4.84 - 4.75 (m, 2H), 4.74 - 4.54 (m, 6H), 4.50 - 4.38 (m, 2H), 4.23 (d, *J* = 7.7 Hz, 1H, C1H), 4.12 (dd, *J* = 11.0, 2.2 Hz, 1H), 4.06 - 3.90 (m, 3H), 3.82 (ddd, *J* = 10.1, 5.3, 2.1 Hz, 1H), 3.72 - 3.46 (m, 6H), 3.41 (dd, *J* = 9.8, 2.8 Hz, 1H), 3.37 (s, 3H), 2.52 (s, 1H); ^{13}C NMR (75 MHz, $CDCl_3$) δ 138.9, 138.8, 138.5, 138.3, 138.2, 138.0, 128.6 (3), 128.5 (2), 128.3 (2), 128.1 (2), 128.0 (2), 127.9, 127.8, 127.7 (2), 127.6, 104.2, 98.1, 82.2, 82.0, 79.9, 78.2, 75.9, 75.1, 74.7, 73.9, 73.7, 73.5, 73.2, 72.5, 71.1, 70.0, 68.7, 68.6, 55.4; HRMS (ESI) *m/z* calcd for $C_{55}H_{60}O_{11}$ [M + Na]$^+$ 919.4028, found 919.4022.

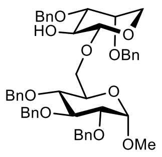

43

Methyl 3,4-di-*O*-benzyl–*β*–D-arabinopyranosyl-(1→6)-2,3,4-tri-O-benzyl-*α*-D-glucopyranoside (**43**). According to the general protocol *C*, (3,4-di-*O*-benzyl-*β*-D-arabinosyl)tri-n-butylstannane **S5** (60.4 mg, 0.100 mmol), methyl 2,3,4-tri-*O*-benzyl-α-D-glucopyranoside[7] (46.5 mg, 0.100 mmol), $Zn(OTf)_2$ (1.83 mg, 0.005 mmol), hydroxy(tosyloxy)iodobenzene (39.2 mg, 0.100 mmol), freshly activated 4Å MS and anh. CH_2Cl_2 (4.00 mL) were successively added into a vial. After stirring at room temperature for 12 h, (3,4-di-*O*-benzyl–*β*–D-arabinosyl)tri-*n*-butylstannane **S5** (60.4 mg, 0.100 mmol) and hydroxy(tosyloxy)iodobenzene (39.2 mg, 0.100 mmol) were added and stirred for additional 12 h. (3,4-Di-*O*-benzyl-*β*-D-arabinosyl)tri-*n*-butylstannane **S5** (60.4 mg, 0.100 mmol) and hydroxy(tosyloxy)iodobenzene (39.2 mg, 0.100 mmol) were added and stirred at room temperature for 12 h and afforded after chromatographic purification on SiO_2 (Hexanes:EtOAc, 1.5:1) **43** (55.9 mg, 72%) as a colorless oil: $[\alpha]_D^{23}$ = -12.3 (c = 0.40, $CHCl_3$); IR (ATR) = 3454, 3020, 2910, 1491, 1452, 1364, 1062, 910, 735, 696, 463, cm^{-1}; ^{1}H NMR (300 MHz, $CDCl_3$) δ 7.43 - 7.27 (m, 25H), 4.99 (d, *J* = 10.9 Hz, 1H), 4.85 (dd, *J* = 10.9, 8.8 Hz, 2H), 4.76 (d, *J* = 8.8 Hz, 1H), 4.74 - 4.57 (m, 7H), 4.27 (d, *J* = 7.4 Hz, 1H, C1H), 4.15 (dd, *J* = 11.5, 4.2 Hz, 1H), 4.08 - 3.93 (m, 3H), 3.81 (dd, *J* = 11.4, 2.0 Hz, 1H), 3.77 - 3.66 (m, 2H), 3.66 - 3.58 (m, 1H), 3.55 (dd, *J* = 9.6, 3.5 Hz, 1H), 3.44 - 3.34 (m, 1H), 3.38 (s, 3H), 3.28 (dd, *J* = 13.0, 1.2 Hz, 1H), 2.57 (s, 1H); ^{13}C NMR (75 MHz, $CDCl_3$) δ 138.9, 138.6, 138.3 (2), 128.6, 128.5 (3), 128.2, 128.1, 128.0 (3) , 127.8, 127.7 (2), 103.8, 98.3, 82.2, 80.3, 80.1, 77.6, 75.8, 75.1, 73.6, 72.2, 72.0, 71.4, 71.0, 70.1, 67.4, 63.7, 55.4; HRMS (ESI) *m/z* calcd for $C_{47}H_{52}O_{10}$ $[M + Na]^+$ 799.3453, found 799.3459.

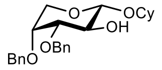

44

Cyclohexyl 3,4-di-*O*-benzyl-*β*-D-arabinopyranoside (44). According to the general protocol *B*, (3,4-Di-*O*-benzyl-*β*-D-arabinosyl)tri-*n*-butylstannane **S5** (121 mg, 0.200 mmol), cyclohexanol (10.4 μL, 0.1 mmol), $Zn(OTf)_2$ (7.30 mg, 0.020 mmol), iodosobenzene (66.0 mg, 0.300 mmol), freshly activated 4Å MS, and anh. $CHCl_3$ (1.00 mL) were successively added into a vial. The reaction mixture was stirred at room temperature for 24 h and afforded after chromatographic purification on SiO_2 (Hexanes:EtOAc, 5:1) **44** (23.5 mg, 57%) as a colorless foam: $[\alpha]_D^{23}$ = -10.8 (c = 0.50, $CHCl_3$); IR (ATR) ν = 3466, 2927, 2855, 1727, 1452, 1363, 1253, 1093, 735, 698 cm^{-1}; ^{1}H NMR (300 MHz, $CDCl_3$) δ 7.42 - 7.27 (m, 10H), 4.78 (d, *J* = 12.6 Hz, 1H), 4.69 - 4.59 (m, 3H), 4.26 (d, *J* = 7.6 Hz, 1H, C1H), 4.05 (dd, *J* = 12.9, 2.2 Hz, 1H), 3.94 (ddd, *J* = 9.4, 7.5, 1.8 Hz, 1H), 3.72 - 3.60 (m, 2H), 3.40 (dd, *J* = 9.6, 3.3 Hz, 1H), 3.28 (dd, *J* = 12.9, 1.2 Hz, 1H), 2.32 (d, *J* = 2.0 Hz, 1H), 1.95 (s, 2H), 1.74 (s, 2H), 1.45 -

1.16 (m, 6H); ^{13}C NMR (75 MHz, $CDCl_3$) δ 138.4, 128.6, 128.5, 128.1, 127.8 (3), 101.9, 80.5, 77.3, 72.2, 72.0, 71.5, 71.3, 63.9, 33.8, 32.0, 25.7, 24.4, 24.3; HRMS (ESI) *m/z* calcd for $C_{25}H_{32}O_5$ $[M + Na]^+$435.2142, found 435.2144.

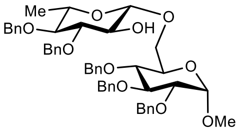

45

Methyl 3,4-di-*O*-benzyl-6-deoxy-*β*-D-glucopyranosyl-(1→6)-2,3,4-tri-O-methyl-*α*-D-glucopyranoside (45). According to the general protocol *C*, (3,4-di-*O*-benzyl-*β*-D-quinovopyranosyl)tri-*n*-butylstannane[10] (61.7 mg, 0.100 mmol), methyl 2,3,4-tri-*O*-benzyl-α-D-glucopyranoside[7] (46.5 mg, 0.100 mmol), $Zn(OTf)_2$ (1.83 mg, 0.005 mmol), hydroxy(tosyloxy)iodobenzene (39.2 mg, 0.100 mmol), freshly activated 4Å MS, and anh. CH_2Cl_2 (4.00 mL) were successively added into a vial. After stirring at room temperature for 12 h, (3,4-di-*O*-benzyl-*β*-D-quinovopyranosyl)tri-*n*-butylstannane (61.7 mg, 0.100 mmol) and hydroxy(tosyloxy)iodobenzene (39.2 mg, 0.100 mmol) were added and stirred for additional 12 h. (3,4-Di-*O*-benzyl-*β*–D-quinovopyranosyl)tri-n-butylstannane (61.7 mg, 0.100 mmol) and hydroxy(tosyloxy)iodobenzene (39.2 mg, 0.100 mmol) were added and stirred at room temperature for 12 h and afforded after chromatographic purification on SiO_2 (Hexanes:EtOAc, 2:1) **45** (41.9 mg, 53%) as a colorless oil: $[\alpha]_D^{23}$ = -23.0 (c = 0.25, $CHCl_3$); IR (ATR) ν = 3433, 2924, 2359, 1731, 1453, 1453, 1361, 1069, 736, 698 cm^{-1}; ^1H NMR (300 MHz, $CDCl_3$) δ 7.41 - 7.26 (m, 25H), 4.99 (d, *J* = 10.7 Hz, 1H), 4.94 - 4.75 (m, 7H), 4.71 - 4.53 (m, 4H), 3.99 (t, *J* = 9.2 Hz, 1H), 3.89 (d, *J* = 10.7 Hz, 1H), 3.77 (dd, *J* = 9.6, 6.1 Hz, 2H), 3.65 (d, *J* = 6.8 Hz, 2H), 3.52 (td, *J* = 10.2, 9.7, 4.6 Hz, 2H), 3.42 (d, *J* = 9.5 Hz, 1H), 3.36 (s, 3H), 3.10 (t, *J* = 9.0 Hz, 1H), 2.04 (d, *J* = 7.8 Hz, 1H), 1.23 (d, *J* = 6.3 Hz, 3H); ^{13}C NMR (75 MHz, $CDCl_3$) δ 138.8 (2), 138.4, 138.3, 138.2, 128.7, 128.6 (3), 128.3, 128.2, 128.1 (2), 128.0, 127.8 (2), 99.1, 98.1, 83.5, 83.1, 82.2, 80.1, 78.2, 76.0, 75.5, 75.4, 75.2, 73.7, 73.5, 70.2, 67.4, 67.3, 55.4, 17.9; HRMS (ESI) *m/z* calcd for $C_{48}H_{54}O_{10}$ $[M + Na]^+$ 813.3609, found 813.3616.

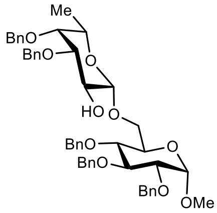

46

Methyl 3,4-di-*O*-benzyl-*β*-D-fucopyranosyl-(1→6)-2,3,4-tri-O-methyl–*α*-D-glucopyranoside (46). According to the general protocol *C*, (3,4-di-*O*-benzyl–*β*-L-fucopyranosyl)tri-*n*-butylstannane **S6** (61.7 mg, 0.100 mmol), methyl 2,3,4-tri-*O*-benzyl-α-D-glucopyranoside[7] (46.5 mg, 0.100 mmol), $Zn(OTf)_2$ (1.83 mg, 0.005 mmol), hydroxy(tosyloxy)iodobenzene (39.2 mg, 0.100 mmol), freshly activated 4Å MS, and anh. CH_2Cl_2 (4.00 mL) were successively added into a vial. After stirring at

room temperature for 12 h, (3,4-di-*O*-benzyl–*β*-L- fucopyranosyl)tri-*n*-butylstannane **S6** (61.7 mg, 0.100 mmol) and hydroxy(tosyloxy)iodobenzene (39.2 mg, 0.100 mmol) were added and stirred for additional 12 h. (3,4-Di-*O*-benzyl–*β*-L-fucopyranosyl)tri-*n*-butylstannane **S6** (61.7 mg, 0.100 mmol) and hydroxy(tosyloxy)iodobenzene (39.2 mg, 0.100 mmol) were added and stirred at room temperature for 12 h and afforded after chromatographic purification on SiO_2 (Hexanes:EtOAc, 1.5:1) **46** (36.4 mg, 46%) as a colorless oil: $[\alpha]_D^{24}$ = +4.8 (c = 0.50, $CHCl_3$); IR (ATR) ν = 3432, 3029, 2872, 1495, 1453, 1358, 1166, 1065, 909, 732, 696, 461 cm^{-1}; ^1H NMR (300 MHz, $CDCl_3$) δ 7.42 - 7.21 (m, 30H), 4.95 (dd, *J* = 11.3, 9.8 Hz, 2H), 4.88 - 4.79 (m, 2H), 4.78 - 4.73 (m, 3H), 4.72 - 4.60 (m, 4H), 4.28 (d, *J* = 7.7 Hz, 1H, C1H), 4.14 (dd, *J* = 11.8, 4.3 Hz, 1H), 3.97 (td, *J* = 8.5, 7.7, 3.3 Hz, 2H), 3.82 - 3.70 (m, 2H), 3.66 - 3.42 (m, 5H), 3.37 (s, 3H), 2.63 (s, 1H), 1.18 (d, *J* = 6.4 Hz, 3H); ^{13}C NMR (75 MHz, $CDCl_3$) δ 139.0, 138.7, 138.5, 138.3, 128.6, 128.6, 128.5, 128.4, 128.4, 128.3, 128.2, 128.1, 128.0, 127.8, 127.8, 127.7, 127.7, 103.5, 98.3, 82.5, 82.1, 80.1, 77.7, 76.2, 75.8, 75.1, 74.8, 73.6, 72.8, 71.3, 71.0, 70.1, 67.4, 55.4, 17.1; HRMS (ESI) *m/z* calcd for $C_{48}H_{54}O_{10}$ $[M + Na]^+$ 813.3609, found 813.3603.

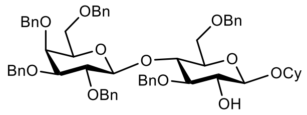

47

Cyclohexyl 2,3,4,6-tetra-*O*-benzyl-*β*-D-galactopyranosyl-(1→4)-3,6-di-O-benzyl-*α*–D-glucopyranoside (47). According to the general protocol *B*, [2,3,4,6-tetra-*O*-benzyl–*β*–D-galactopyranosyl-(1→4)-3,6-di-*O*-benzyl-*β*-D-glucopyranosyl]tri-*n*-butylstannane[1] **10** (231.2 mg, 0.200 mmol), cyclohexanol (10.4 μl, 0.100 mmol), $Zn(OTf)_2$ (7.30 mg, 0.020 mmol), iodosobenzene (66.0 mg, 0.300 mmol), freshly activated 4Å MS, and anh. $CHCl_3$ (1.00 mL) were successively added into a vial. The reaction mixture was stirred at room temperature for 24 h and afforded after chromatographic purification on SiO_2 (Hexanes:EtOAc, 5:1) **47** (68.5 mg, 71%) as a colorless foam:$[\alpha]_D^{24}$ = -1.1 (c = 0.40, $CHCl_3$); IR (ATR) ν = 3453, 3029, 2928, 2856, 1495, 1452, 1361, 1207, 1060, 731, 704, 462 cm^{-1}; ^1H NMR (300 MHz, $CDCl_3$) δ 7.41 - 7.16 (m, 30H), 5.05 (d, *J* = 11.2 Hz, 1H), 4.97 (d, *J* = 11.5 Hz, 1H), 4.83 (d, *J* = 11.2 Hz, 1H), 4.81 - 4.66 (m, 4H), 4.61 - 4.33 (m, 6H), 4.27 (d, *J* = 11.7 Hz, 1H), 3.97 - 3.86 (m, 2H), 3.84 - 3.72 (m, 3H), 3.71 - 3.35 (m, 8H), 2.36 (s, 1H), 2.02 - 1.91 (m, 2H), 1.78 - 1.63 (m, 2H), 1.55 - 1.14 (m, 6H); ^{13}C NMR (75 MHz, $CDCl_3$) δ 139.3, 139.1, 138.9, 138.6 (2), 138.1, 128.5 (2), 128.4, 128.3 (2), 128.0 (2), 127.9, 127.8, 127.7, 127.6, 127.5 (2), 127.4, 103.0, 101.1, 83.0, 82.7, 80.1, 77.8, 75.6, 75.4, 74.8, 74.7, 73.8, 73.7, 73.6, 73.2, 72.7, 68.6, 68.3, 33.8, 32.2, 25.7, 24.4, 24.3; HRMS (ESI) *m/z* calcd for $C_{60}H_{68}O_{11}$ $[M + Na]^+$987.4654, found 987.4666.

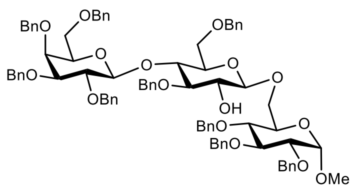

48

Methyl 2,3,4,6-tetra-*O*-benzyl–*β*–D-galactopyranosyl-(1→4)-3,6-di-*O*-benzyl - *β*–D-glucopyranosyl-(1→6)-2,3,4-tri-*O*-benzyl–*α*-D-glucopyranoside (48). According to the general protocol *C*, [2,3,4,6-tetra-*O*-benzyl-*β*-D-galactopyranosyl-(1→4)-3,6-di-*O*-benzyl-*β*–D-glucopyranosyl]tri-*n*-butylstannane[1] (116 mg, 0.100 mmol), methyl 2,3,4-tri-*O*-benzyl-α-D-glucopyranoside[7] (46.5 mg, 0.100 mmol), Zn(OTf)$_2$ (1.83 mg, 0.005 mmol), hydroxy(tosyloxy)iodobenzene (39.2 mg, 0.100 mmol), freshly activated 4Å MS and anh. CH_2Cl_2 (4.00 mL) were successively added into a vial. After stirring at room temperature for 12 h, [2,3,4,6-tetra-*O*-benzyl-*β*-D-galactopyranosyl-(1→4)-3,6-di-*O*-benzyl-*β*-D-glucopyranosyl]tri-*n*-butylstannane[1] (115.6 mg, 0.100 mmol) and hydroxy(tosyloxy)iodobenzene (39.2 mg, 0.100 mmol) were added and stirred for additional 12 h. [2,3,4,6-tetra-*O*-benzyl-*β*-D-galactopyranosyl-(1→4)-3,6-di-*O*-benzyl-*β*-D-glucopyranosyl]tri-*n*-butylstannane[1] (115.6 mg, 0.100 mmol) and hydroxy(tosyloxy)iodobenzene (39.2 mg, 0.100 mmol) were added and stirred at room temperature for 12 h and afforded after chromatographic purification on SiO_2 (Hexanes:EtOAc, 1.5:1) **48** (83.8 mg, 63%) as a colorless oil: $[\alpha]_D^{24}$ = +15.3 (c = 1.00, $CHCl_3$); IR (ATR) ν = 3461, 3029, 2865, 1602, 1495, 1452, 1360, 1207, 1048, 908, 731, 695 cm^{-1}; ^{1}H NMR (300 MHz, $CDCl_3$) δ 7.47 - 7.17 (m, 45H), 5.09 (d, *J* = 11.1 Hz, 1H), 5.01 (dd, *J* = 11.1, 5.9 Hz, 2H), 4.93 (d, *J* = 11.0 Hz, 1H), 4.91 - 4.79 (m, 4H), 4.79 - 4.62 (m, 6H), 4.59 (d, *J* = 11.2 Hz, 1H), 4.54 (d, *J* = 12.2 Hz, 1H), 4.48 (d, *J* = 7.6 Hz, 1H), 4.40 (d, *J* = 12.0 Hz, 2H), 4.30 (dd, *J* = 9.7, 2.0 Hz, 2H), 4.15 (dd, *J* = 11.0, 2.0 Hz, 1H), 4.09 - 3.90 (m, 3H), 3.89 - 3.63 (m, 6H), 3.63 - 3.43 (m, 6H), 3.41 (s, 3H), 3.44 - 3.35 (m, 2H), 2.51 (s, 1H); ^{13}C NMR (75 MHz, $CDCl_3$) ^{13}C NMR (75 MHz, $CDCl_3$) δ 139.1, 138.9 (2), 138.6 (2), 138.5, 138.2, 138.1, 128.6 (2), 128.5, 128.5 (3), 128.3 (2), 128.2 (2), 128.1, 128.0 (2), 127.9 (2), 127.8 (2), 127.7 (2), 127.6, 127.5 (2), 127.4 (2), 103.5, 102.9, 98.2, 82.9, 82.6, 82.2, 80.1, 79.9, 78.0, 76.3, 75.9, 75.8, 75.4, 75.1, 74.8, 73.7, 73.6, 73.5, 73.4, 73.2, 72.7, 70.8, 70.0, 68.6, 68.4, 68.3, 62.0, 55.4; HRMS (ESI) *m/z* calcd for $C_{82}H_{88}O_{16}$ [M + Na]$^+$ 1351.5965, found 1351.5945.

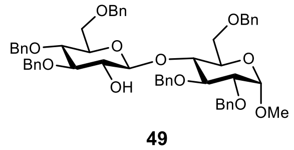

49

Methyl 3,4,6-tri-*O*-benzyl-*β*-D-glucopyranosyl-(1→4)-2,3,6-tri-O-benzyl-*α*-D-glucopyranoside (**49**). According to the general protocol *C*, (3,4,6-tri-*O*-benzyl–*β*–D-glucopyranosyl)tri-*n*-butylstannane **10** (72.4 mg, 0.100 mmol), methyl 2,3,6-tri-*O*-benzyl-α-D-glucopyranoside[11] (46.5 mg, 0.100 mmol), Zn(OTf)$_2$ (1.83 mg, 0.005 mmol), 3,5-bis(trifluoromethyl)-1-hydroxy(tosyloxy)iodobenzene (52.8 mg, 0.100

mmol), freshly activated 4Å MS and anh. CH_2Cl_2 (4.00 mL) were successively added into a vial. After stirring at room temperature for 12 h, (3,4,6-tri-*O*-benzyl–*β*–D-glucopyranosyl)tri-*n*-butylstannane (72.4 mg, 0.100 mmol), 3,5-bis(trifluoromethyl)-1-hydroxy(tosyloxy)iodobenzene (52.8 mg, 0.100 mmol) were added and stirred for additional 12 h. (3,4,6-tri-*O*-benzyl–*β*–D-glucopyranosyl)tri-*n*-butylstannane **10** (72.4 mg, 0.100 mmol) and 3,5-bis(trifluoromethyl)-1-hydroxy(tosyloxy)iodobenzene (52.8 mg, 0.100 mmol) were added and stirred at room temperature for 12 h and afforded after chromatographic purification on SiO_2 (Hexanes:EtOAc, 3:1) **49** (76.0 mg, 85%) as a colorless oil: $[\alpha]_D^{24}$ = +14.6 (c = 1.00, $CHCl_3$); IR (ATR) ν = 3480, 3067, 3033, 2925, 2869, 1499, 1458, 1365, 1111, 1055, 739, 702 cm^{-1}; ^1H NMR (500 MHz, $CDCl_3$) δ 7.39 – 7.27 (m, 28H), 7.15 (dd, *J* = 7.3, 2.2 Hz, 2H), 5.03 (d, *J* = 11.4 Hz, 1H), 4.92 – 4.87 (m, 2H), 4.83 – 4.76 (m, 2H), 4.74 (d, *J* = 12.2 Hz, 1H), 4.66 (d, *J* = 12.0 Hz, 1H), 4.61 – 4.49 (m, 5H), 4.44 (d, *J* = 12.1 Hz, 1H), 4.40 (d, *J* = 12.1 Hz, 1H), 4.01 – 3.96 (m, 3H), 3.80 (td, *J* = 6.5, 3.0 Hz, 1H), 3.67 (dd, *J* = 11.2, 2.3 Hz, 1H), 3.63 – 3.58 (m, 1H), 3.52 – 3.41 (m, 4H), 3.37 (s, 3H), 3.22 (ddd, *J* = 9.9, 3.8, 2.3 Hz, 1H), 2.06 (s, 1H); ^{13}C NMR (126 MHz, $CDCl_3$) δ 139.4, 138.9, 138.4, 138.3, 138.2, 137.5, 129.7, 128.7, 128.6, 128.5 (4), 128.4, 128.3, 128.2 (2), 128.1, 128.0 (3), 127.8 (3), 127.6, 127.2 (2), 127.1, 103.3, 98.3, 84.5, 81.1, 79.6, 77.0, 75.7, 75.3, 75.2, 75.1, 75.0, 73.9, 73.6, 73.4, 69.6, 68.8, 68.6, 55.4; HRMS (ESI) *m/z* calcd for $C_{55}H_{60}O_{11}$ $[M + Na]^+$ 919.4033, found 919.4020.

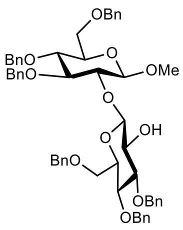

50

Methyl 3,4,6-tri-*O*-benzyl–*β*–D-glucopyranosyl-(1→2)-3,4,6-tri-*O*-benzyl-*β*-D-glucopyranoside (**50**). According to the general protocol *C*, (3,4,6-tri-*O*-benzyl–*β*–D-glucopyranosyl)tri-*n*-butylstannane **10** (72.4 mg, 0.100 mmol), methyl 3,4,6-tri-*O*-benzyl-*α*-D-glucopyranoside[12] (46.5 mg, 0.100 mmol), $Zn(OTf)_2$ (1.8 mg, 0.005 mmol), 3,5-bis(trifluoromethyl) -1-hydroxy(tosyloxy)iodobenzene (52.8 mg, 0.100 mmol), freshly activated 4Å MS and anh. CH_2Cl_2 (4.00 mL) were successively added into a vial. After stirring at room temperature for 12 h, (3,4,6-tri-*O*-benzyl–*β*–D-glucopyranosyl)tri-*n*-butylstannane **10** (72.4 mg, 0.100 mmol) and 3,5-bis(trifluoromethyl)-1-hydroxy(tosyloxy)iodobenzene (52.8 mg, 0.100 mmol) were added and stirred for additional 12 h. (3,4,6-tri-*O*-benzyl–*β*–D-glucopyranosyl)tri-*n*-butylstannane **10** (72.4 mg, 0.100 mmol) and 3,5-bis(trifluoromethyl)-1-hydroxy(tosyloxy)iodobenzene (52.8 mg, 0.100 mmol) were added and stirred at room temperature for 12 h and afforded after chromatographic purification on SiO_2 (Hexanes:EtOAc, 2:1) **50** (53.0 mg, 59%) as a colorless oil: $[\alpha]_D^{24}$ = -0.9 (c = 1.00, $CHCl_3$); IR (ATR) ν = 3473, 3067, 3033, 2903, 2869, 1499, 1458, 1365, 1067, 739, 702 cm^{-1}; ^1H NMR (500 MHz, $CDCl_3$) δ 7.40 – 7.27 (m, 26H), 7.22 – 7.15 (m, 4H),

4.95 (d, J = 10.7 Hz, 1H), 4.90 – 4.82 (m, 3H), 4.82 (d, J = 10.8 Hz, 1H), 4.73 (d, J = 11.1 Hz, 1H), 4.68 – 4.57 (m, 3H), 4.57 (d, J = 12.2 Hz, 0H), 4.55 – 4.49 (m, 3H), 4.36 (d, J = 7.6 Hz, 1H, C1H), 3.79 – 3.70 (m, 5H), 3.68 – 3.60 (m, 3H), 3.57 – 3.53 (m, 1H), 3.52 (s, 3H), 3.53 – 3.44 (m, 3H), 3.03 (d, J = 2.2 Hz, 1H); ^{13}C NMR (126 MHz, $CDCl_3$) δ 138.9, 138.3, 138.2, 137.8 (2), 128.7, 128.6, 128.5 (3), 128.4, 128.3, 128.2, 128.1, 128.0 (2), 127.9 (2), 127.8 (2), 127.7, 127.6, 104.1, 103.7, 84.3, 84.1, 81.3, 78.5, 76.3, 76.2, 75.6, 75.1, 75.0 (2), 74.9, 73.6, 73.5, 69.1, 68.7, 57.6; HRMS (ESI) *m/z* calcd for $C_{55}H_{60}O_{11}$ $[M + Na]^+$ 919.4033, found 919.4037.

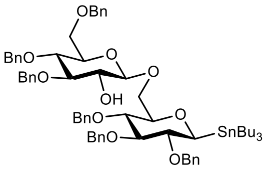

52

[3,4,6-Tri-*O*-benzyl-*β*-D-glucopyranosyl-(1→6)-2,3,4-tri-*O*-benzyl-*β*-D-glucopyranosyl]tri-*n*-butylstannane (**52**).[10] According to the general protocol *C*, (3,4,6-tri-*O*-benzyl–*β*–D-glucopyranosyl)tri-*n*-butylstannane **10** (72.4 mg, 0.100 mmol), (2,3,4-tri-*O*-benzyl-*β*-D-glucopyranosyl)tri-*n*-butylstannane[1] **51** (72.4 mg, 0.100 mmol), $Zn(OTf)_2$ (1.83 mg, 0.005 mmol), hydroxy(tosyloxy)iodobenzene (39.2 mg, 0.100 mmol), freshly activated 4Å MS, and anh. CH_2Cl_2 (4.00 mL) were successively added into a vial. After stirring at room temperature for 12 h, (3,4,6-tri-*O*-benzyl-β-D-glucopyranosyl)tri-*n*-butylstannane **10** (72.4 mg, 0.100 mmol) and hydroxy(tosyloxy)iodobenzene (39.2 mg, 0.100 mmol) were added and stirred for additional 12 h. (3,4,6-Tri-*O*-benzyl-β-D-glucopyranosyl)tri-*n*-butylstannane **10** (72.4 mg, 0.100 mmol) and hydroxy(tosyloxy)iodobenzene (39.2 mg, 0.100 mmol) were added and stirred at room temperature for 12 h and afforded after chromatographic purification on SiO_2 (Hexanes:EtOAc, 15:1) **52** (49.7 mg, 60%) as a pale yellow liquid. Characterization data matched the literature report.[10]

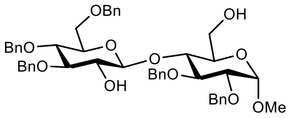

55

Methyl 3,4,6-tri-*O*-benzyl-*β*-D-glucopyranosyl-(1→4)-2,3-di-*O*-benzyl- *α*-D-glucopyranoside (55). According to the general protocol *C*, (3,4,6-tri-*O*-benzyl-*β*-D-glucopyranosyl)tri-*n*-butylstannane **10** (72.4 mg, 0.100 mmol), methyl 2,3-di-*O*-benzyl-6-*O*-pivaloyl-*α*-D-glucopyranoside **53** (45.9 mg, 0.100 mmol), $Zn(OTf)_2$ (1.83 mg, 0.005 mmol), 3,5-bis(trifluoromethyl)-1-hydroxy(tosyloxy)iodobenzene (52.8 mg, 0.100 mmol), freshly activated 4Å MS and anh. CH_2Cl_2 (4.00 mL) were successively added into a vial. After stirring at room temperature for 12 h, (3,4,6-tri-*O*-benzyl–*β*–D-glucopyranosyl)tri-*n*-butylstannane (72.4 mg, 0.100 mmol), 3,5-bis(trifluoromethyl)-1-hydroxy(tosyloxy)iodobenzene (52.8 mg, 0.100 mmol) were added and stirred for

additional 12 h. (3,4,6-tri-*O*-benzyl–*β*–D-glucopyranosyl)tri-*n*-butylstannane **10** (72.4 mg, 0.100 mmol) and 3,5-bis(trifluoromethyl)-1-hydroxy(tosyloxy)iodobenzene (52.8 mg, 0.100 mmol) were added and stirred at room temperature for 12 h. The resulting mixture was filtered through Celite® and diluted with MeOH (10 mL). To the suspension in MeOH was added MeONa (0.1 mL, 25% wt. in MeOH) the mixture was allowed to stir at 50 °C for 10 h, which afforded after chromatographic purification on SiO_2 (Hexanes:EtOAc, 1:1) **55** (44.4 mg, 55%) as a colorless gel: $[\alpha]_D^{24}$= +37.0 (c = 1.6, $CHCl_3$); IR (ATR) ν = 3469, 3067, 3033, 2906, 1499, 1458, 1365, 1108, 1055, 1033, 739,702 cm^{-1}; ^1H NMR (300 MHz, $CDCl_3$) δ 7.38 – 7.22 (m, 23H), 7.12 – 7.06 (m, 2H), 5.12 (d, *J* = 11.4 Hz, 1H), 4.86 (d, *J* = 11.3 Hz, 1H), 4.81 – 4.67 (m, 4H), 4.63 – 4.56 (m, 3H), 4.53 (d, *J* = 12.1 Hz, 1H), 4.45 – 4.32 (m, 2H), 4.08 (dd, *J* = 12.5, 6.4 Hz, 1H), 3.97 (t, *J* = 9.2 Hz, 1H), 3.89 (t, *J* = 9.3 Hz, 1H), 3.70 – 3.41 (m, 9H), 3.36 (s, 3H), 3.30 (dt, *J* = 9.7, 2.6 Hz, 1H), 3.21 (t, *J* = 7.1 Hz, 1H); ^{13}C NMR (75 MHz, $CDCl_3$) δ 138.7, 138.4, 138.2, 137.9, 137.7, 128.7, 128.6, 128.5(2), 128.4(2), 128.2, 128.1(2), 127.9(3), 127.7, 127.6, 102.7, 98.1, 84.2, 80.5, 80.4, 75.5, 75.3, 75.1, 73.6, 73.4, 73.3, 72.8, 71.2, 67.8, 60.9, 55.3; HRMS (ESI) *m/z* calcd for $C_{48}H_{54}O_{11}$ $[M + Na]^+$ 829.3564, found 829.3568.

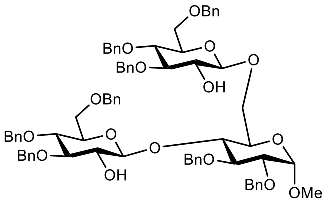

56

Methyl 3,4,6-tri-*O*-benzyl-*β*-D-glucopyranosyl-(1→6)-[3,4,6-tri-*O*-benzyl-*β*-D-glucopyranosyl-(1→4)-2,3-di-O-(4-methoxybenzyl)-*α*-D-glucopyranoside (56). According to the general protocol *C*, (3,4,6-tri-*O*-benzyl-*β*-D-glucopyranosyl)tri-*n*-butylstannane **10** (72.4 mg, 0.100 mmol), methyl 3,4,6-tri-*O*-benzyl-*β*-D-glucopyranosyl-(1→4)-2,3-di-*O*-benzyl-*α*-D-glucopyranoside **55** (80.7 mg, 0.100 mmol), $Zn(OTf)_2$ (2.70 mg, 0.010 mmol), hydroxy(tosyloxy)iodobenzene (39.2 mg, 0.100 mmol), freshly activated 4Å MS and anh. CH_2Cl_2 (4.00 mL) were successively added into a vial. After stirring at room temperature for 12 h, (3,4,6-tri-*O*-benzyl-*β*-D-glucopyranosyl)tri-*n*-butylstannane[1] **10** (72.4 mg, 0.100 mmol) and hydroxy(tosyloxy)iodobenzene (39.2 mg, 0.100 mmol) were added and stirred for additional 12 h. (3,4,6-Tri-*O*-benzyl-*β*-D-glucopyranosyl)tri-*n*-butylstannane **10** (72.4 mg, 0.100 mmol) and hydroxy(tosyloxy)iodobenzene (39.2 mg, 0.100 mmol) were added and stirred at room temperature for 12 h and afforded after chromatographic purification on SiO_2 (Hexanes:EtOAc, 1:1) **56** (69.4 mg, 56%) as a colorless oil: $[\alpha]_D^{24}$ = +37.8 (c = 0.110, $CHCl_3$); IR (ATR) ν = 3461, 3089, 3067, 3033, 2910, 2869, 1499, 1458, 1365, 1055, 739, 702cm^{-1}; ^1H NMR (300 MHz, $CDCl_3$) δ 7.38 - 7.08 (m, 40H), 5.11 (d, *J* = 3.4 Hz, 1H), 5.04 - 4.70 (m, 8H), 4.69 - 4.38 (m, 9H), 4.16 - 3.38 (m, 18H), 3.34 (s, 3H), 3.31 - 3.21 (m, 1H), 2.74 (m, 1H), 2.58 (m, 1H); ^{13}C NMR (75 MHz, $CDCl_3$) δ 139.4, 138.8, 138.7, 138.4 (2), 138.3, 138.1 (2), 128.7, 128.6 (2), 128.5 (2),

128.4, 128.3, 128.2, 128.1 (2), 128.0 (2), 127.9, 127.8 (3), 127.7 (2), 127.6, 127.2, 126.9, 103.5, 98.9, 98.1, 84.6, 83.5, 80.8, 80.3, 77.4 (2), 75.9, 75.7, 75.4 (2), 75.1, 75.0 (2), 73.7, 73.6, 73.4 (2), 71.1, 69.8, 68.7, 65.8, 55.4.; HRMS (ESI) *m/z* calcd for $C_{75}H_{82}O_{16}Na$ $[M + Na]^+$ 1261.5495, found 1261.6104.

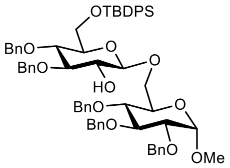

59

Methyl 3,4-di-*O*-benzyl-6-*O*-*tert*-butyldimethylsilyl-*β*-D-glucopyranosyl-(1→6)-2,3,4-tri-O-benzyl–*α*–D-glucopyranoside (**59**). According to the general protocol *C*, (3,4-di-*O*-benzyl-6-*O*-tert-butyldiphenylsilyl–*β*–D-glucopyranosyl)tri-*n*-butylstannane[10] **57** (87.2 mg, 0.100 mmol), methyl 2,3,4-tri-*O*-benzyl-α-D-glucopyranoside[7] **58** (46.5 mg, 0.100 mmol), $Zn(OTf)_2$ (1.83 mg, 0.005 mmol), hydroxy(tosyloxy)iodobenzene (39.2 mg, 0.100 mmol), freshly activated 4Å MS and anh. CH_2Cl_2 (4.00 mL) were successively added into a vial. After stirring at room temperature for 12 h, 3,4-di-*O*-benzyl-6-*O*-tert-butyldiphenylsilyl–*β*–D-glucopyranosyl)tri-*n*-butylstannane[10] **57** (87.2 mg, 0.100 mmol) and hydroxy(tosyloxy)iodobenzene (39.2 mg, 0.100 mmol) were added and stirred for additional 12 h. 3,4-Di-*O*-benzyl-6-*O*-tert-butyldiphenylsilyl-*β*-D-glucopyranosyl)tri-*n*-butylstannane[10] **57** (87.2 mg, 0.100 mmol) and hydroxy(tosyloxy)iodobenzene (39.2 mg, 0.100 mmol) were added and stirred at room temperature for 12 h and afforded after chromatographic purification on SiO_2 (Hexanes:EtOAc, 3:1) **59** (85.7 mg, 82%) as a colorless oil: $[\alpha]_D^{23}$ = + 5.6 (c = 0.14, $CHCl_3$) ; IR (ATR) ν = 3464, 3031, 2929, 1454, 2929, 1454, 1359, 1156, 1068, 823, 737, 698, 612, 504 cm^{-1}; ^1H NMR (300 MHz, $CDCl_3$) δ 7.79 -7.68 (m, 4H), 7.53 - 7.34 (m, 25H), 7.29 - 7.19 (m, 6H), 5.05 (d, *J* = 10.8 Hz, 1H), 5.03 - 4.82 (m, 7H), 4.77 - 4.65 (m, 3H), 4.61 (d, *J* = 11.1 Hz, 1H), 4.06 (t, *J* = 9.2 Hz, 1H), 3.97 - 3.64 (m, 9H), 3.60 - 3.47 (m, 2H), 3.43 (s, 3H), 2.21 (d, *J* = 7.3 Hz, 1H), 1.10 (s, 9H); ^{13}C NMR (75 MHz, $CDCl_3$) δ 138.8 (2), 138.6, 138.2, 138.1, 136.0, 135.8, 133.8, 133.4, 129.7, 129.7, 128.6 (2), 128.5 (2), 128.3, 128.2 (2), 128.1, 127.9, 127.8 (3), 127.7 (2), 99.1, 98.0, 83.5, 82.2, 80.3, 77.8, 77.3, 76.0, 75.5, 75.1, 73.6, 73.5, 72.2, 69.8, 66.8, 62.7, 55.4, 26.9, 19.5; HRMS (ESI) *m/z* calcd for $C_{64}H_{72}O_{11}Si$ $[M + Na]^+$ 1067.4736, found 1067.4749.

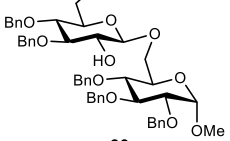

60

Methyl 3,4-di-*O*-benzyl-*β*-D-glucopyranosyl-(1→6)-2,3,4-tri-O-benzyl-*α*-D-glucopyranoside (**60**). Under N_2, methyl 3,4-di-*O*-benzyl-6-*O*-*tert*-butyldimethylsilyl-*β*-D-glucopyranosyl-(1→6)- 2,3,4-tri-*O*-benzyl–*α*–D-glucopyranoside **59** (105 mg,

0.100 mmol) and anh. THF (2.00 mL) were successively added into a flame dried flask. The mixture was cooled to 0°C for 0.5 h followed by the addition of TBAF (0.30 mL, 0.300 mmol, 1.0 M in THF). The reaction mixture was warmed up to rt and stirred for 24 h. Purification by column chromatography on SiO_2 (Hexanes:EtOAc, 2:1) afforded **60** (71.0 mg, 88%) as a colorless oil: $[\alpha]_D^{23}$ = + 6.4 (c = 0.13, $CHCl_3$); IR (ATR) = 3925, 3314, 2922, 1621, 1452, 1364, 1206, 1149, 1047, 848, 749, 697, 622, 499 cm^{-1}; ^1H NMR (300 MHz, $CDCl_3$) δ 7.43 - 7.22 (m, 25H), 5.00 (d, *J* = 10.7 Hz, 1H), 4.94 (d, *J* = 11.2 Hz, 1H), 4.93 (d, *J* = 11.2 Hz, 1H), 4.91 - 4.76 (m, 5H), 4.72 - 4.54 (m, 4H), 4.01 (t, *J* = 9.2 Hz, 1H), 3.88 (dd, *J* = 11.2, 4.6 Hz, 1H), 3.82 - 3.59 (m, 7H), 3.56 - 3.43 (m, 3H), 3.37 (s, 3H), 2.07 (m, 1H), 1.64 (m, 1H); ^{13}C NMR (75 MHz, $CDCl_3$) δ 138.7 (2), 138.3, 138.2 (2), 128.6 (2), 128.5, 128.2, 128.1 (2), 128.0 (2), 127.8, 99.1, 98.1, 83.1, 82.2, 80.2, 77.8, 77.1, 76.0, 75.3, 75.1 (2), 73.5, 73.4, 71.5, 69.7, 67.1, 61.8, 55.5; HRMS (ESI) *m/z* calcd for $C_{48}H_{54}O_{11}$ $[M + Na]^+$ 829.3558, found 829.3551.

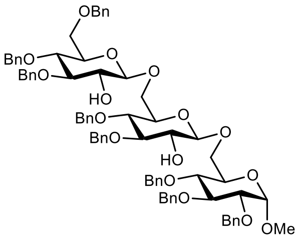

61

Methyl 3,4,6-tri-*O*-benzyl-*β*-D-glucopyranosyl-(1→6)-3,4-di-O-benzyl-*β*–D-glucopyranosyl-(1→6)-2,3,4-tri-O-benzyl-*α*-D-glucopyranoside (**61**). According to the general protocol *C*, methyl 3,4-di-*O*-benzyl-*β*-D-glucopyranosyl-(1→6)-2,3,4-tri-O-benzyl-*α*-D- glucopyranoside **60** (80.7 mg, 0.100 mmol), (3,4,6-tri-*O*-benzyl–*β*–D-glucopyranosyl)tri-*n*-butylstannane[7] **10** (72.4 mg, 0.100 mmol), $Zn(OTf)_2$ (1.83 mg, 0.005 mmol), hydroxy(tosyloxy)iodobenzene (39.2 mg, 0.100 mmol), freshly activated 4Å MS, and anh. CH_2Cl_2 (4.00 mL) were successively added into a vial. After stirring at room temperature for 12 h, (3,4,6-tri-*O*-benzyl–*β*–D-glucopyranosyl)tri-*n*-butylstannane **10** (72.4 mg, 0.100 mmol) and hydroxy(tosyloxy)iodobenzene (39.2 mg, 0.100 mmol) were added and stirred for additional 12 h. (3,4,6-tri-*O*-benzyl–*β*–D-glucopyranosyl)tri-n-butylstannane[1] **10** (72.4 mg, 0.100 mmol) and hydroxy(tosyloxy)iodobenzene (39.2 mg, 0.100 mmol) were added and stirred at room temperature for 12 h and afforded after chromatographic purification on SiO_2 (Hexanes:EtOAc, 1:1) **61** (88.0 mg, 71%) as a colorless oil: $[\alpha]_D^{23}$ = + 15.6 (c = 0.07, $CHCl_3$); IR (ATR) ν = 3458, 3031, 2910, 1496, 1453, 1359, 1275, 1064, 909, 747, 697 cm^{-1}; ^1H NMR (300 MHz, $CDCl_3$) δ 7.43 - 7.09 (m, 40H), 4.99 (d, *J* = 10.8 Hz, 1H), 4.96 - 4.73 (m, 9H), 4.71 - 4.46 (m, 8H), 4.24 (d, *J* = 7.0 Hz, 1H), 4.10 (d, *J* = 10.6 Hz, 1H), 4.00 (t, *J* = 9.2 Hz, 1H), 3.88 (dt, *J* = 11.3, 5.8 Hz, 2H), 3.83 - 3.42 (m, 14H), 3.36 (s, 3H), 2.63 (m, 1H), 2.12 (m, 1H); ^{13}C NMR (75 MHz, $CDCl_3$) δ 138.8 (2), 138.7, 138.4, 138.3 (2), 138.1, 128.7, 128.6 (2) 128.5 (3), 128.2 (3), 128.1 (2), 128.0, 127.9 (3), 127.8 (2), 127.7, 103.7, 99.0, 98.0, 92.7, 84.7, 83.3, 82.5, 82.2, 80.3, 77.7, 77.4, 76.0, 75.4, 75.2, 75.1 (2), 74.7, 73.6 (2), 73.3, 70.9 (2), 69.9, 69.1, 68.6, 67.3, 55.5;

HRMS (ESI) *m/z* calcd for $C_{75}H_{82}O_{16}$ $[M + Na]^+$ 1261.5495, found 1261.5498.

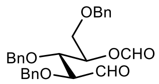

63

2,3,5-tri-*O*-benzyl-4-*O*-formyl-D-arabinose (**63**). To a solution of 3,4,6-tri-*O*-benzyl–*β*–D-glucose (45.0 mg, 0.100 mmol) in anh. $CHCl_3$ (2.00 mL) was added iodosobenzene (23.0 mg, 0.104 mmol). The mixture was allowed to stir vigorously at room temperature for 4 h. The resulting suspension was filtered through Celite® and concentrated under vacuum to afford **63** (43.3 mg, 97%) as a colorless oil: $[\alpha]_D^{23}$ = -20.9 (c = 1.00, $CHCl_3$); IR (ATR) = 3067, 3033, 2929, 2873, 1730, 1499, 1458, 1175, 1104, 1033, 743, 702 cm^{-1}; ^1H NMR (500 MHz, $CDCl_3$) δ 9.65 (d, *J* = 1.2 Hz, 1H), 7.85 (s, 1H), 7.37 - 7.27 (m, 13H), 7.22 - 7.16 (m, 2H), 5.30 (ddd, *J* = 7.4, 4.4, 3.0 Hz, 1H), 4.72 (d, *J* = 11.8 Hz, 1H), 4.56 - 4.43 (m, 5H), 4.23 (dd, *J* = 7.4, 3.1 Hz, 1H), 3.92 (dd, *J* = 3.1, 1.3 Hz, 1H), 3.81 (dd, *J* = 11.1, 2.9 Hz, 1H), 3.76 (dd, *J* = 11.1, 4.6 Hz, 1H); ^{13}C NMR (126 MHz, $CDCl_3$) δ 203.1, 160.0, 137.6, 137.1, 136.5, 128.9, 128.8, 128.6 (2), 128.5, 128.4, 128.3, 128.0 (2), 82.5, 76.7, 74.4, 73.6, 73.5, 71.5, 67.7; HRMS (ESI) *m/z* calcd for $C_{27}H_{28}O_6$ $[M + Na]^+$ 471.1783, found 471.1781.

4. Detailed Experimental Procedures for Compounds S1 - S6

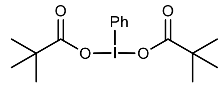

S1

Phenyliodonium dipivalate (S1). A mixture of PIDA (0.010 mmol, 1.00 equiv.) and pivalic acid (0.020 mmol, 2.00 equiv.) was stirred in m-xylene (5 mL) at 80 °C for 5 min. The solvent was then distilled off under vacuum at this temperature to afford S1 as white solid, which was used directly in the next step without further purification: mp 99-102 °C; IR (ATR) ν = 3078, 2981, 2873, 1704, 1644, 1555, 1480, 1398, 1286, 1168, 996, 892, 750, 605 cm^{-1}; ^{1}H NMR (500 MHz, $CDCl_3$) δ 8.03 - 7.98 (m, 2H), 7.58 - 7.52 (m, 1H), 7.51 - 7.44 (m, 2H), 1.17 - 1.05 (m, 18H). ^{13}C NMR (126 MHz, $CDCl_3$) δ 183.7, 134.4, 131.4, 130.7, 122.2, 39.1, 27.9.

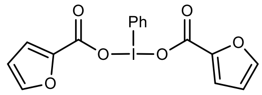

S2

Phenyliodonium difuroate (**S2**). A mixture of PIDA (0.010 mmol, 1.00 equiv.) and furan-2-carboxylic acid (0.020 mmol, 2.00 equiv.) was stirred in m-xylene (5 mL) at 80 °C for 5 min. The solvent was then distilled off under vacuum at this temperature to afford **S2** as light solid, which was used directly in the next step without further purification**:** mp 104-107 °C; IR (ATR) ν = 3136, 3051, 1630, 1564, 1469, 1389, 1308, 1228, 1113, 1011, 931, 882, 805, 727 cm^{-1}; ^{1}H NMR (300 MHz, $CDCl_3$) δ 8.30 - 8.19 (m, 2H), 7.66 - 7.57 (m, 1H), 7.56 - 7.51 (m, 2H), 7.50 (dd, J = 1.7, 0.9 Hz, 2H), 7.06 (dd, J = 3.5, 0.9 Hz, 2H), 6.44 (dd, J = 3.5, 1.8 Hz, 2H); ^{13}C NMR (126 MHz, $CDCl_3$) δ 146.1, 137.6, 135.2, 132.2, 131.3, 130.4, 118.2, 112.4, 111.9.

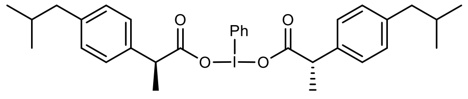

S3

Phenyliodonium bis(ibuprofen) (**S3**). A mixture of PIDA (0.010 mmol, 1.00 equiv.) and (*S*)-2-(4-isobutylphenyl)propanoic acid (0.020 mmol, 2.00 equiv.) was stirred in m-xylene (5 mL) at 80 °C for 5 min. The solvent was then distilled off under vacuum at this temperature to afford **S3** as white oil, which was used directly in the next step without further purification**:** IR (ATR) ν = 3055, 2969, 2873, 1707, 1689, 1610, 1417, 1271, 1231, 854, 735 cm^{-1}; ^{1}H NMR (300 MHz, $CDCl_3$) δ 7.79 (dd, J = 8.5, 1.2 Hz, 2H), 7.53 – 7.45 (m, 1H), 7.40 – 7.28 (m, 2H), 7.11 (d, J = 8.0 Hz, 4H), 7.03 (d, J = 8.3 Hz, 4H), 3.65 (q, J = 7.1 Hz, 2H), 2.44 (d, J = 7.2 Hz, 3H), 1.84 (sept, J = 6.8 Hz, 2H), 1.41 (d, J = 7.1 Hz, 6H), 0.90 (d, J = 6.6 Hz, 12 h). ^{13}C NMR (101 MHz, $CDCl_3$) δ 179.2, 140.3, 138.4, 134.3, 131.4, 130.7, 129.2, 127.2, 122.1, 45.2, 45.1, 30.3, 22.5, 19.1.

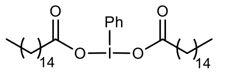
S4

Phenyliodonium dipalmate (S4). A mixture of PIDA (0.010 mmol, 1.00 equiv.) and pivalic acid (0.020 mmol, 2.00 equiv.) was stirred in m-xylene (5 mL) at 80 °C for 5 min. The solvent was then distilled off under vacuum at this temperature to afford S1 as white solid, which was used directly in the next step without further purification**:** mp 73-75 °C; IR (ATR) ν = 3059, 2918, 2851, 1704, 1659, 1473, 1376, 1312, 1204, 1186, 743, 720 cm^{-1}; ^{1}H NMR (500 MHz, $CDCl_3$) δ 8.03 - 7.98 (m, 2H), 7.58 - 7.52 (m, 1H), 7.51 - 7.44 (m, 2H), 1.17 - 1.05 (m, 18H). ^{13}C NMR (126 MHz, $CDCl_3$) δ 183.7, 134.4, 131.4, 130.7, 122.2, 39.1, 27.9. ^{1}H NMR (500 MHz, $CDCl_3$) δ 8.07 (d, *J* = 7.8 Hz, 2H), 7.58 (t, *J* = 7.4 Hz, 1H), 7.48 (t, *J* = 7.7 Hz, 2H), 2.24 (t, *J* = 7.5 Hz, 4H), 1.53 (m, 4H), 1.24 (d, *J* = 13.8 Hz, 48H), 0.87 (t, *J* = 6.8 Hz, 6H). ^{13}C NMR (126 MHz, $CDCl_3$) δ 179.1, 135.0, 131.7, 131.0, 121.9, 34.2, 32.1, 29.8, 29.8, 29.8, 29.7, 29.6, 29.5, 29.4, 29.4, 25.8, 22.8, 14.3.

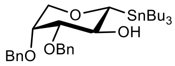
S5

(3,4-Di-*O*-benzyl-*β*-D-arabinosyl)tri-*n*-butylstannane (S5). To a solution of 3,4-di-*O*-benzyl-D-arabinal[13] (500.0 mg, 1.69 mmol) in the ice bath, vigorously stirring biphasic solution of CH_2Cl_2 (10.0 mL), satd. aq. $NaHCO_3$ (18.0 mL), and acetone (1.00 mL), a solution of Oxone® (4.16 g, 6.76 mmol) in H_2O (18.0 mL) was added dropwise over 15 min. After the addition was complete, the mixture was stirred for 0.5 h at 0 °C then for 2 h at rt. The organic phase was separated, and the aqueous phase was extracted with CH_2Cl_2 (2x 10.0 mL). The combined organic phases were dried over Na_2SO_3 and concentrated to afford the epoxide as a white solid. The crude epoxide was dissolved in anh. and degassed THF (15.0 mL) under N_2 and cooled to -20 °C for the addition of $MeMgSnBu_3$ (837.0 mg, 2.53 mmol). The solutions were stirred at -20 °C for 2 h and then quenched with H_2O (18.0 mL). The mixture is filtered twice through Celite® and the organic phase is separated. The aqueous phase was extracted with CH_2Cl_2 (3 x 50.0 mL), and the combined organic layers were dried ($MgSO_4$), concentrated, and purified by flash column chromatography on SiO_2 (Hexanes:EtOAc, 15:1) to afford **S5** (785.7 mg, 45%) as a pale yellow oil: $[\alpha]_D^{23}$ = -23.3 (c = 0.60, $CHCl_3$); IR (ATR) ν = 3451, 3029, 2920, 1453, 1343, 1226, 1067, 862, 733, 696, 592, 505 cm^{-1}; ^{1}H NMR (300 MHz, $CDCl_3$) δ 7.42 - 7.27 (m, 10H), 4.74 (d, *J* = 12.4 Hz, 1H), 4.62 (d, *J* = 11.7 Hz, 1H), 4.57 (d, *J* = 12.4 Hz, 1H), 4.43 (d, *J* = 11.6 Hz, 1H), 4.28 - 4.10 (m, 2H), 3.80 (ddt, *J* = 4.0, 2.7, 1.3 Hz, 1H), 3.45 - 3.34 (m, 1H), 3.30 (dd, *J* = 8.9, 3.2 Hz, 1H), 3.18 (dd, *J* = 12.6, 1.2 Hz, 1H), 2.29 (dd, *J* = 2.7, 0.9 Hz, 1H), 1.69 - 1.41 (m, 6H), 1.40 -1.24 (m, 6H), 1.11 - 0.81 (m, 15H); ^{13}C NMR (75 MHz, $CDCl_3$) δ 138.6, 138.2, 128.6, 128.5, 128.0, 128.0, 127.7, 84.4, 76.8, 72.6, 70.9, 70.4, 70.2, 29.2, 27.6, 13.9, 9.1; HRMS (ESI) *m/z* calcd for $C_{31}H_{48}O_4Sn$ $[M + Na]^+$ 627.2467, found 627.2468.

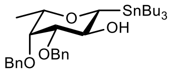

S6

(3,4-Di-*O*-benzyl-*β*-L-fucopyranosyl)tri-*n*-butylstannane (S6). To a solution of 3,4-di-*O*-benzyl-D-fucal[14] (2.24 g, 7.21 mmol) in a cooled (0 °C), vigorously stirring biphasic solution of CH_2Cl_2 (60.0 mL), saturated aq. $NaHCO_3$ (100.0 mL), and acetone (6.00 mL), a solution of Oxone® (17.8 g, 28.8 mmol) in H_2O (70.0 mL) was added dropwise over 15 min. The mixture was stirred at 0 °C for 0.5 h at rt for 2 h, the organic phase was then separated and the aqueous layer was extracted with CH_2Cl_2 (2 x 50.0 mL). The combined organic layers were dried (Na_2SO_3) and concentrated to afford crude epoxide as a white solid. This material was dissolved in anh. and degassed THF (70.0 mL), cooled to -20 °C, followed by the addition of a solution of $MeMgSnBu_3$ (3.57 g, 10.8 mmol) in THF. The solution was stirred at -20 °C for 2 h, quenched with H_2O (100 mL), filtered twice through Celite® and the organic phase was separated. The aqueous phase was extracted with CH_2Cl_2 (3 x 50.0 mL), and the combined organic layers were dried ($MgSO_4$), concentrated, and purified by flash column chromatography on SiO_2 (Hexanes:EtOAc, 10:1) to afford **S6** (1.90 g, 43%,) as a pale yellow oil: $[\alpha]_D^{24}$ = - 4.8 (c = 1.00, $CHCl_3$); IR (ATR) = 3029, 2921, 1494, 1454, 1352, 1110, 1063, 1022, 855, 730, 695, 595 cm^{-1}; ^1H NMR (300 MHz, $CDCl_3$) δ 7.41 - 7.27 (m, 10H), 4.92 (d, *J* = 11.7 Hz, 1H), 4.77 (d, *J* = 11.5 Hz, 1H), 4.67 (d, *J* = 11.8 Hz, 1H), 4.55 (d, *J* = 11.5 Hz, 1H), 4.19 (ddd, *J* = 10.9, 9.0, 2.7 Hz, 1H), 3.72 (dd, *J* = 2.9, 1.1 Hz, 1H), 3.40 (d, *J* = 10.9 Hz, 1H), 3.36 - 3.29 (m, 2H), 2.21 (d, *J* = 2.7 Hz, 1H), 1.65 - 1.44 (m, 6H), 1.40 - 1.24 (m, 6H), 1.17 (d, *J* = 6.4 Hz, 3H), 1.08 - 0.82 (m, 15H); ^{13}C NMR (75 MHz, $CDCl_3$) δ 139.1, 138.3, 128.7, 128.3, 128.0, 127.9 (2), 127.5, 87.1, 78.6, 76.5, 76.1, 74.7, 71.8, 70.2, 29.2, 27.5, 17.7, 13.9, 9.1; HRMS (ESI) *m/z* calcd for $C_{32}H_{50}O_4Sn$ $[M + Na]^+$ 641.2623, found 641.2629.

Supplementary Figures

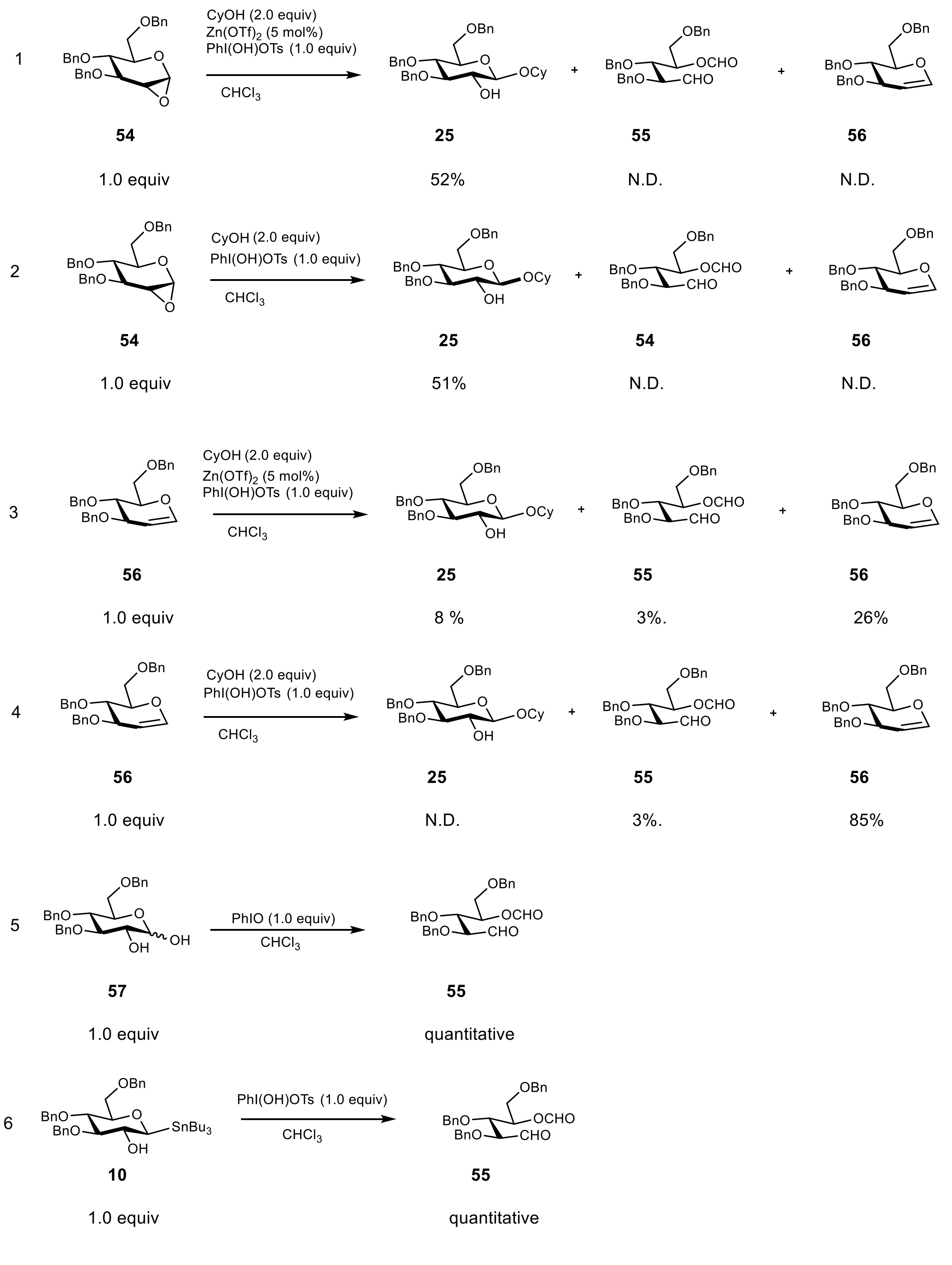

Supplementary Figure 1. Additional reactions for mechanistic studies.

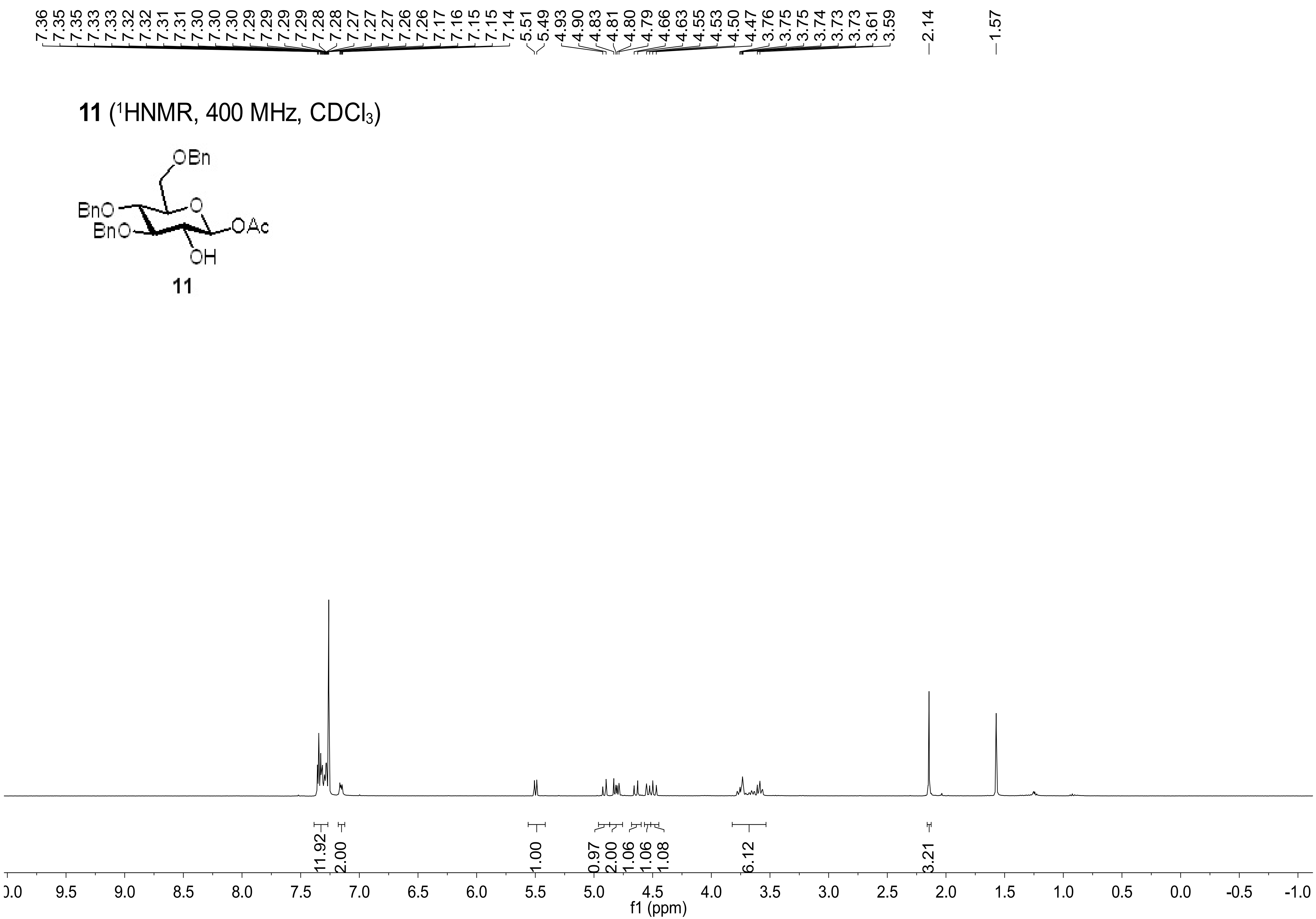

Supplementary Figure 2. ^{1}H NMR Spectrum for Compound 11

11 (^{13}CNMR, 101 MHz, $CDCl_3$)

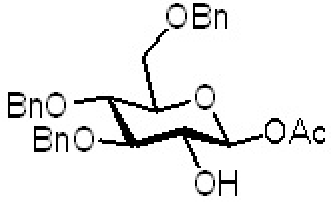

11

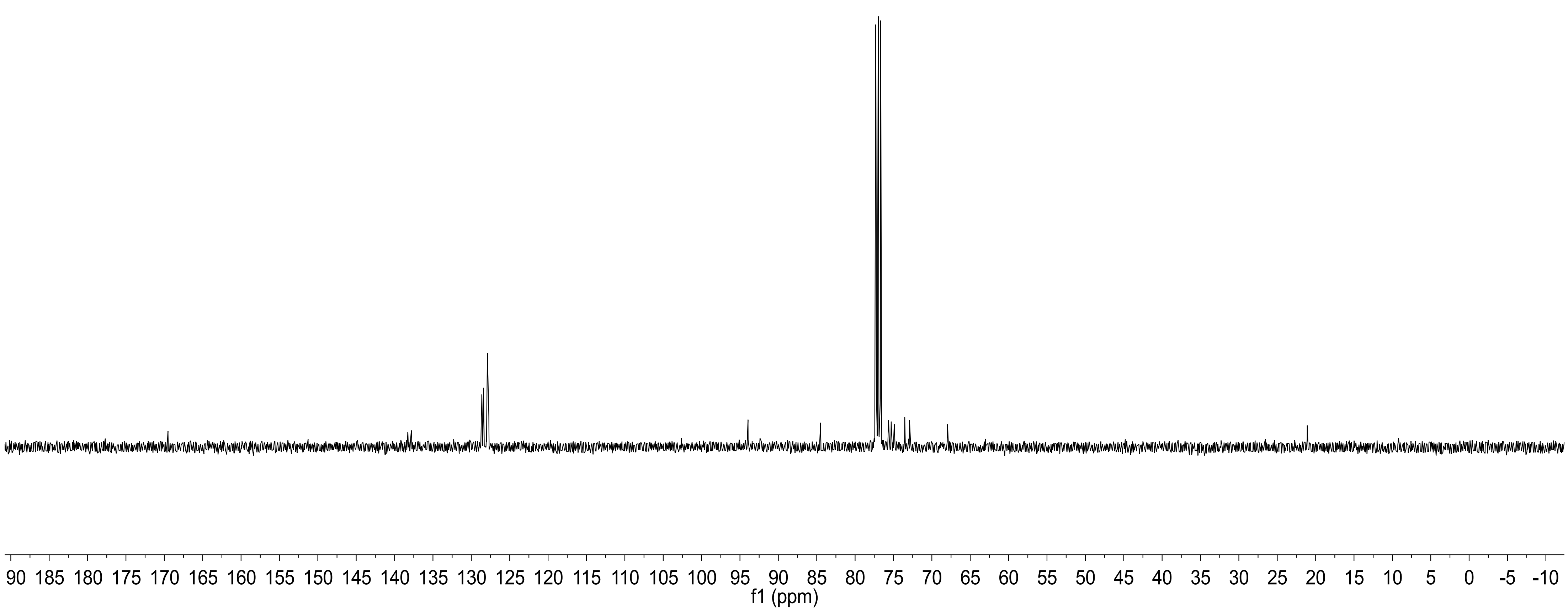

Supplementary Figure 3. ^{13}**C NMR Spectrum for Compound 11**

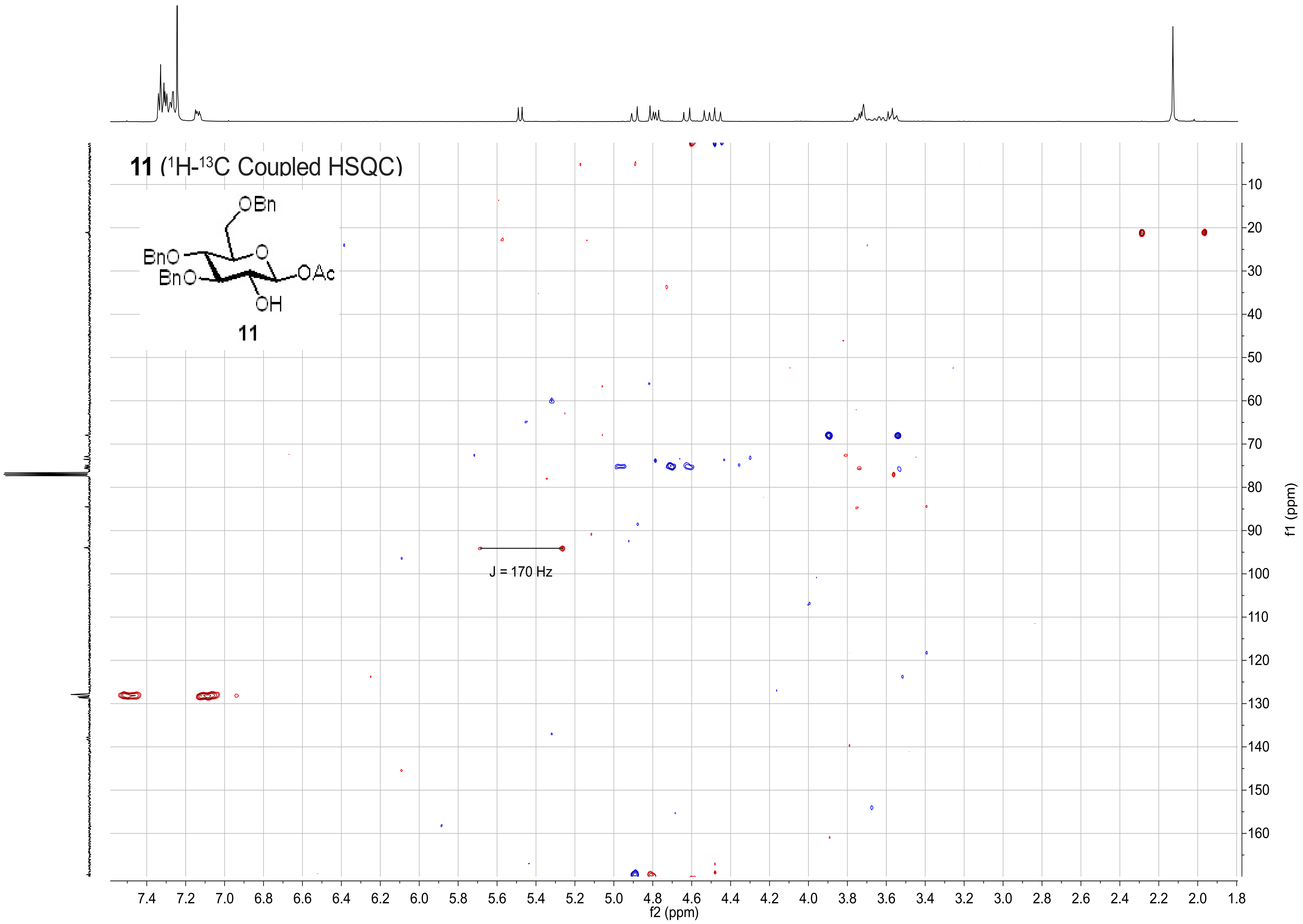

Supplementary Figure 4. ¹H-¹³C HSQC Coupled Spectrum for Compound 11

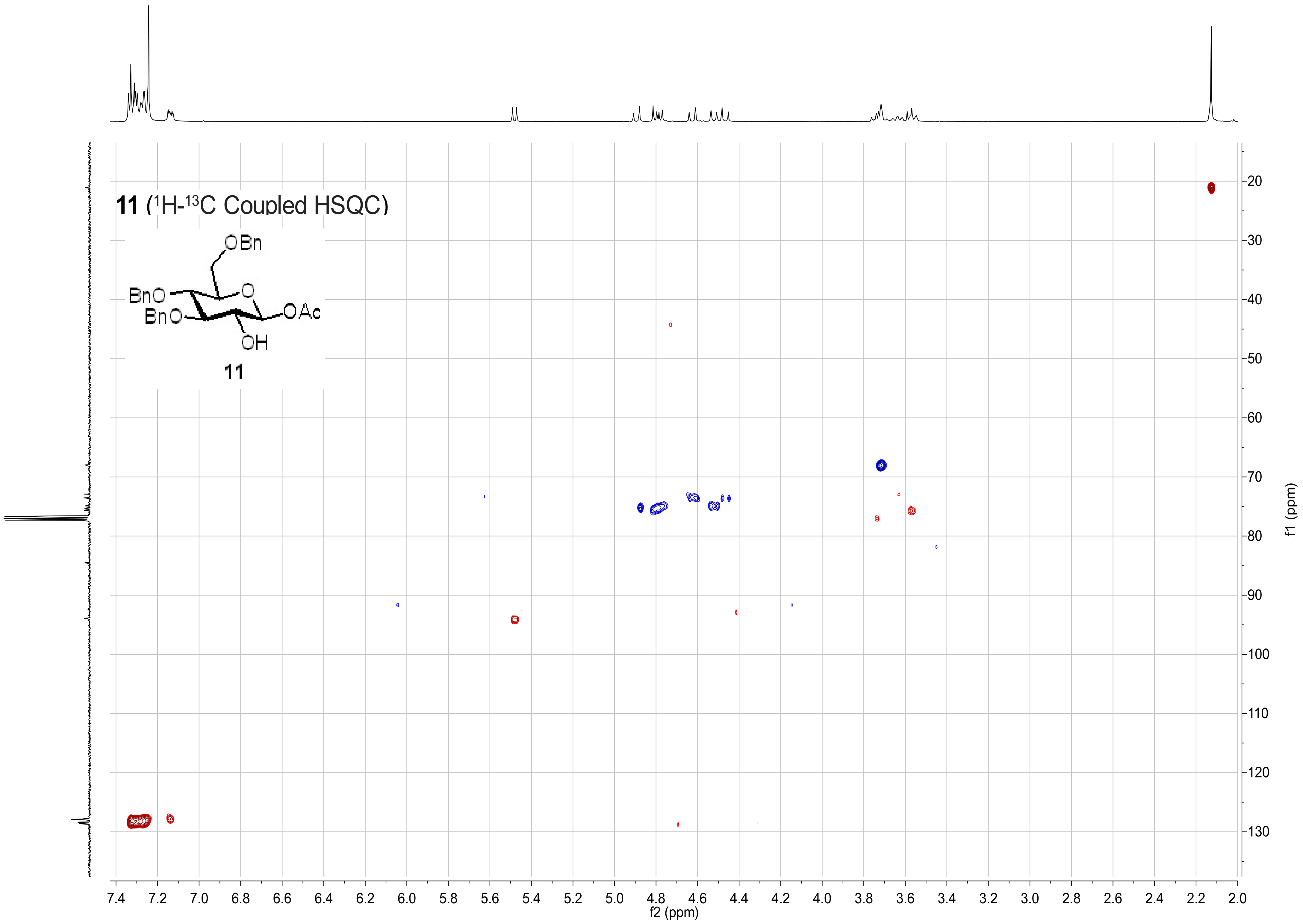

Supplementary Figure 5. ^{1}H-^{13}C **HSQC Decoupled Spectrum for Compound 11**

16 (^{1}HNMR, 500 MHz, $CDCl_3$)

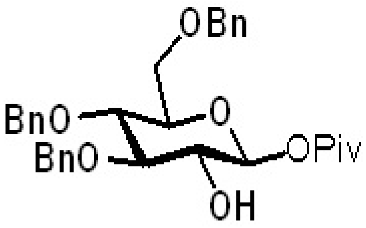

16

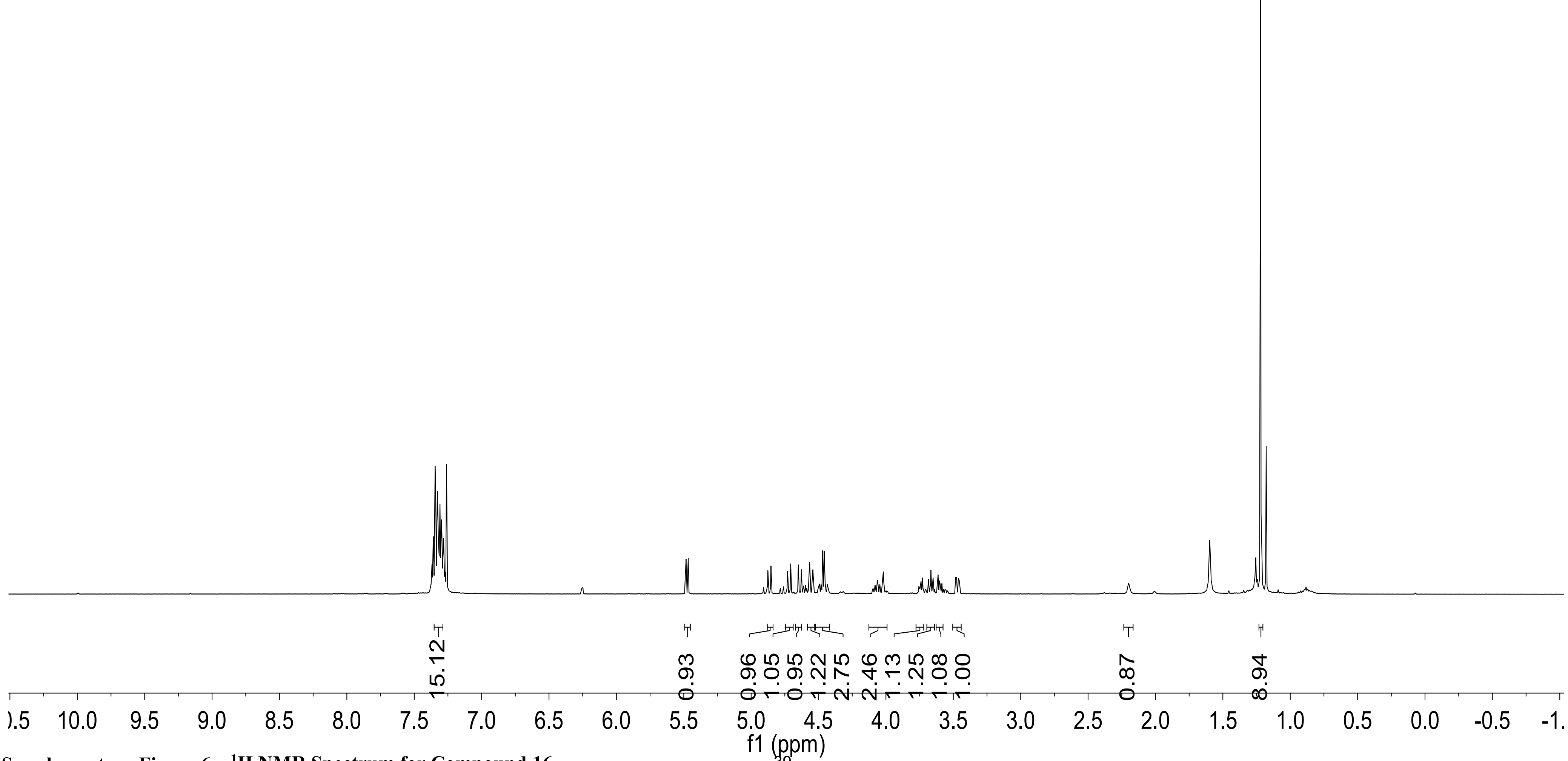

Supplementary Figure 6. ^{1}H NMR Spectrum for Compound 16

16 (^{13}C NMR, 101 MHz, $CDCl_3$)

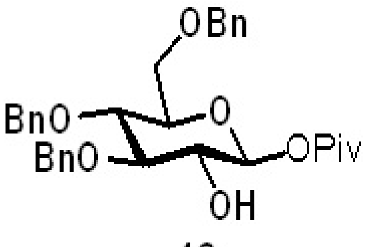

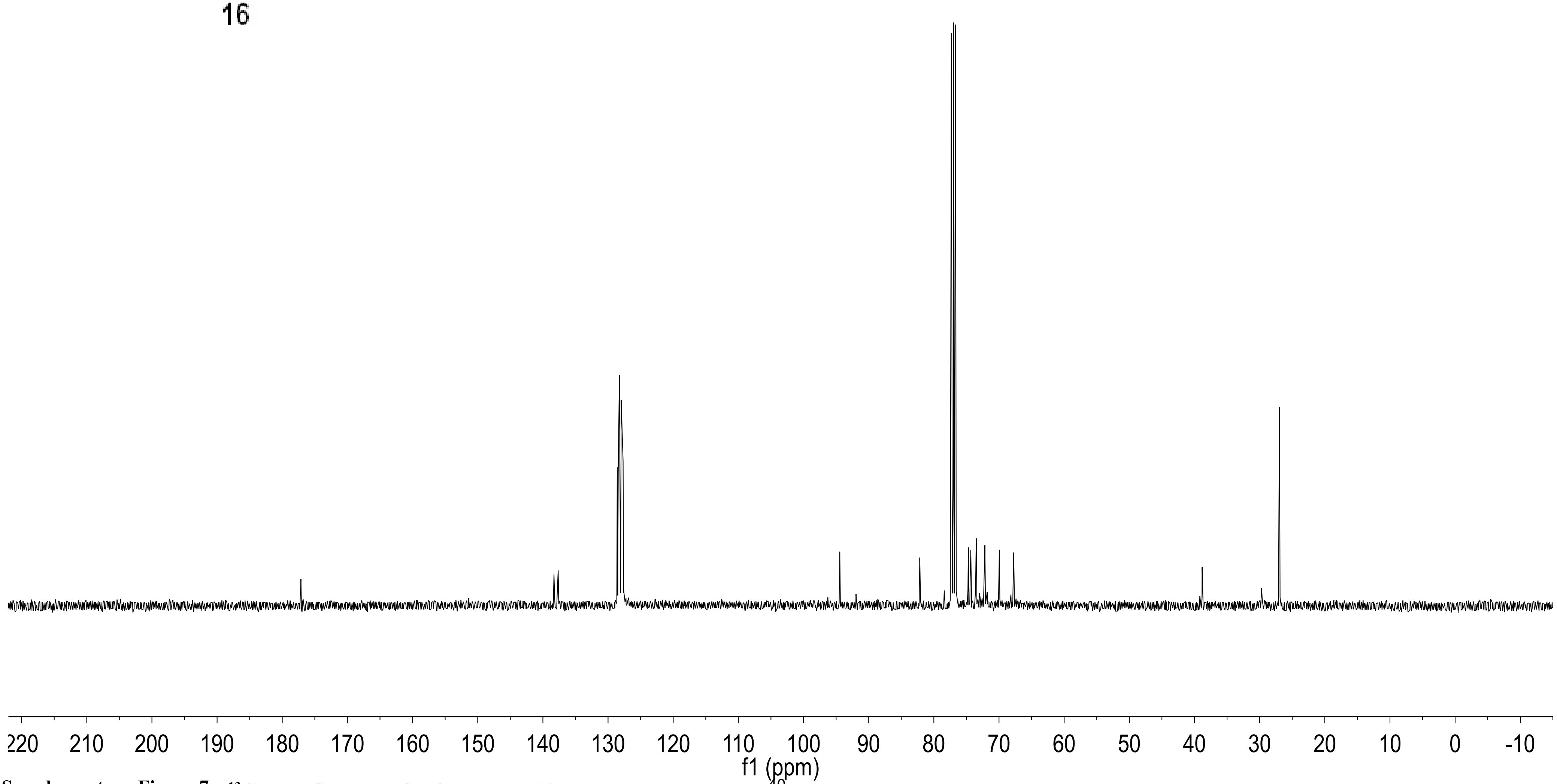

Supplementary Figure 7. ^{13}C NMR Spectrum for Compound 16

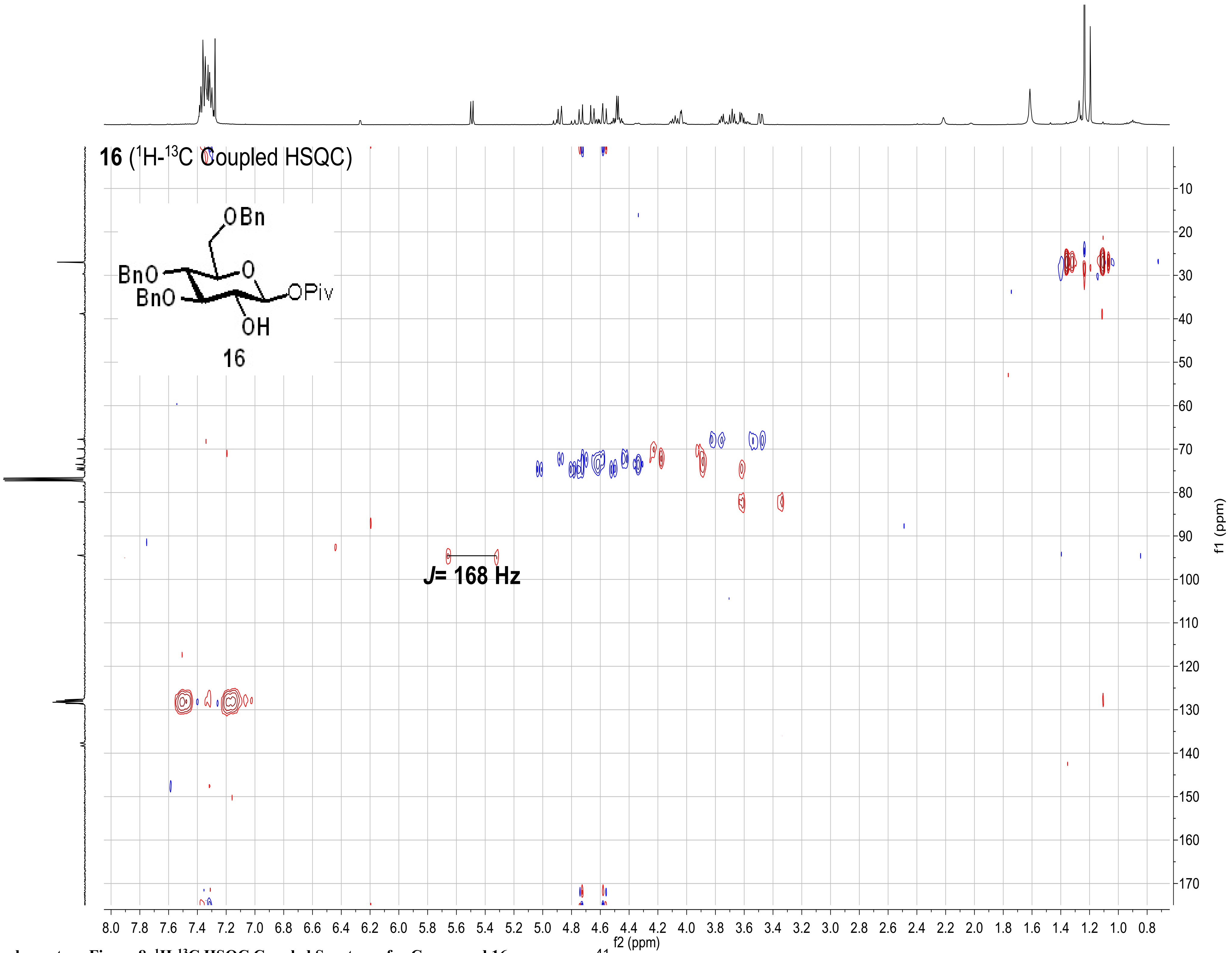

Supplementary Figure 8. ^{1}H-^{13}C HSQC Coupled Spectrum for Compound 16

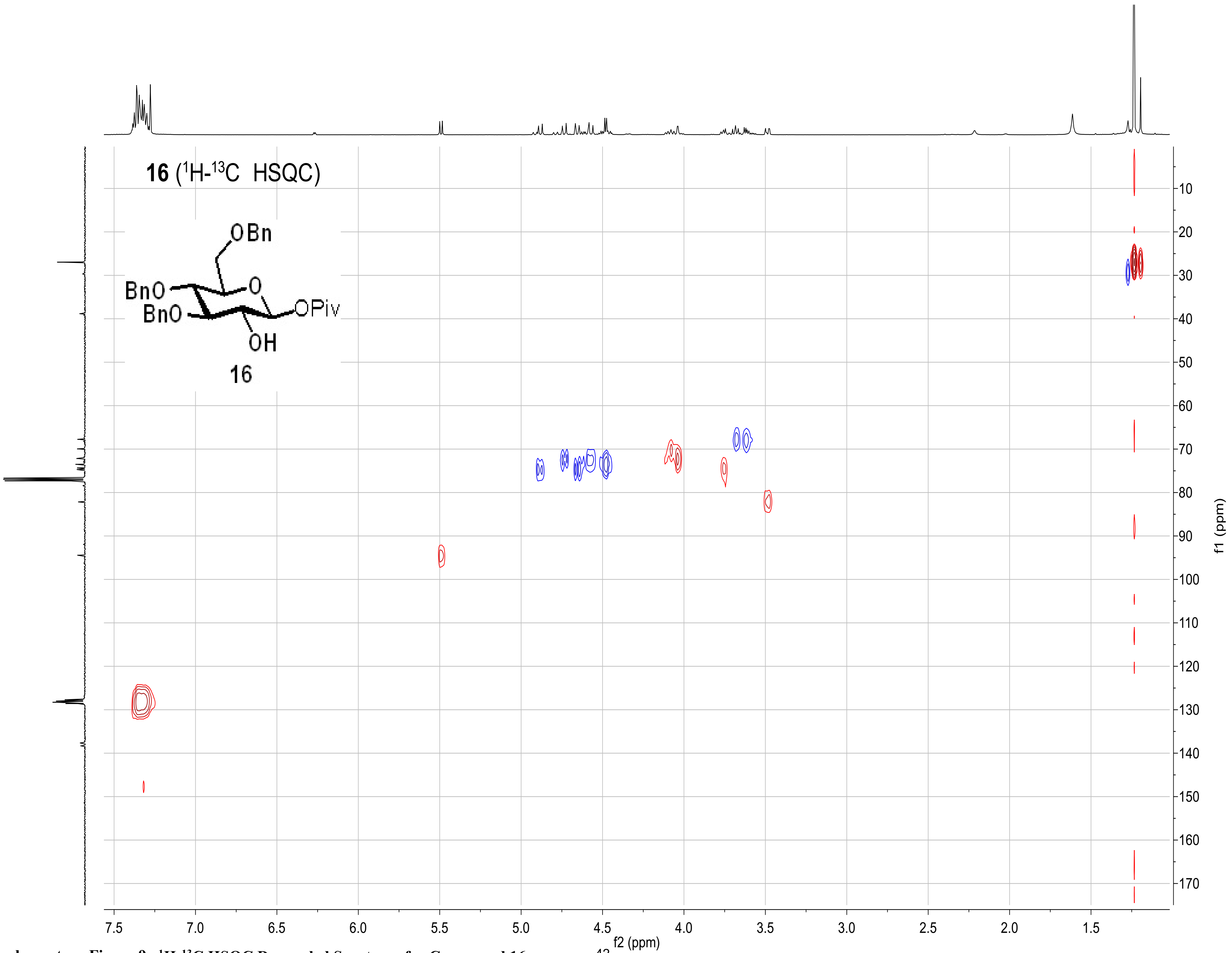

Supplementary Figure 9. ^{1}H-^{13}C HSQC Decoupled Spectrum for Compound 16

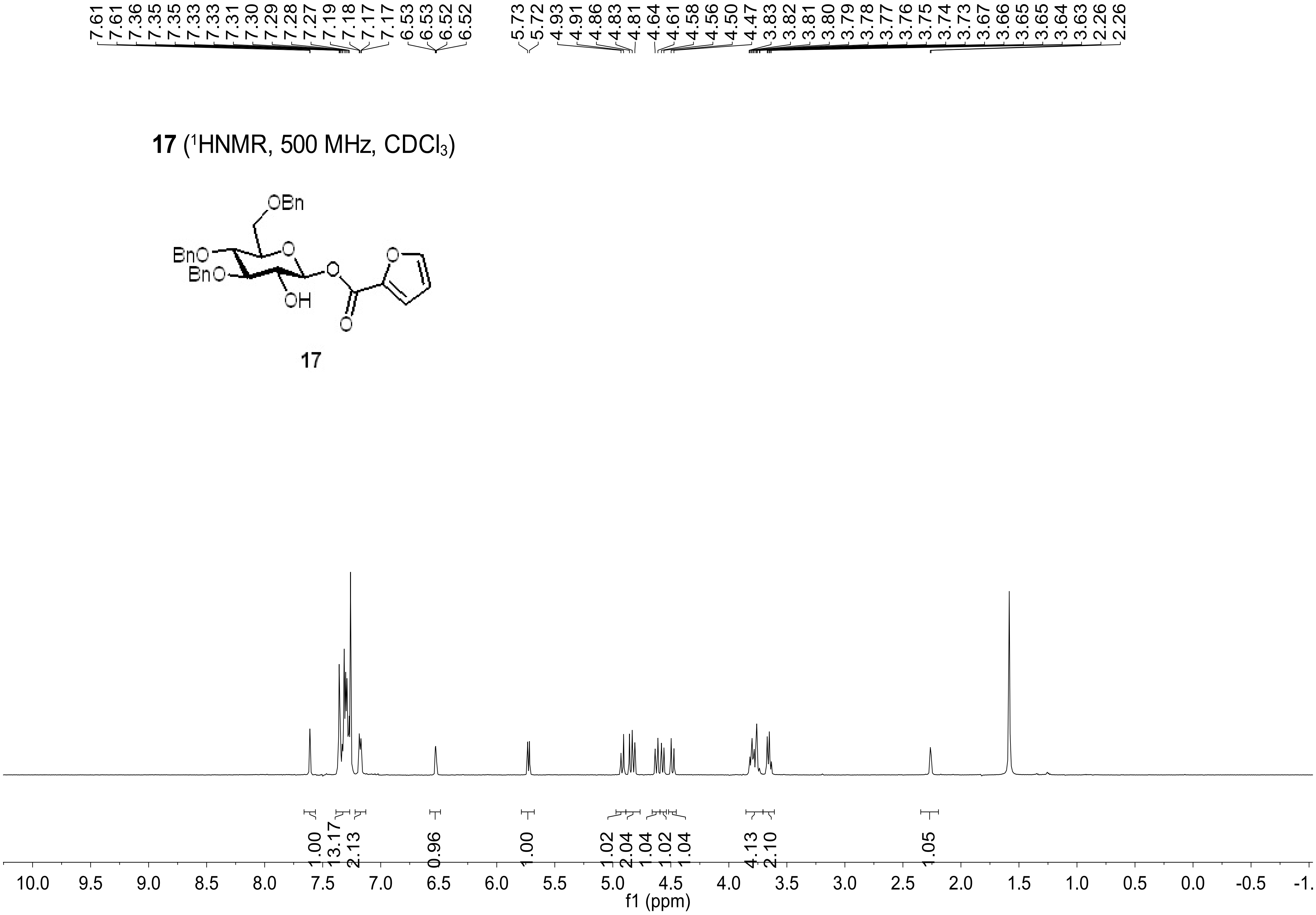

Supplementary Figure 10. ^{1}H NMR Spectrum for Compound 17

17 (^{13}CNMR, 101 MHz, $CDCl_3$)

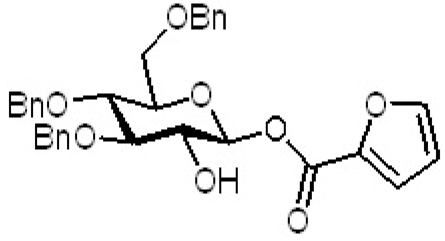

17

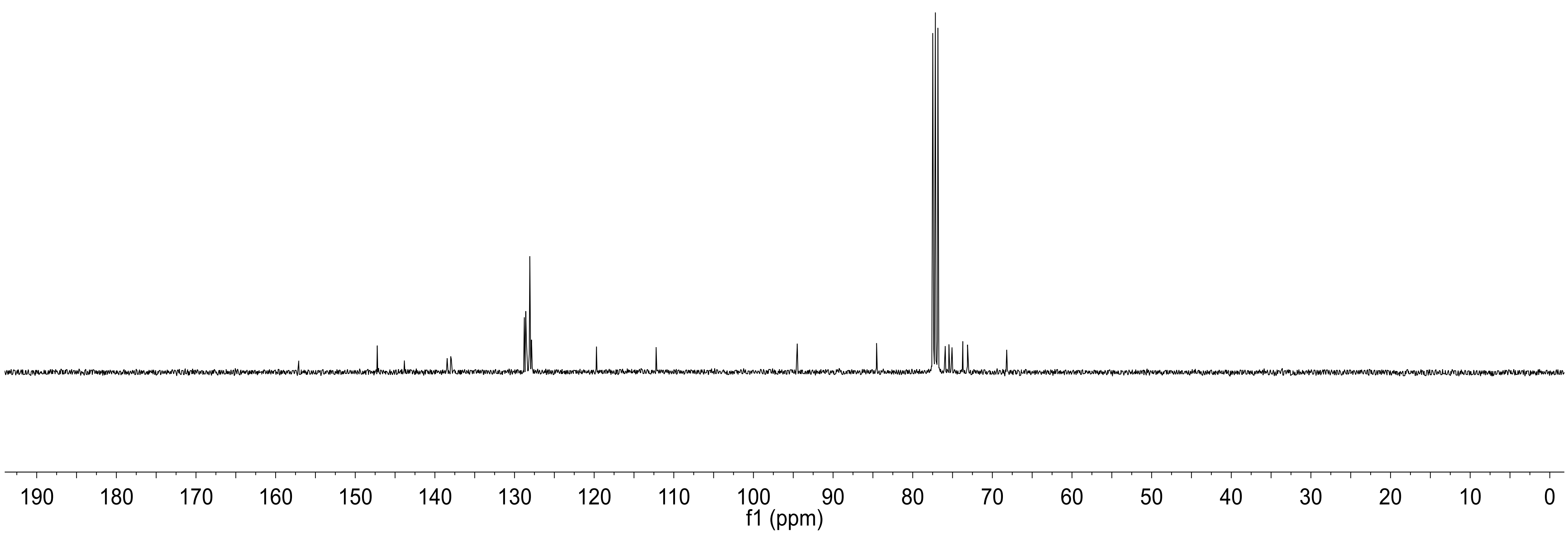

Supplementary Figure 11. ^{13}C NMR Spectrum for Compound 17

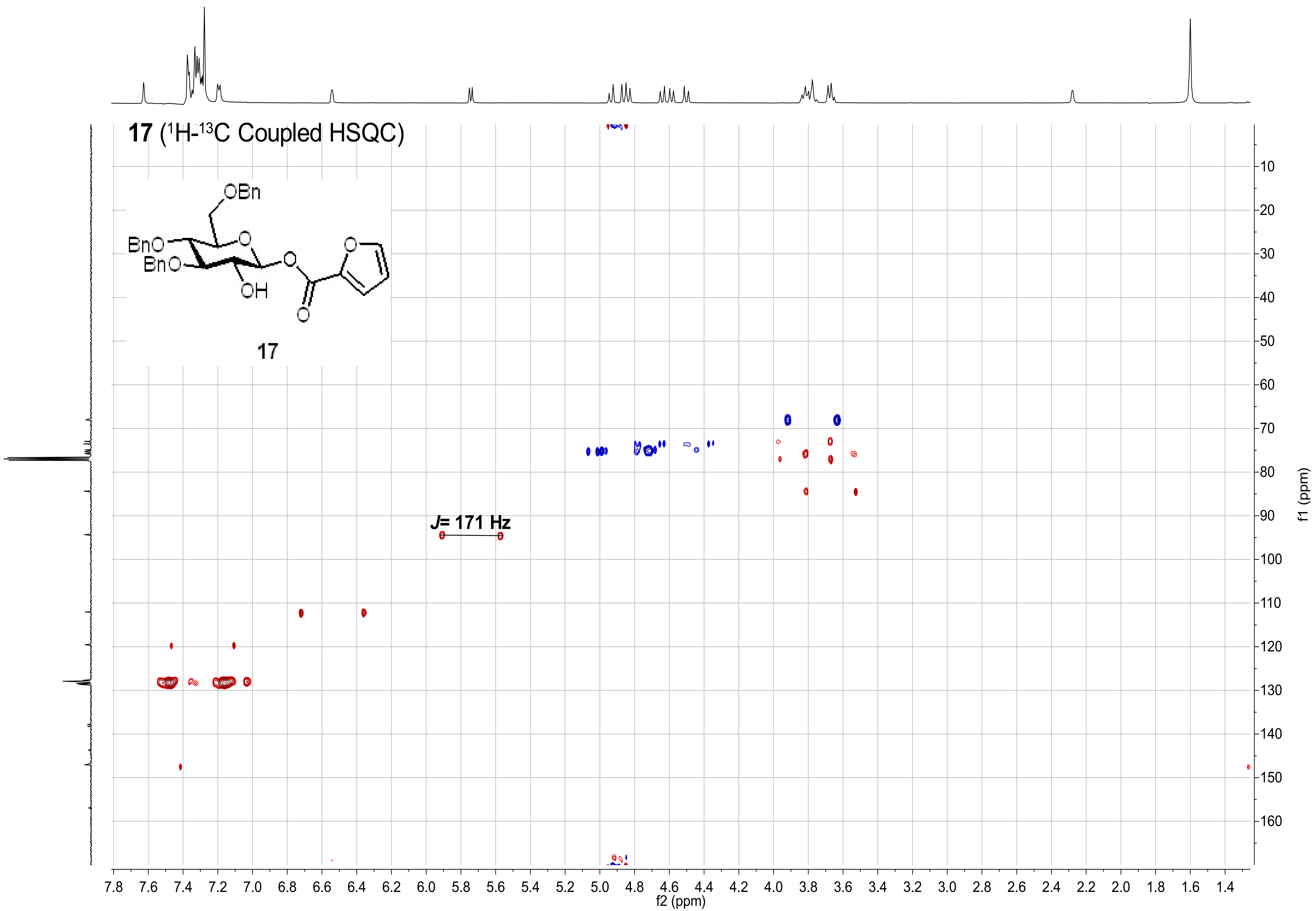

Supplementary Figure 12. ^{1}H-^{13}C HSQC Coupled Spectrum for Compound 17

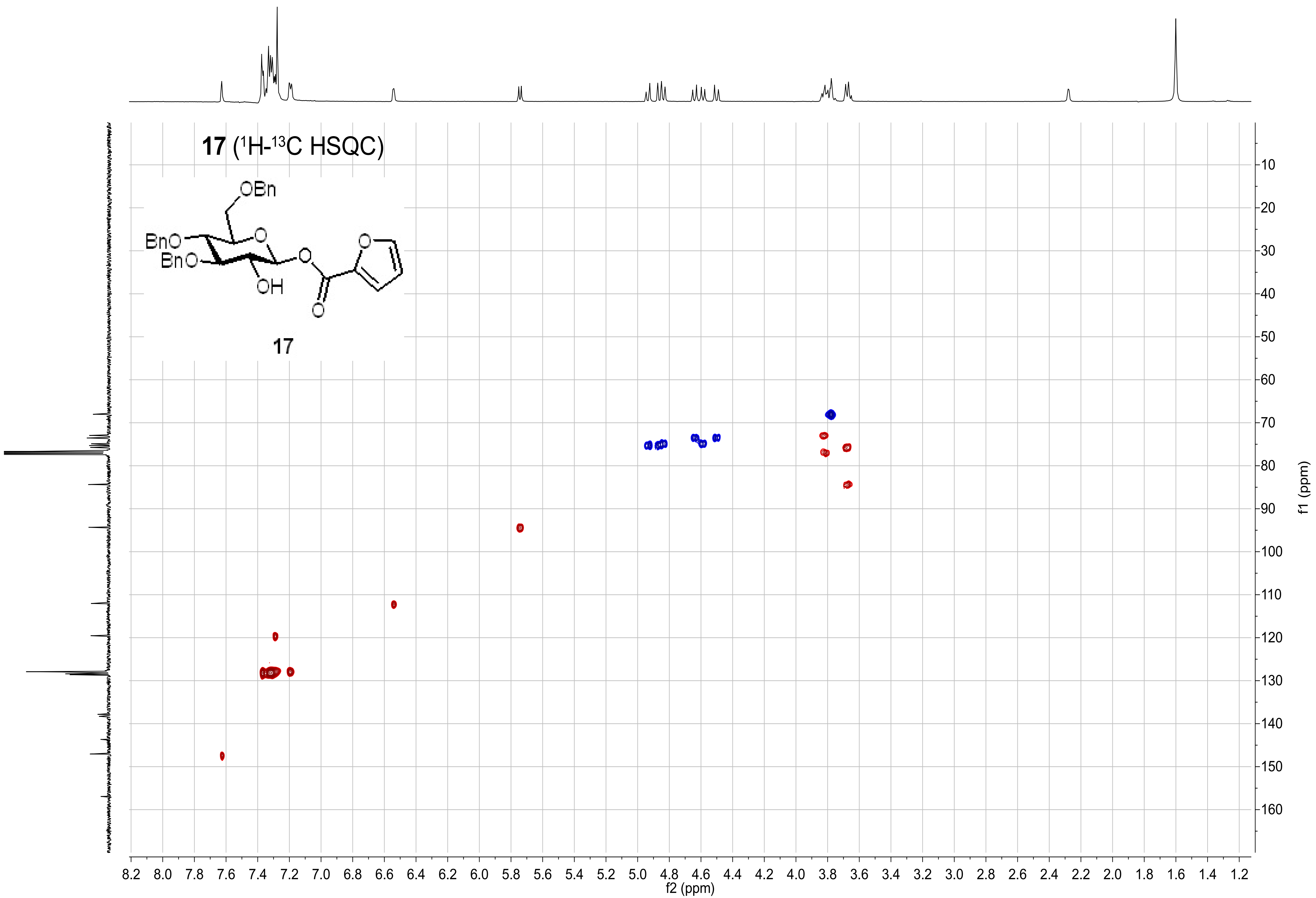

Supplementary Figure 13. ^{1}H-^{13}C HSQC Decoupled Spectrum for Compound 17

18 (^{1}HNMR, 500 MHz, $CDCl_3$)

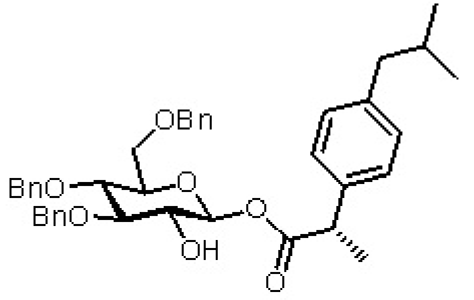

18

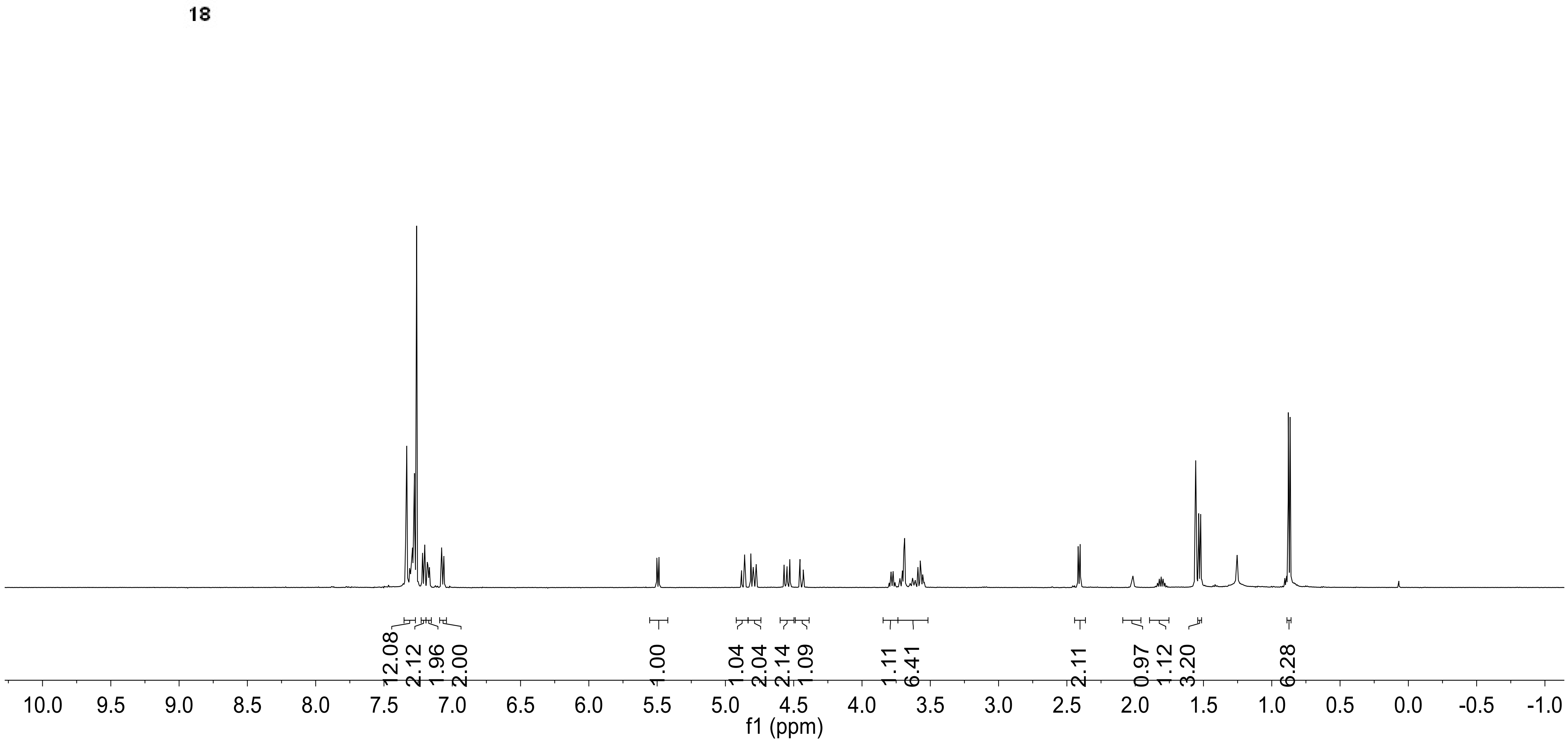

Supplementary Figure 14. ^{1}H NMR Spectrum for Compound 18

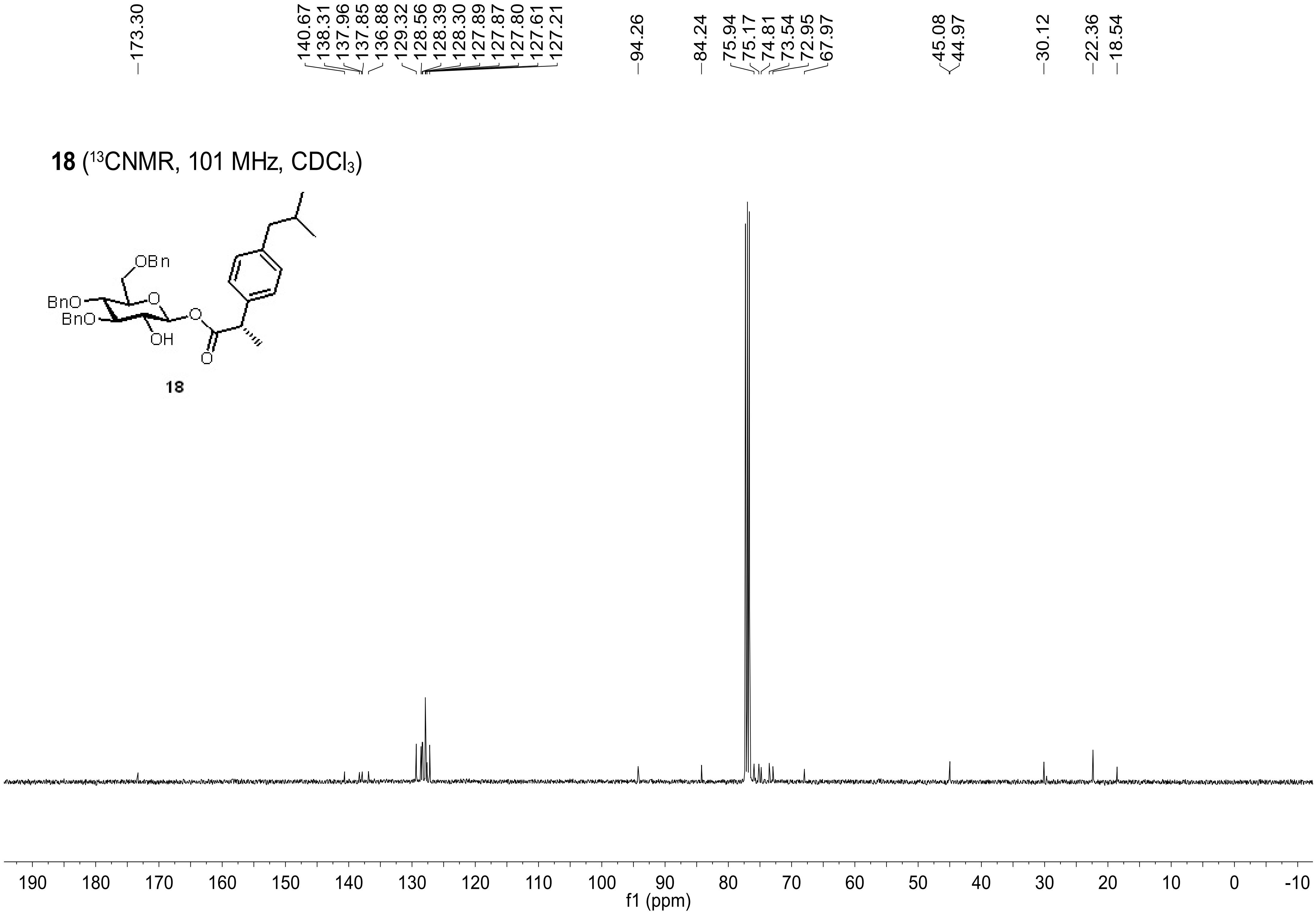

Supplementary Figure 15. ^{13}C NMR Spectrum for Compound 18

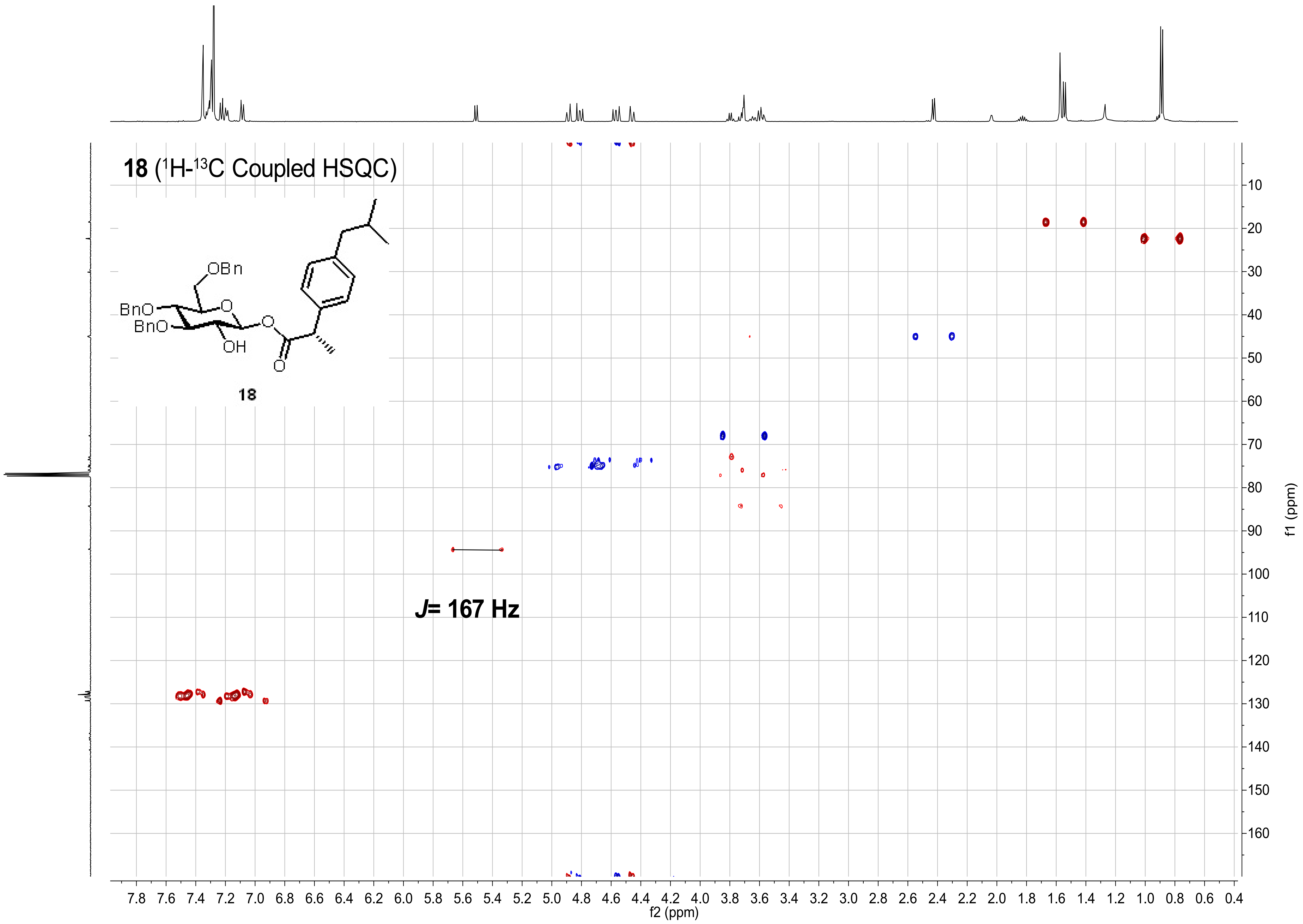

Supplementary Figure 16. ^{1}H-^{13}C **HSQC Coupled Spectrum for Compound 18**

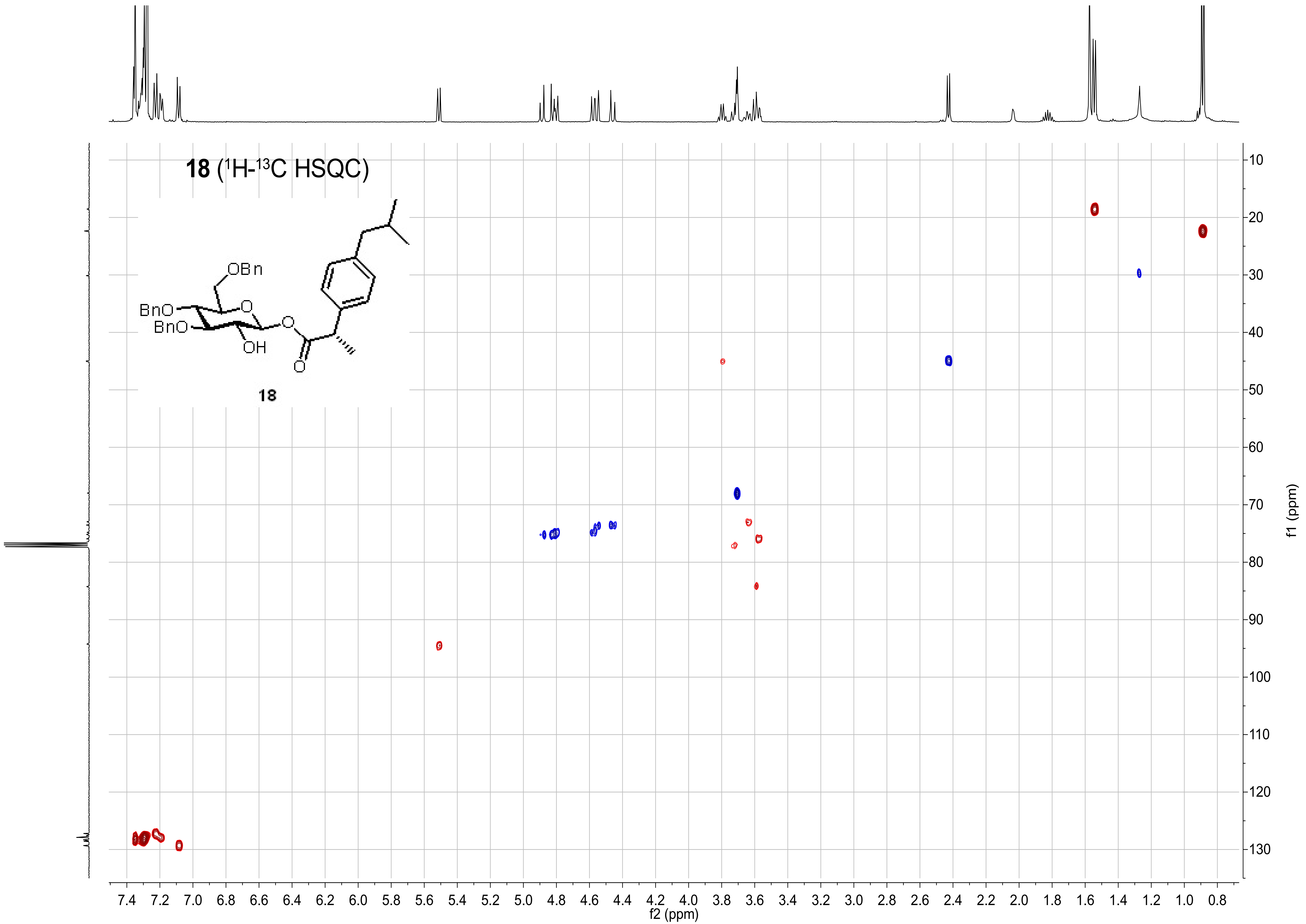

Supplementary Figure 17. ^{1}H-^{13}C HSQC Decoupled Spectrum for Compound 18

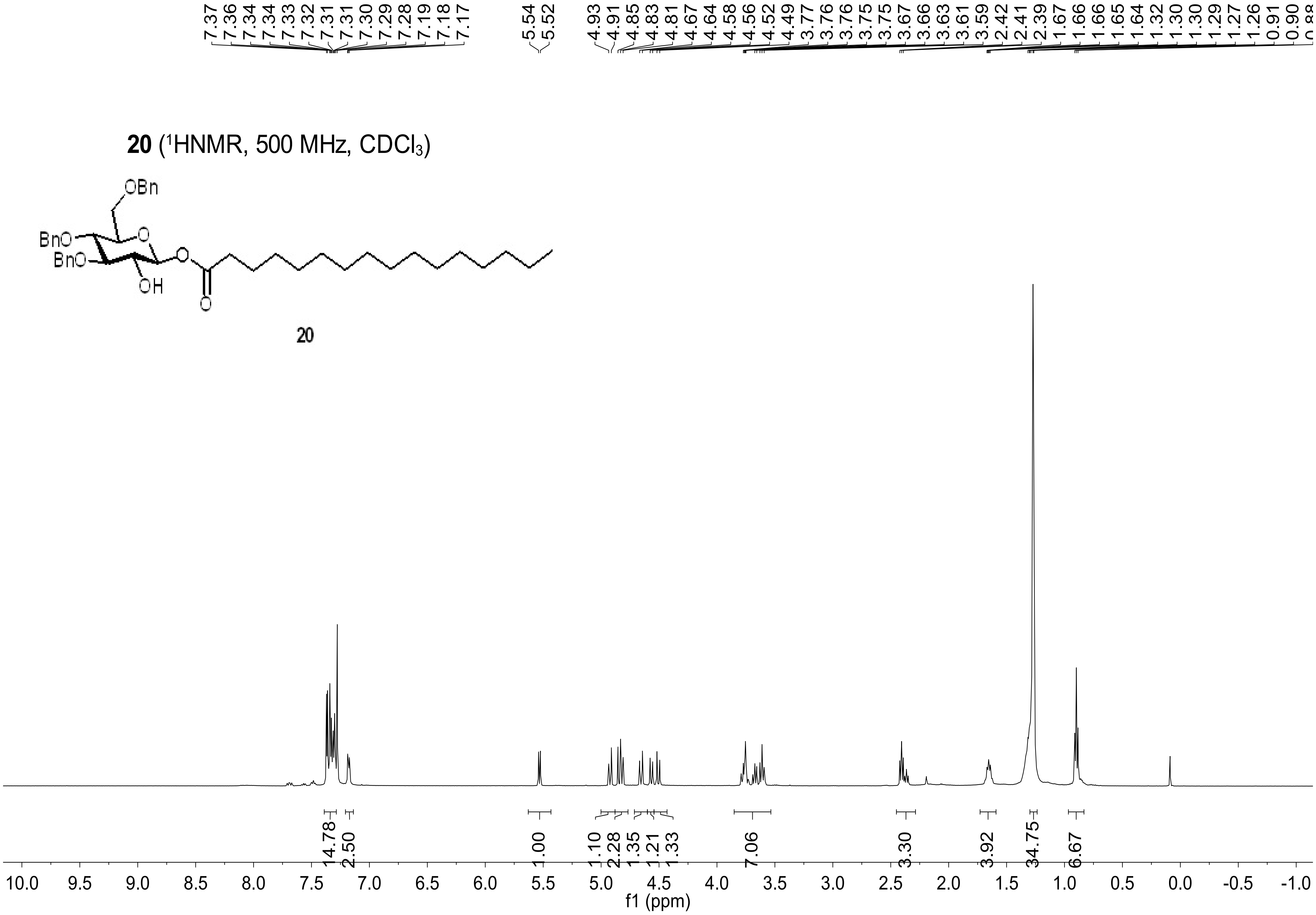

Supplementary Figure 18. ^{1}H NMR Spectrum for Compound 20

20 (^{13}C NMR, 101 MHz, $CDCl_3$)

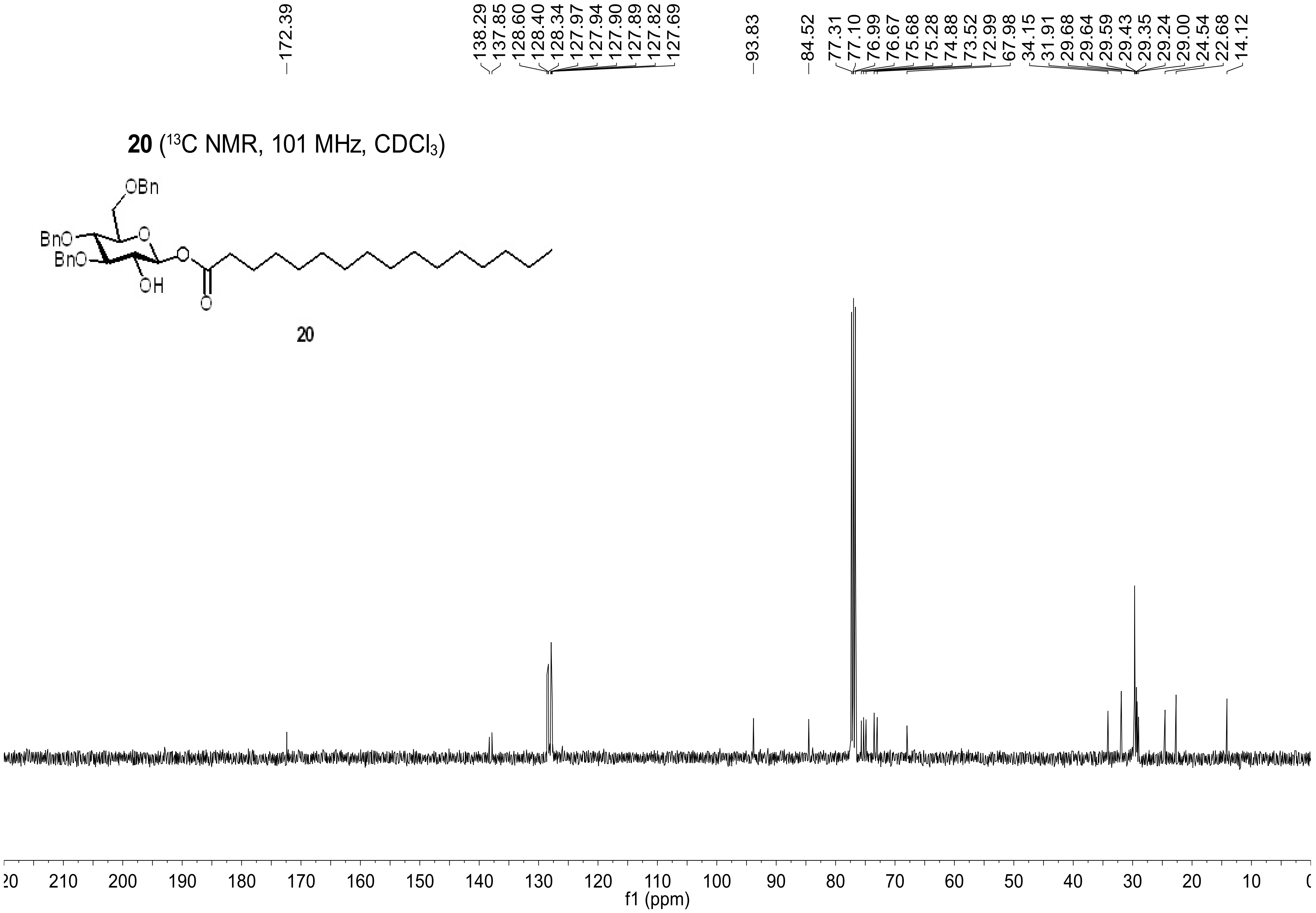

Supplementary Figure 19. ^{13}C NMR Spectrum for Compound 20

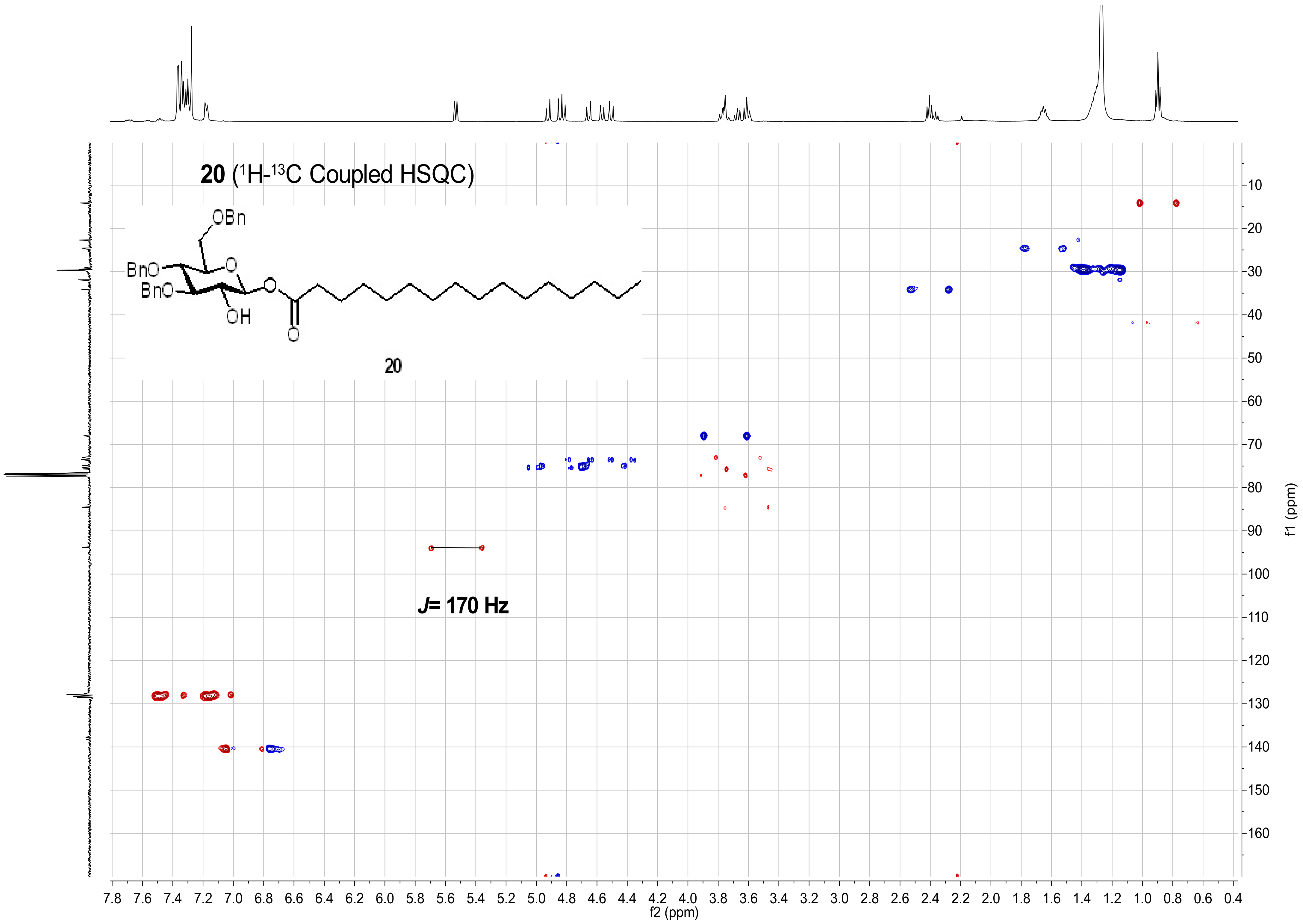

Supplementary Figure 20. ^{1}H-^{13}C HSQC Coupled Spectrum for Compound 20

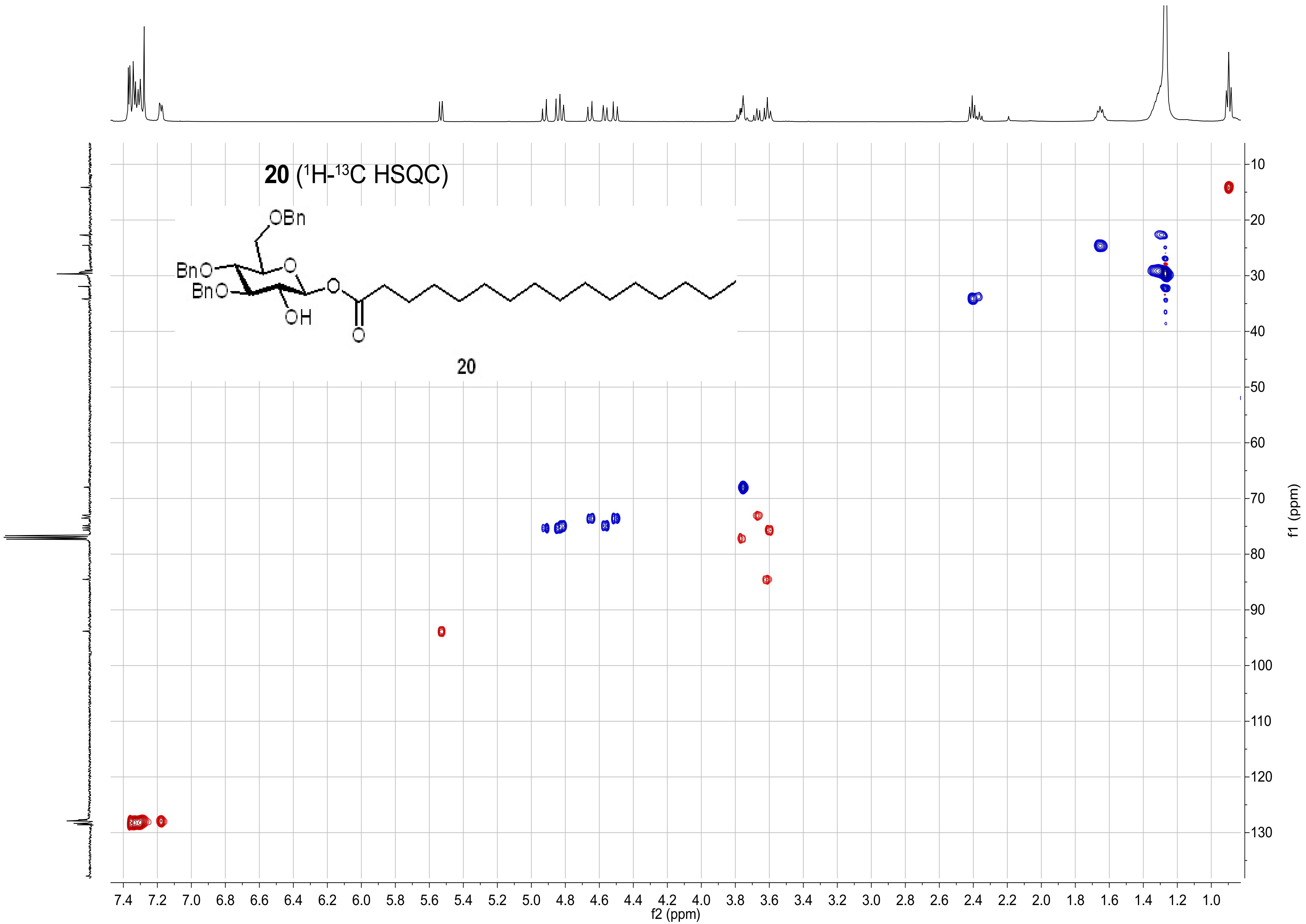

Supplementary Figure 21. **^{1}H-^{13}C HSQC Decoupled Spectrum for Compound 20**

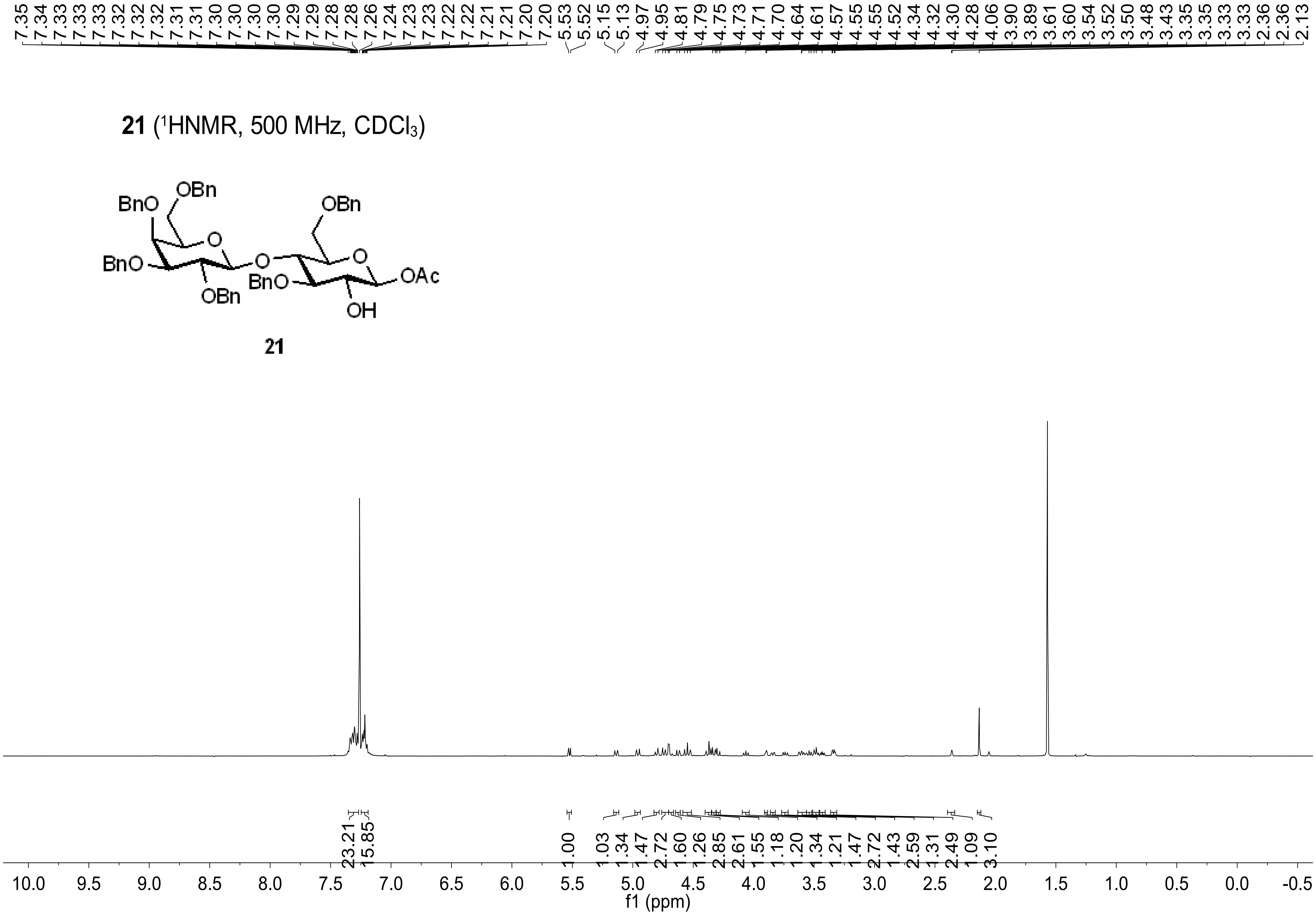

Supplementary Figure 22. ^{1}H NMR Spectrum for Compound 21

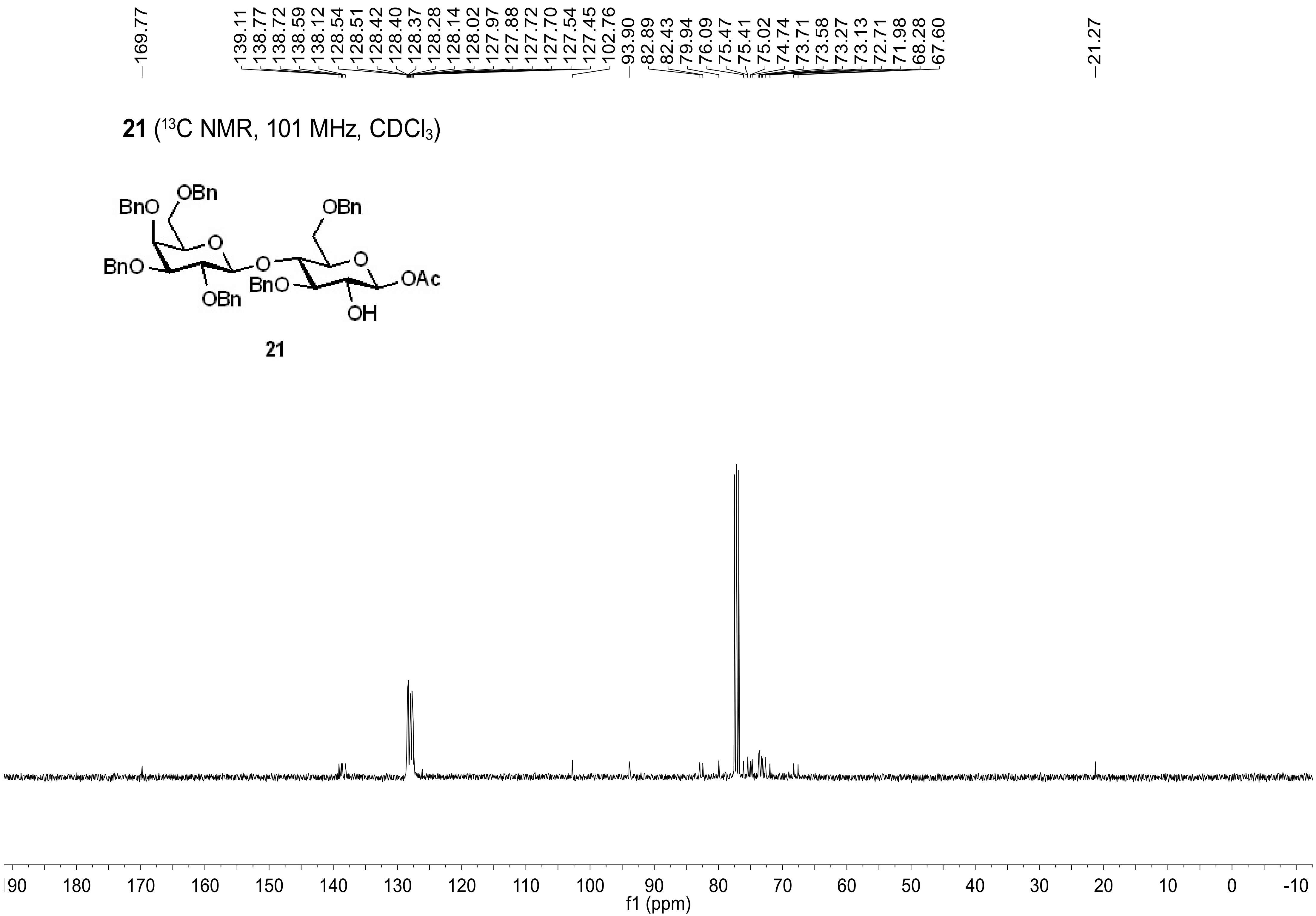

Supplementary Figure 23. ^{13}C **NMR Spectrum for Compound 21**

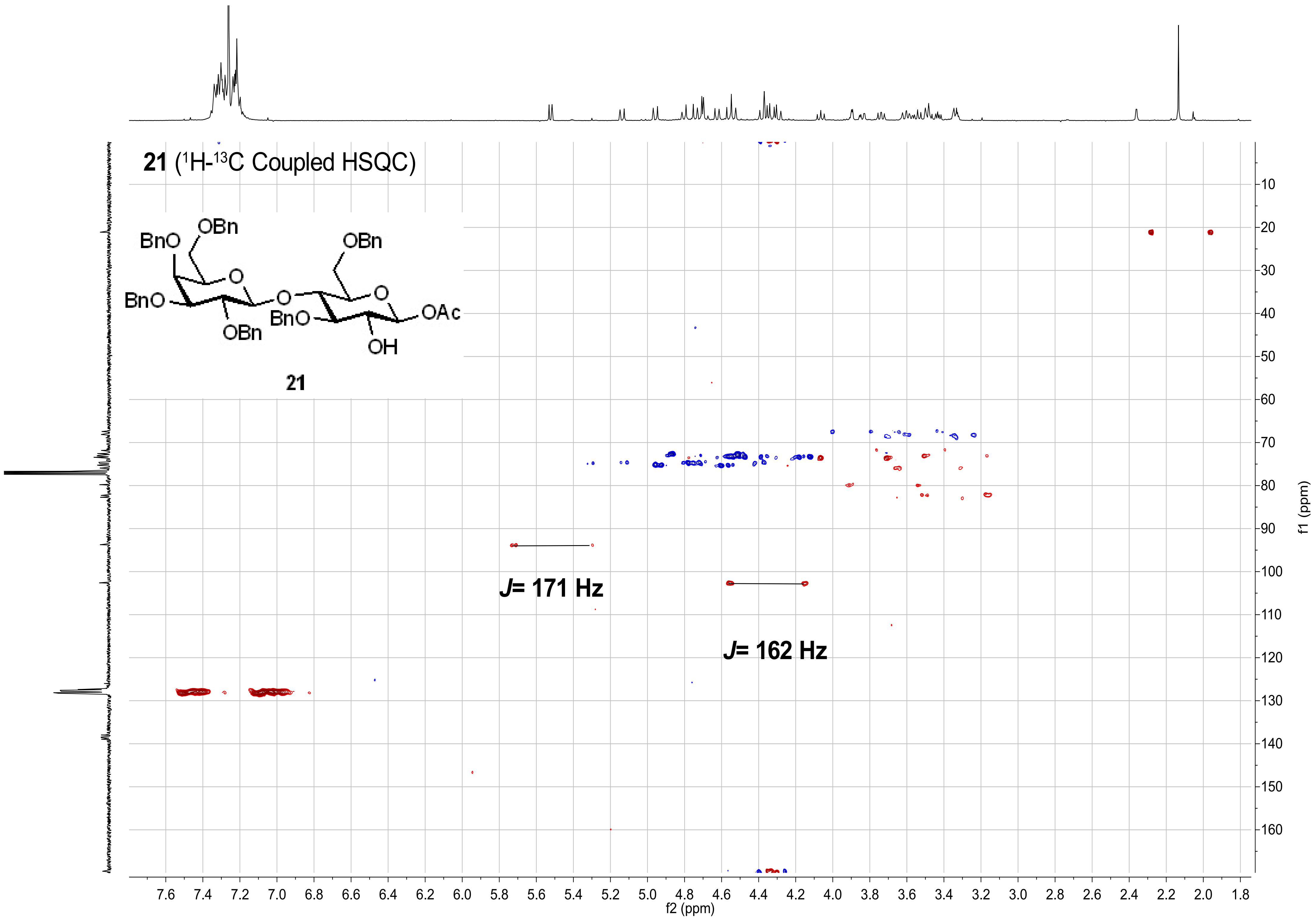

Supplementary Figure 24. ^{1}H-^{13}C HSQC Coupled Spectrum for Compound 21

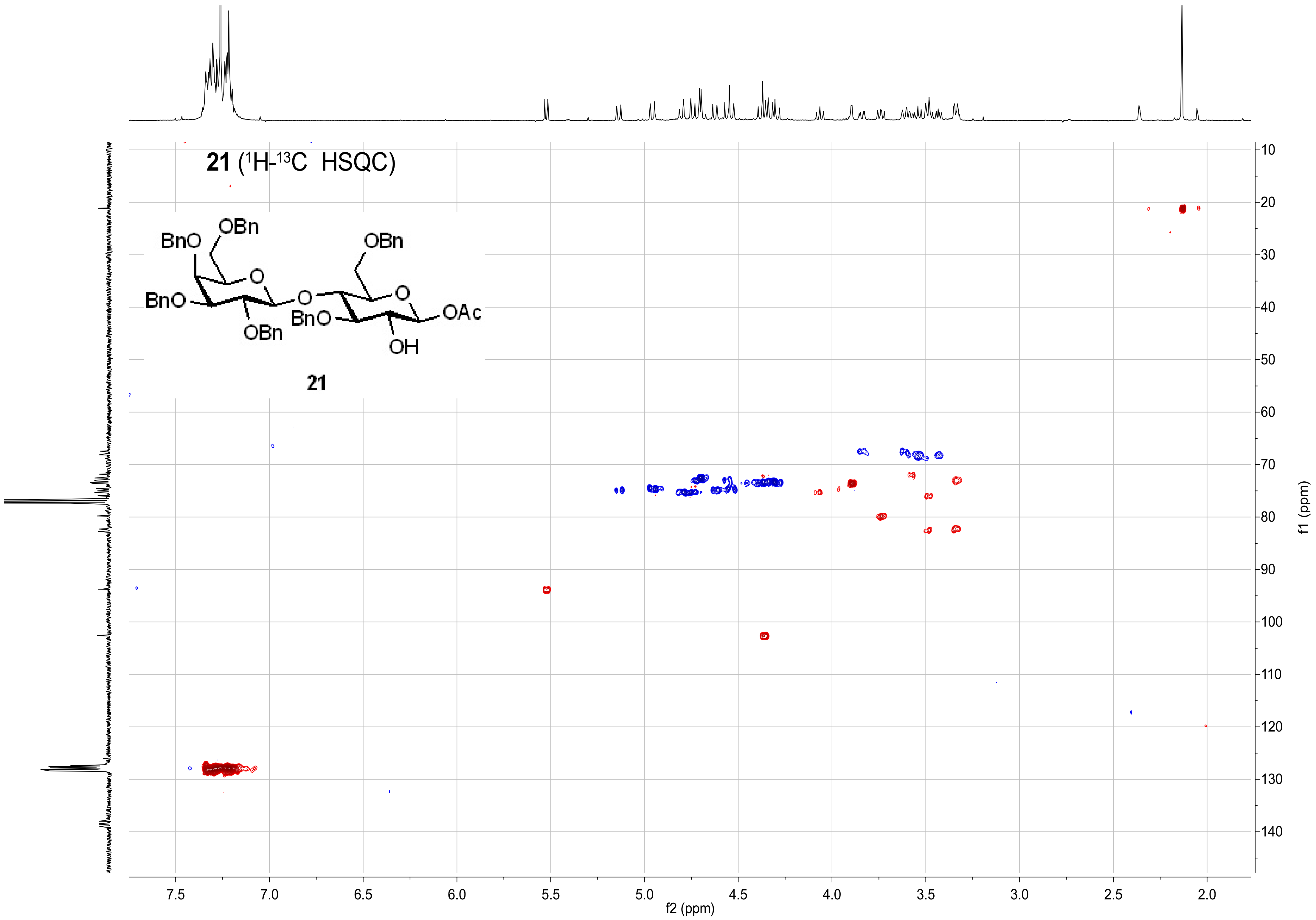

Supplementary Figure 25. ^{1}H-^{13}C HSQC Decoupled Spectrum for Compound 21

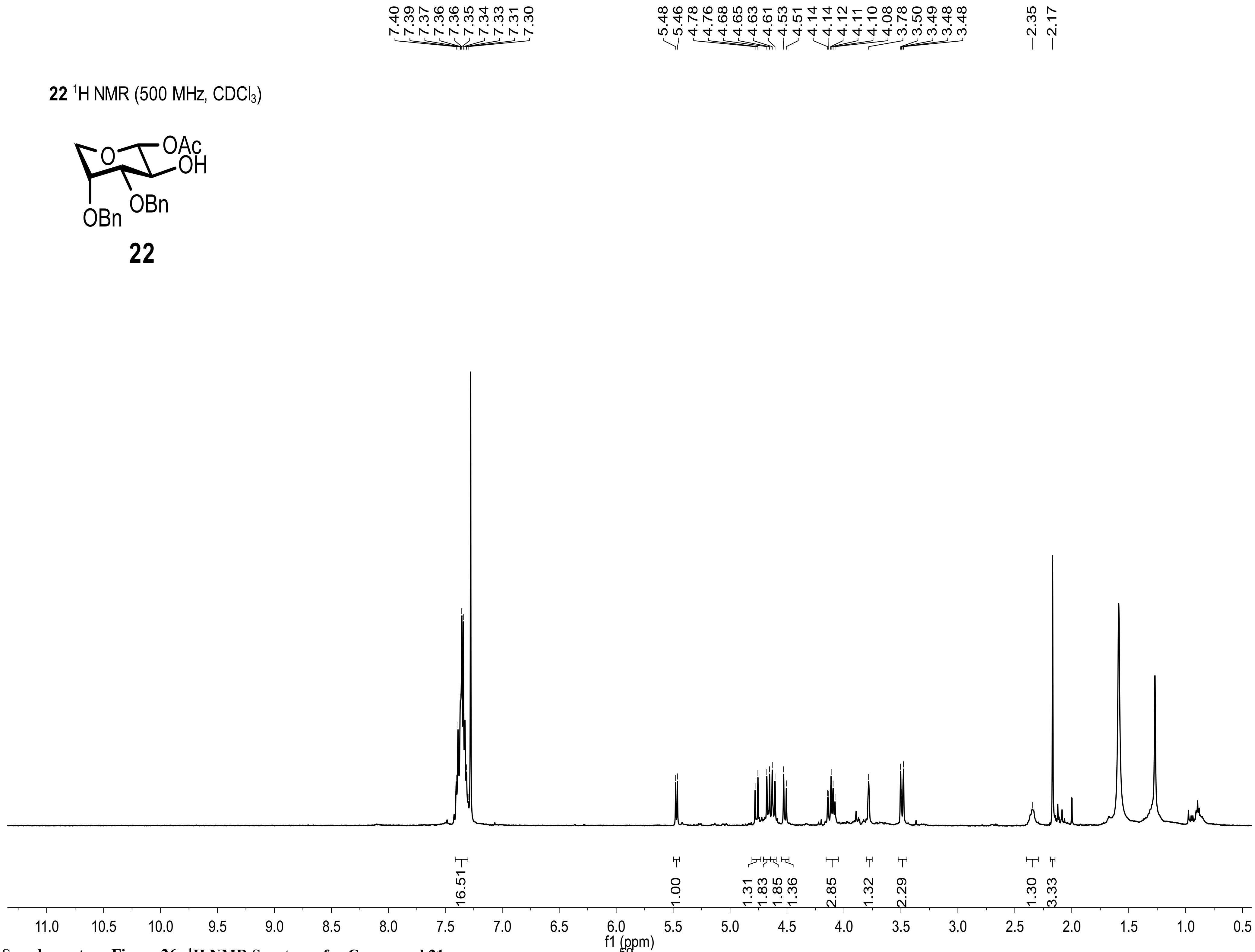

Supplementary Figure 26. ^{1}H NMR Spectrum for Compound 21

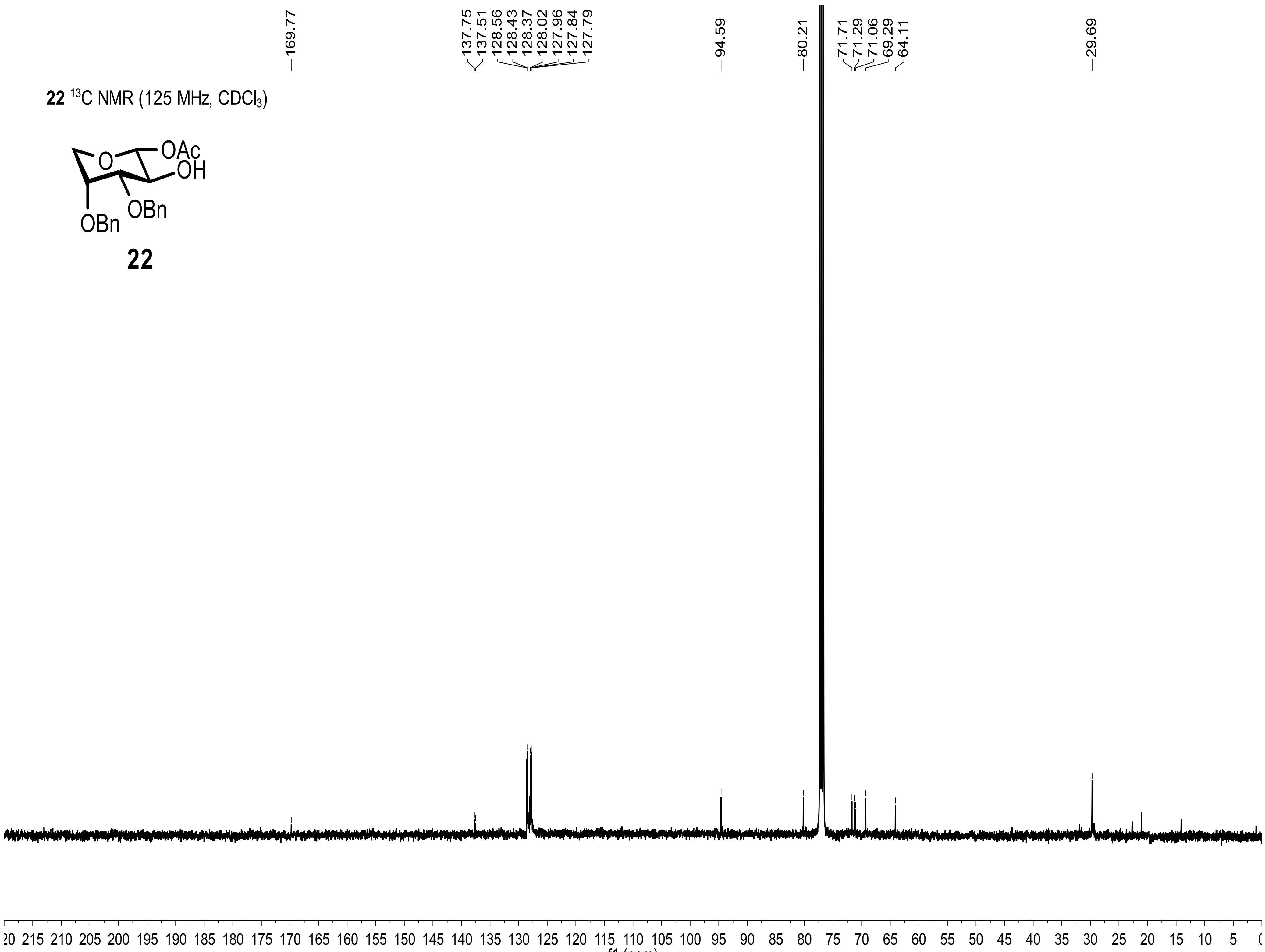

Supplementary Figure 27. ^{13}C NMR Spectrum for Compound 22

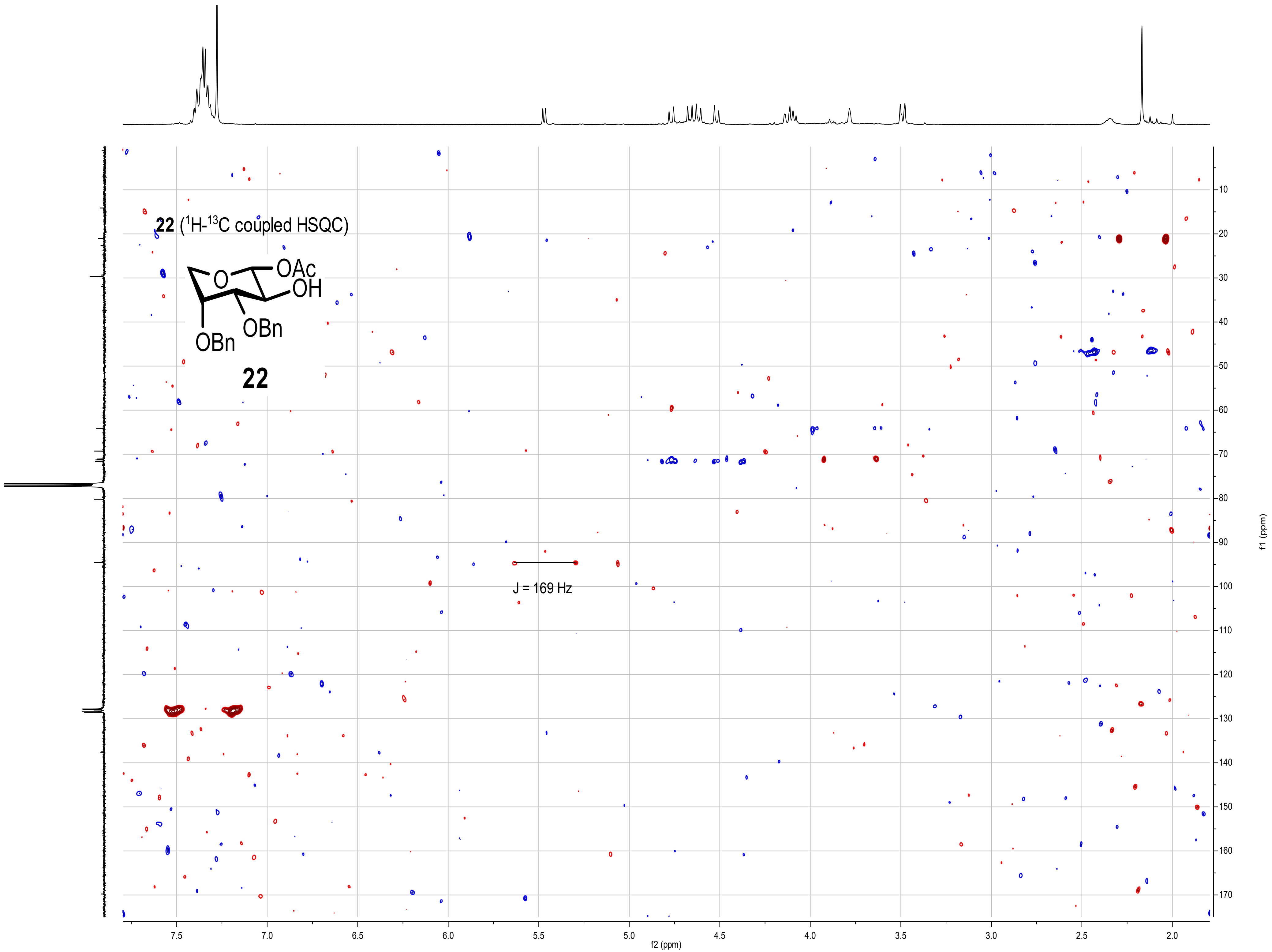

Supplementary Figure 28. **^{1}H-^{13}C HSQC Decoupled Spectrum for Compound 22**

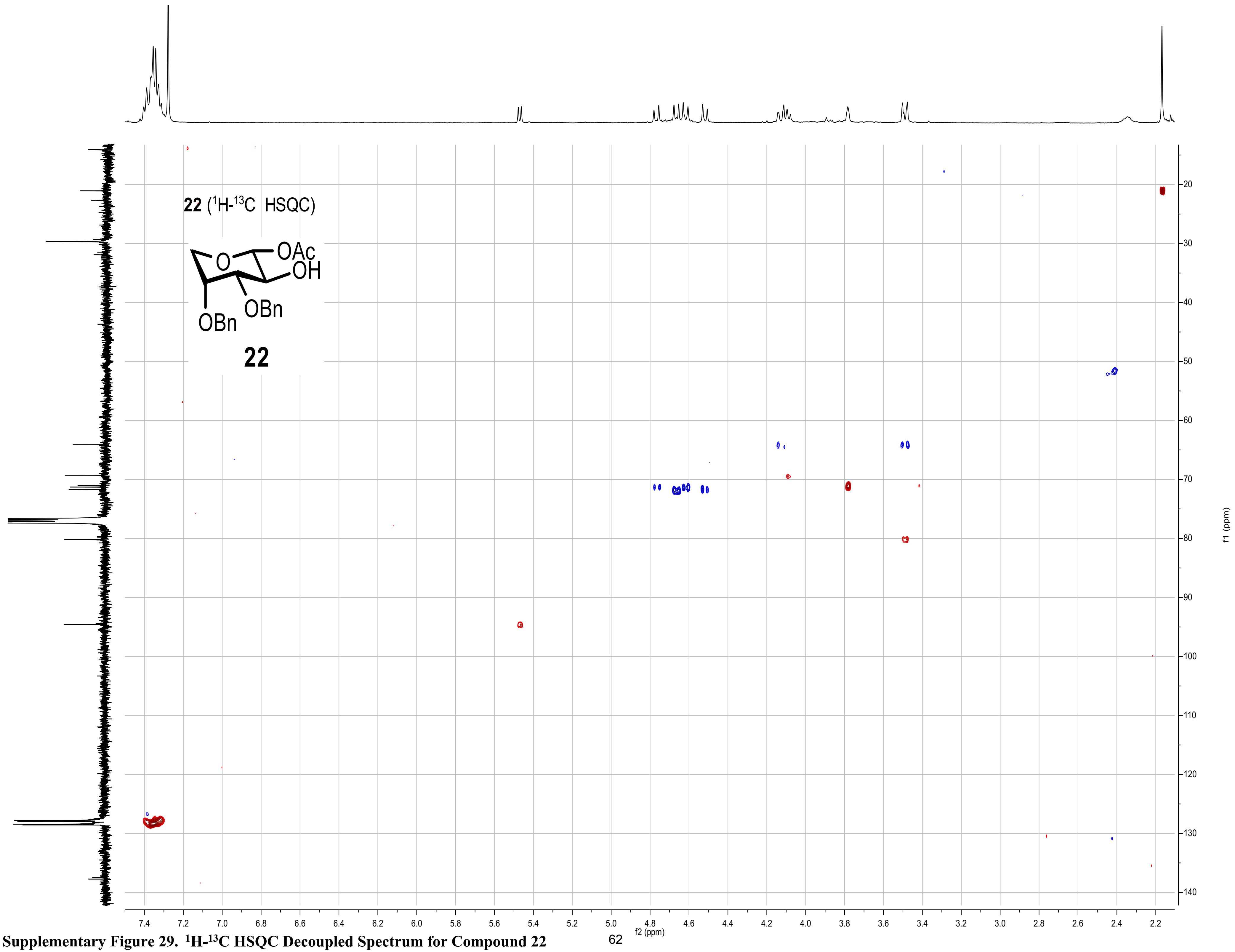

Supplementary Figure 29. ^{1}H-^{13}C HSQC Decoupled Spectrum for Compound 22

23 (^{1}HNMR, 400 MHz, $CDCl_3$)

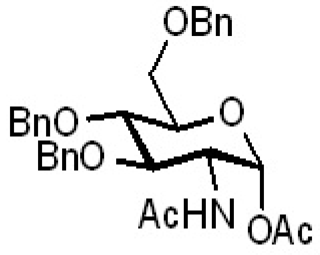

23

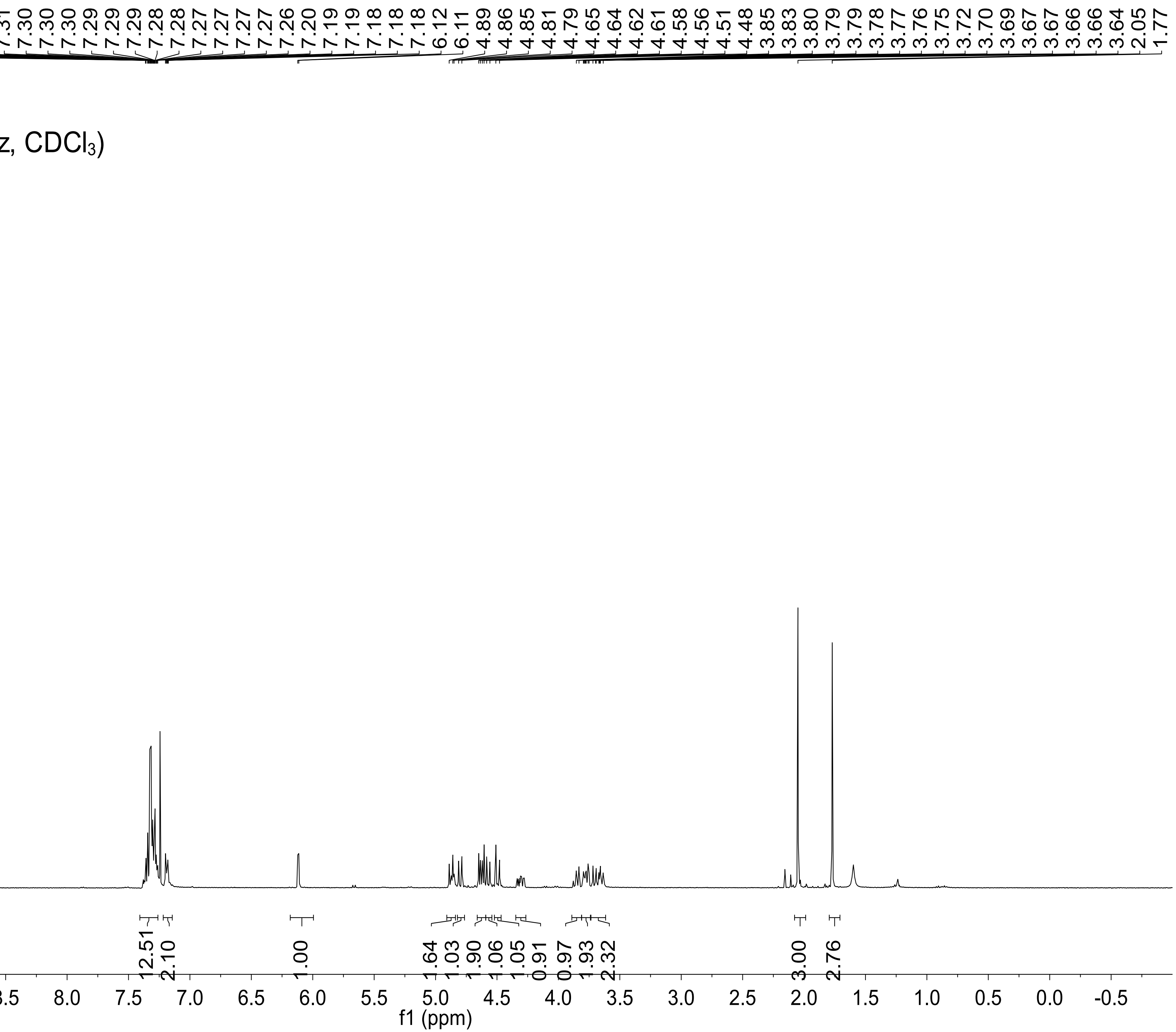

Supplementary Figure 30. ^{1}H NMR Spectrum for Compound 23

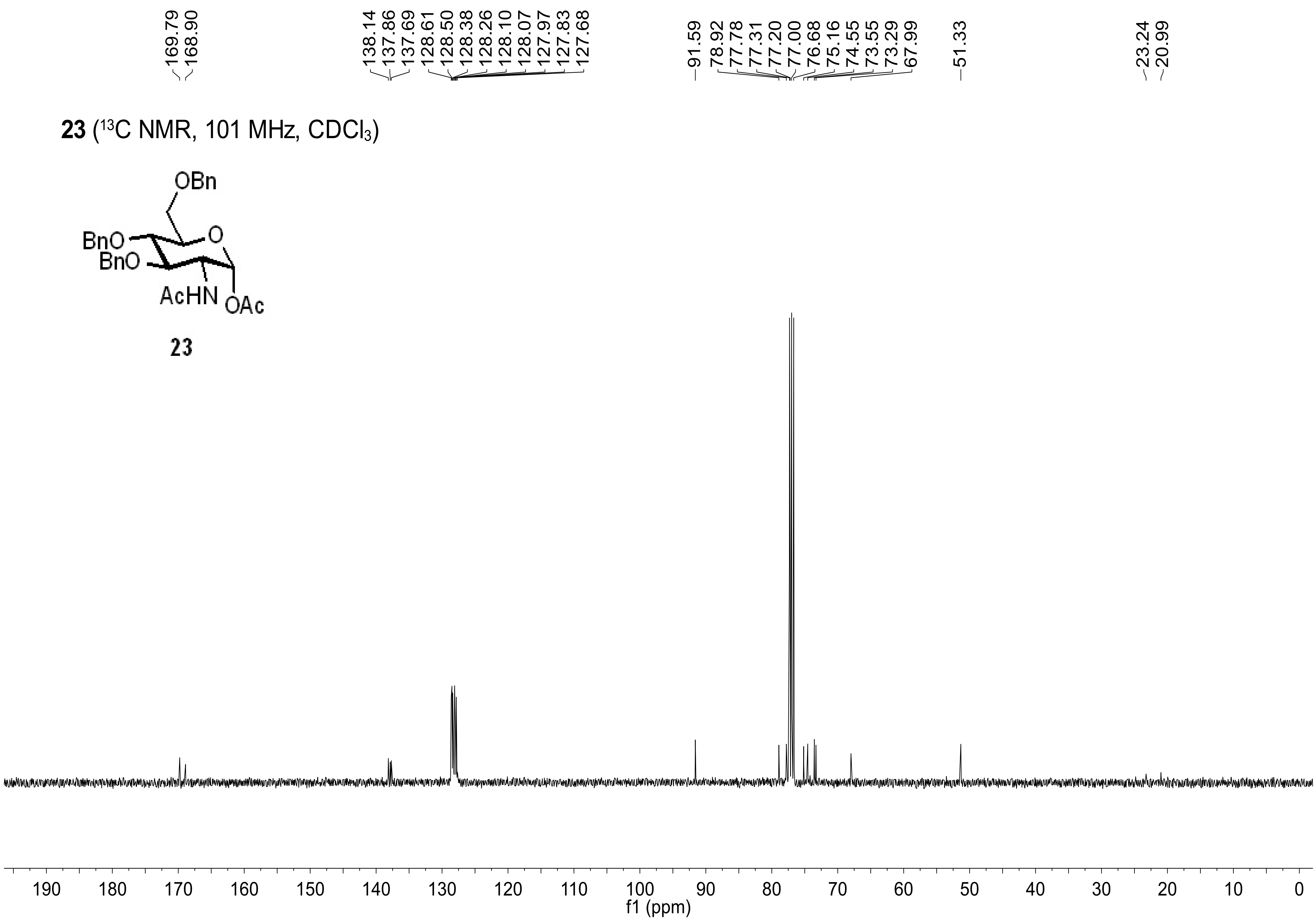

Supplementary Figure 31. ^{13}**C NMR Spectrum for Compound 23**

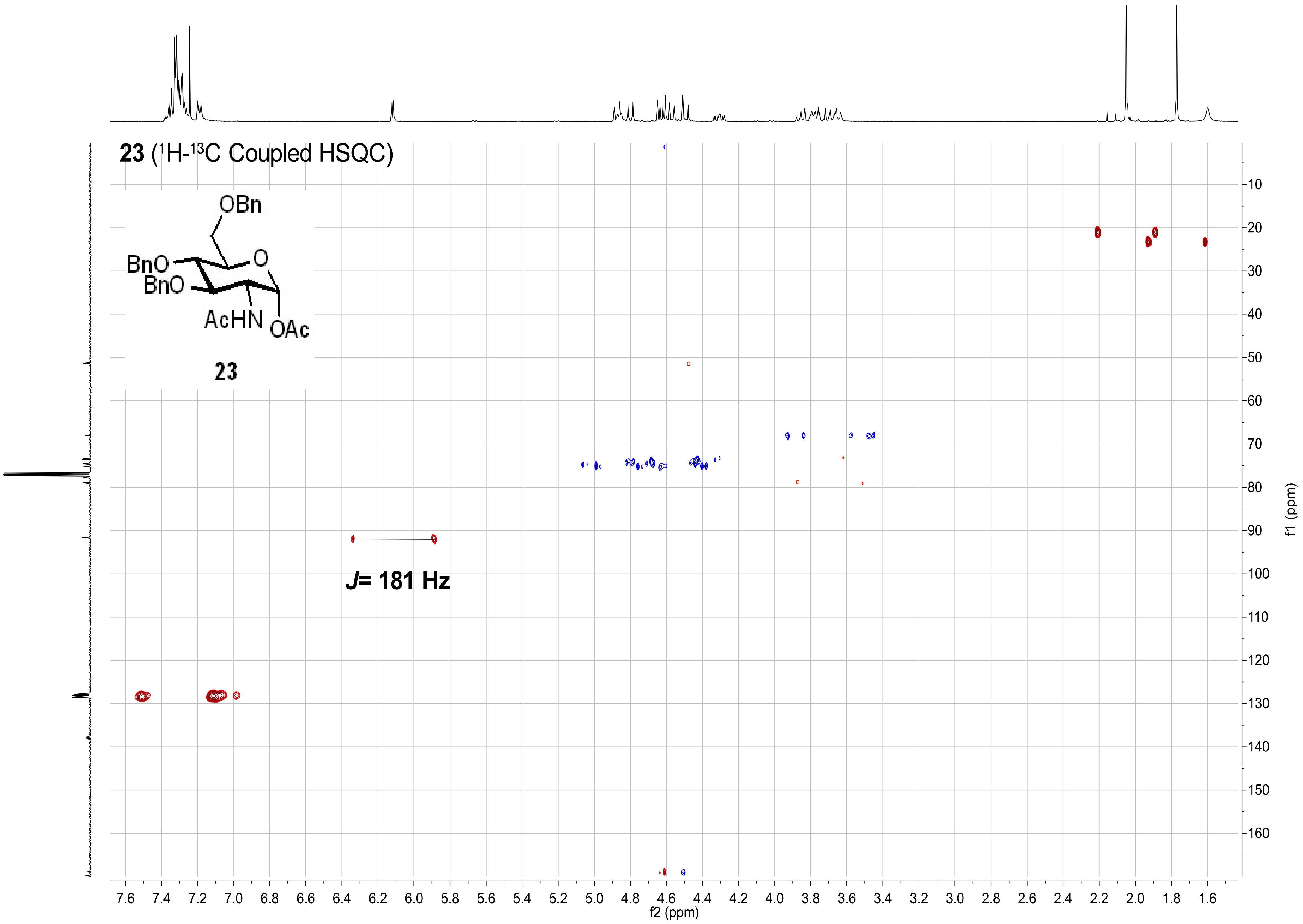

Supplementary Figure 32. ^{1}H-^{13}C HSQC Coupled Spectrum for Compound 23

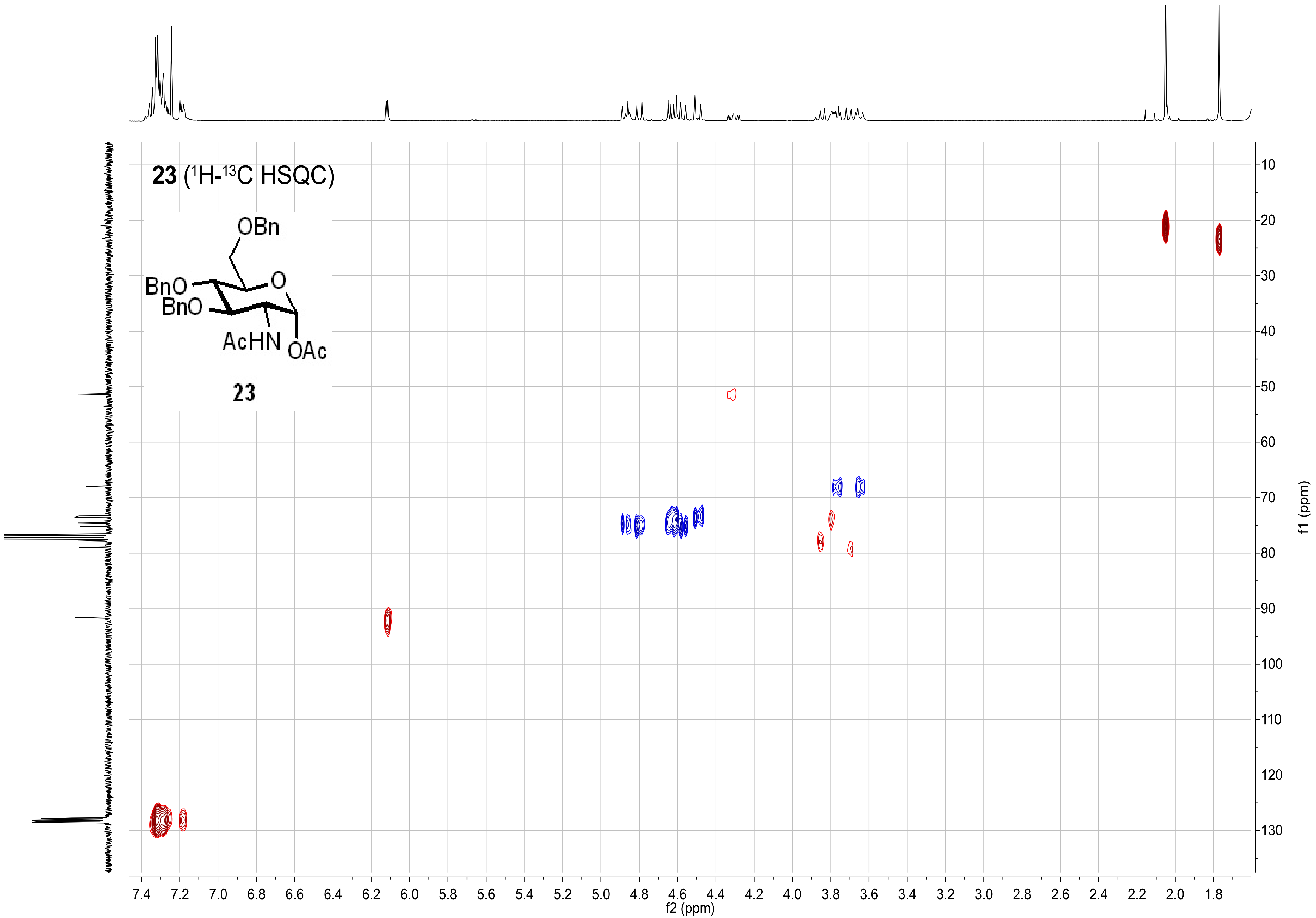

Supplementary Figure 33. ^{1}H-^{13}C HSQC Decoupled Spectrum for Compound 23

25 (^1H NMR, 300 MHz, $CDCl_3$)

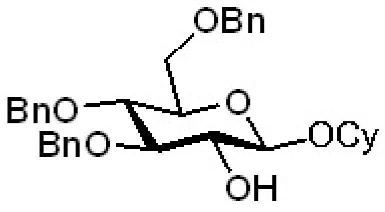

25

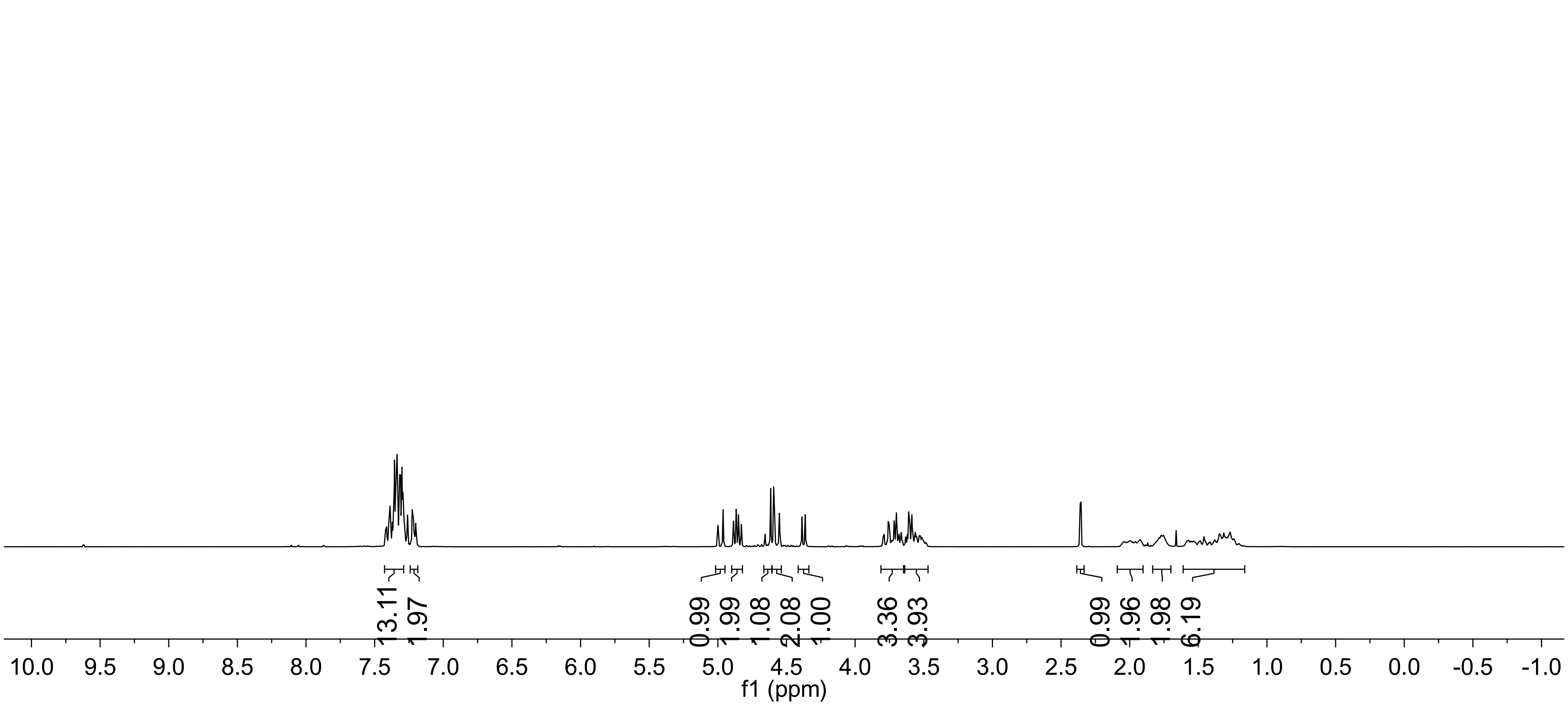

Supplementary Figure 34. ^1H NMR Spectrum for Compound 25

25 (^{13}C NMR, 75 MHz, $CDCl_3$)

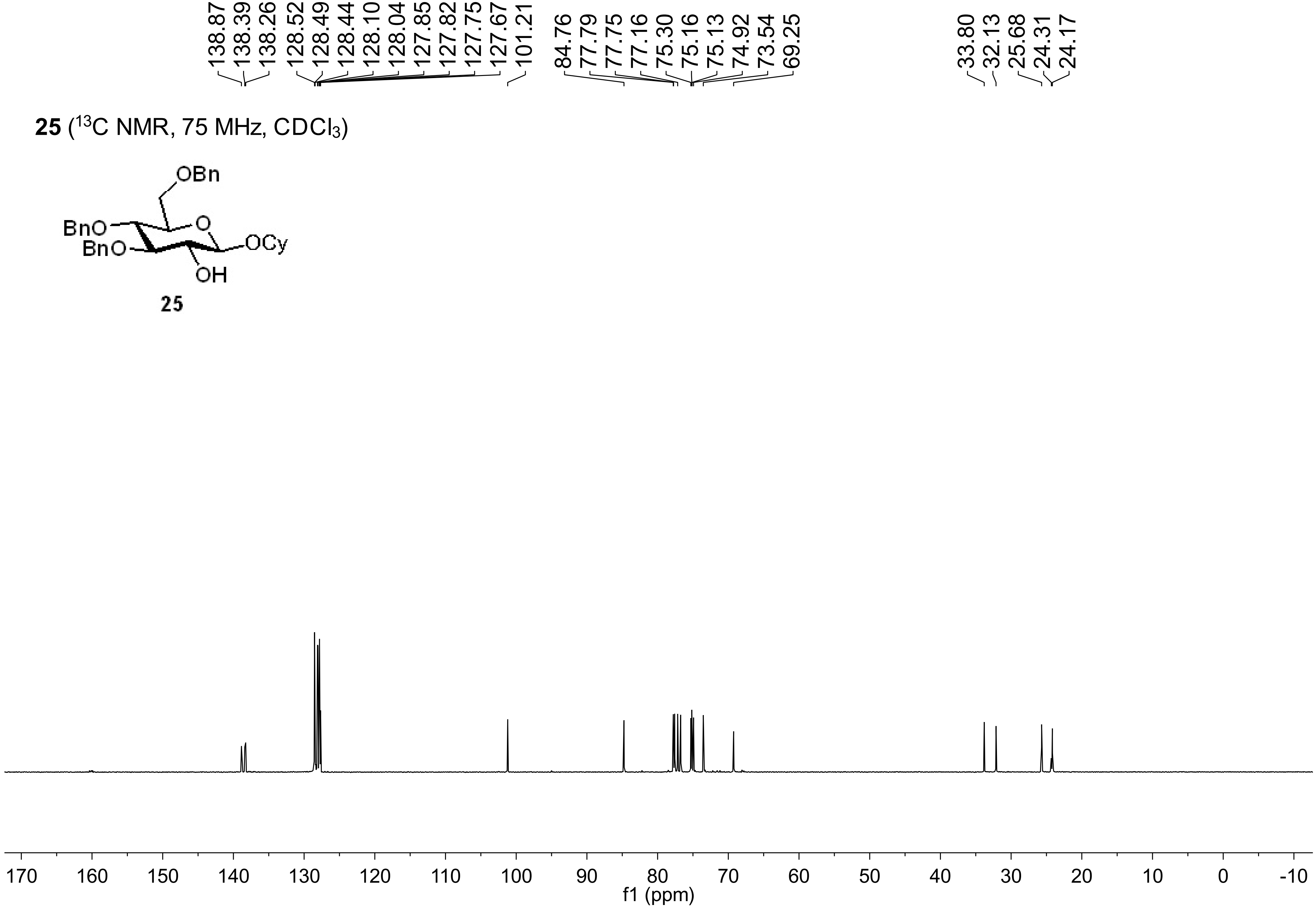

Supplementary Figure 35. 13C NMR Spectrum for Compound 25

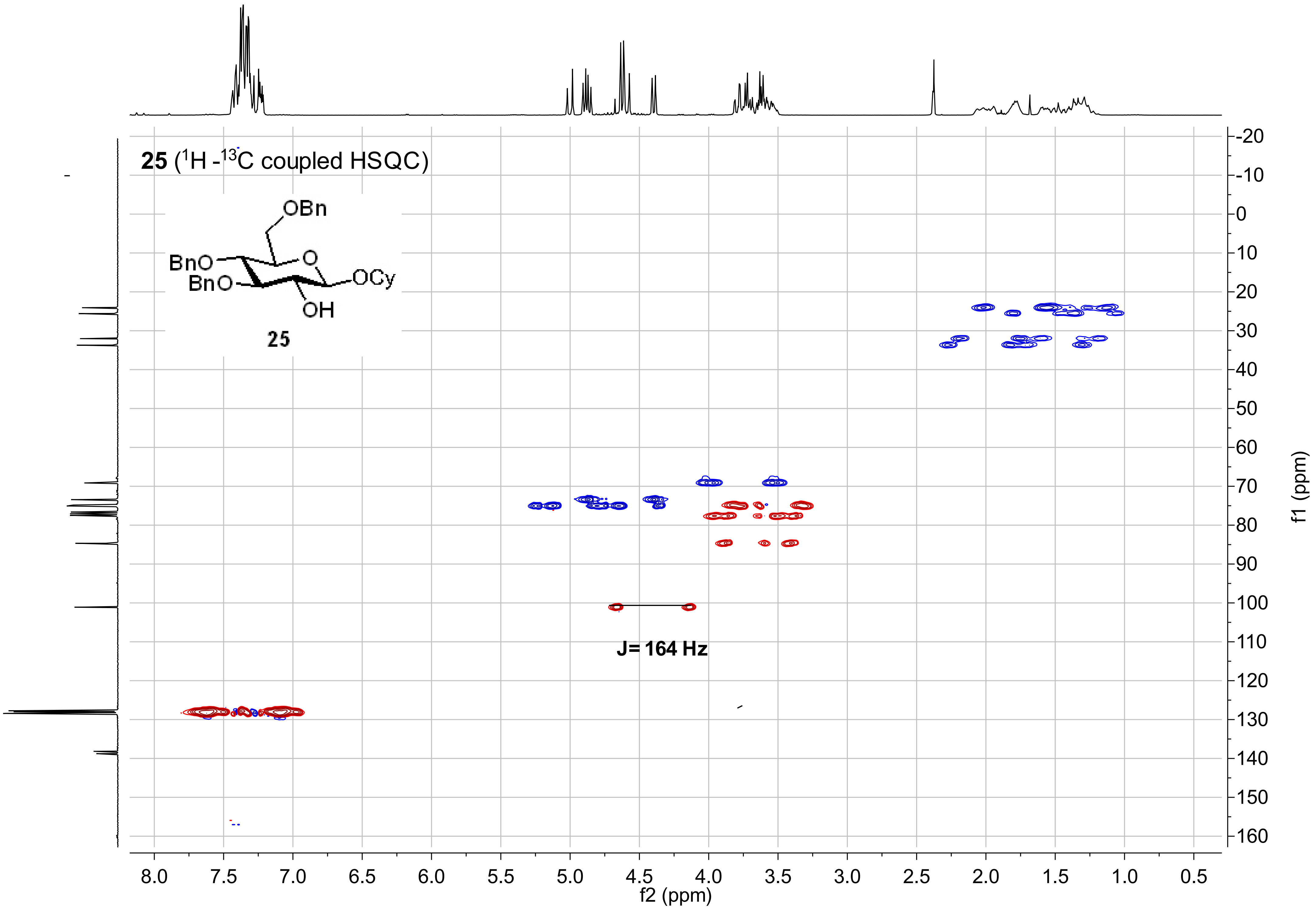

Supplementary Figure 36. ^{1}H-^{13}C **HSQC Coupled Spectrum for Compound 25**

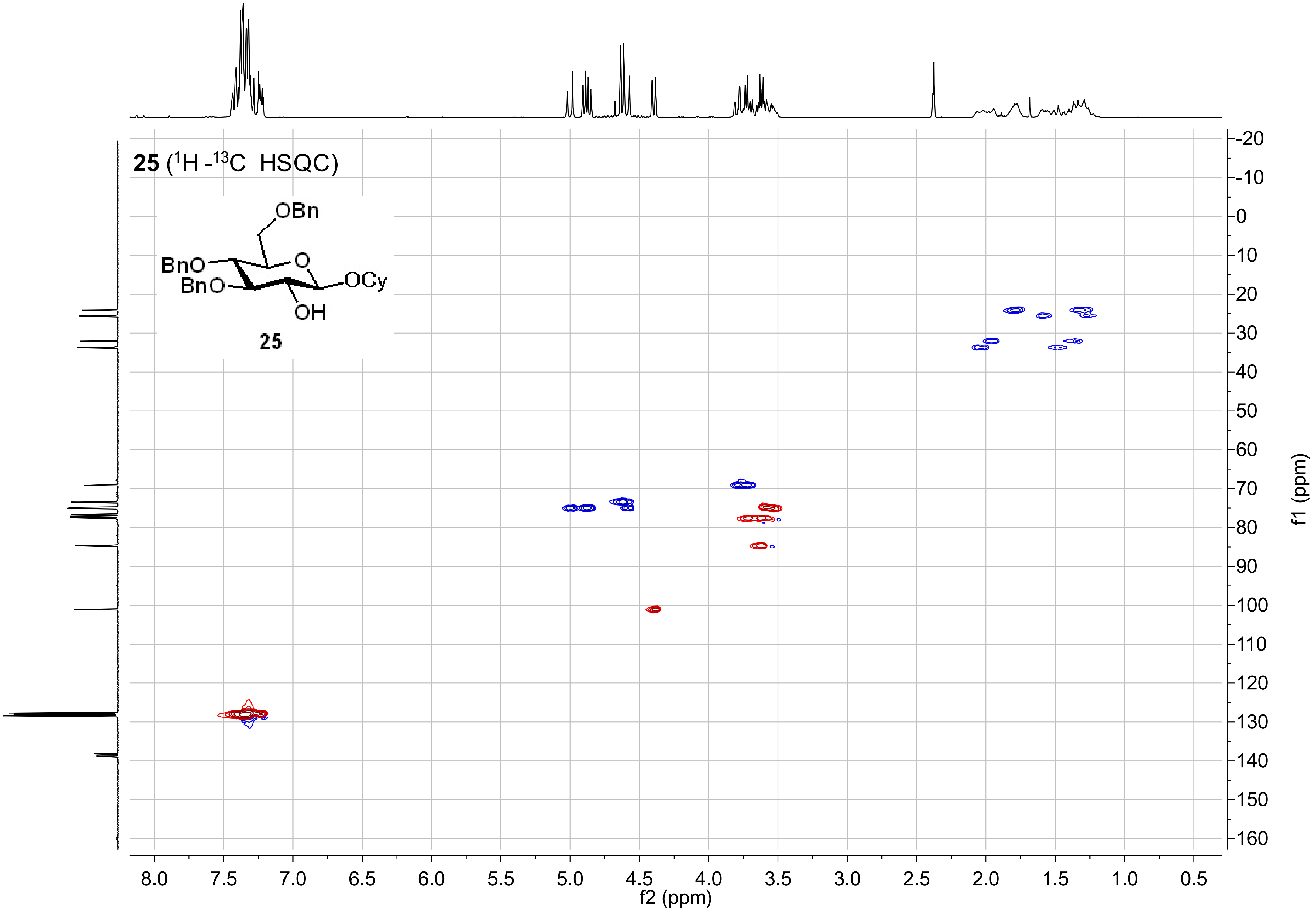

Supplementary Figure 37. ¹H-¹³C HSQC Decoupled Spectrum for Compound 25

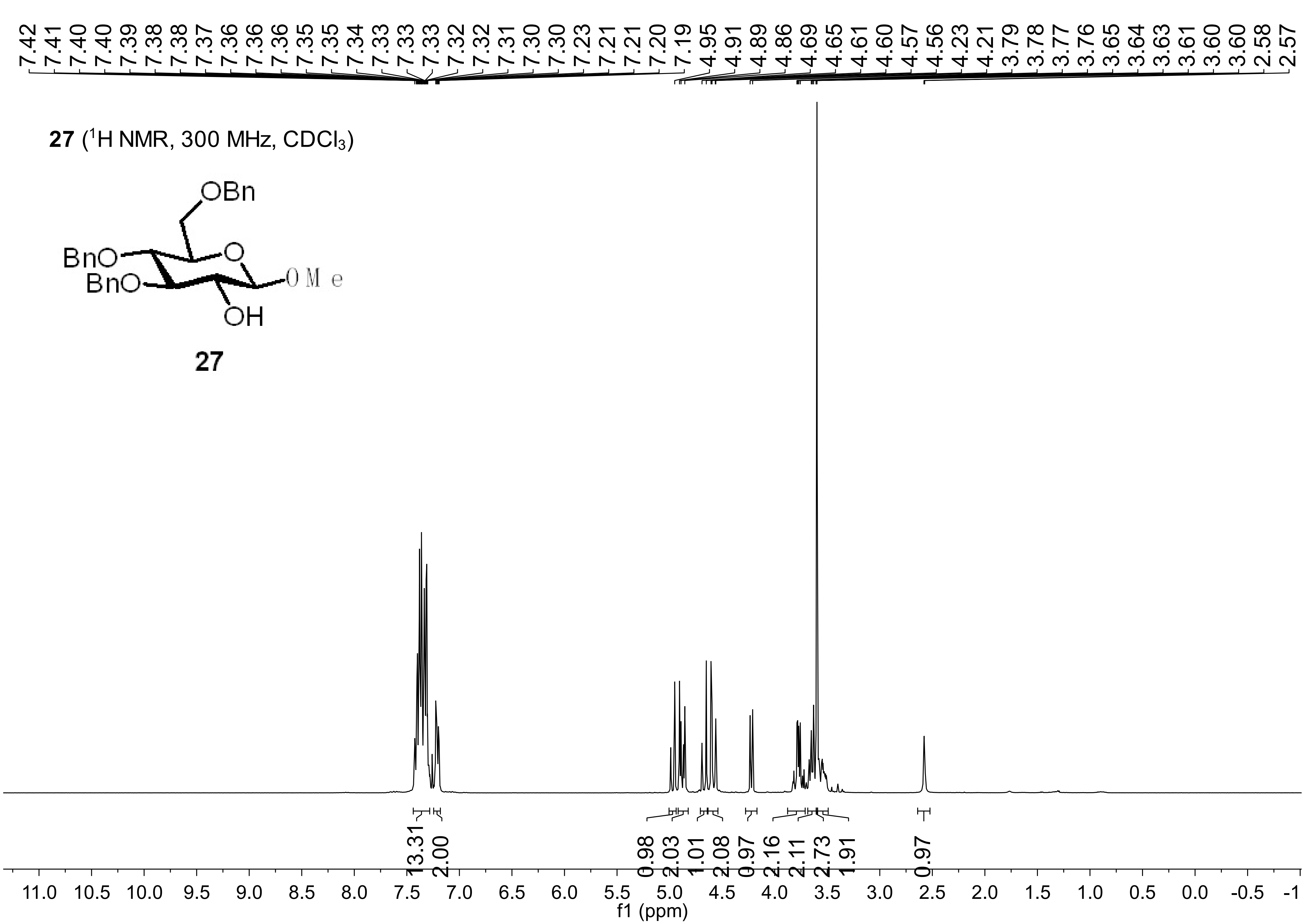

Supplementary Figure 38. ¹H NMR Spectrum for Compound 27

27 (^{13}C NMR, 75 MHz, $CDCl_3$)

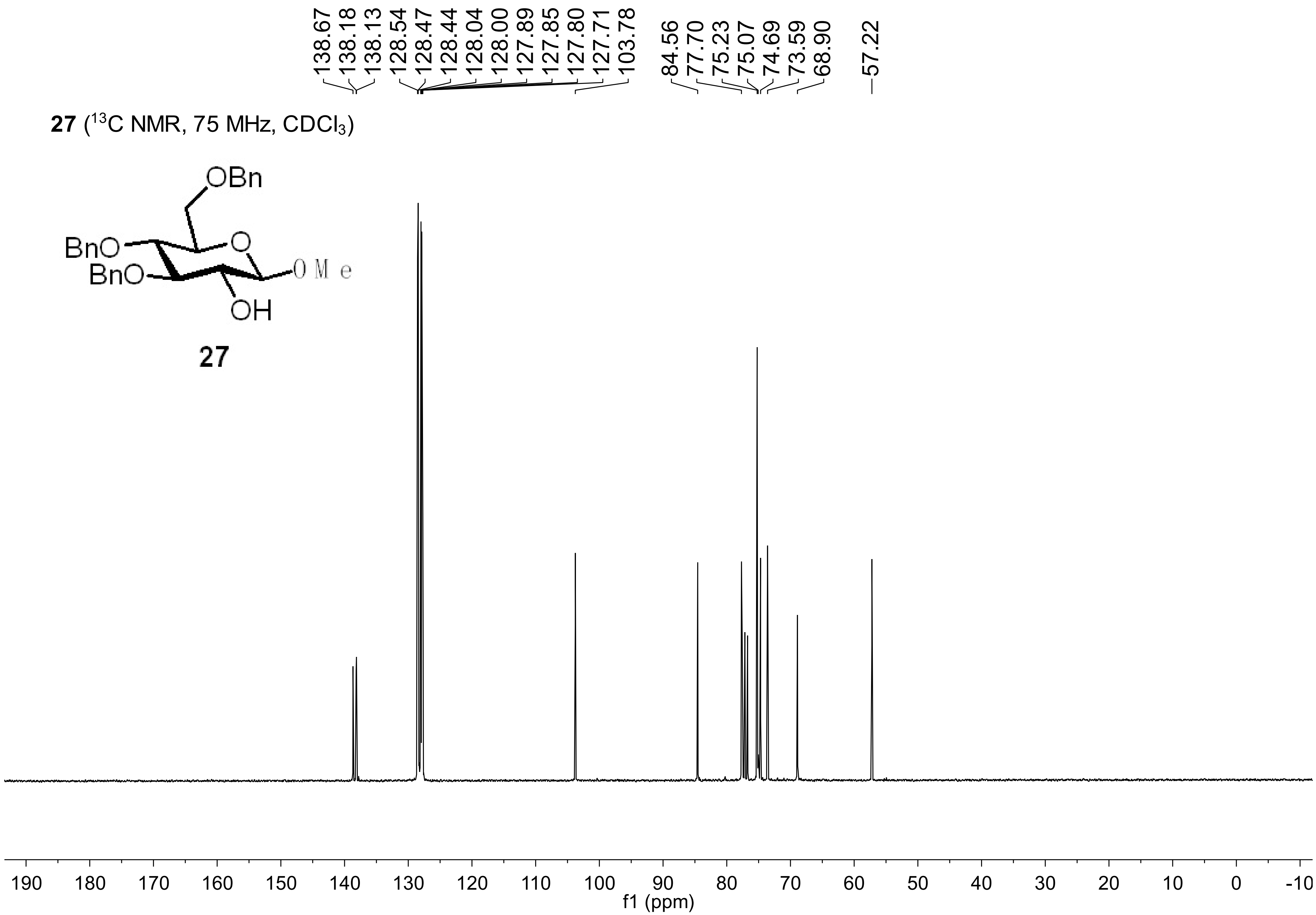

Supplementary Figure 39. ^{13}C NMR Spectrum for Compound 27

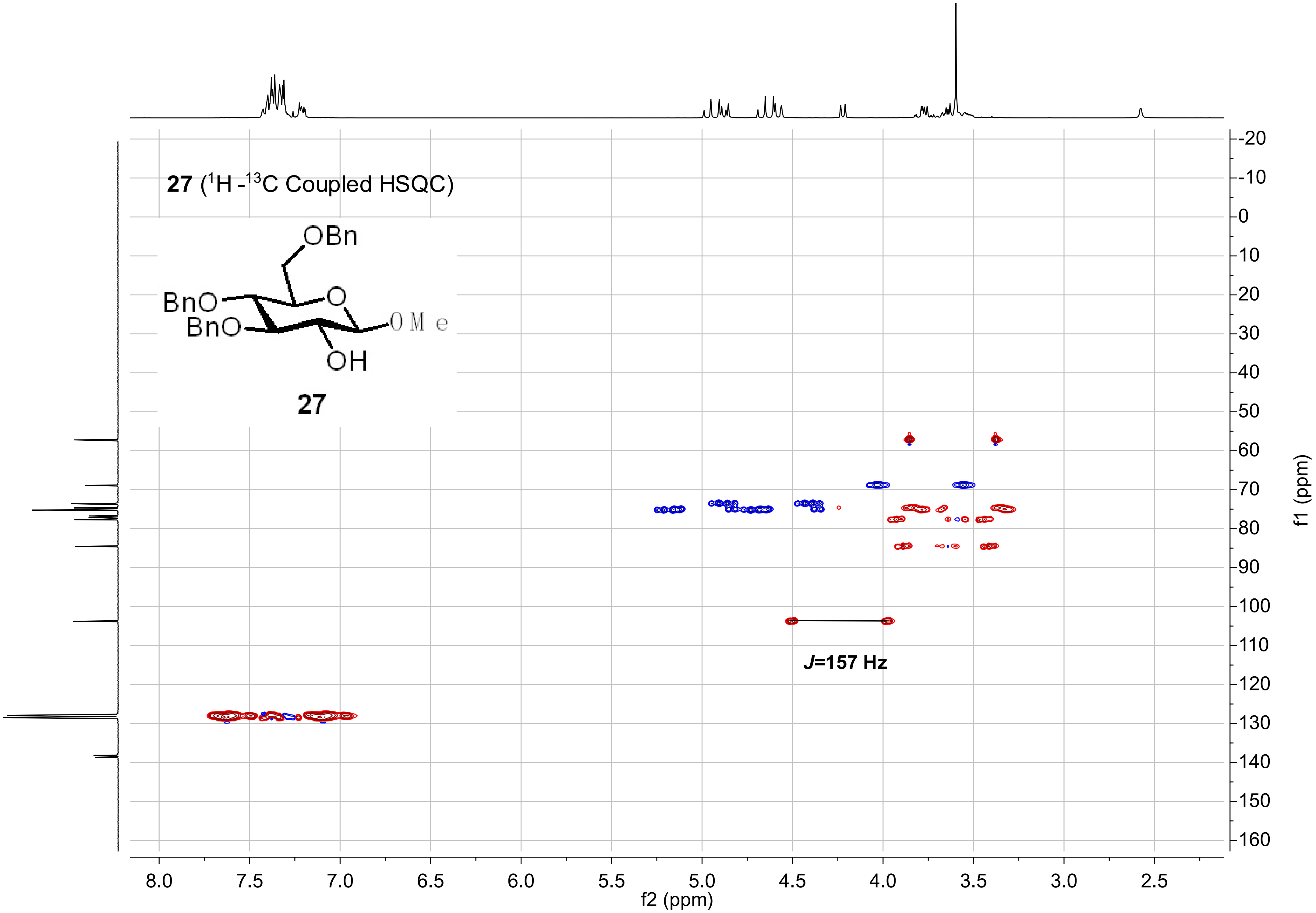

Supplementary Figure 40. ^1H-^{13}C **HSQC Coupled Spectrum for Compound 27**

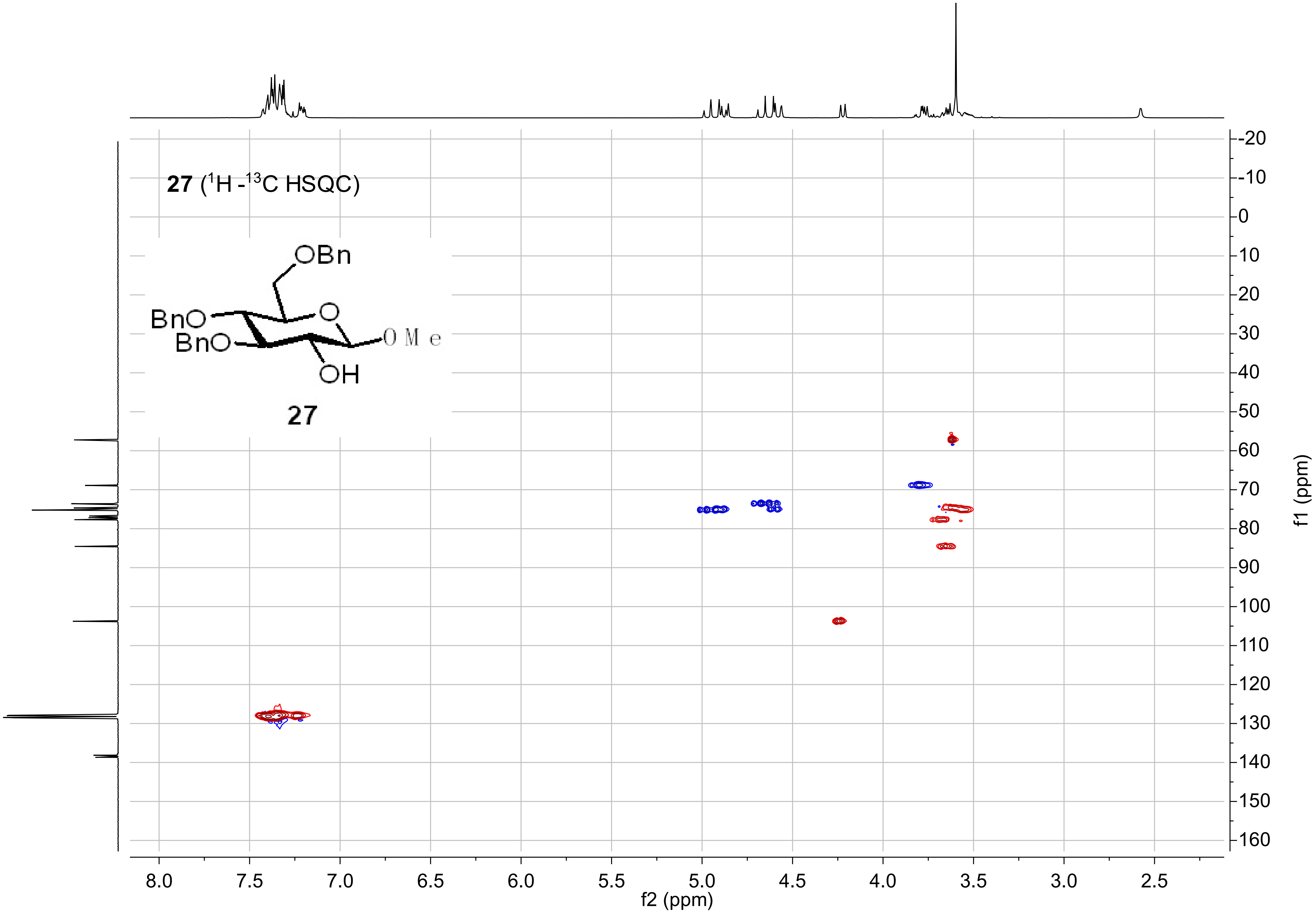

Supplementary Figure 41. ^{1}H-^{13}C HSQC Decoupled Spectrum for Compound 27

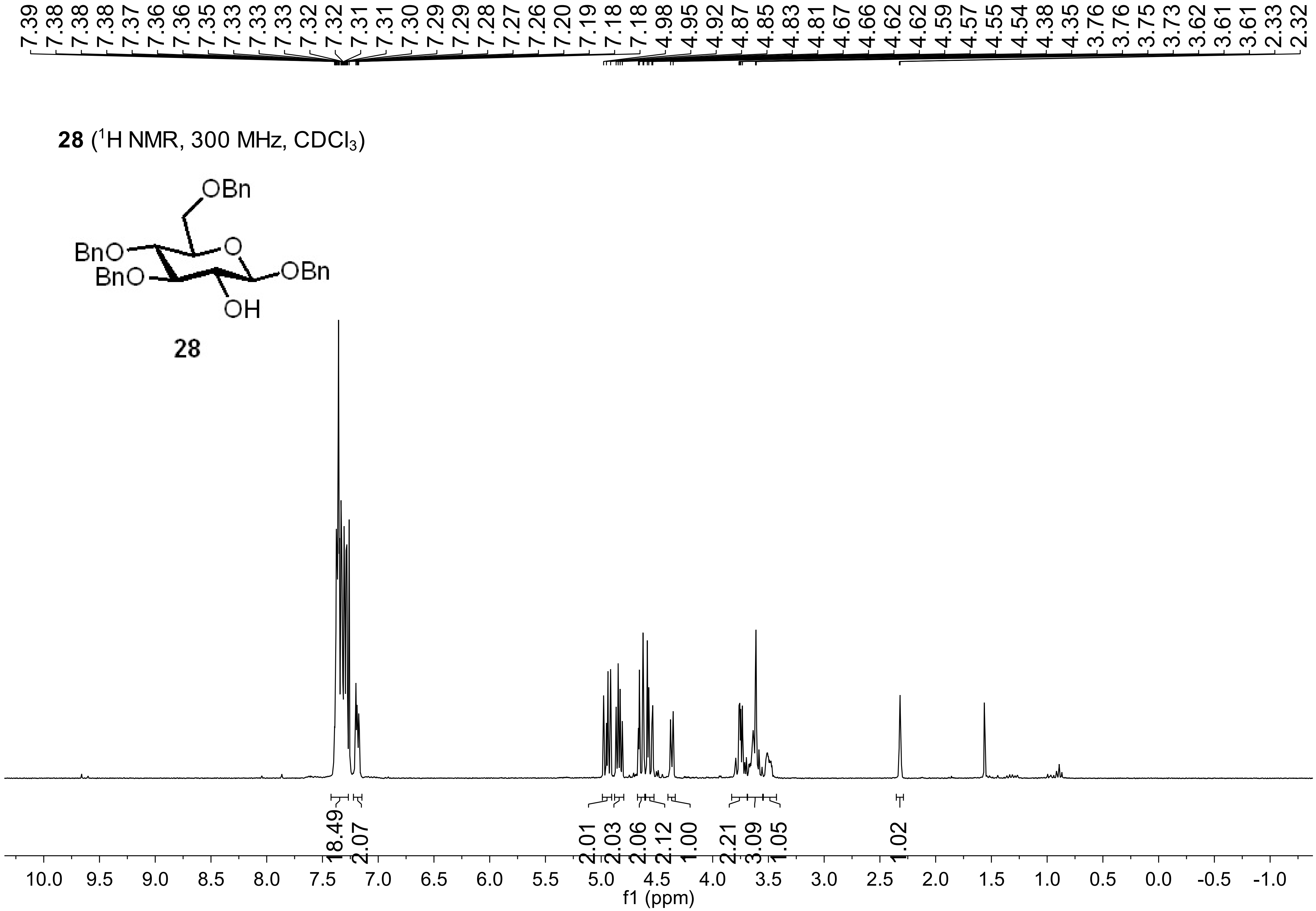

Supplementary Figure 42. ¹H NMR Spectrum for Compound 28

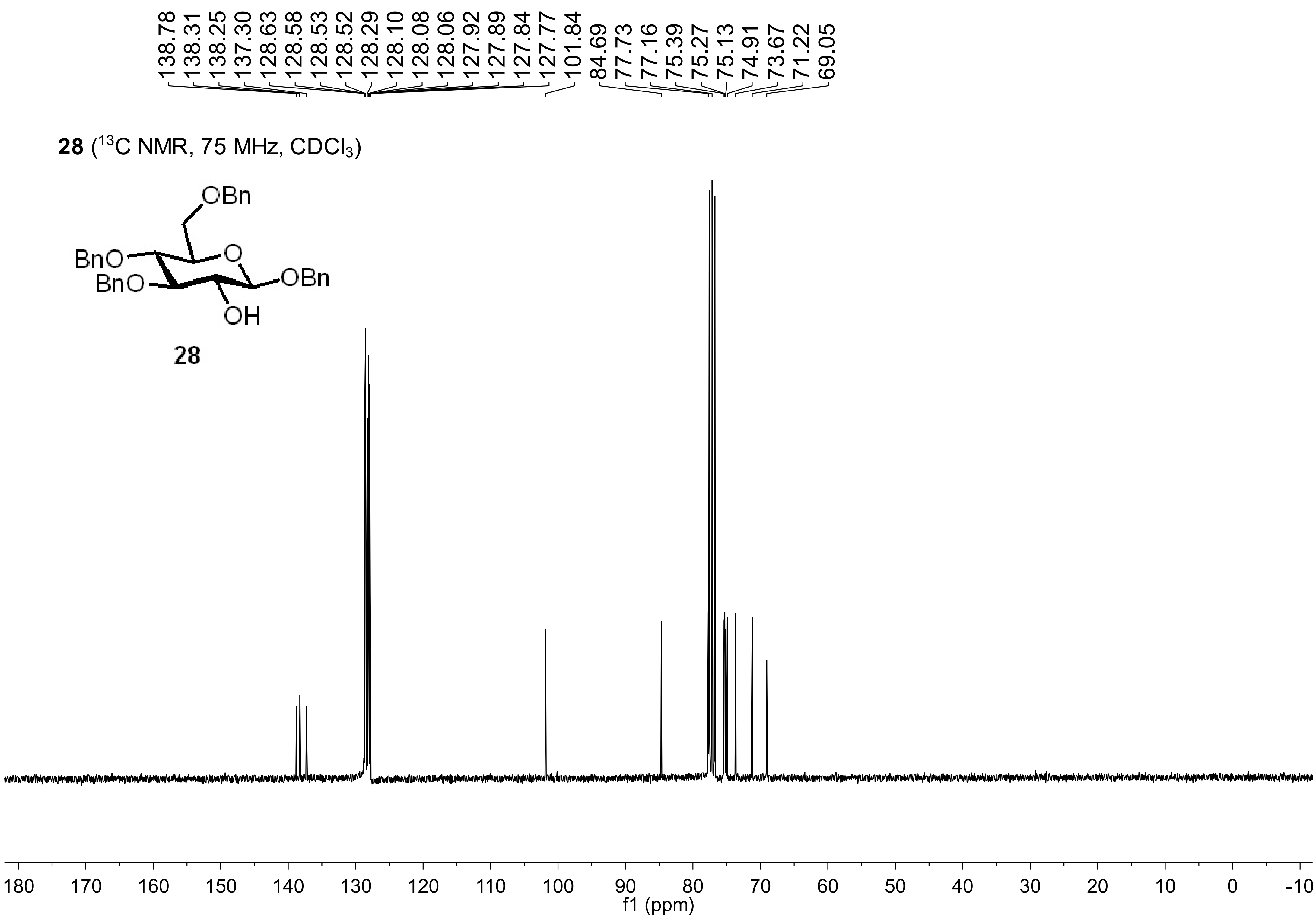

Supplementary Figure 43. ^{13}C NMR Spectrum for Compound 28

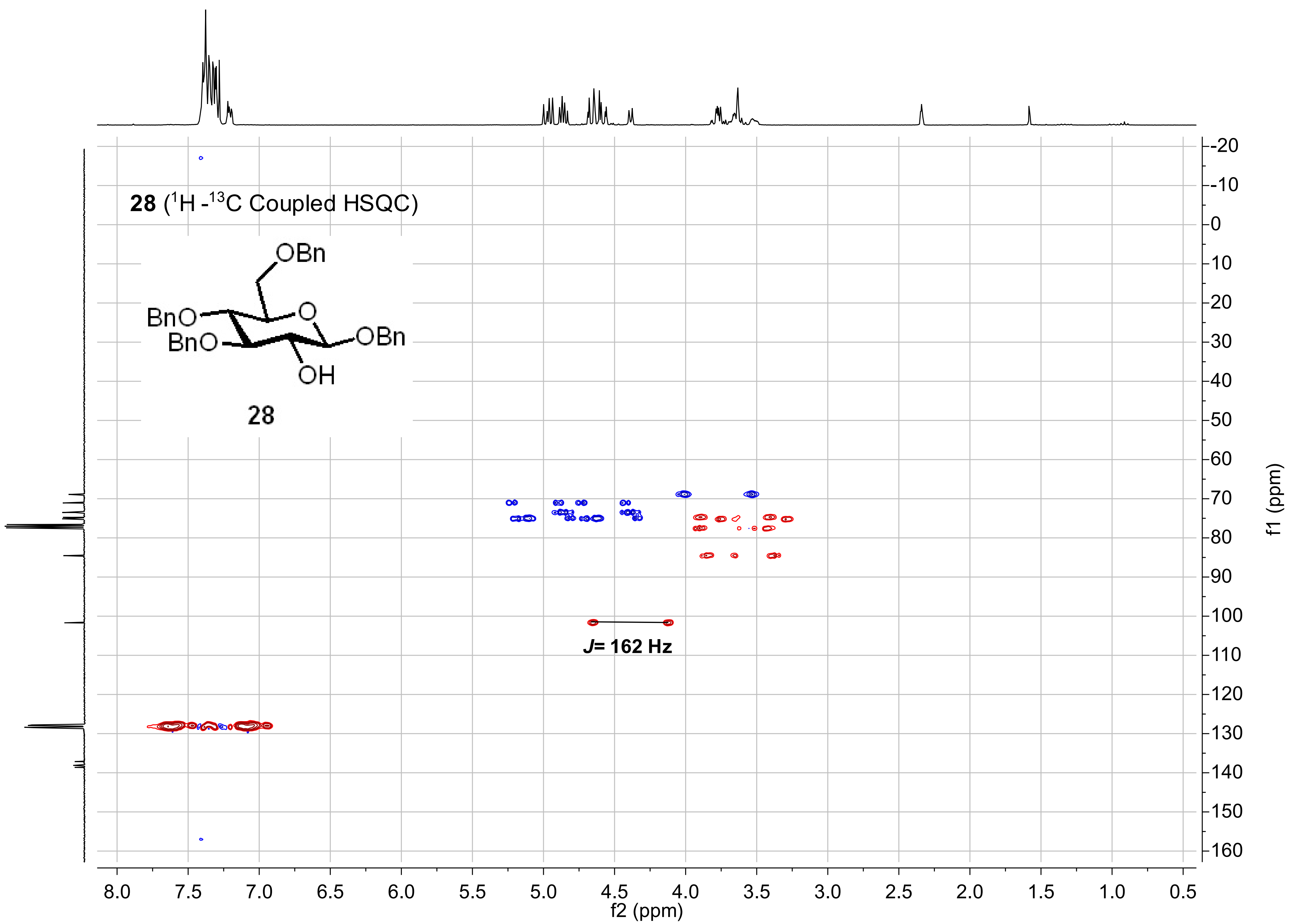

Supplementary Figure 44. ^{1}H-^{13}C HSQC Coupled Spectrum for Compound 28

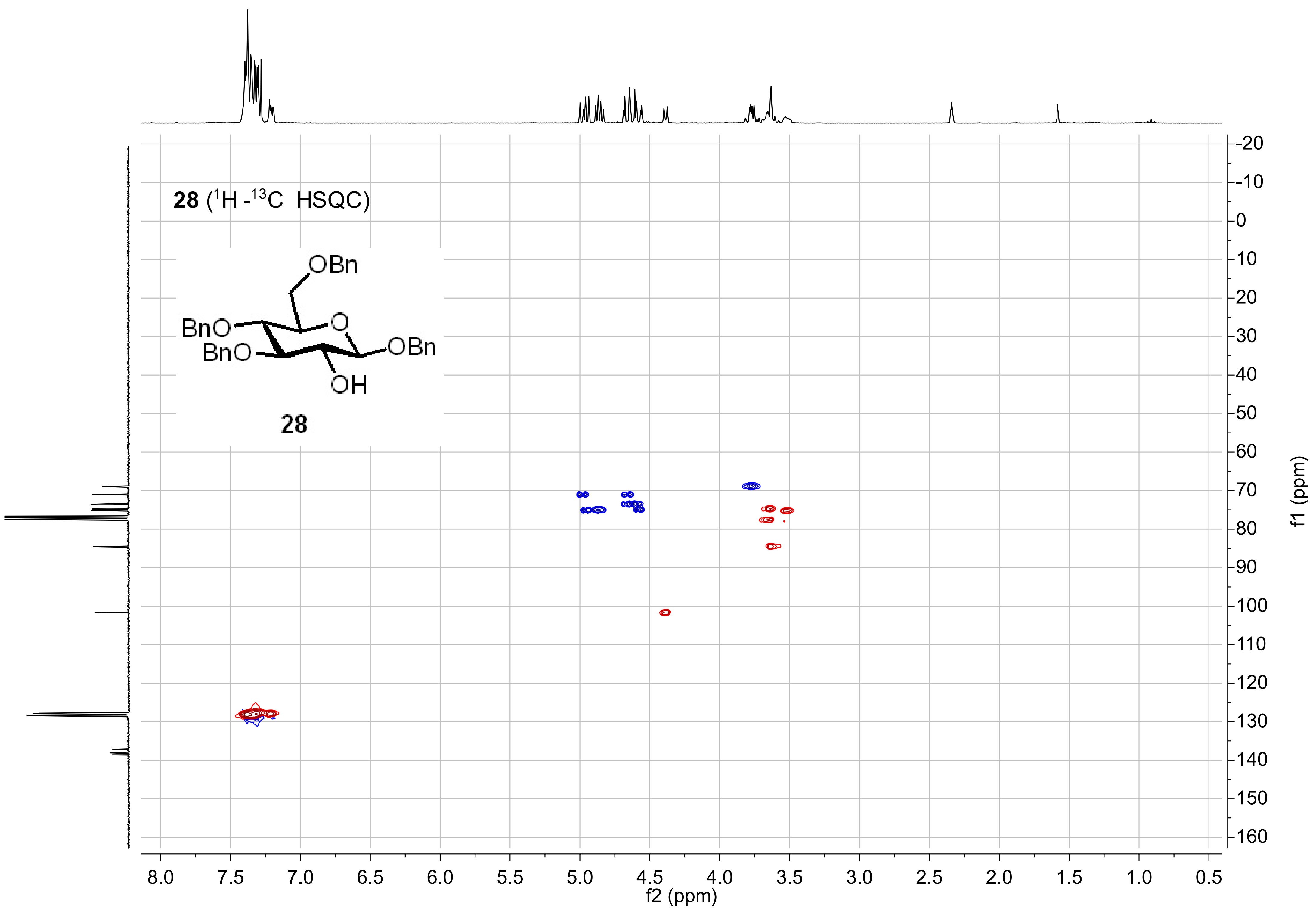

Supplementary Figure 45. ^{1}**H-**^{13}**C HSQC Decoupled Spectrum for Compound 28**

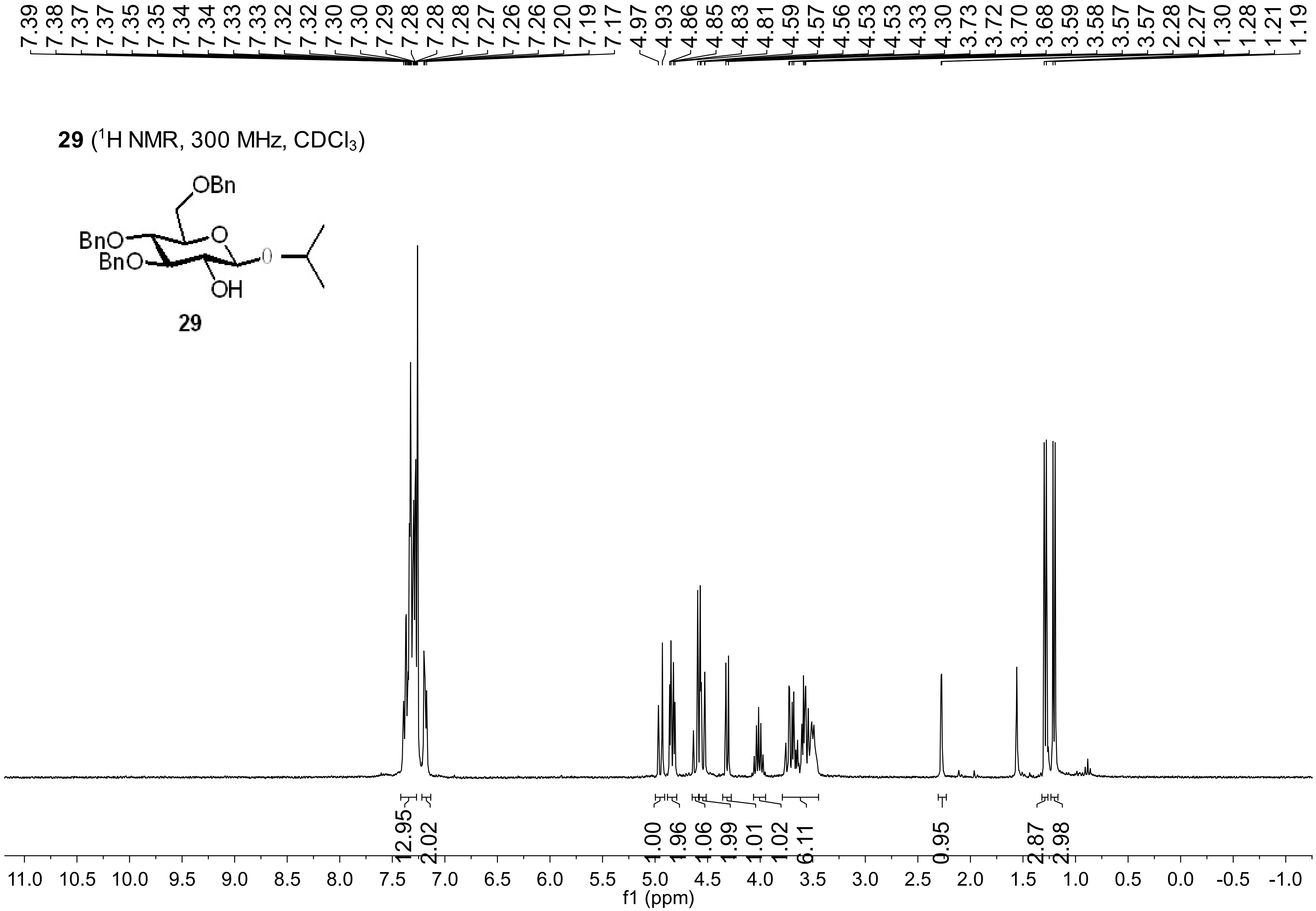

Supplementary Figure 46. **^{1}H NMR Spectrum for Compound 29**

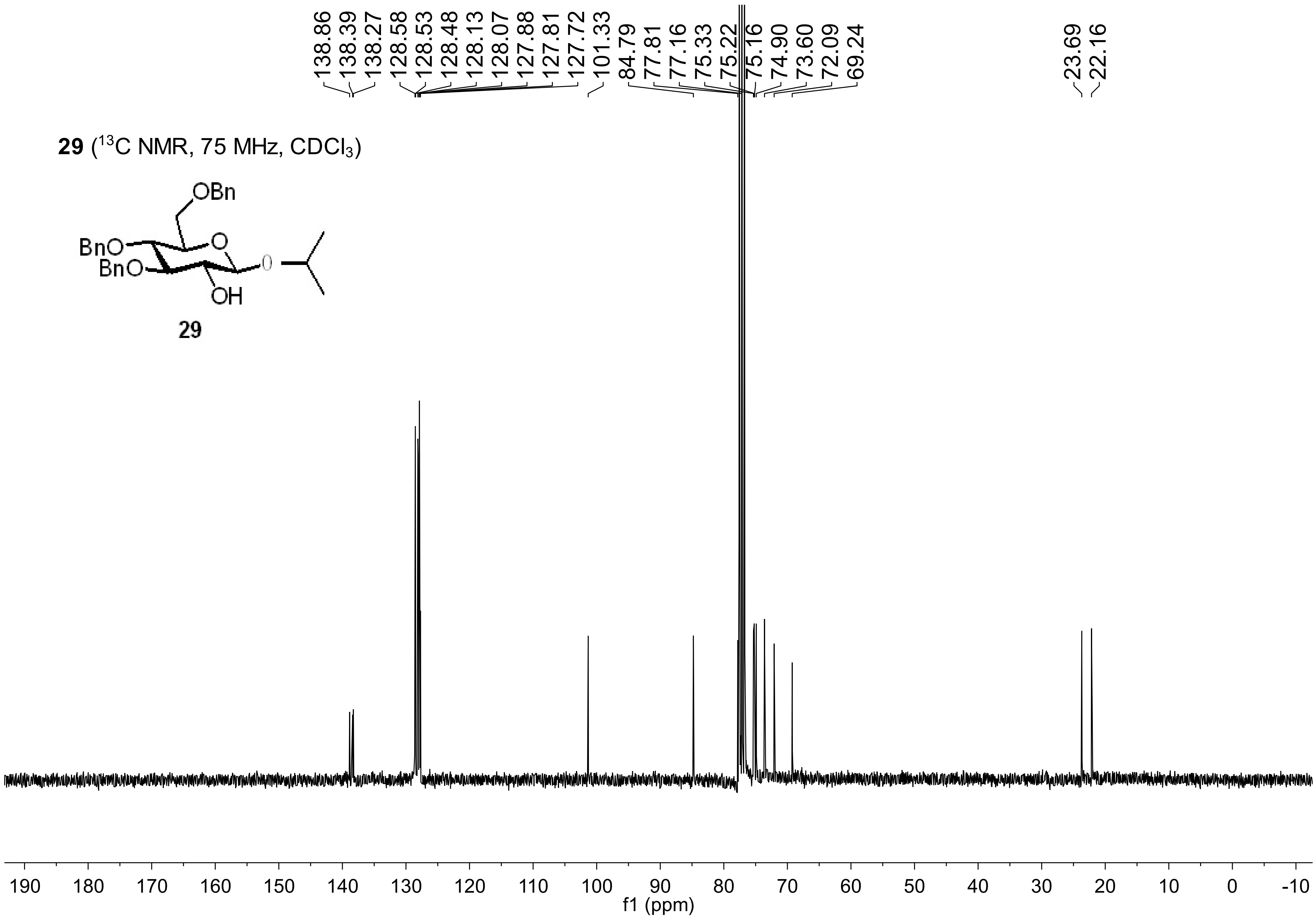

Supplementary Figure 47. **^{13}C NMR Spectrum for Compound 29**

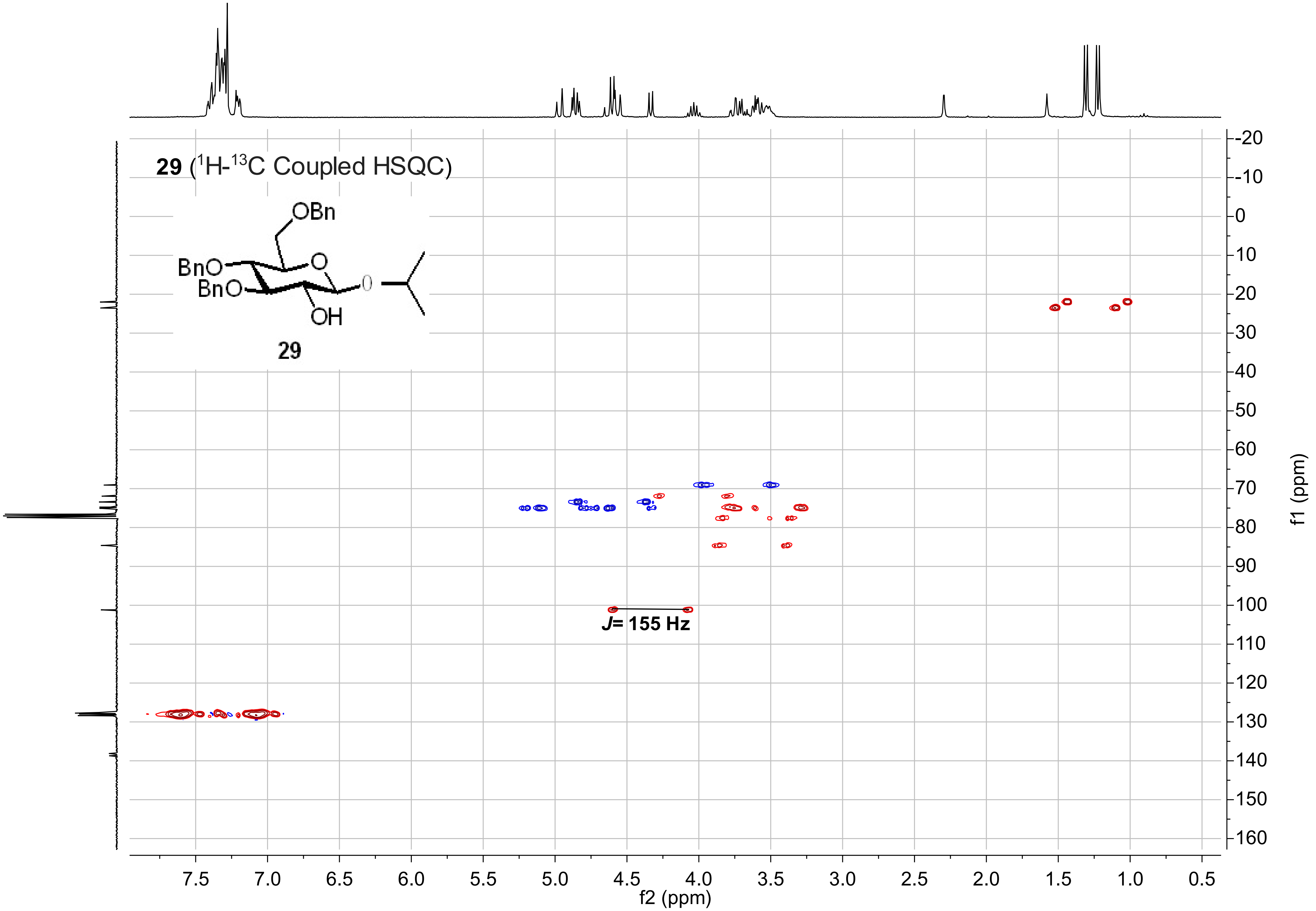

Supplementary Figure 48. **^{1}H-^{13}C HSQC Coupled Spectrum for Compound 25**

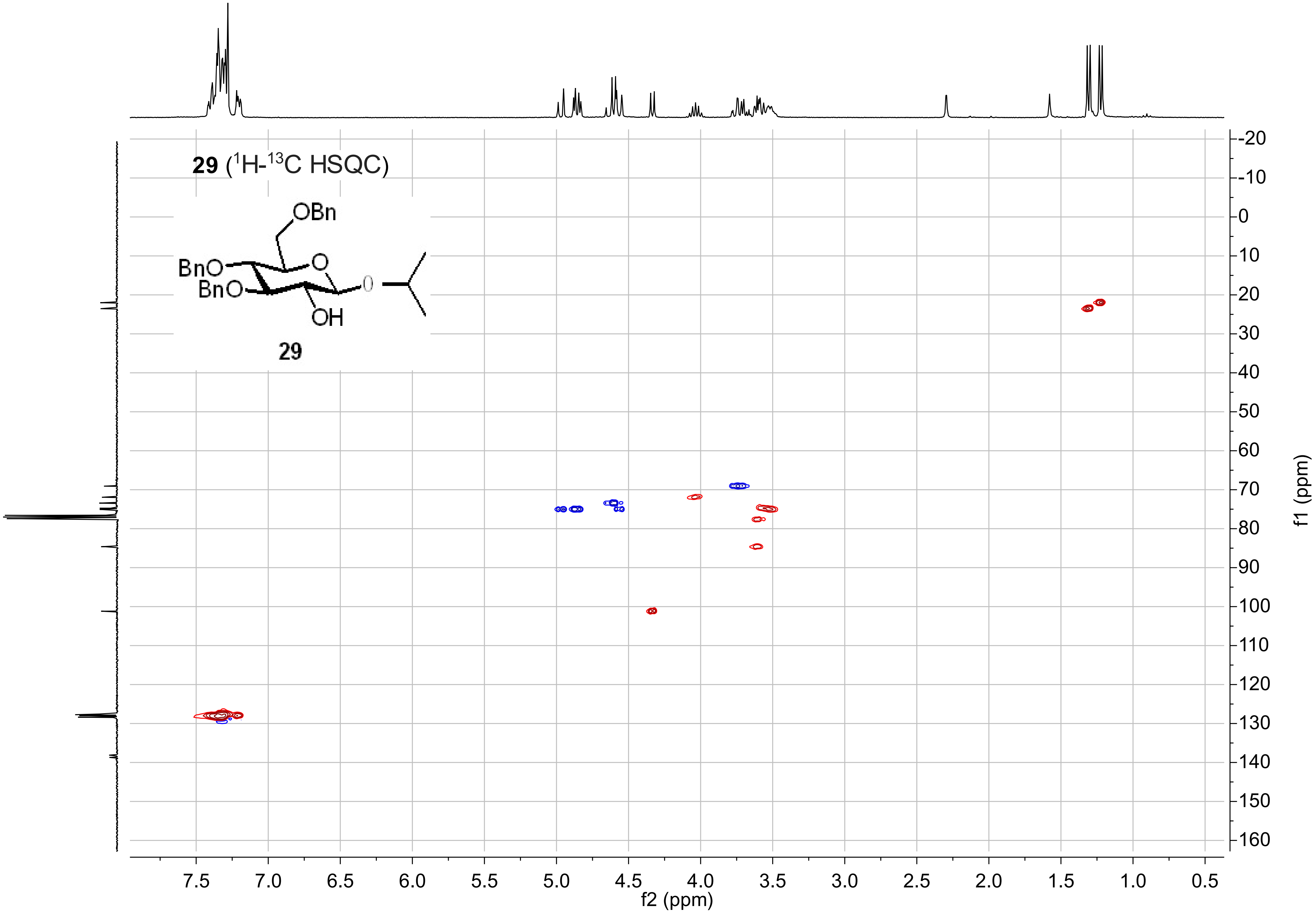

Supplementary Figure 49. ^{1}H-^{13}C HSQC Decoupled Spectrum for Compound 29

30 (^{1}H NMR, 300 MHz, $CDCl_3$)

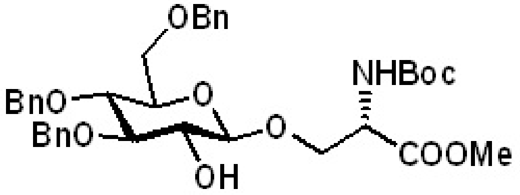

30

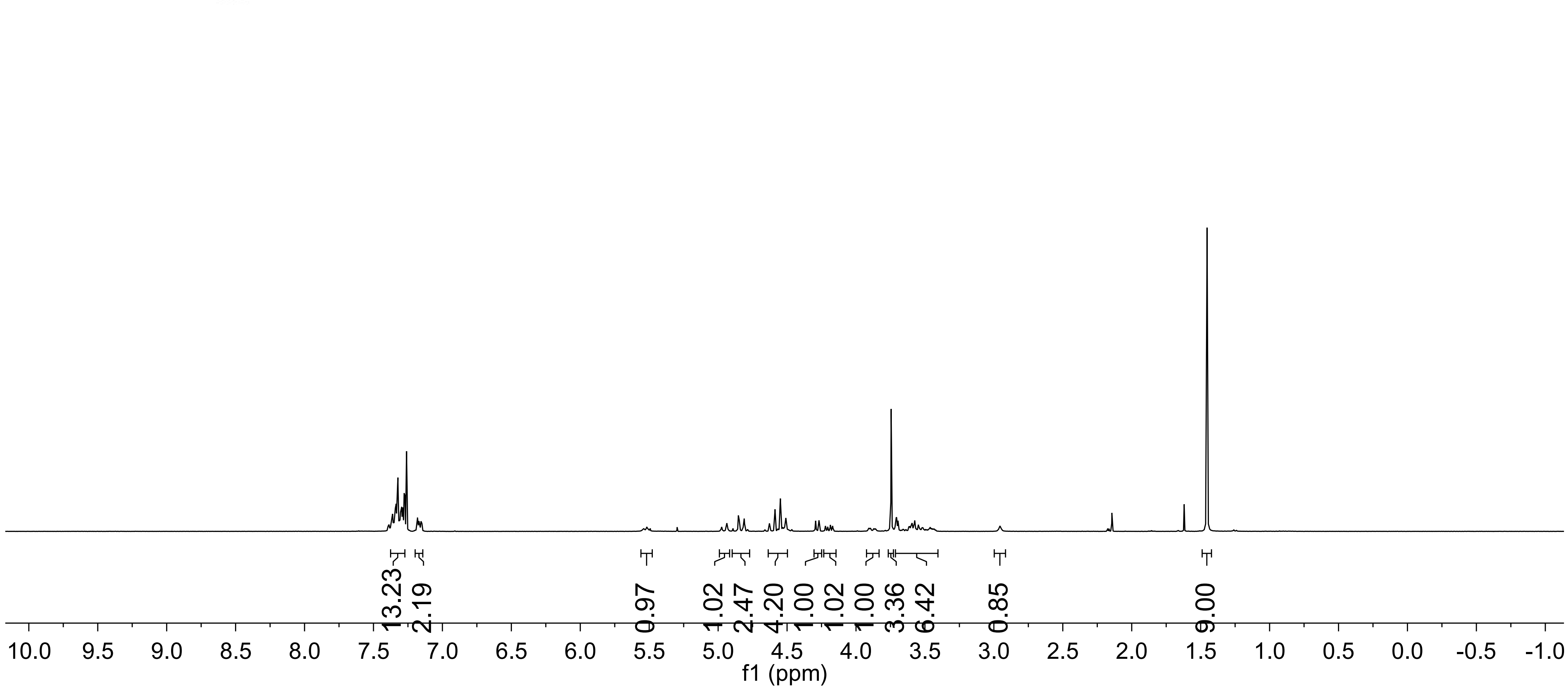

Supplementary Figure 50. ^{1}H NMR Spectrum for Compound 30

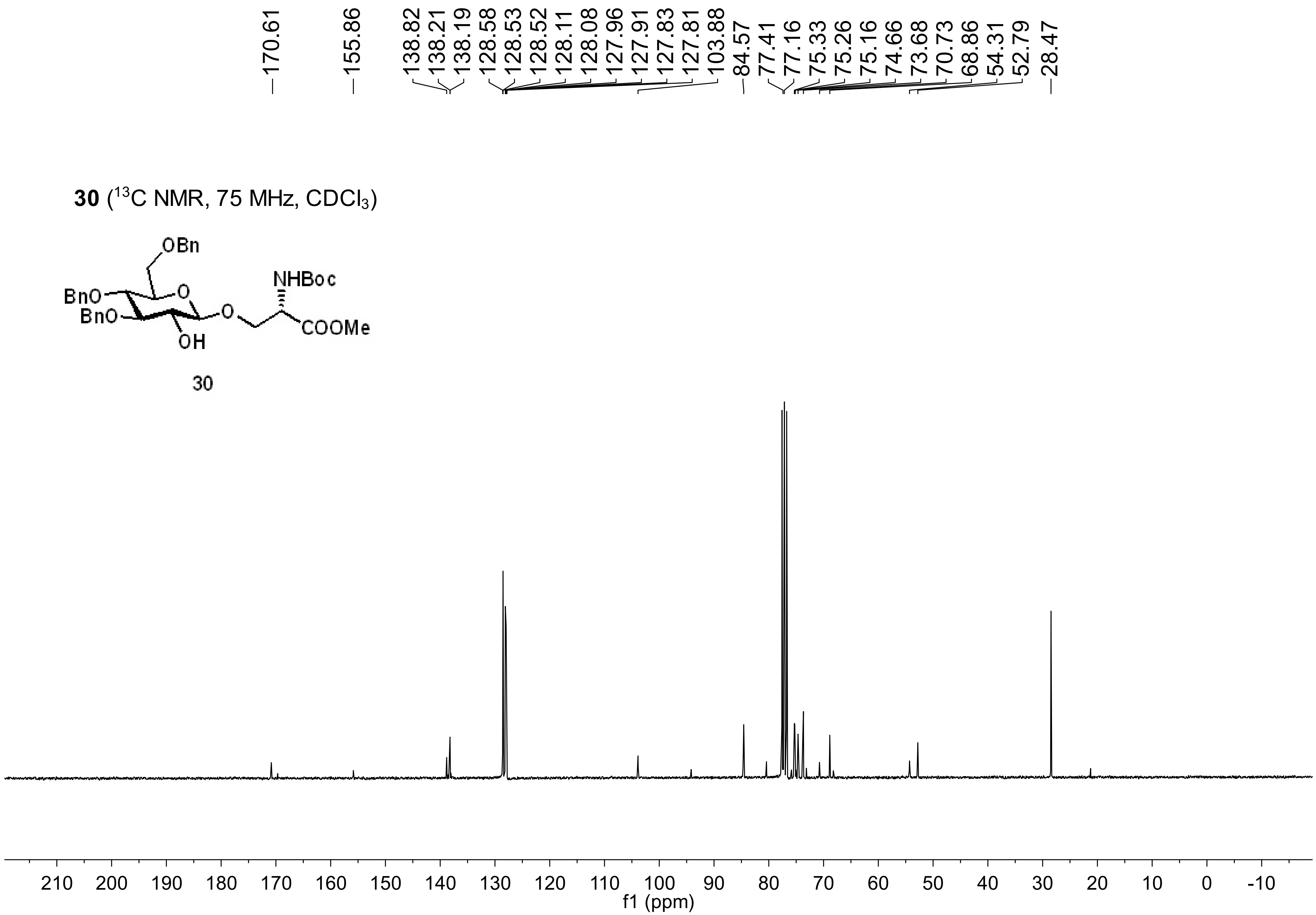

Supplementary Figure 51. ^{13}C NMR Spectrum for Compound 30

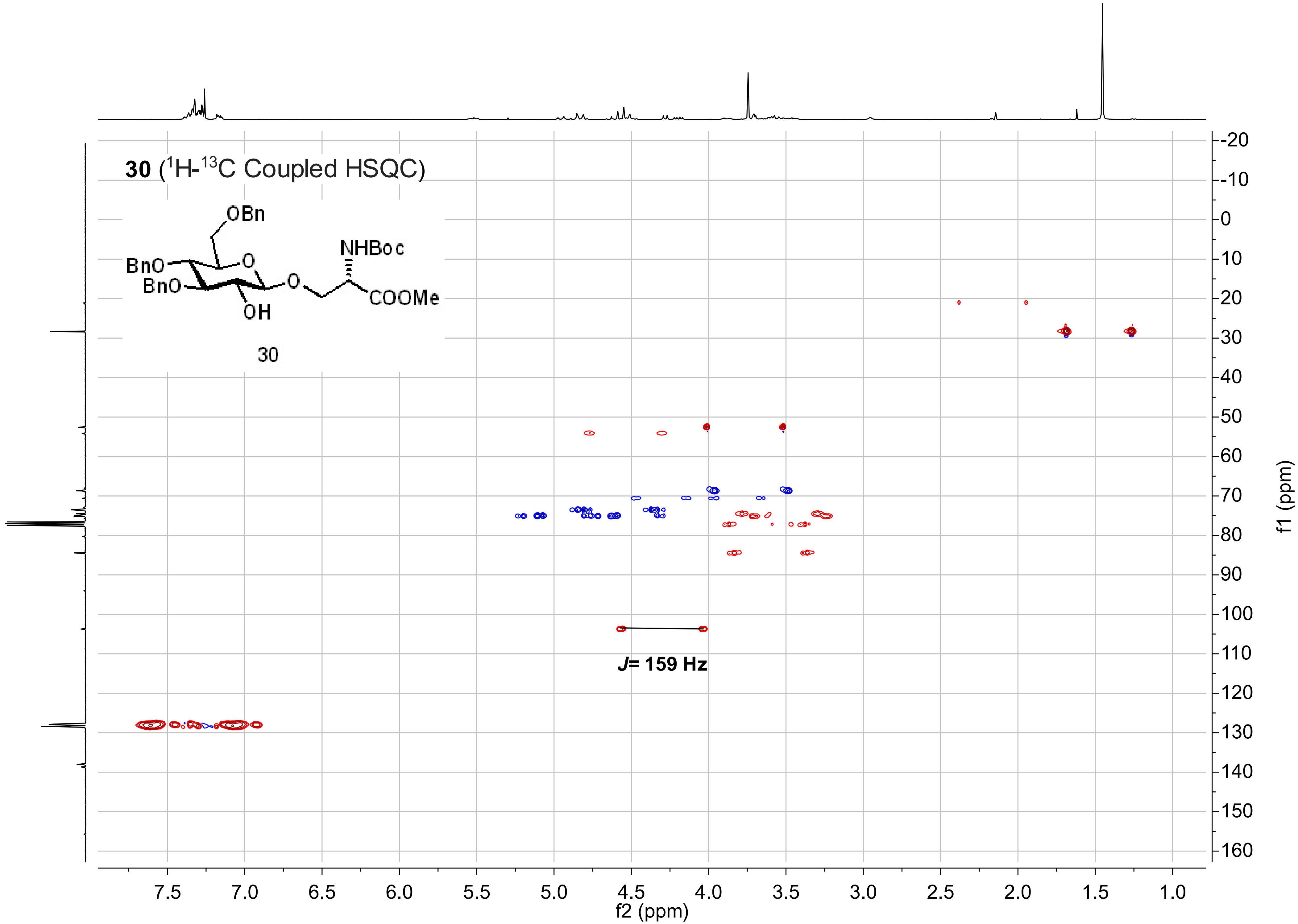

Supplementary Figure 52. ^{1}H-^{13}C **HSQC Coupled Spectrum for Compound 30**

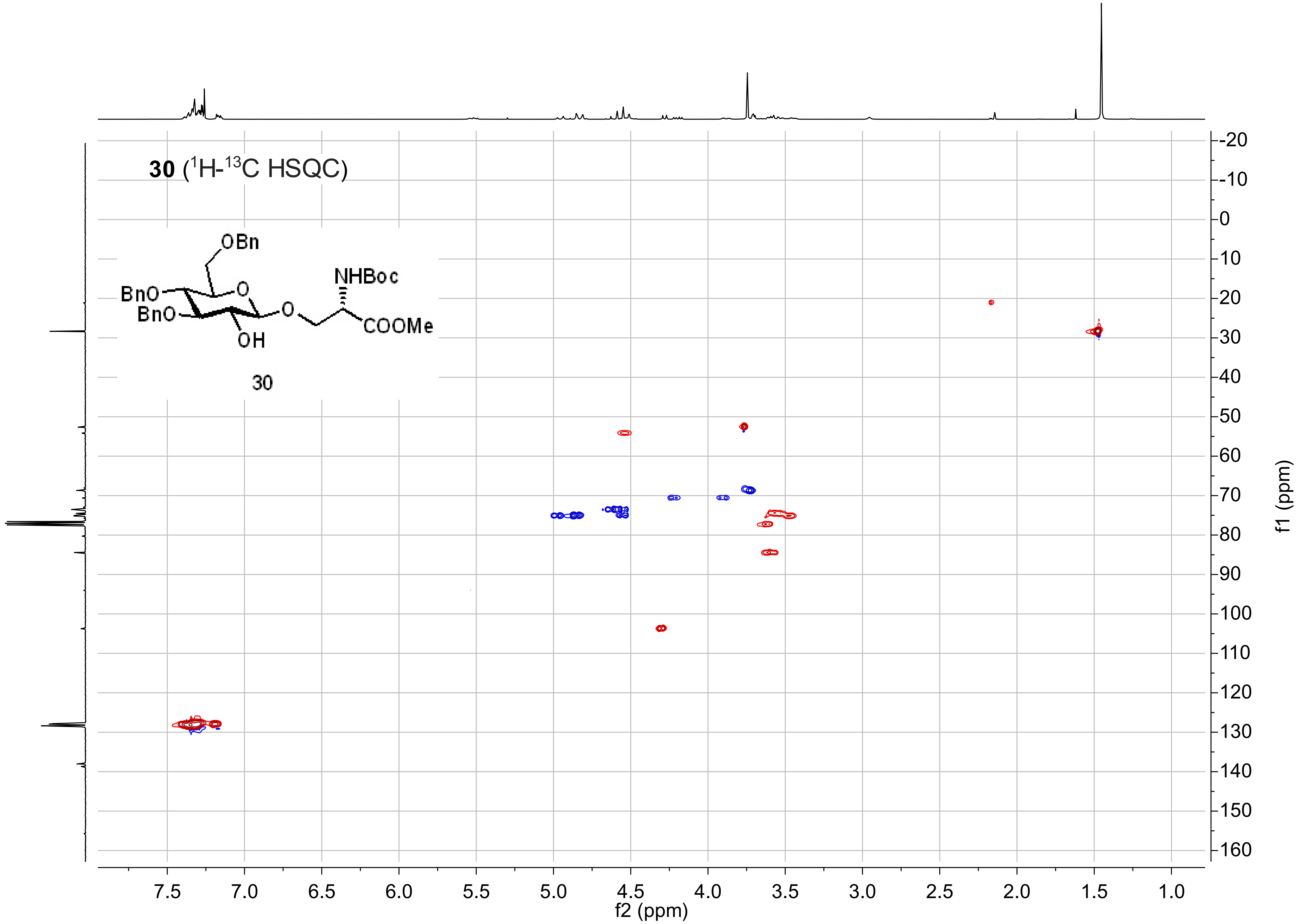

Supplementary Figure 53. **^{1}H-^{13}C HSQC Decoupled Spectrum for Compound 30**

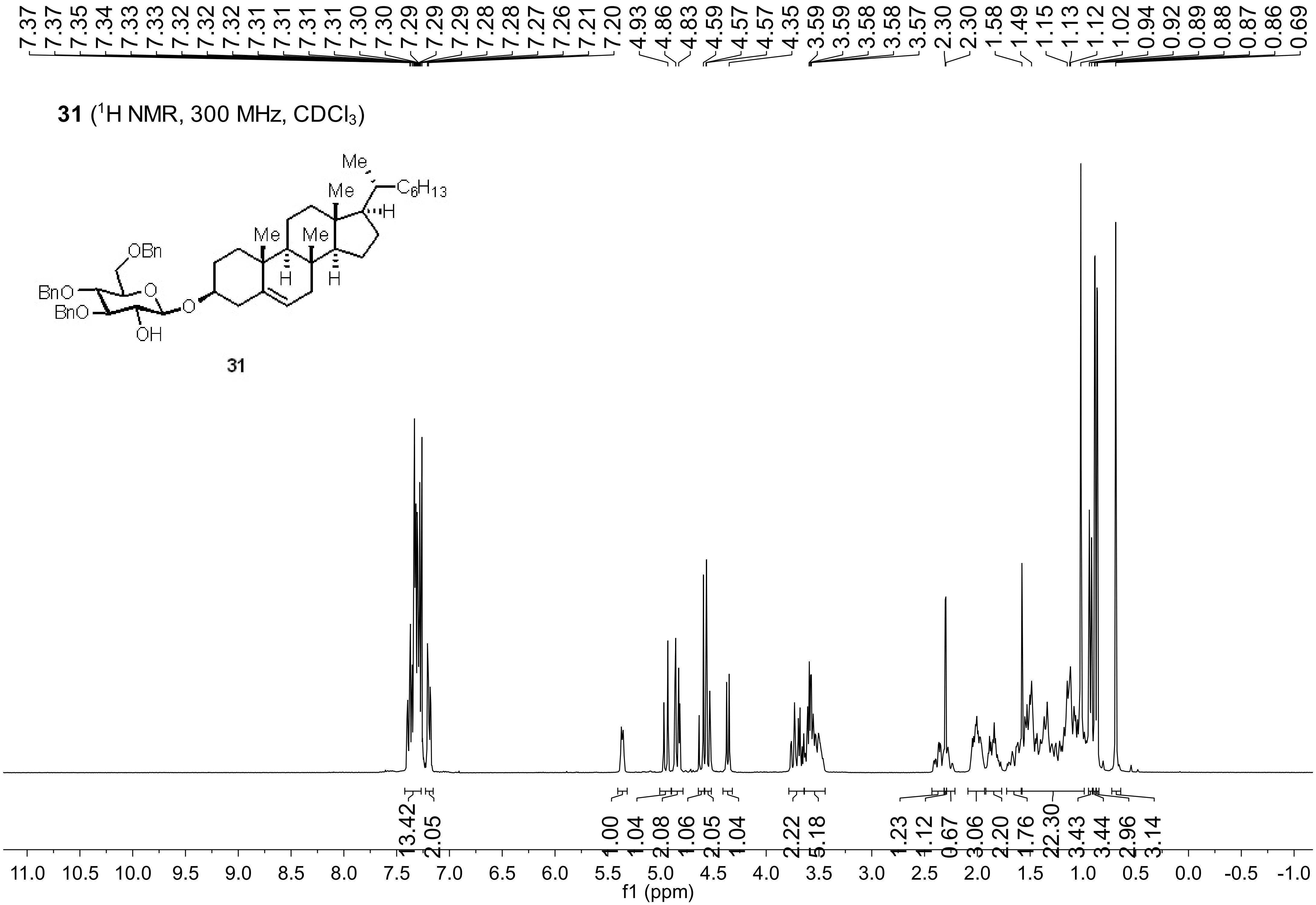

Supplementary Figure 54. ¹H NMR Spectrum for Compound 31

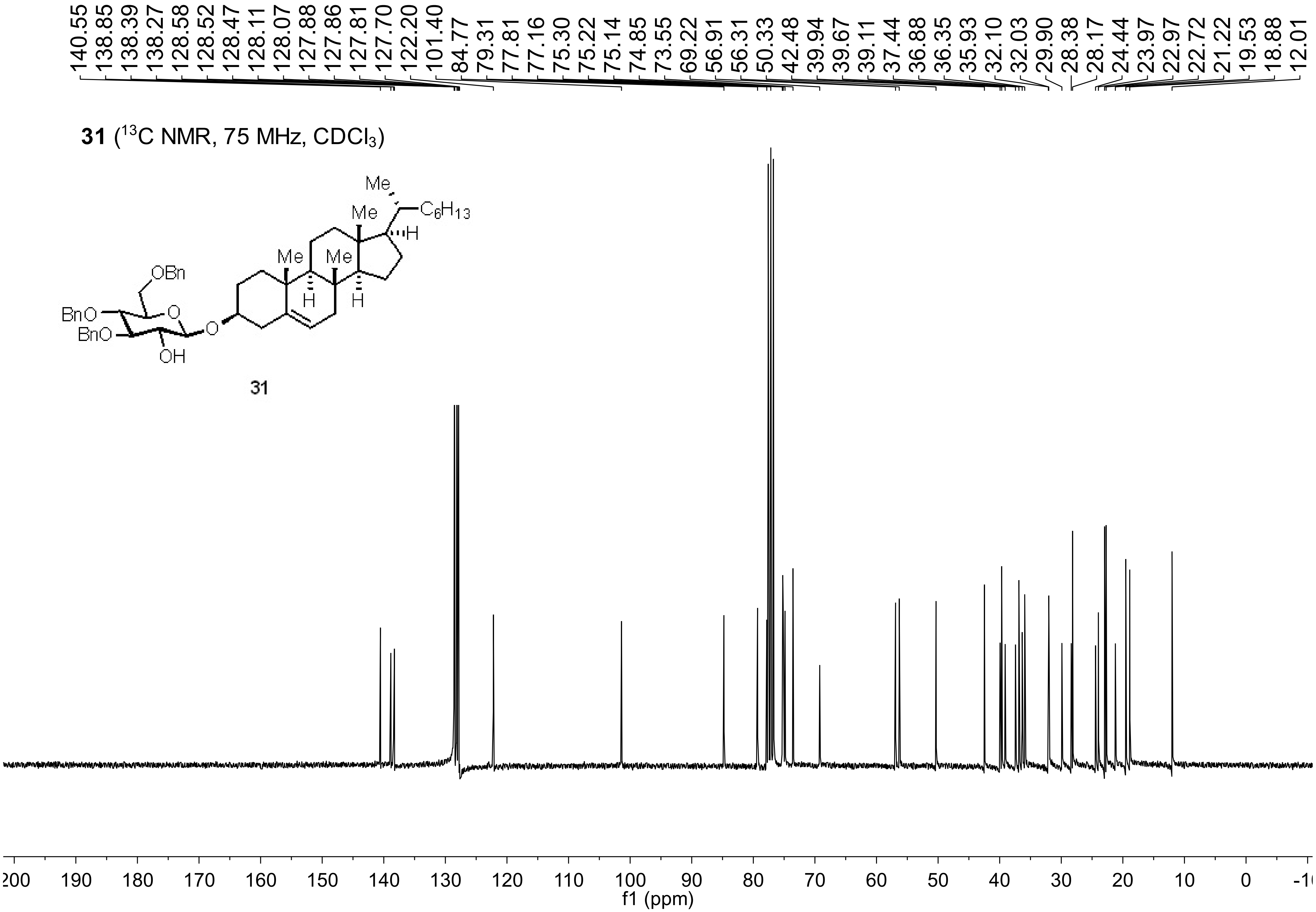

Supplementary Figure 55. ^{13}**C NMR Spectrum for Compound 31**

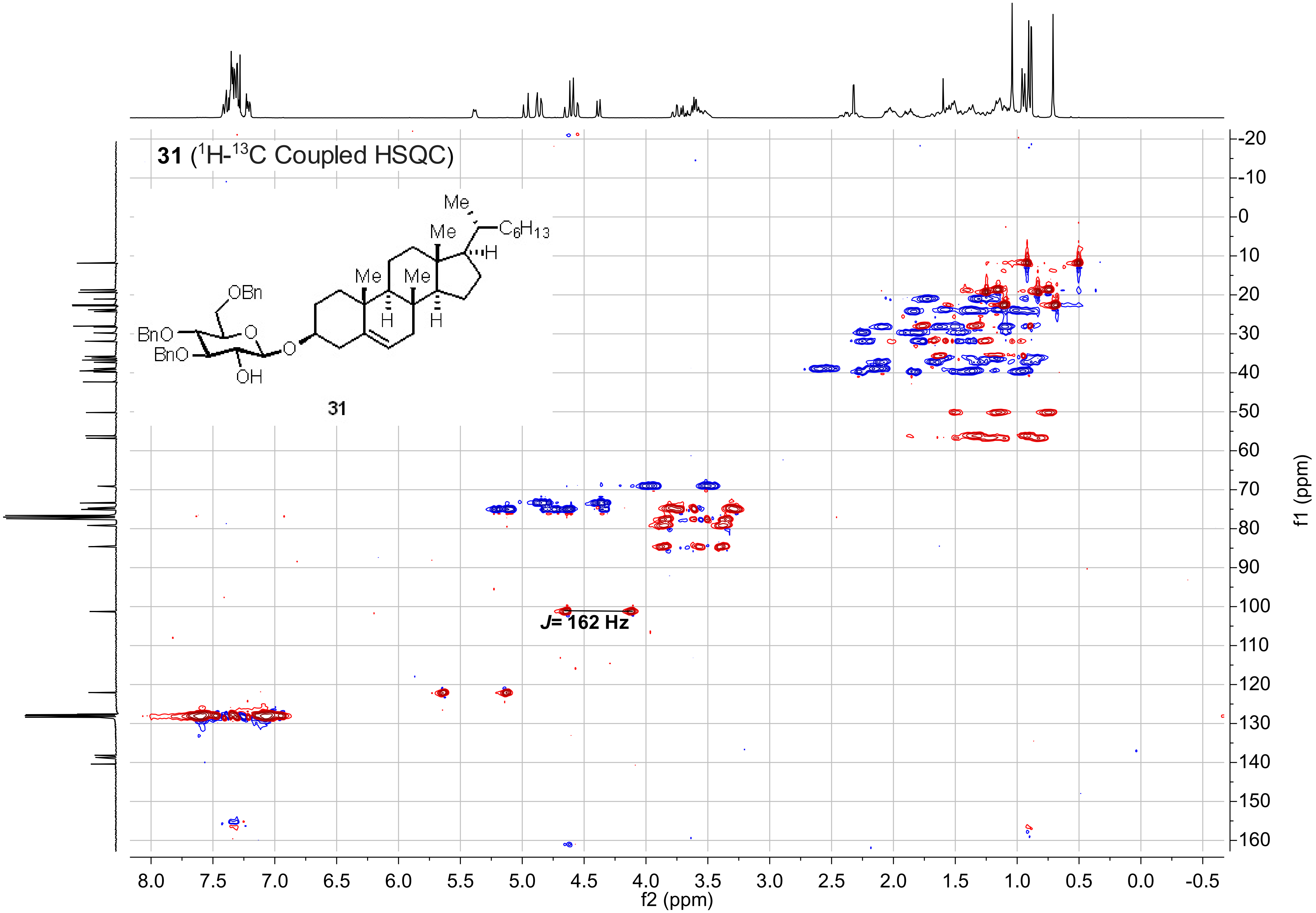

Supplementary Figure 56. ^{1}H-^{13}C **HSQC Coupled Spectrum for Compound 31**

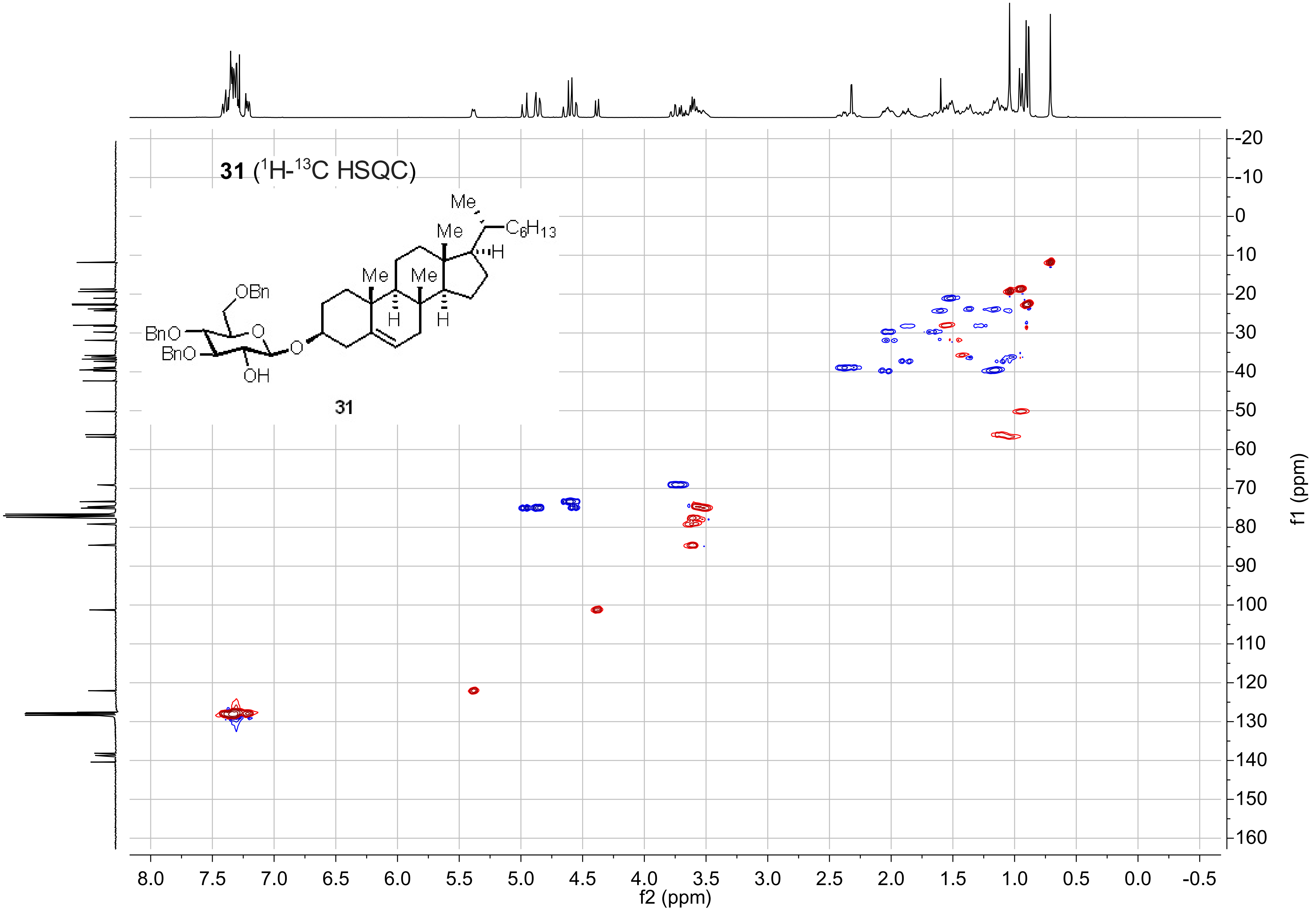

Supplementary Figure 57. ^{1}H-^{13}C HSQC Decoupled Spectrum for Compound 31

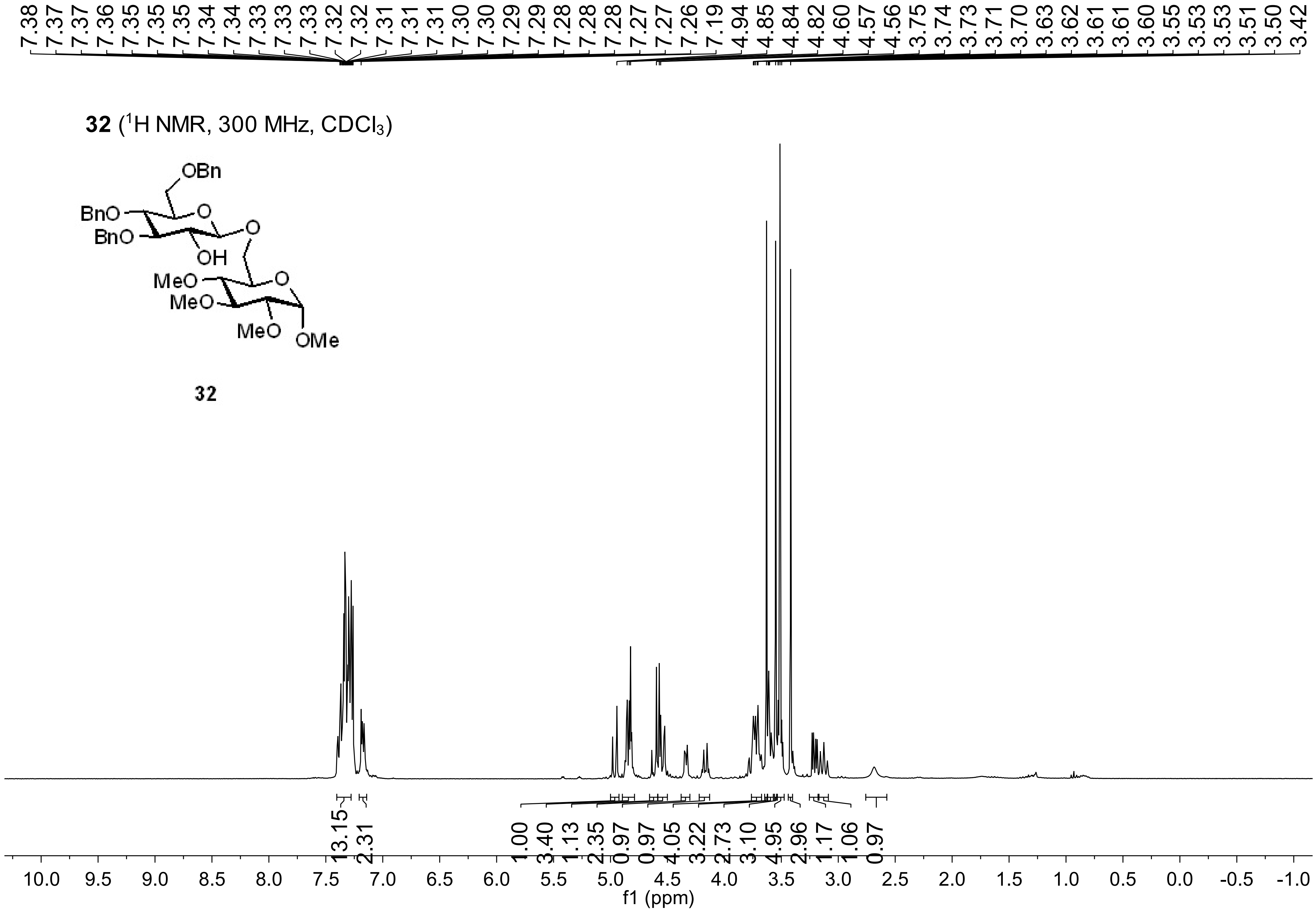

Supplementary Figure 58. ^{1}H NMR Spectrum for Compound 32

32 (^{13}C NMR, 75 MHz, $CDCl_3$)

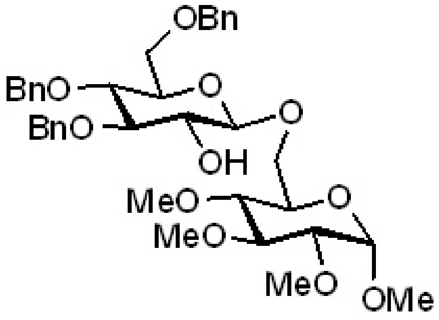

32

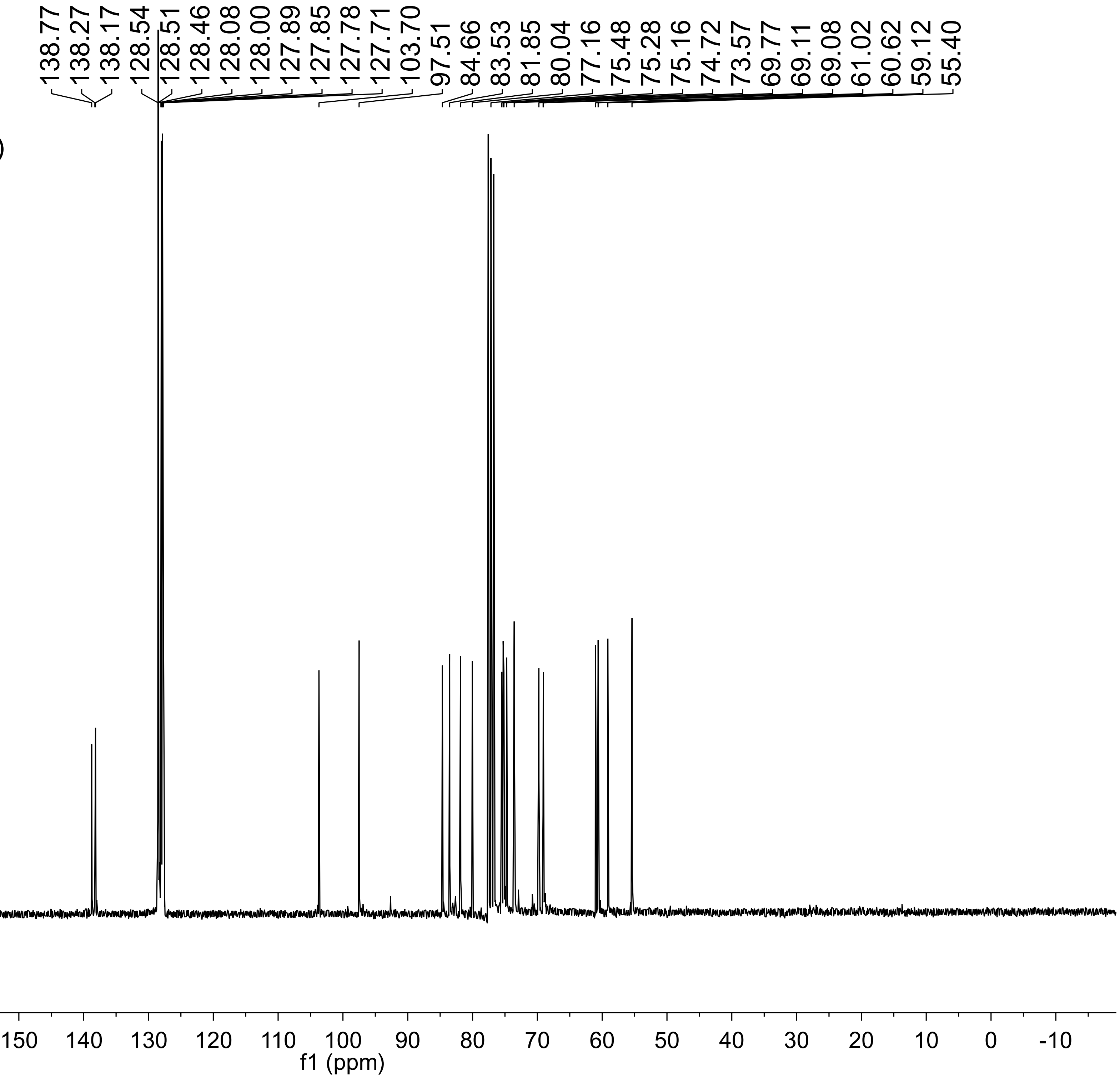

Supplementary Figure 59. ^{13}C NMR Spectrum for Compound 32

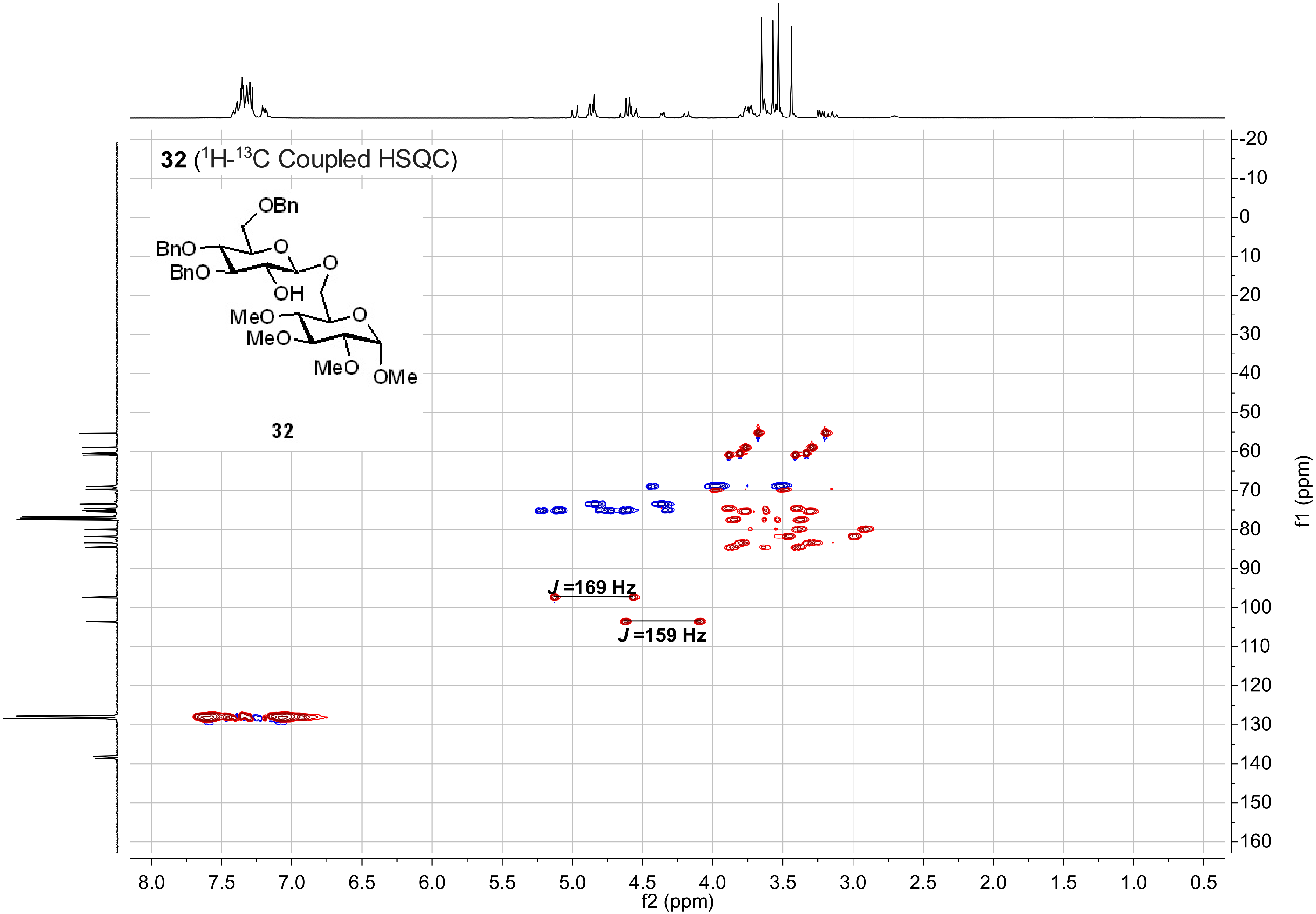

Supplementary Figure 60. ^{1}H-^{13}C HSQC Coupled Spectrum for Compound 32

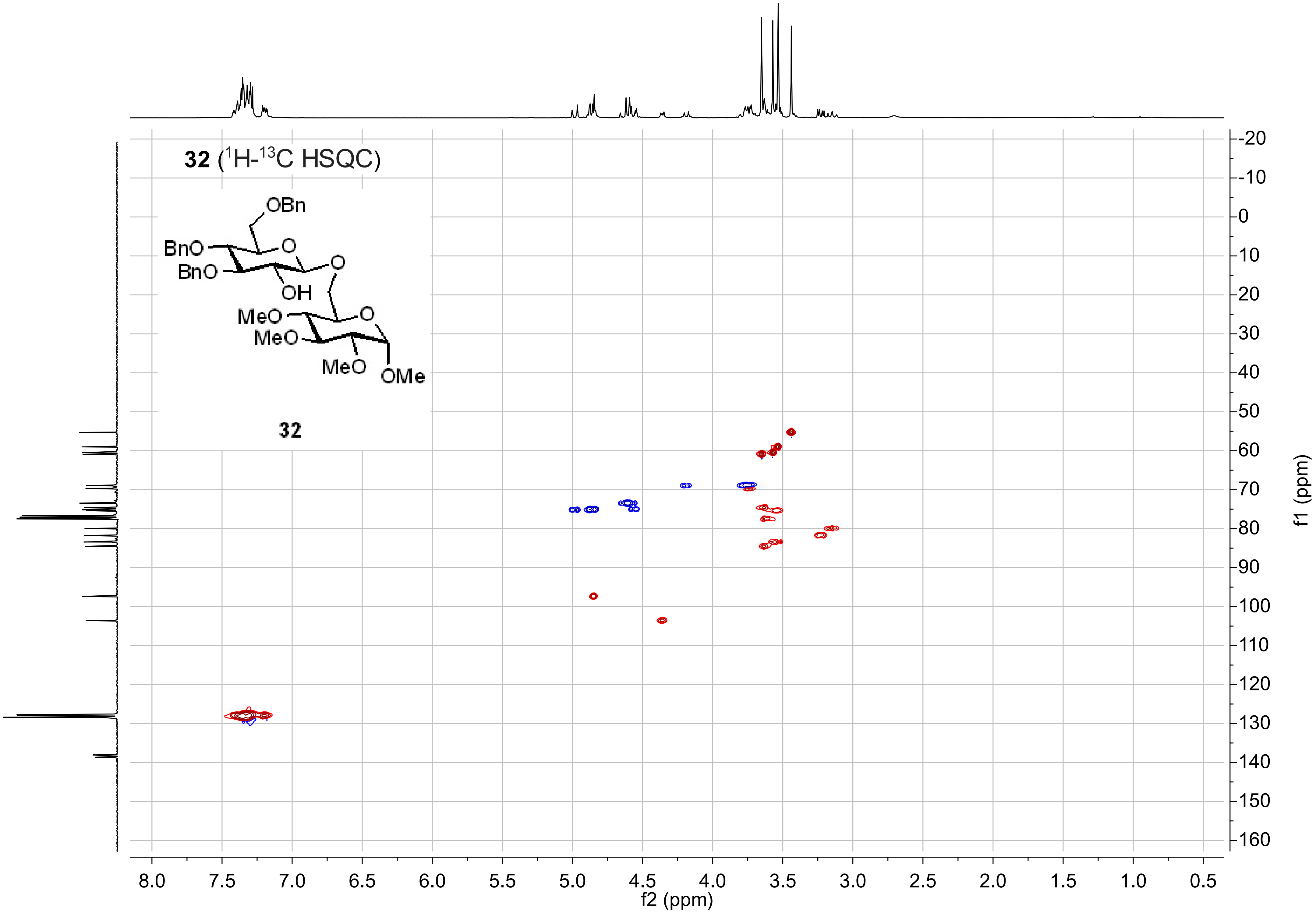

Supplementary Figure 61. **^{1}H-^{13}C HSQC Decoupled Spectrum for Compound 32**

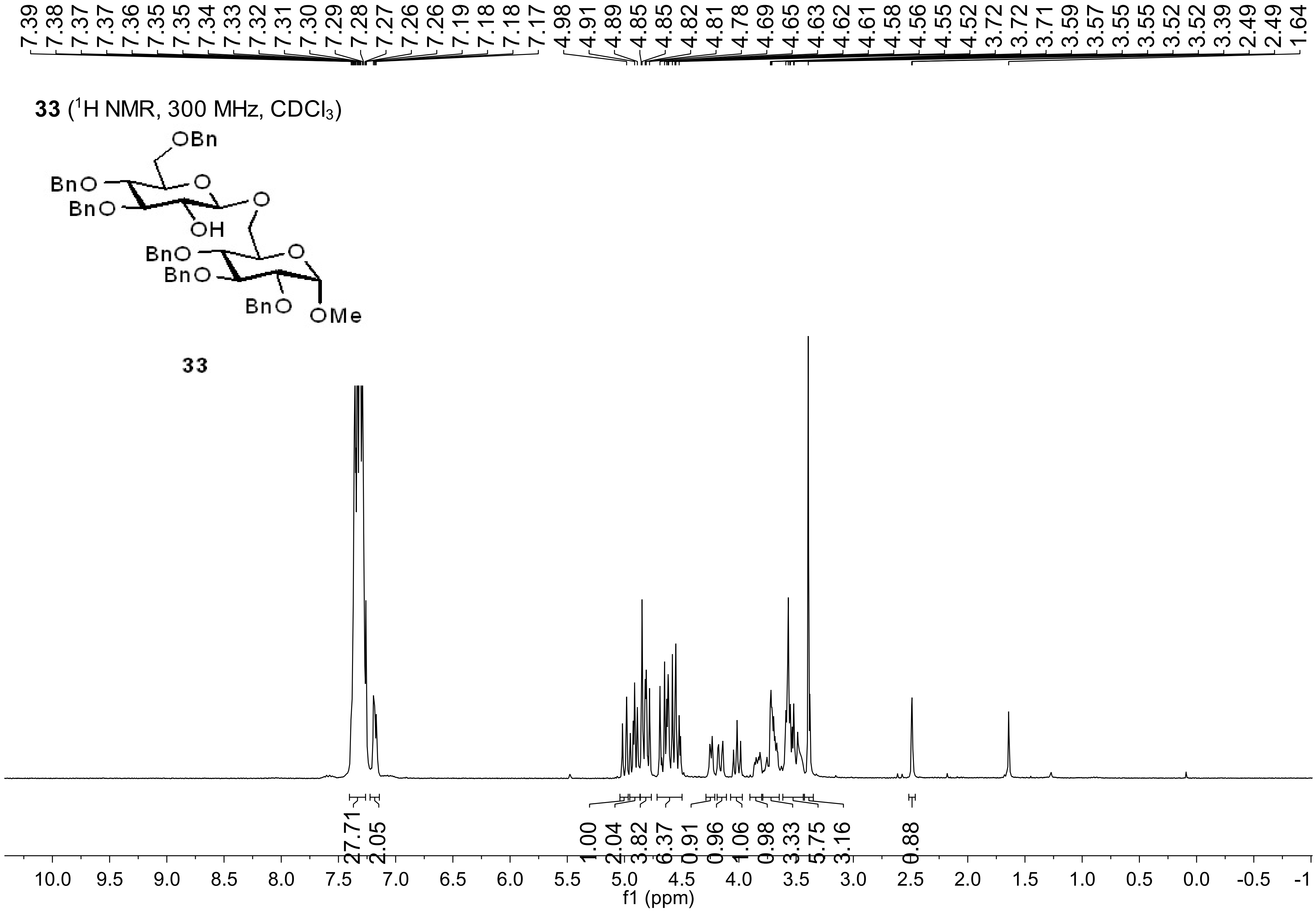

Supplementary Figure 62. ¹H NMR Spectrum for Compound 33

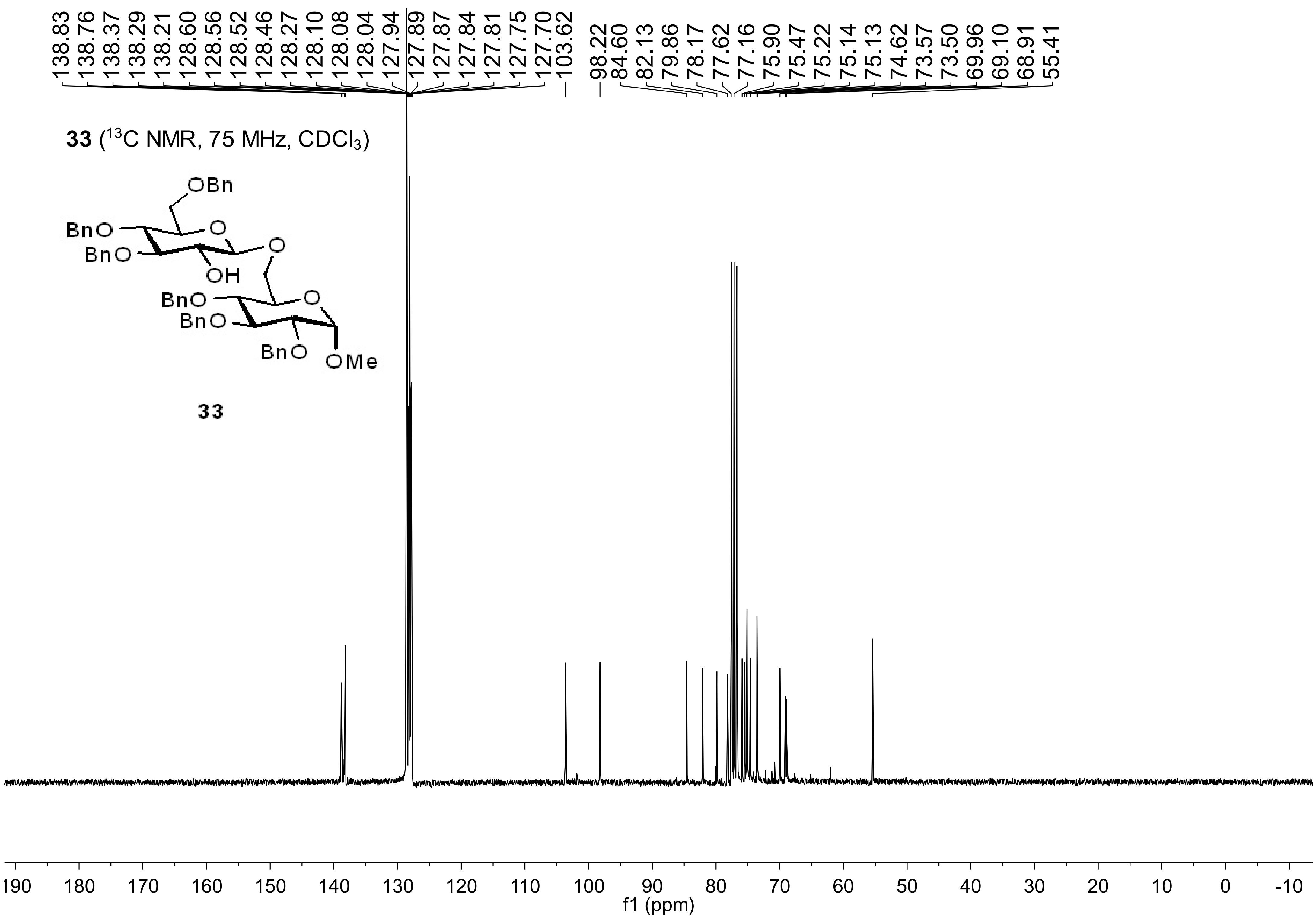

Supplementary Figure 63. ^{13}C NMR Spectrum for Compound 33

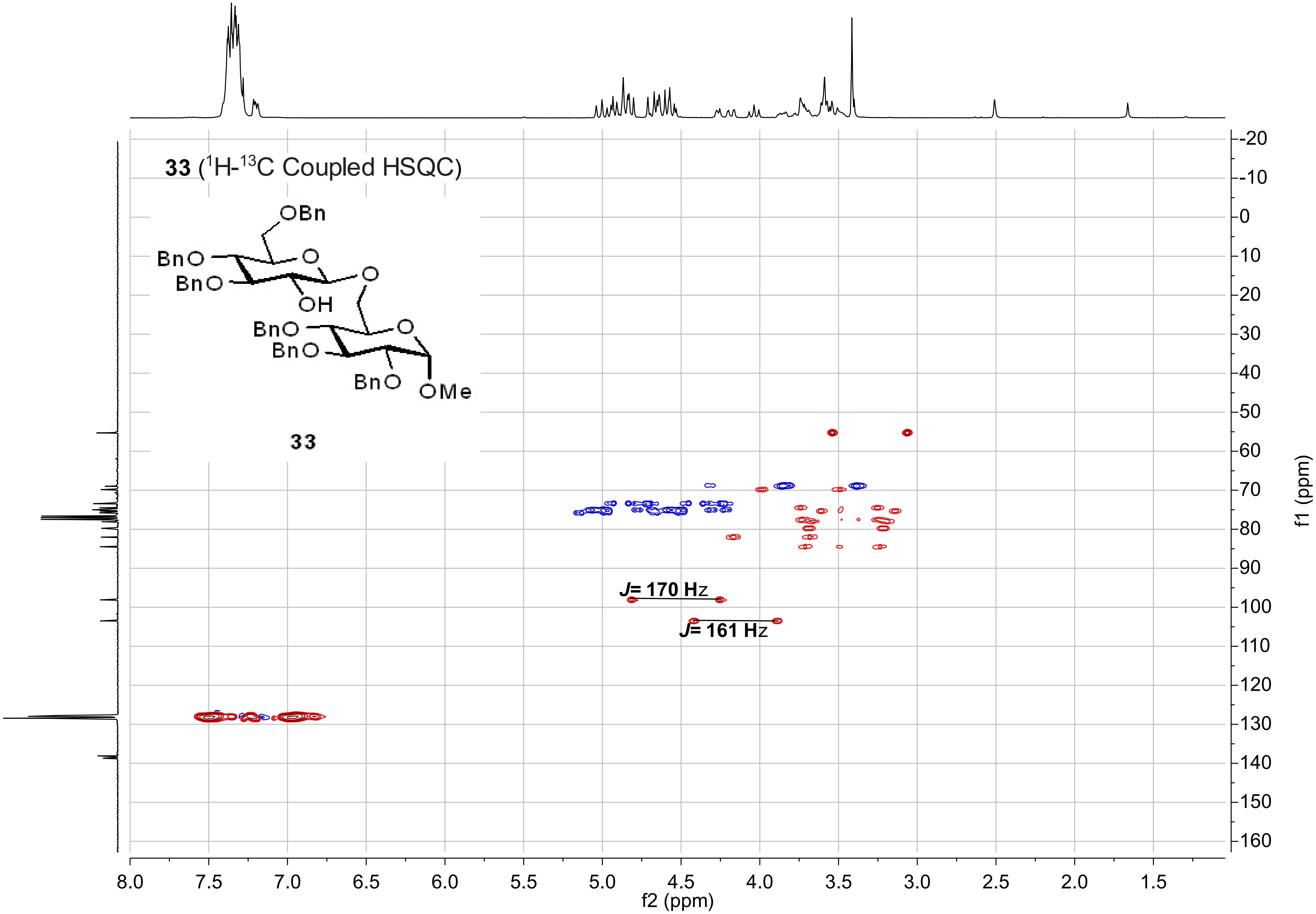

Supplementary Figure 64. ^{1}H-^{13}C HSQC Coupled Spectrum for Compound 33

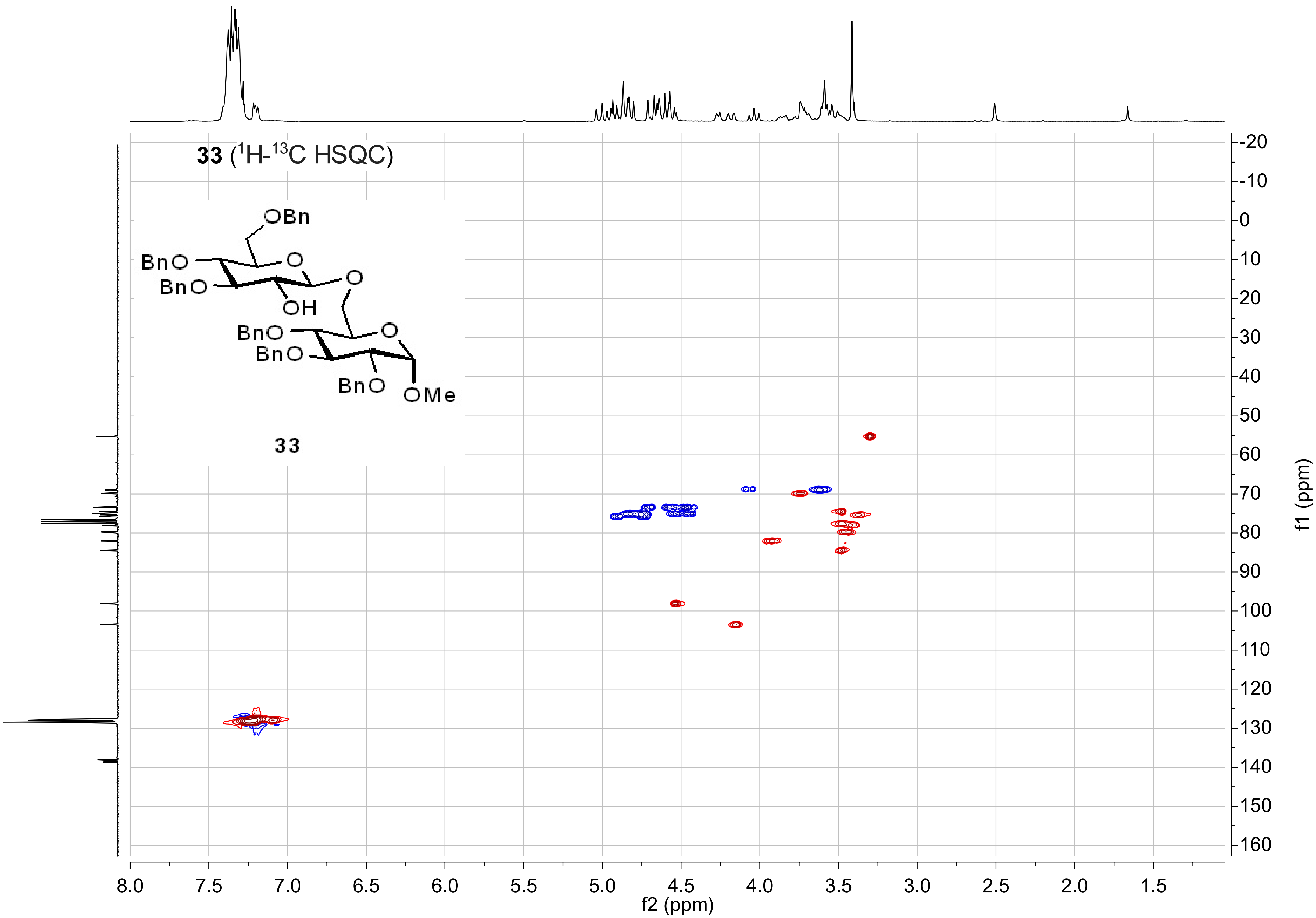

Supplementary Figure 65. ^{1}H-^{13}C HSQC Decoupled Spectrum for Compound 33

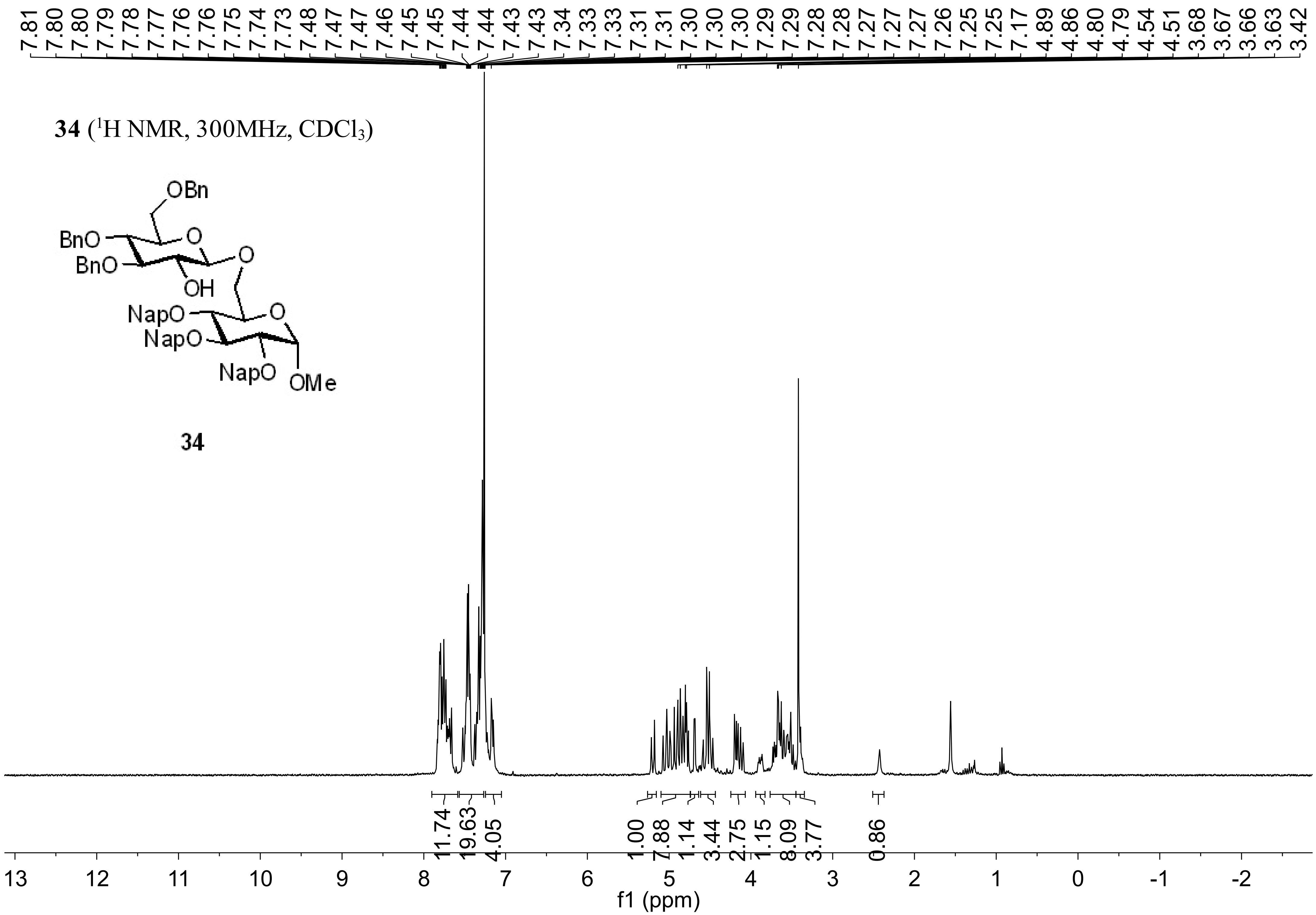

Supplementary Figure 66. ^{1}H NMR Spectrum for Compound 34

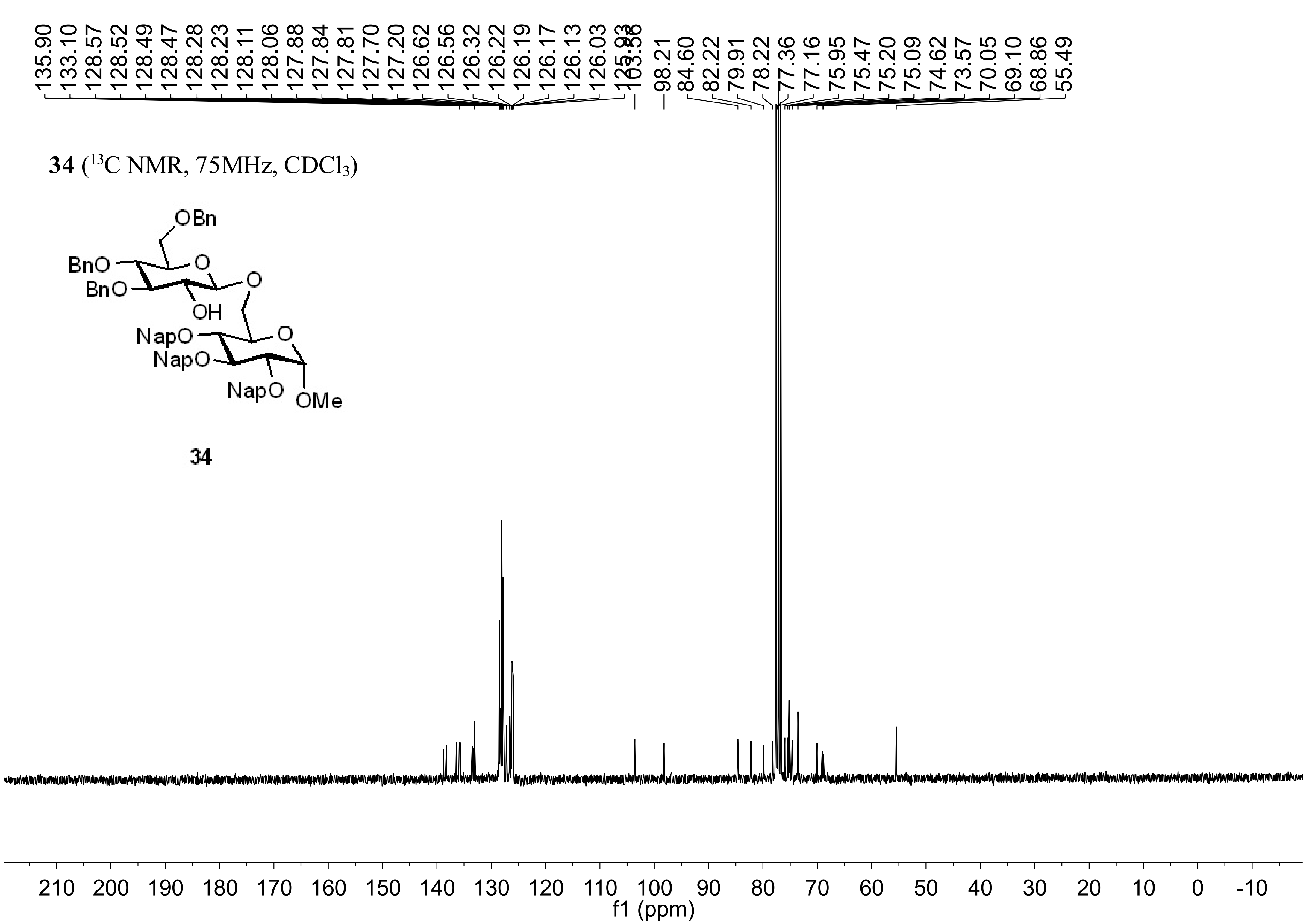

Supplementary Figure 67. ^{13}C NMR Spectrum for Compound 34

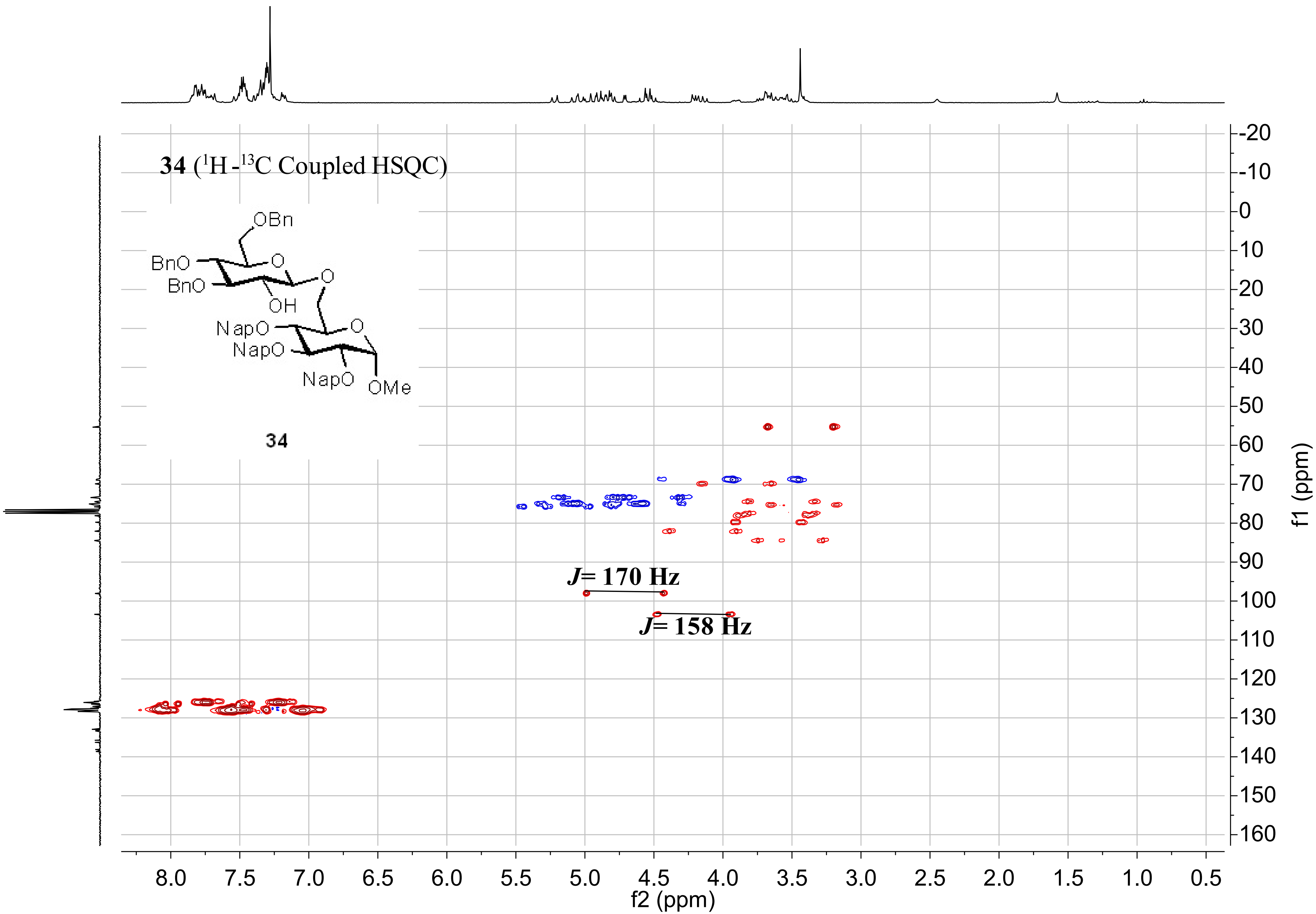

Supplementary Figure 68. ^{1}H-^{13}C HSQC Coupled Spectrum for Compound 34

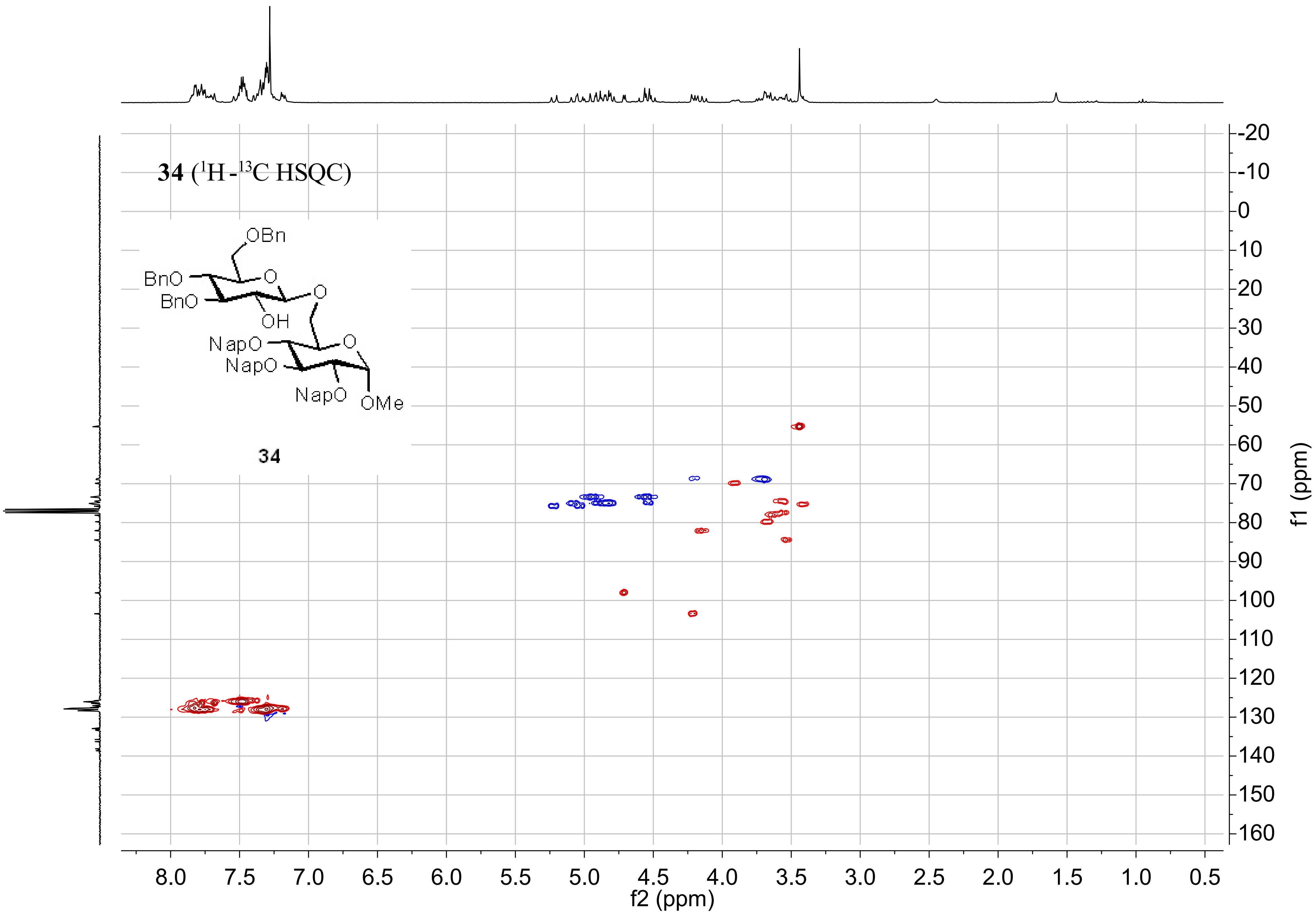

Supplementary Figure 69. ^{1}H-^{13}C HSQC Decoupled Spectrum for Compound 34

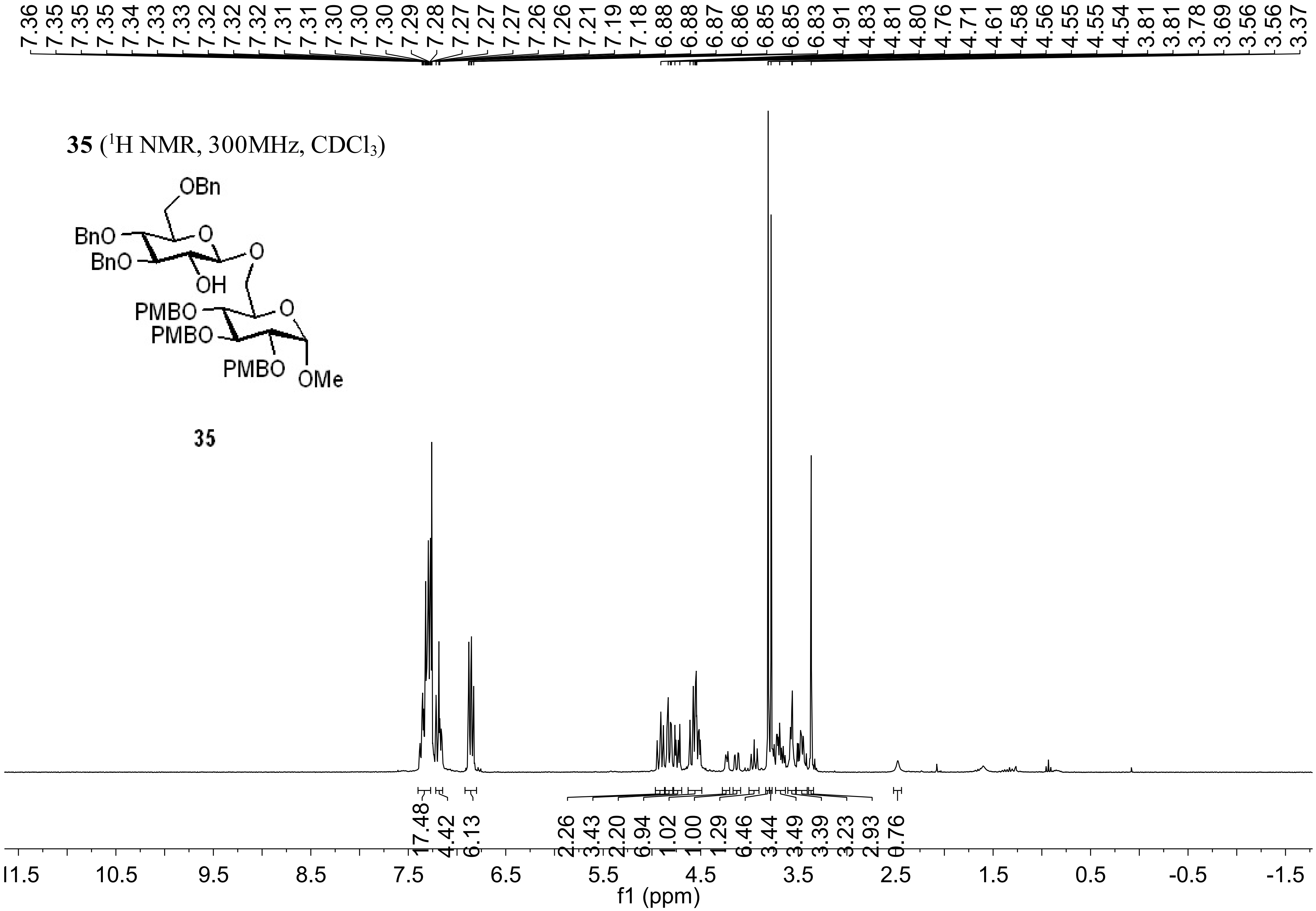

Supplementary Figure 70. ^{1}H NMR Spectrum for Compound 35

35 (^{13}C NMR, 75MHz, $CDCl_3$)

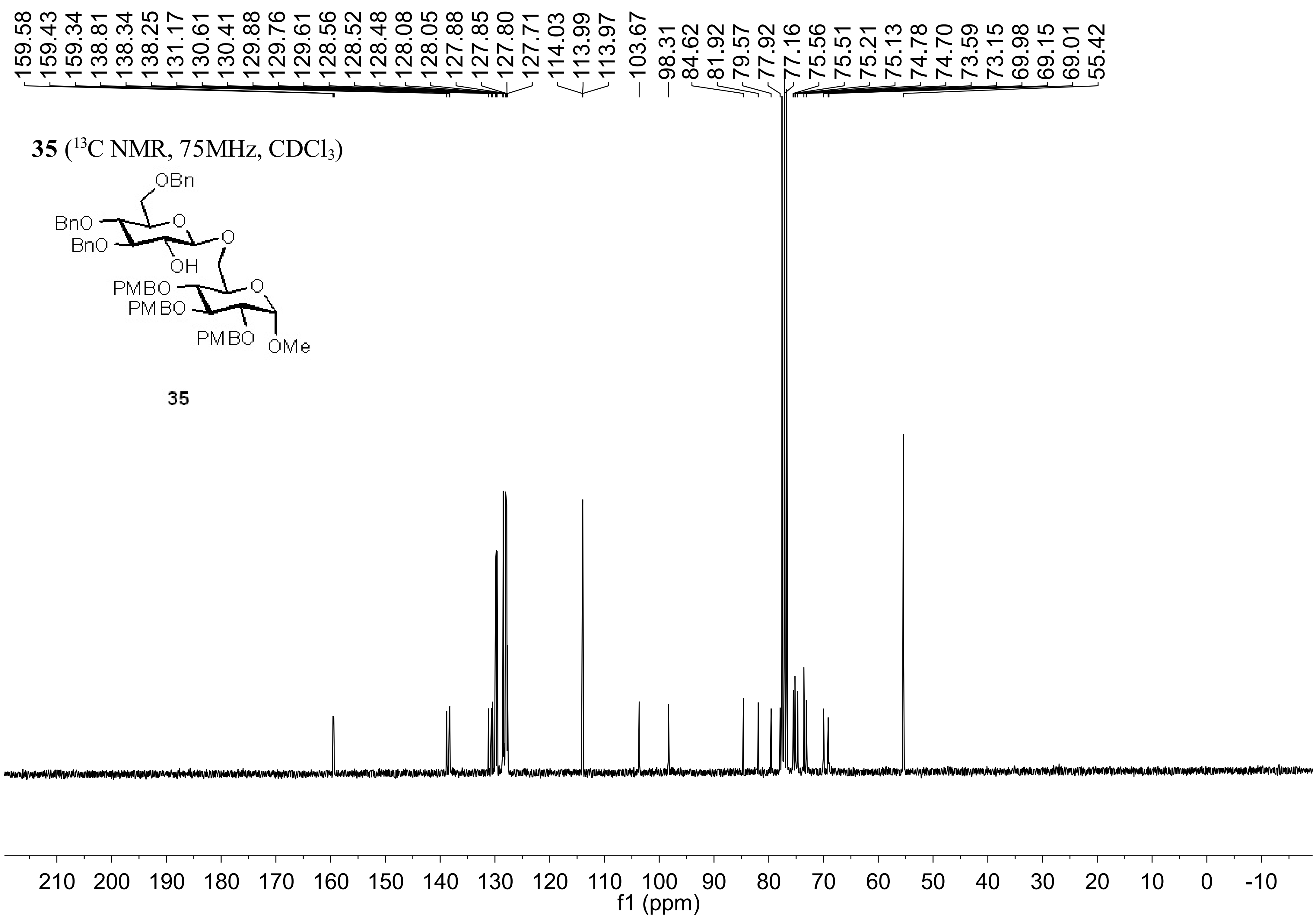

Supplementary Figure 71. ^{13}C NMR Spectrum for Compound 35

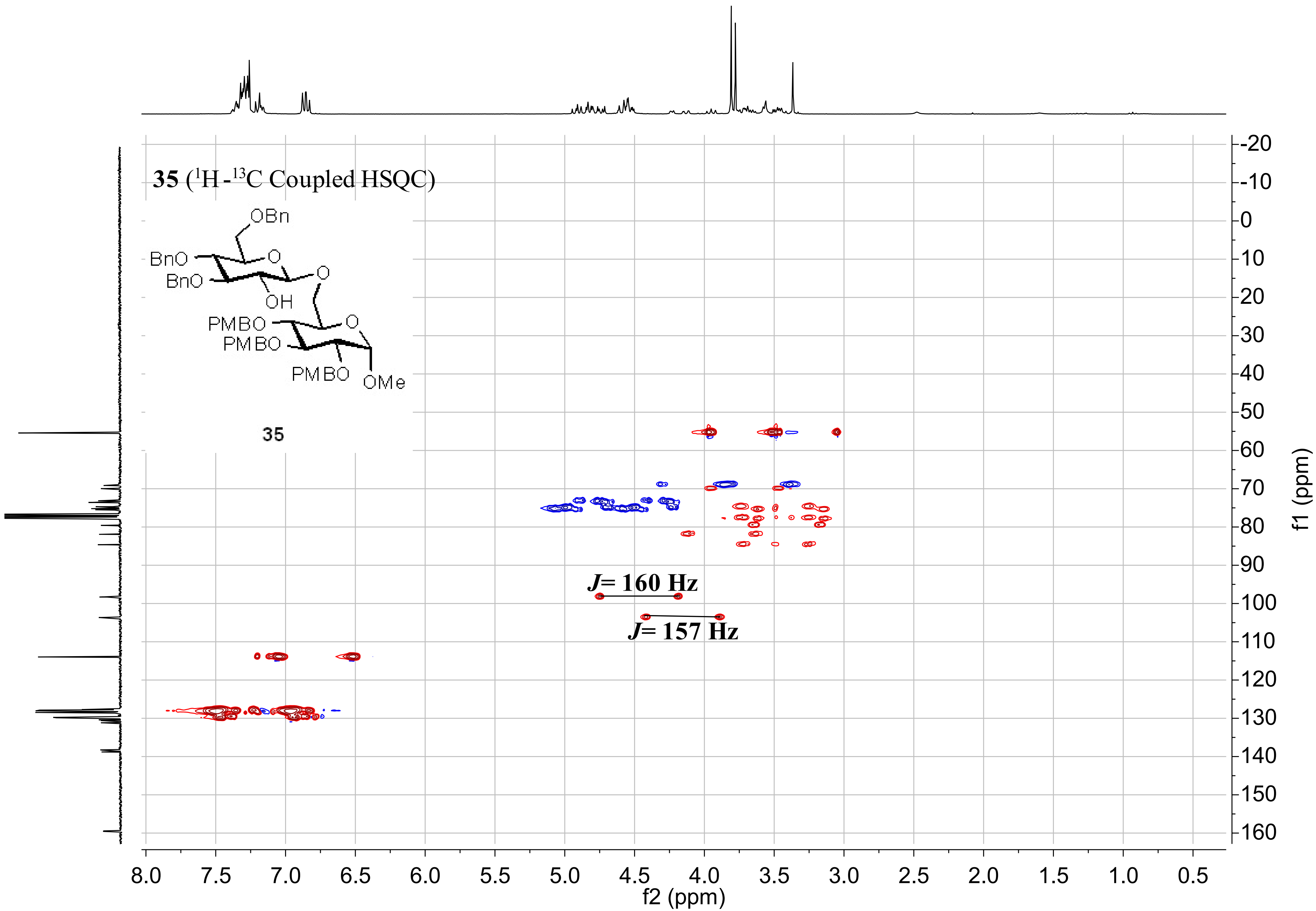

Supplementary Figure 72. ^{1}H-^{13}C HSQC Coupled Spectrum for Compound 35

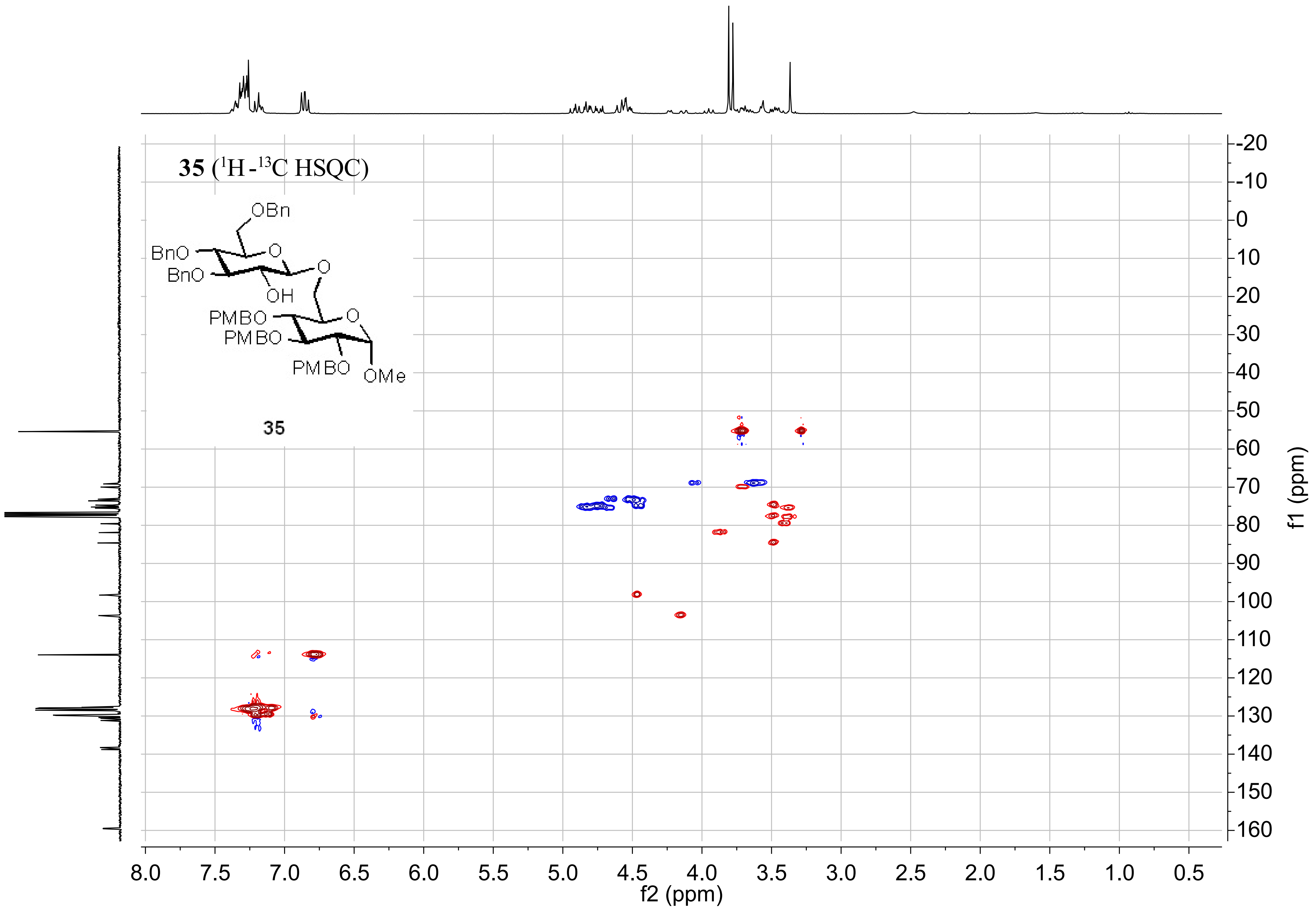

Supplementary Figure 73. ^{1}H-^{13}C **HSQC Decoupled Spectrum for Compound 35**

36 ^{1}H NMR (500 MHz, CDCl$_3$)

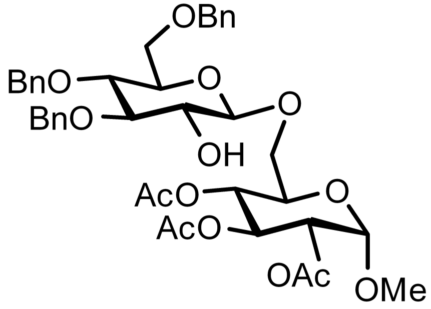

36

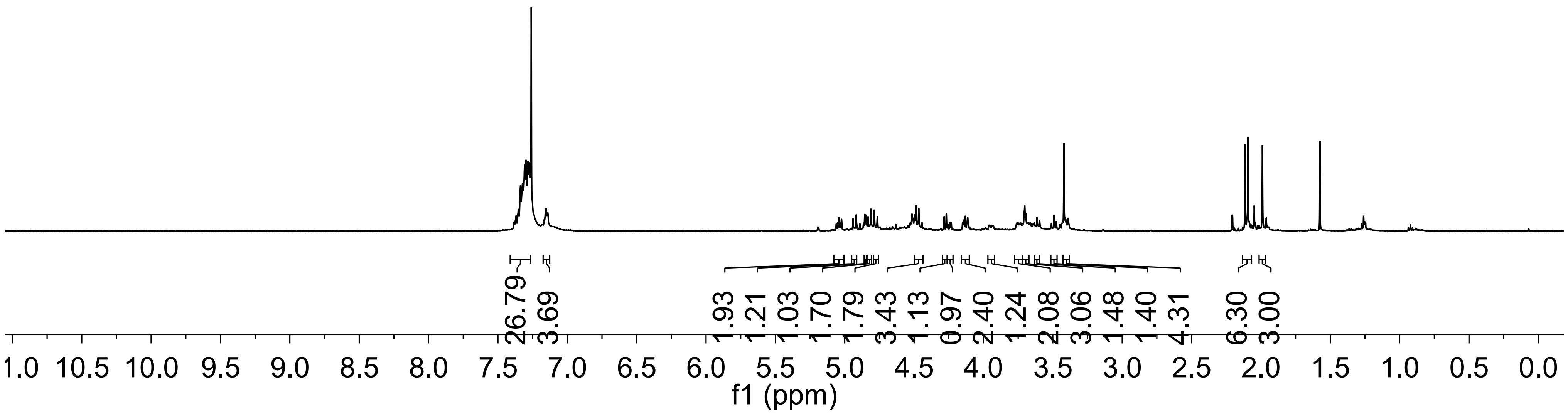

Supplementary Figure 74. ^{1}H NMR Spectrum for Compound 36

36 ^{13}C NMR (126 MHz, CDCl$_3$)

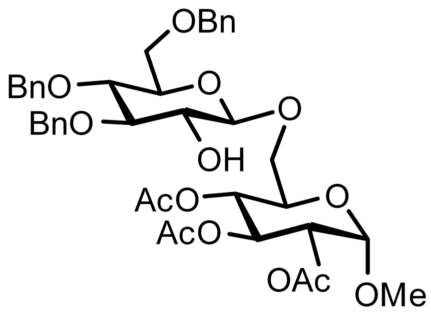

36

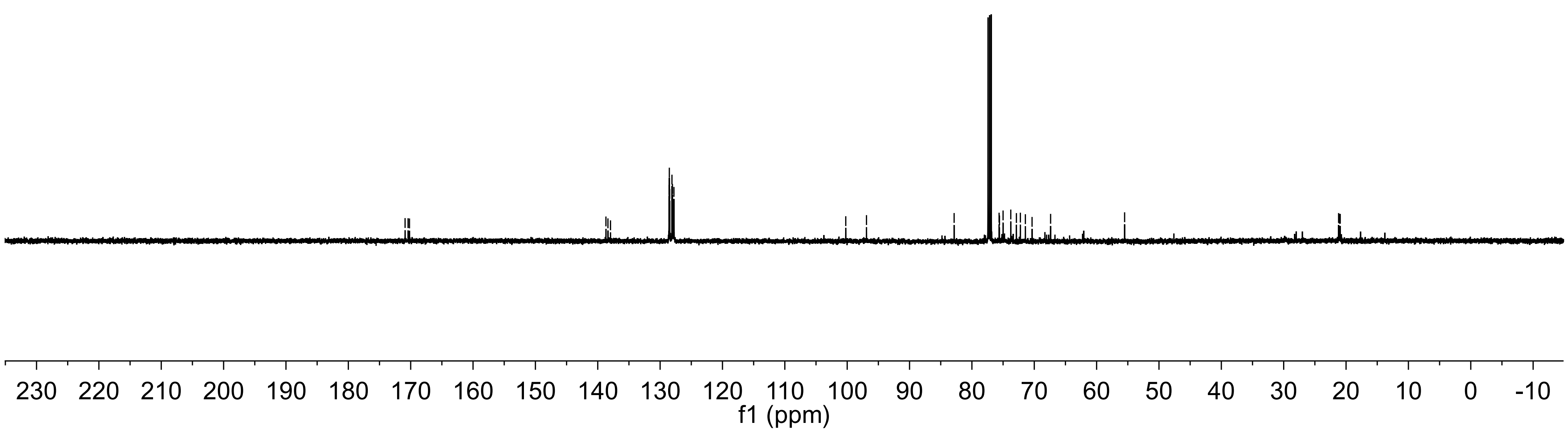

Supplementary Figure 75. ^{13}C NMR Spectrum for Compound 36

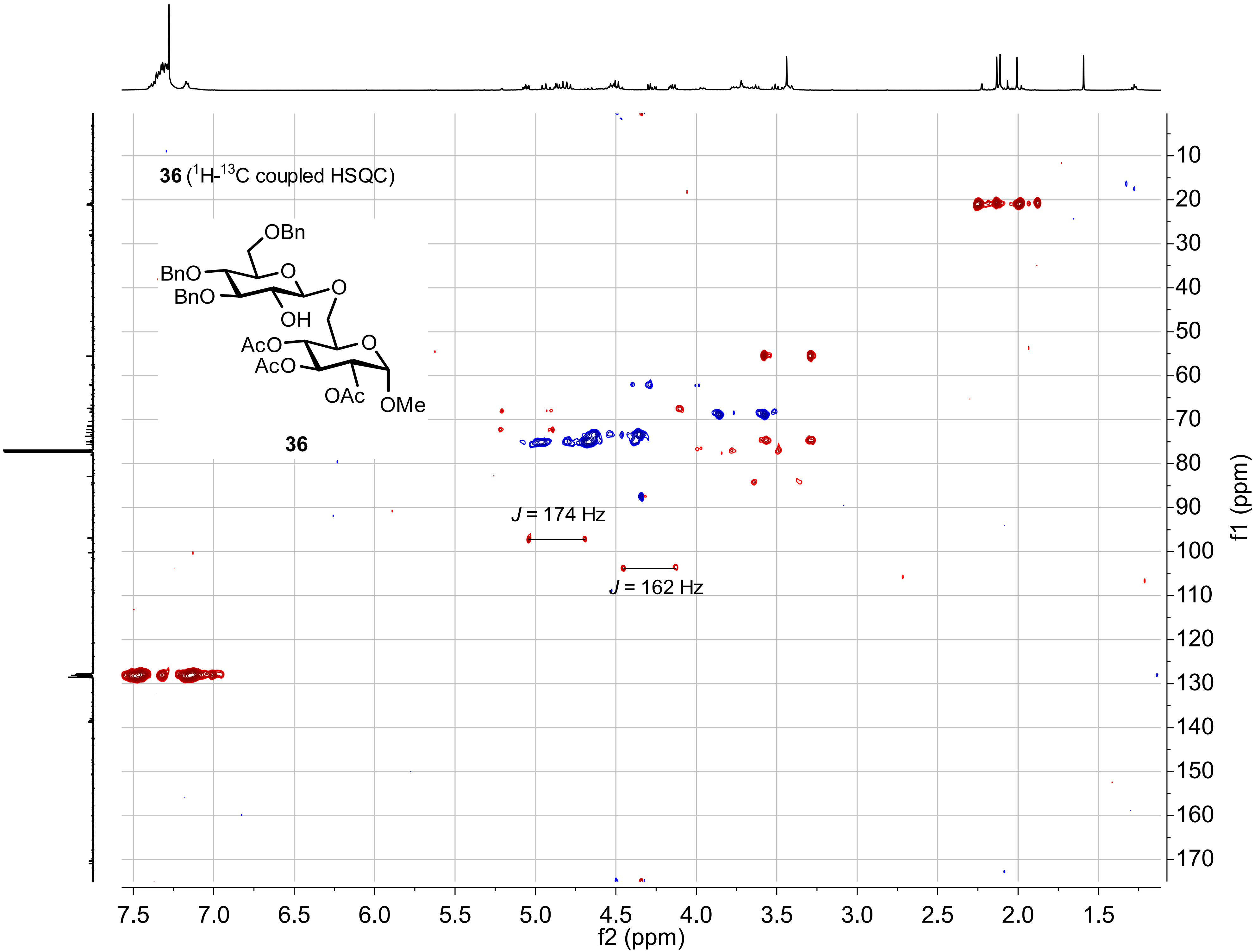

Supplementary Figure 76. ^{1}H-^{13}C HSQC Decoupled Spectrum for Compound 36

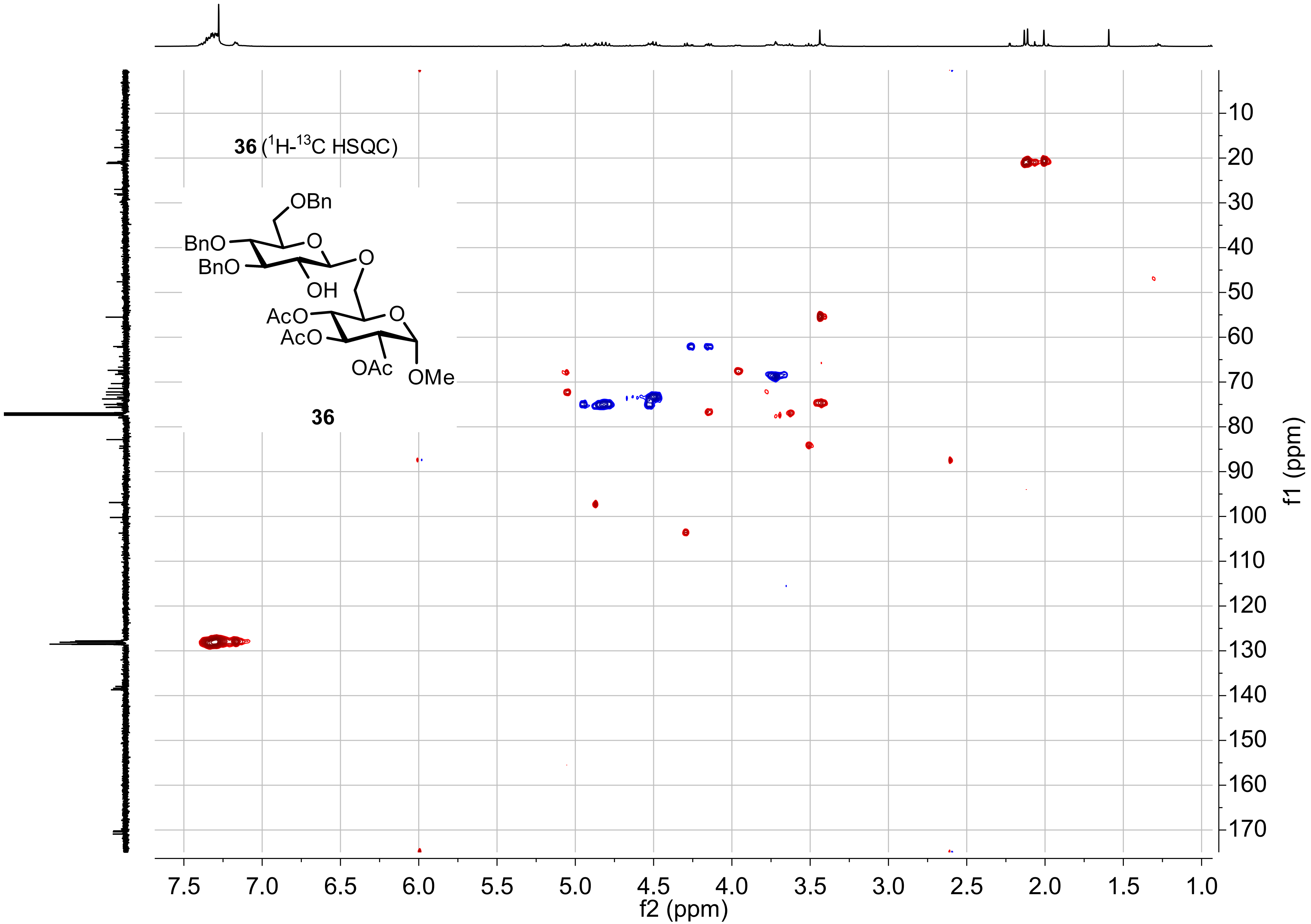

Supplementary Figure 77. **^{1}H-^{13}C HSQC Decoupled Spectrum for Compound 36**

37 (^{1}H NMR, 300MHz, $CDCl_3$)

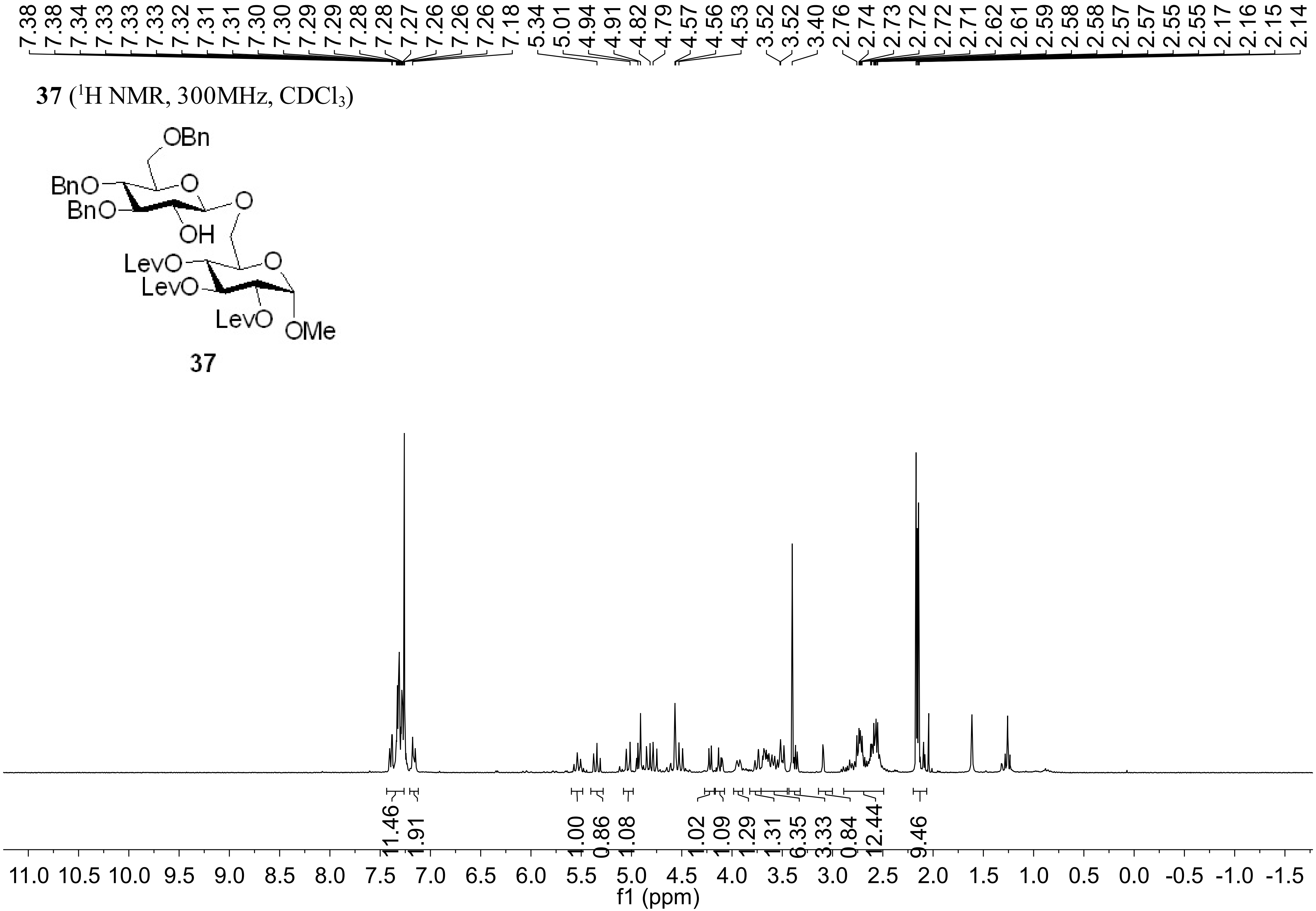

Supplementary Figure 78. ^{1}H NMR Spectrum for Compound 37

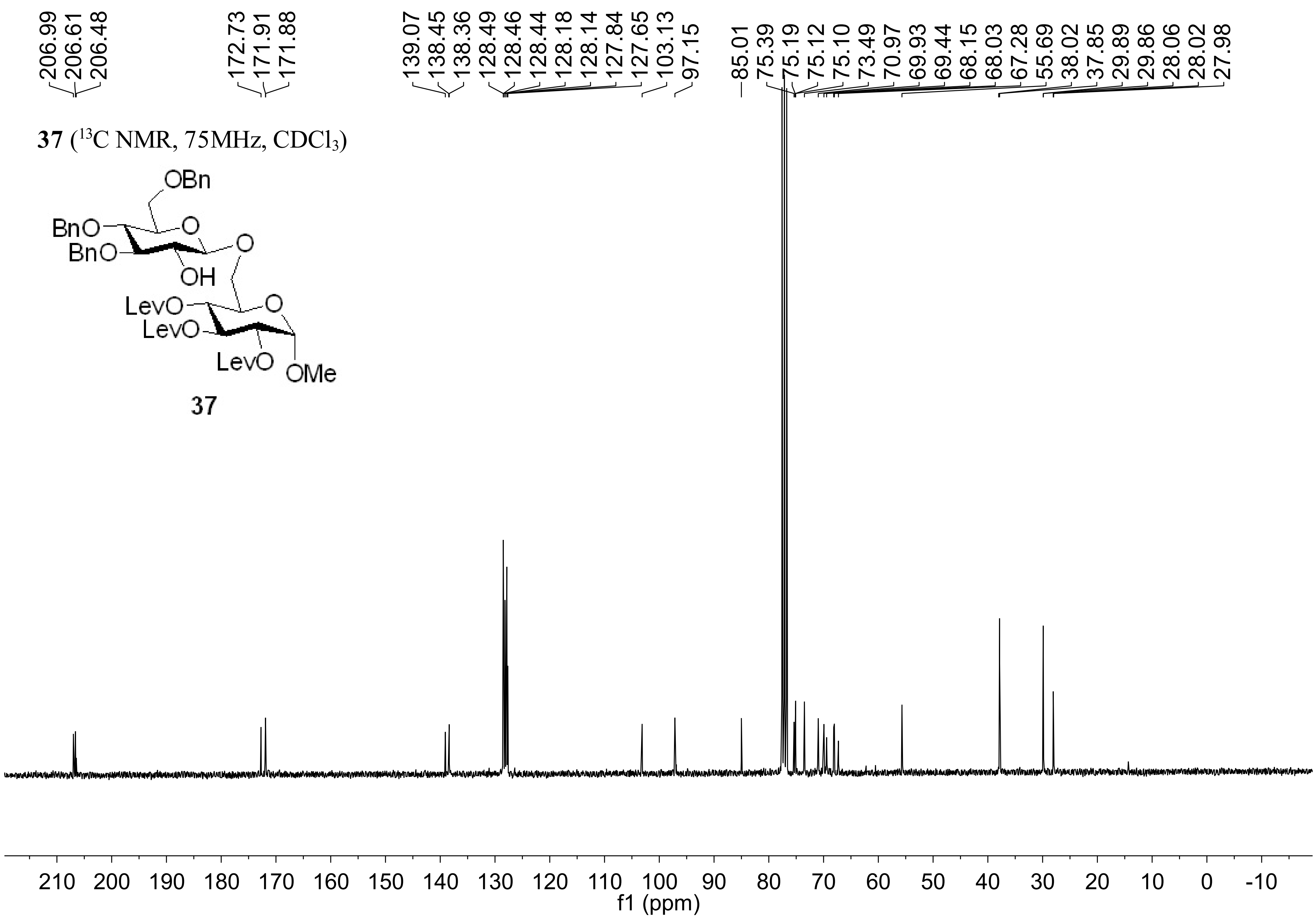

Supplementary Figure 79. **^{13}C NMR Spectrum for Compound 37**

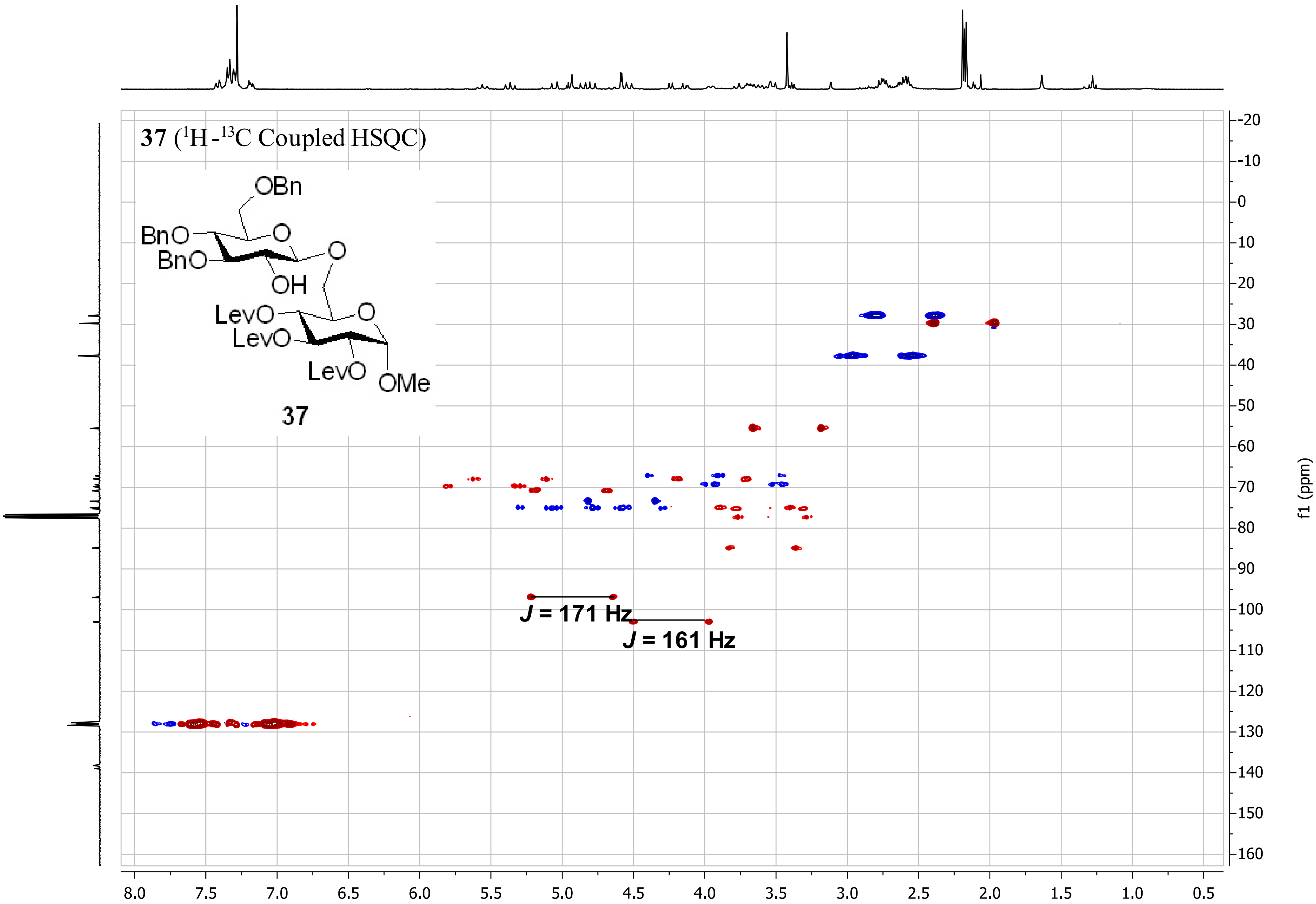

Supplementary Figure 80. **^{1}H-^{13}C HSQC Coupled Spectrum for Compound 37**

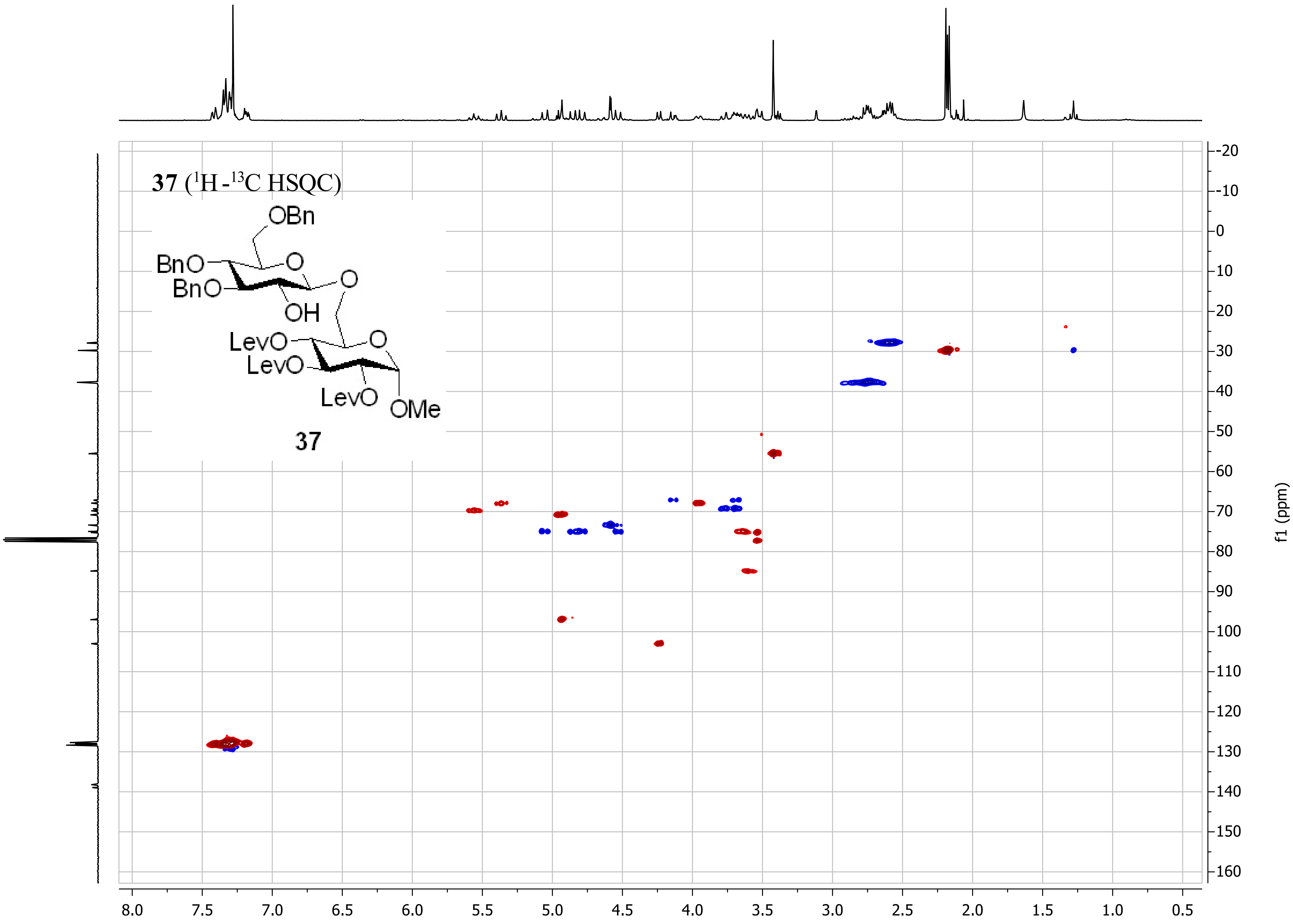

Supplementary Figure 81. **^{1}H-^{13}C HSQC Decoupled Spectrum for Compound 37**

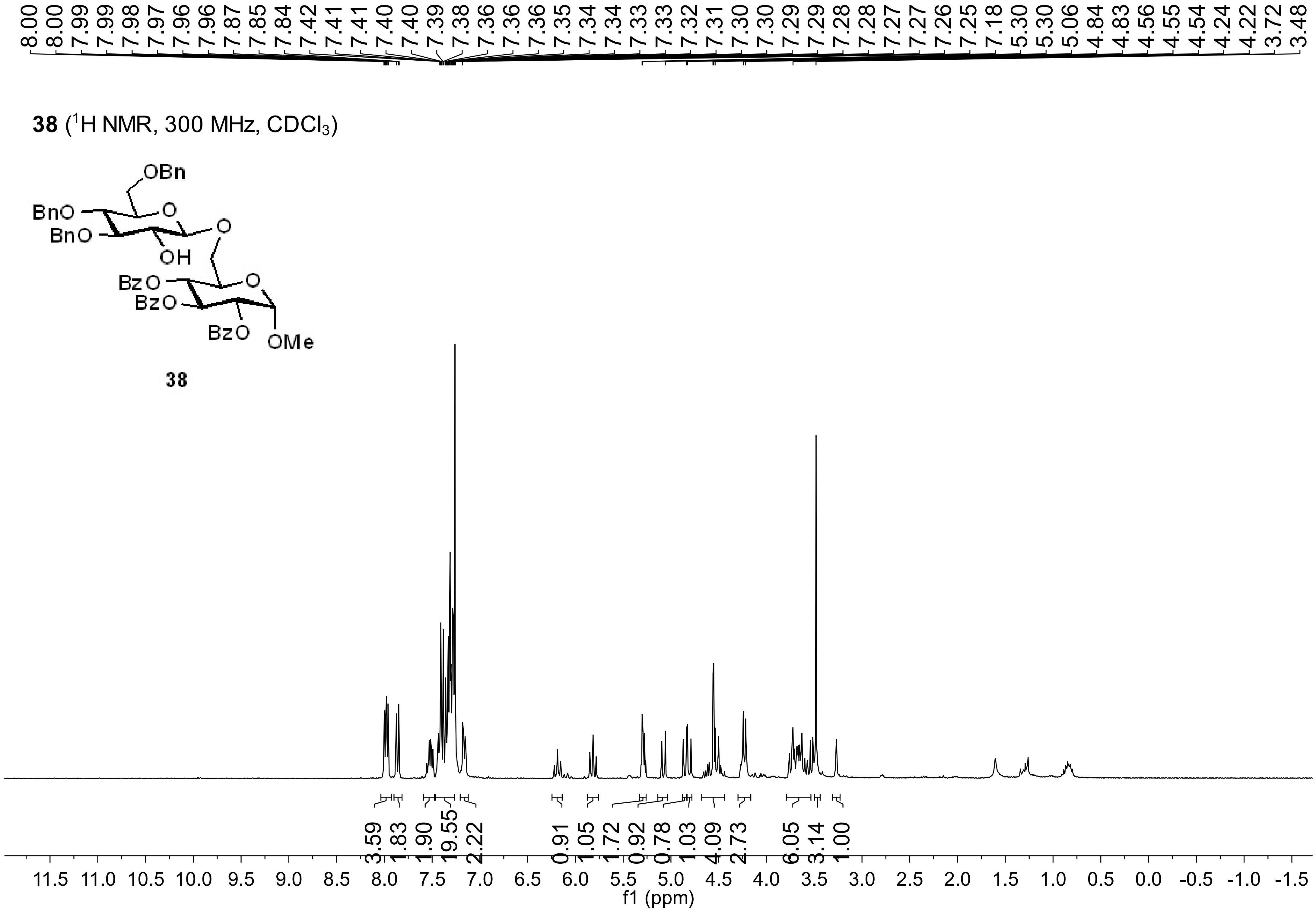

Supplementary Figure 82. ^{1}H NMR Spectrum for Compound 38

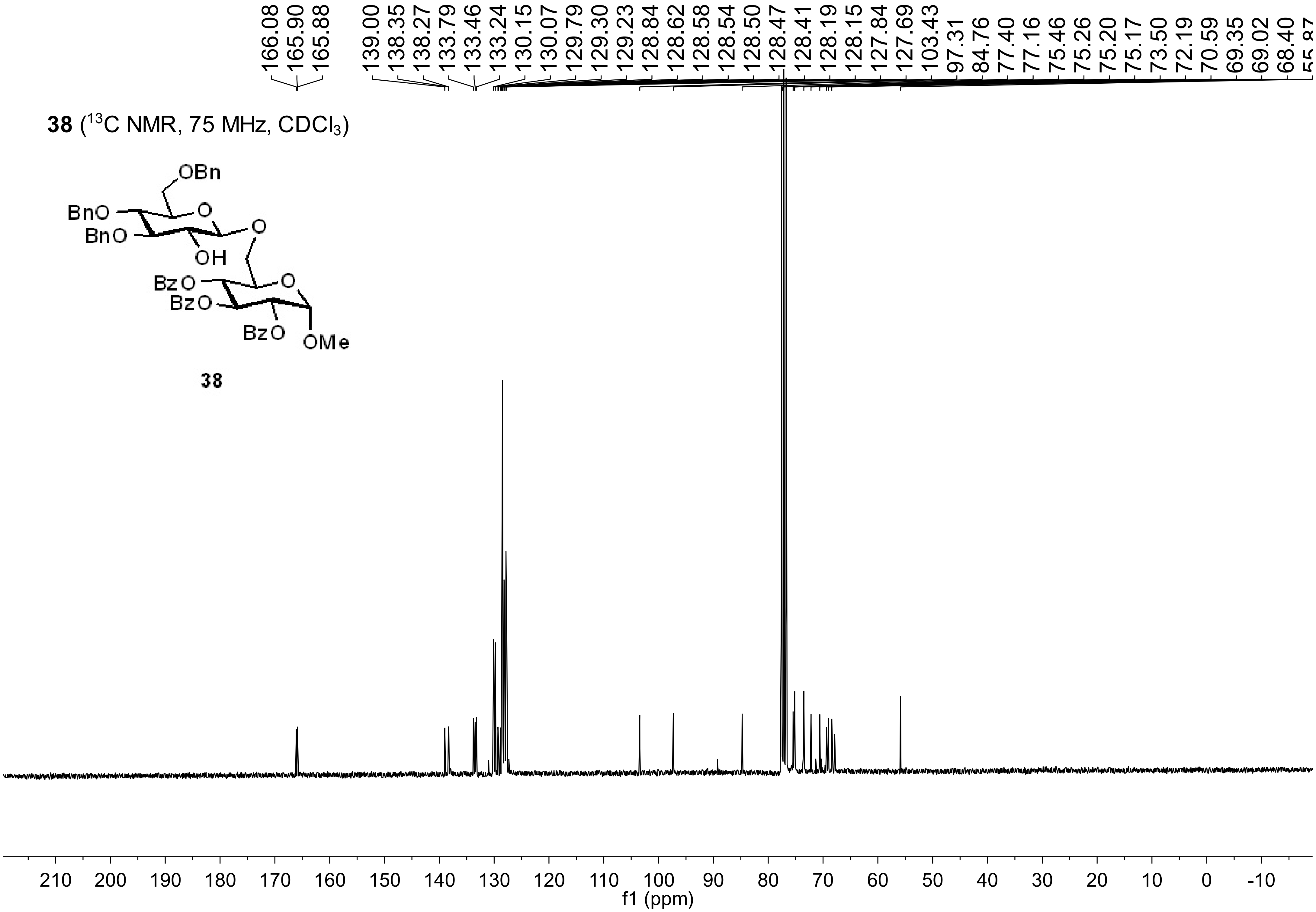

Supplementary Figure 83. ^{13}C NMR Spectrum for Compound 38

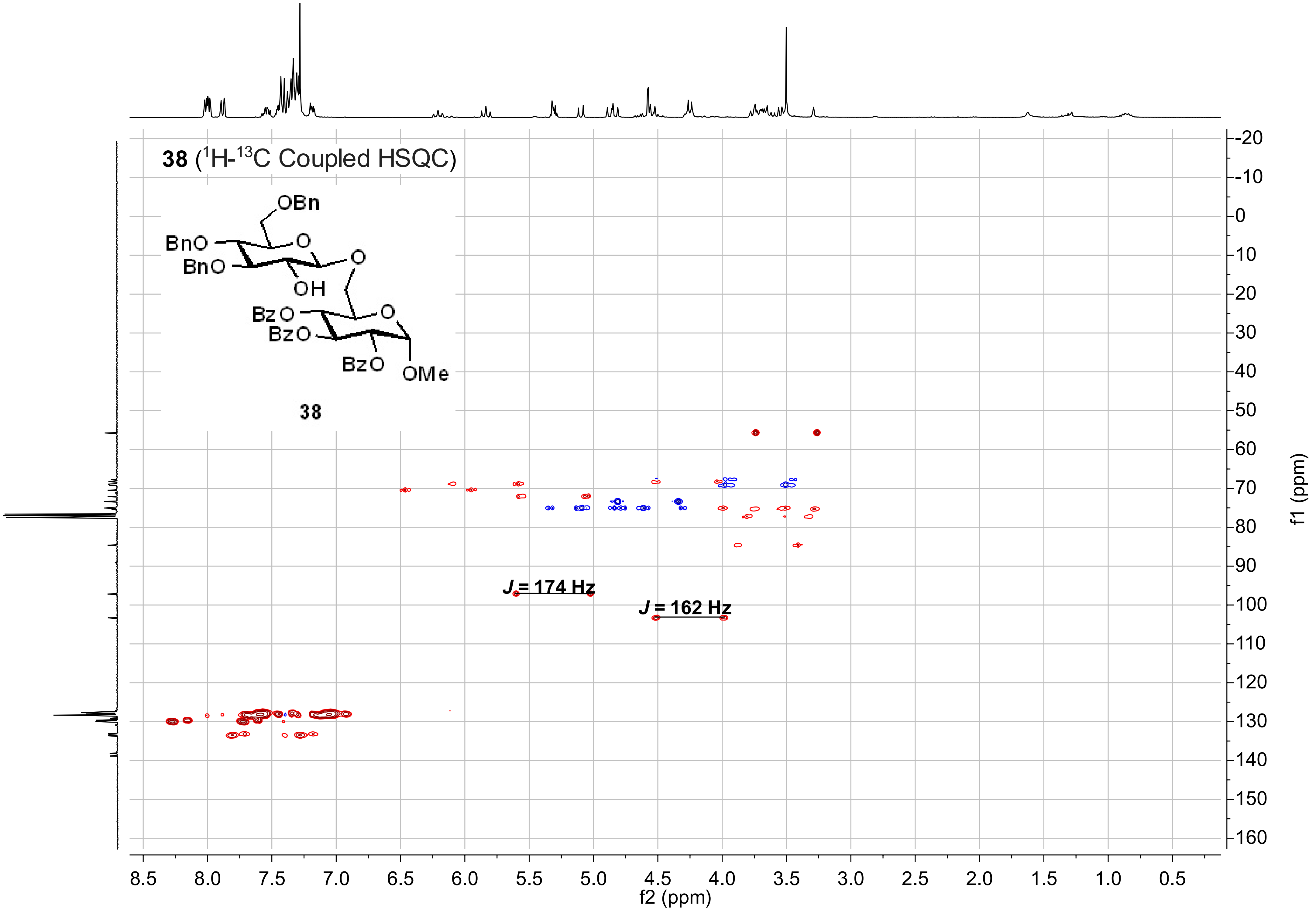

Supplementary Figure 84. **^{1}H-^{13}C HSQC Coupled Spectrum for Compound 38**

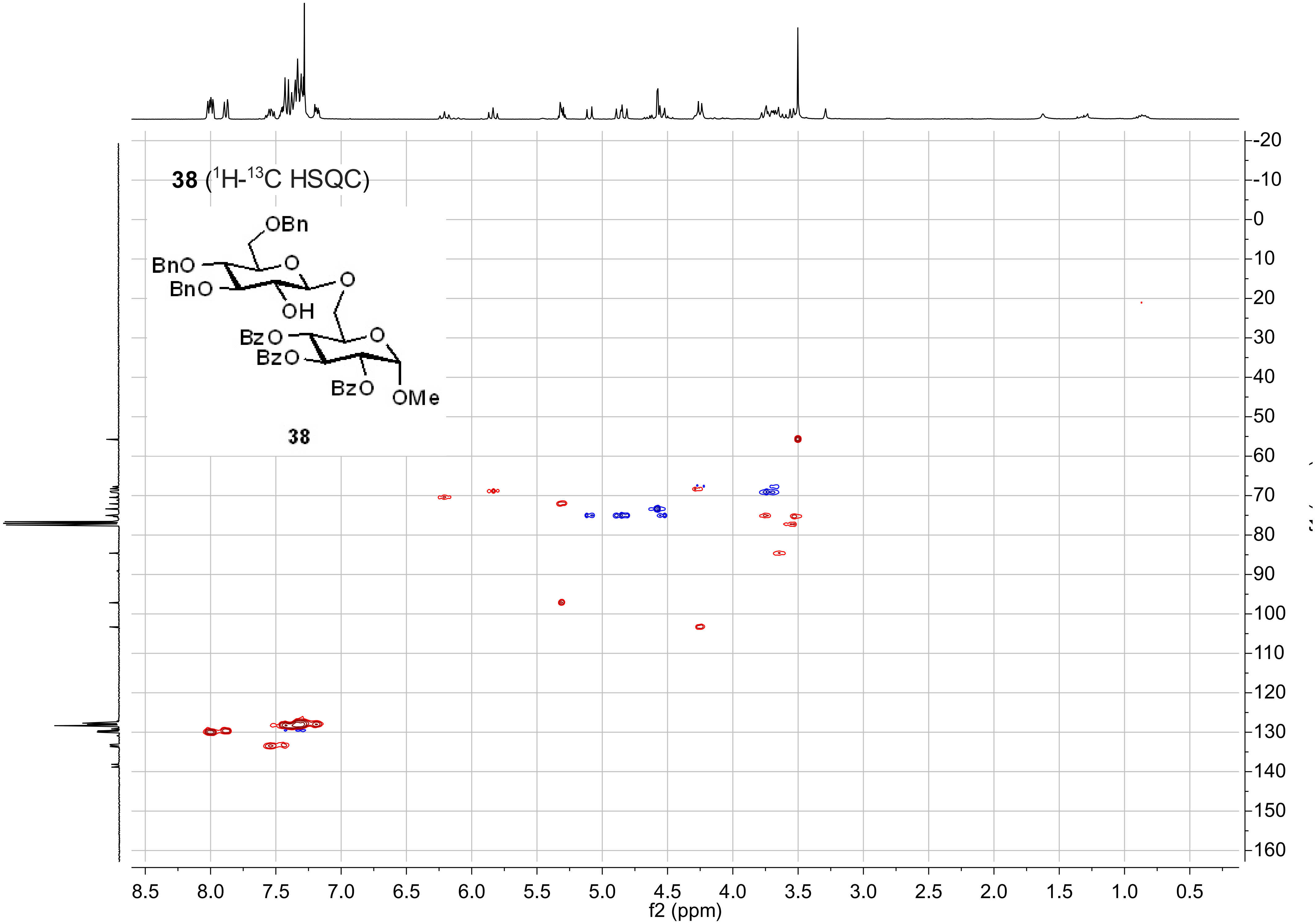

Supplementary Figure 85. **^{1}H-^{13}C HSQC Decoupled Spectrum for Compound 38**

39 (^{1}H NMR, 300MHz, $CDCl_3$)

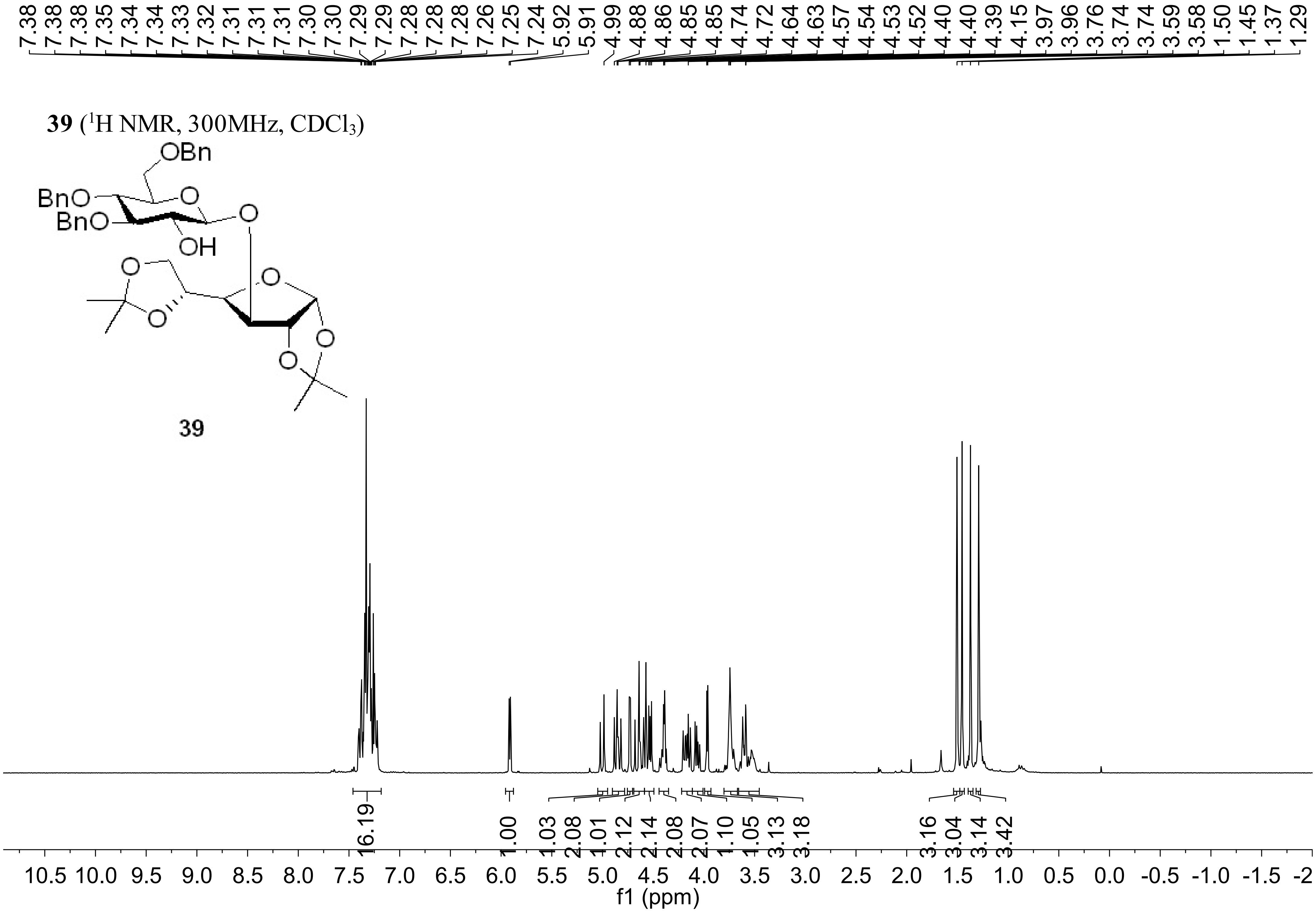

Supplementary Figure 86. ^{1}H NMR Spectrum for Compound 39

39 (^{13}C NMR, 75MHz, $CDCl_3$)

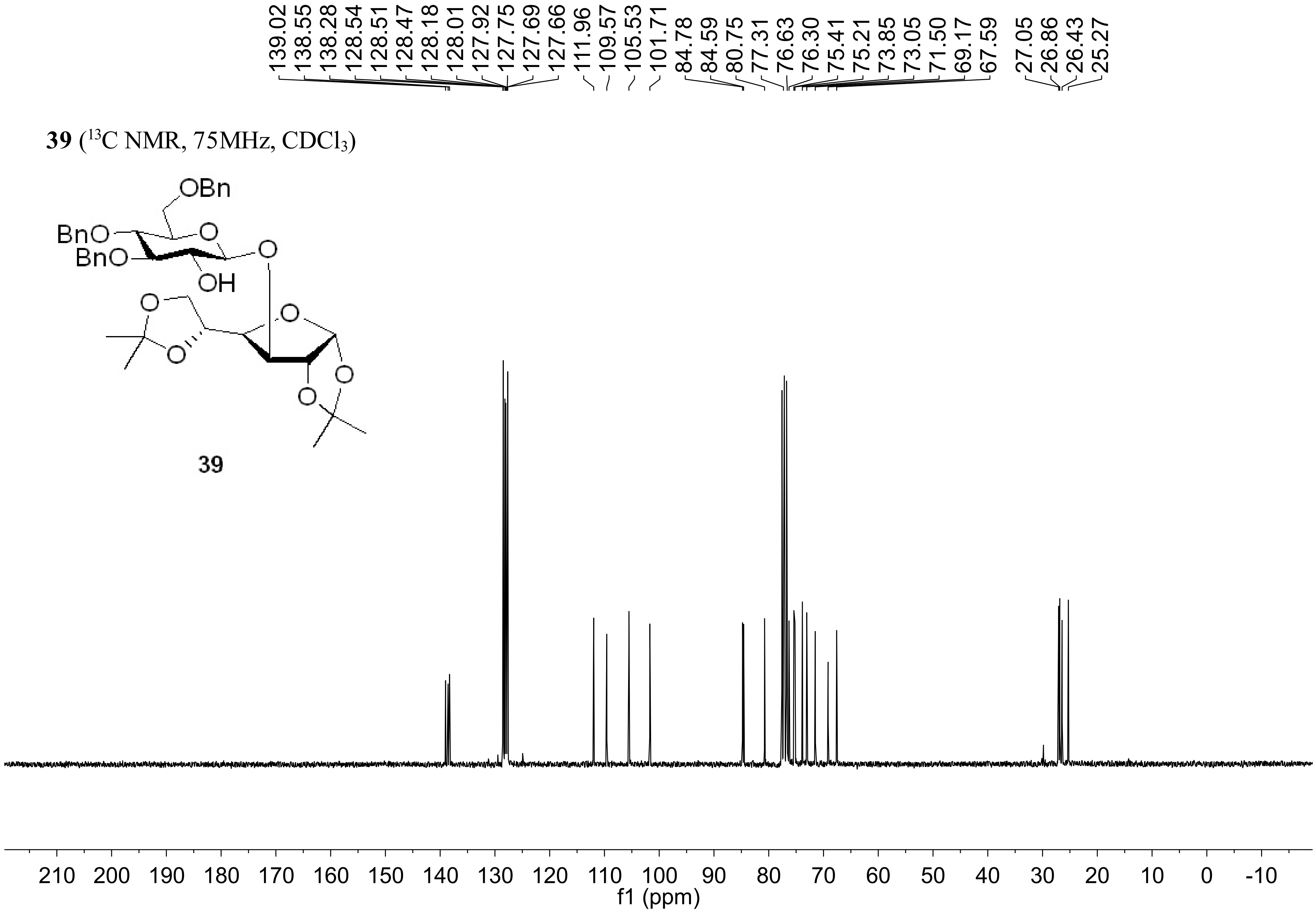

Supplementary Figure 87. **^{13}C NMR Spectrum for Compound 39**

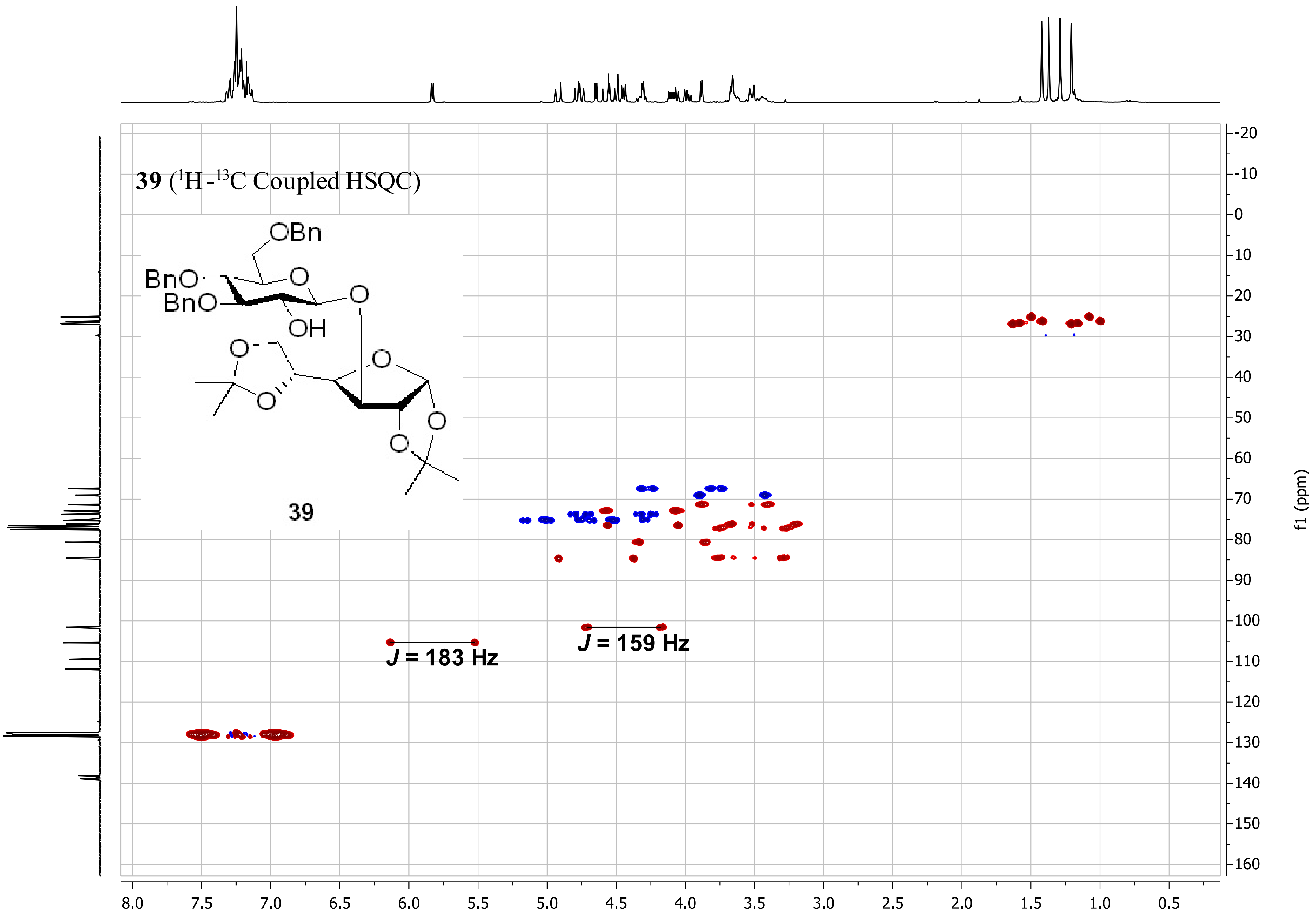

Supplementary Figure 88. ^{1}H-^{13}C HSQC Coupled Spectrum for Compound 39

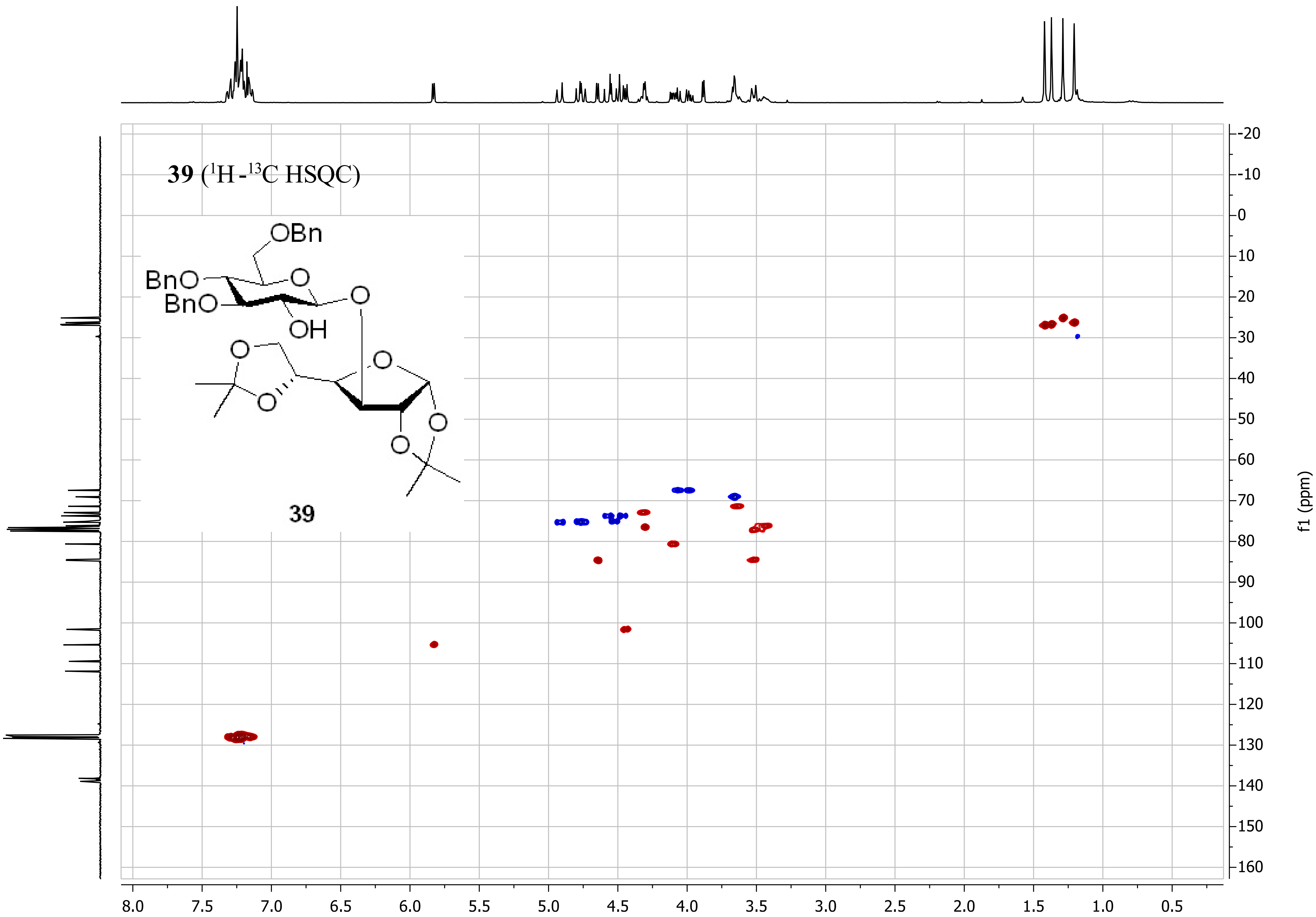

Supplementary Figure 89. ^{1}H-^{13}C HSQC Decoupled Spectrum for Compound 39

40 ^{1}H NMR (500 MHz, $CDCl_3$)

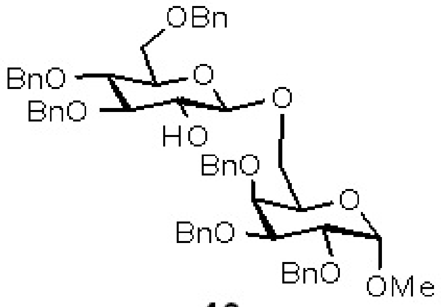

40

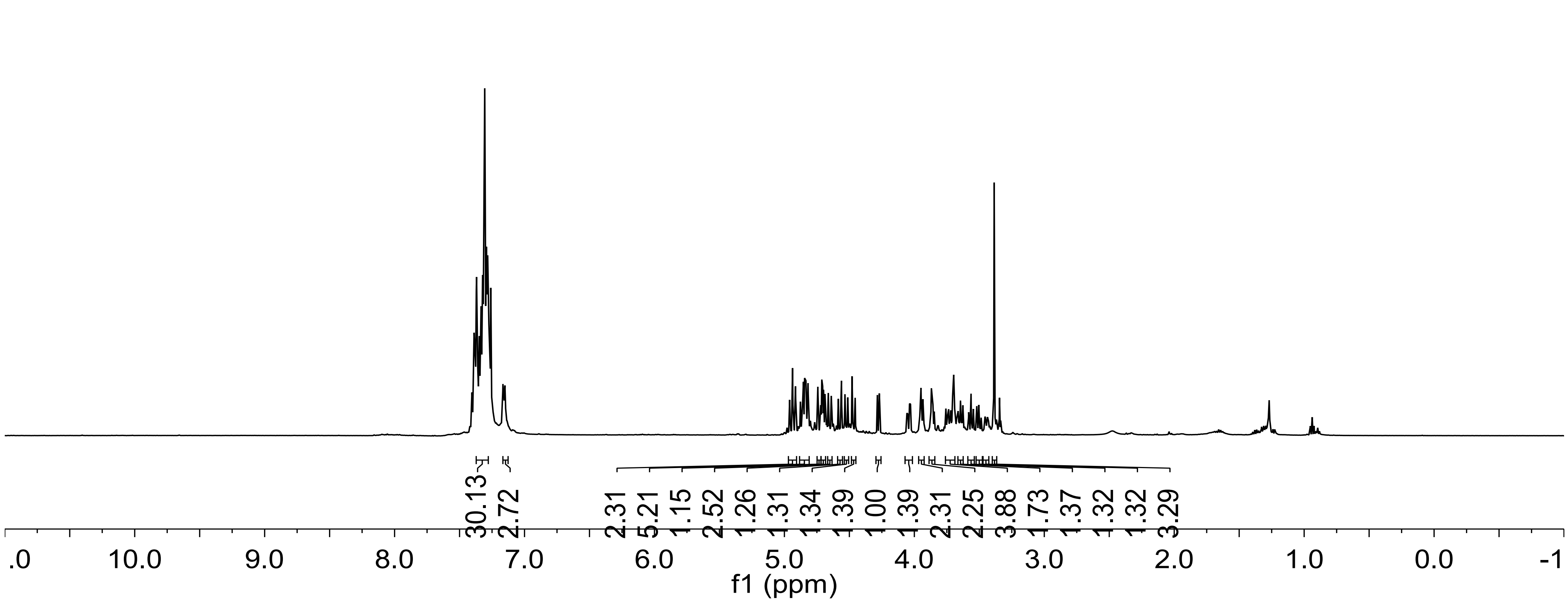

Supplementary Figure 90. ^{1}H NMR Spectrum for Compound 40

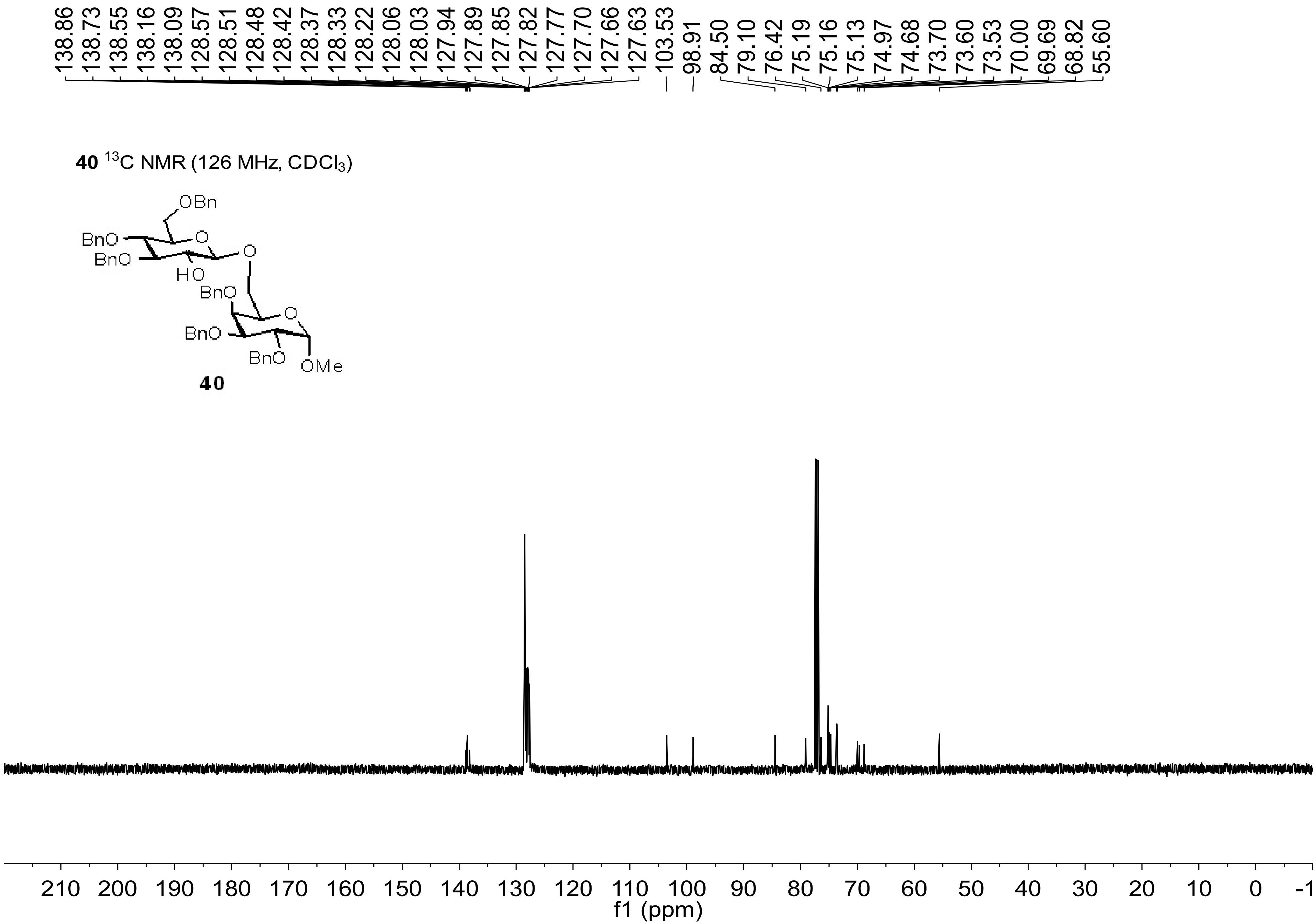

Supplementary Figure 91. ^{13}**C NMR Spectrum for Compound 40**

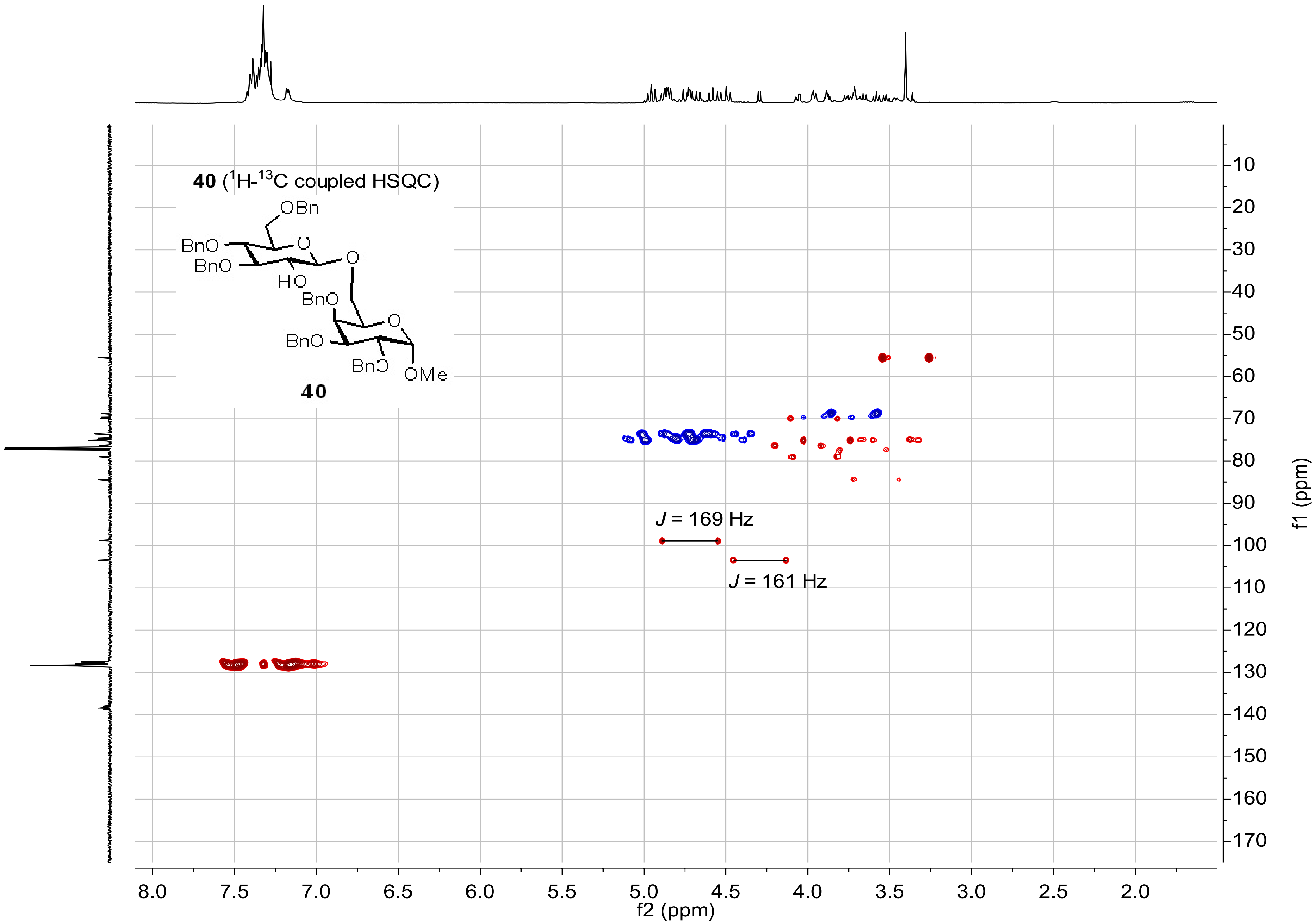

Supplementary Figure 92. ^{1}H-^{13}C **HSQC Coupled Spectrum for Compound 40**

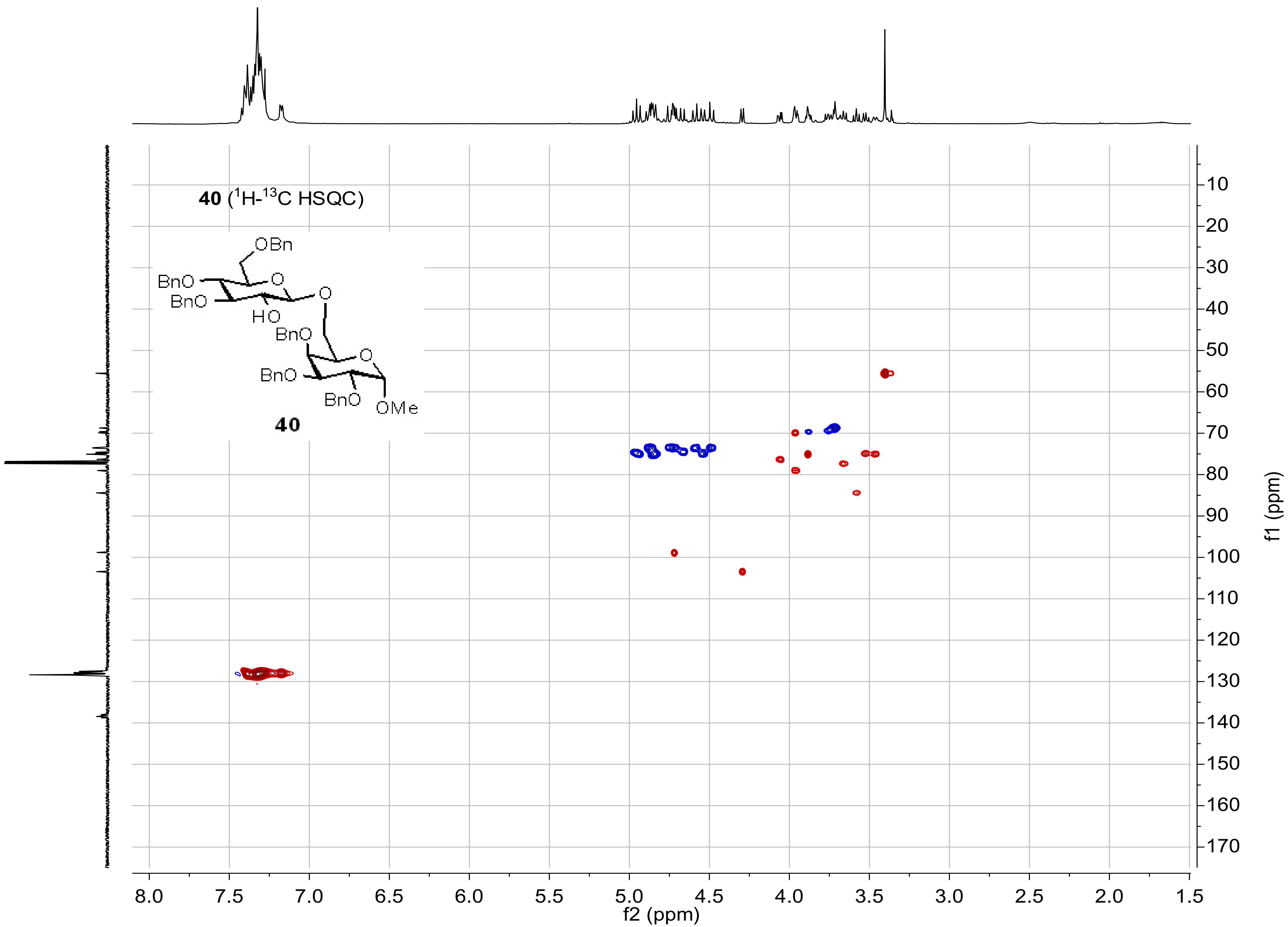

Supplementary Figure 93. ^{1}H-^{13}C HSQC Decoupled Spectrum for Compound 39

41 (^{1}H NMR, 300 MHz, $CDCl_3$)

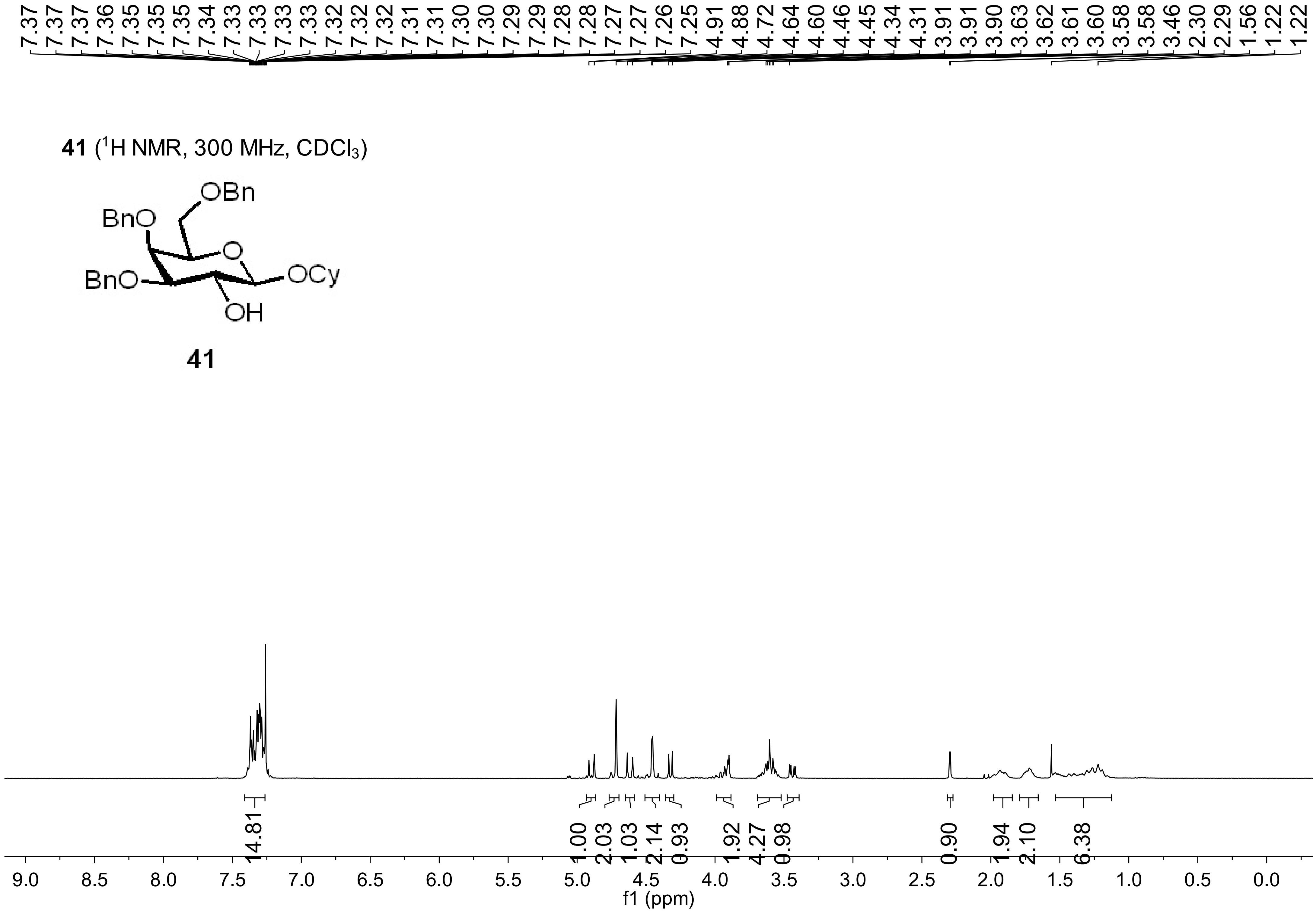

Supplementary Figure 94. ^{1}H NMR Spectrum for Compound 41

41 (^{13}C NMR, 75 MHz, $CDCl_3$)

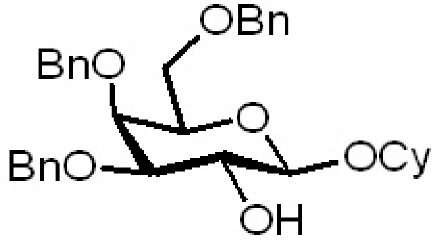

41

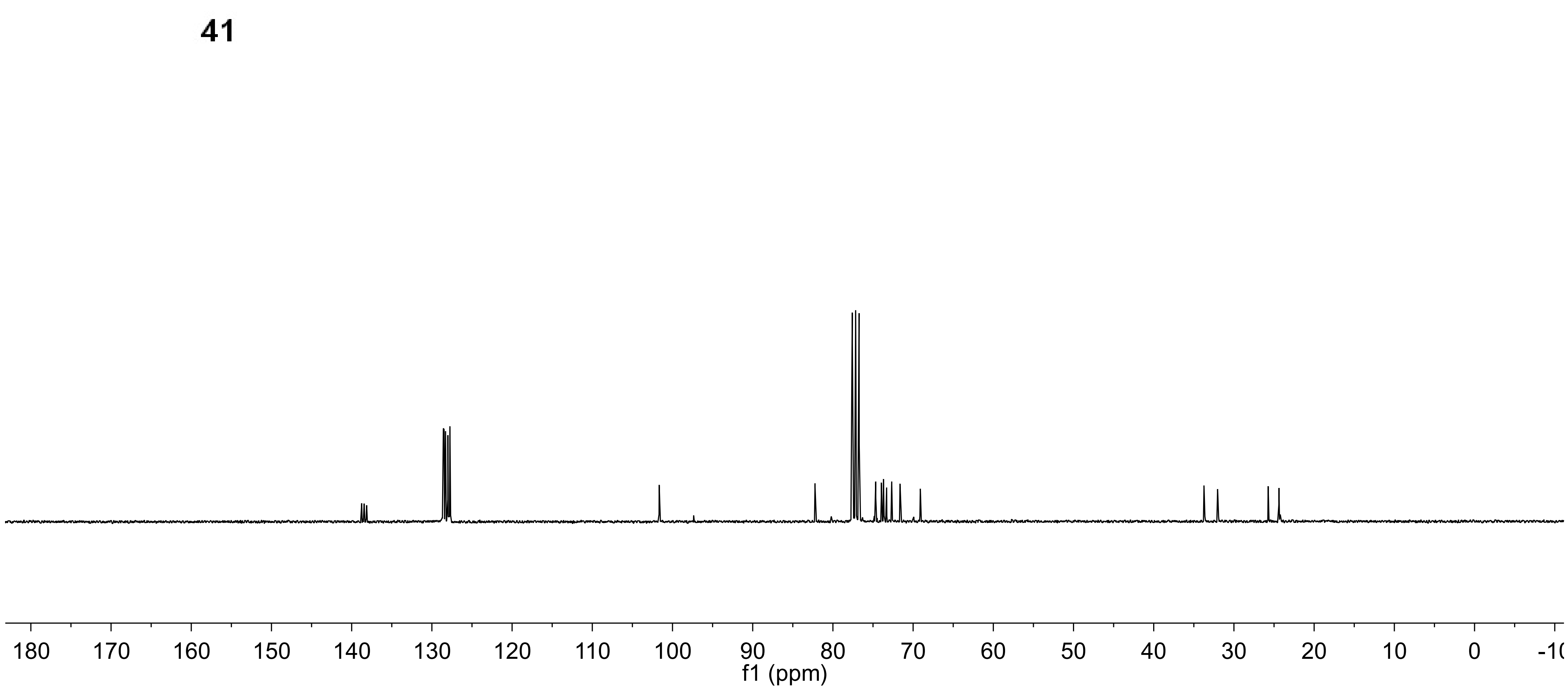

Supplementary Figure 95. ^{13}C NMR Spectrum for Compound 41

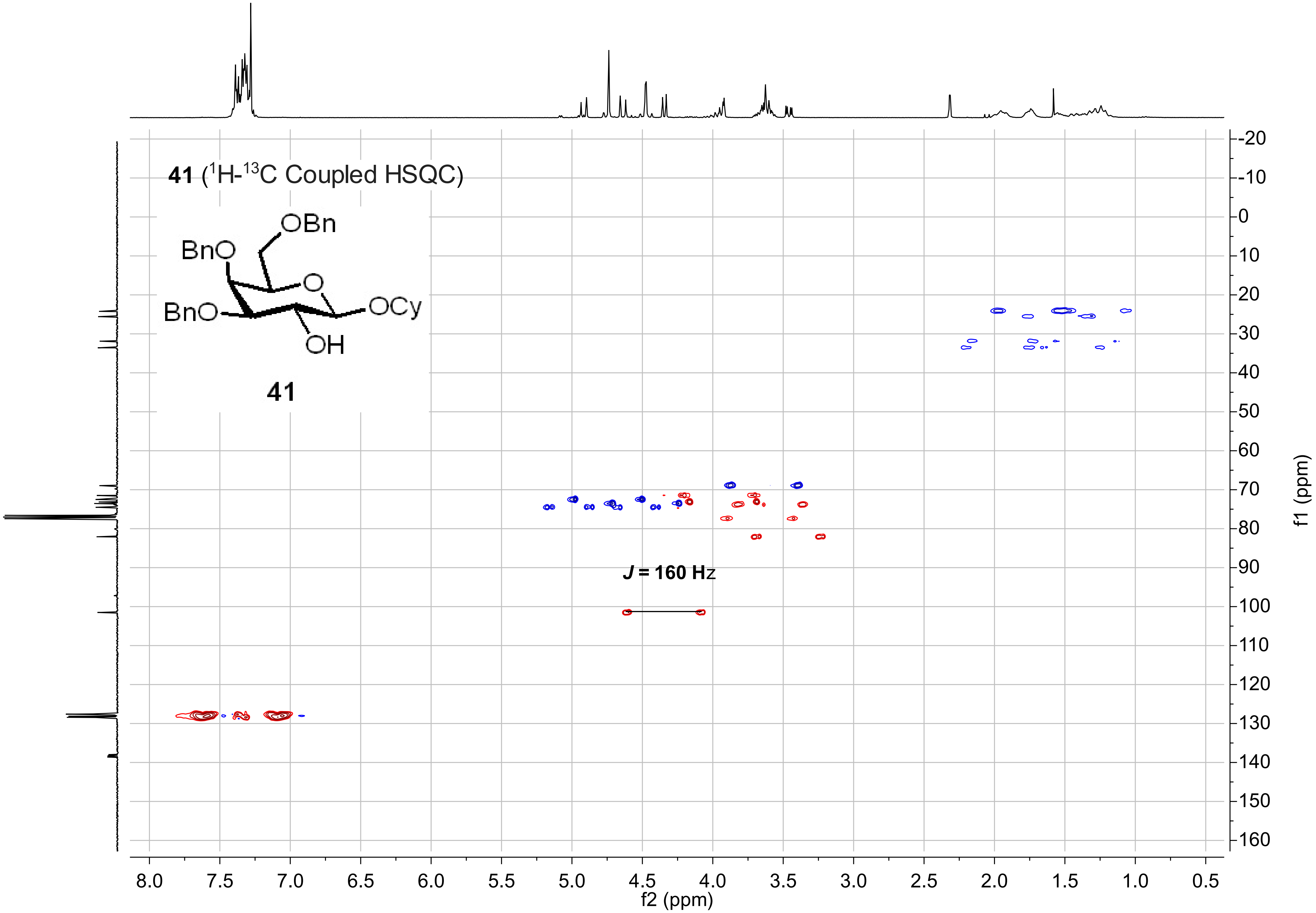

Supplementary Figure 96. **^{1}H-^{13}C HSQC Coupled Spectrum for Compound 41**

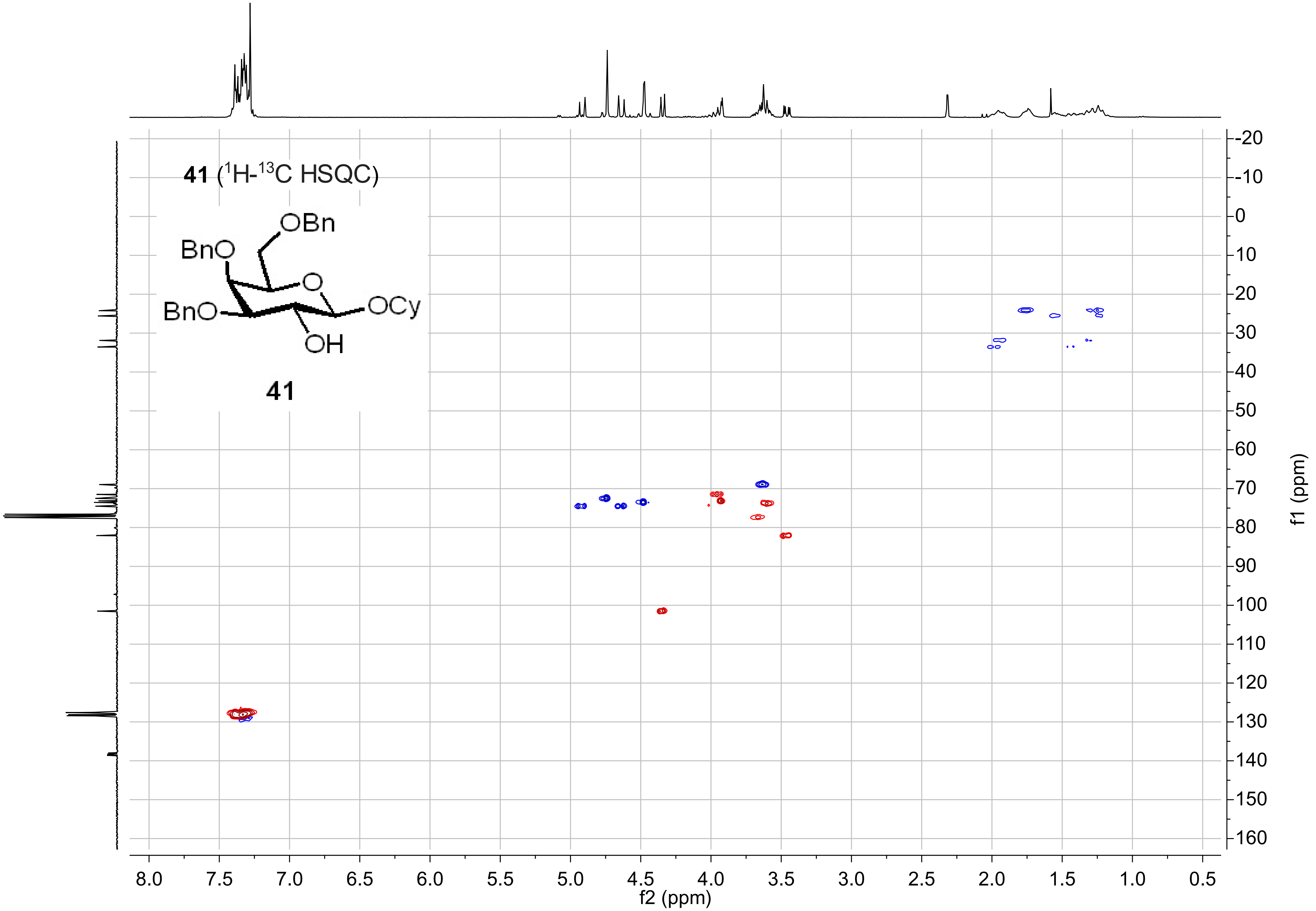

Supplementary Figure 97. ^{1}H-^{13}C HSQC Decoupled Spectrum for Compound 41

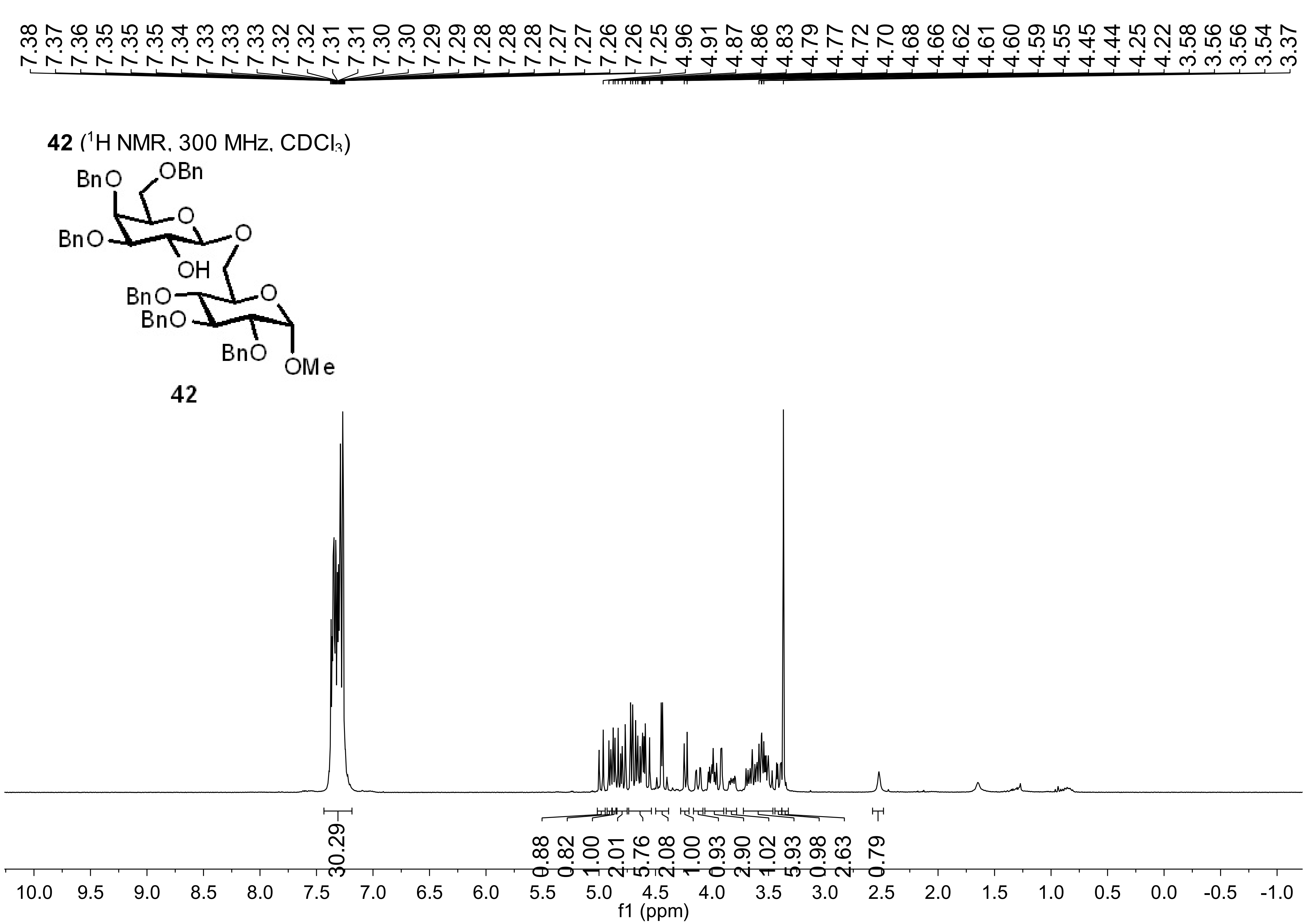

Supplementary Figure 98. ^{1}H NMR Spectrum for Compound 42

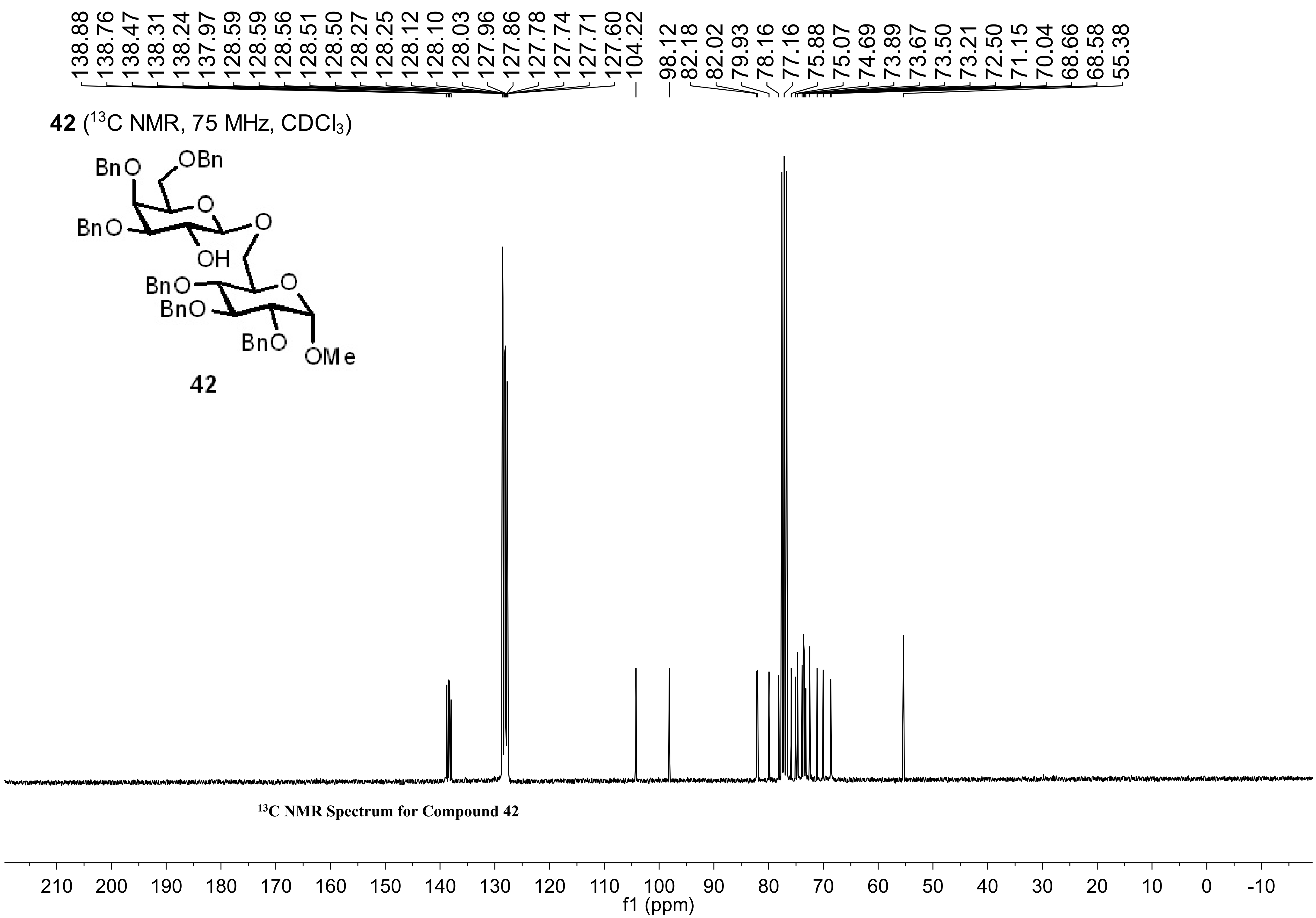

^{13}C NMR Spectrum for Compound 42

Supplementary Figure 99.

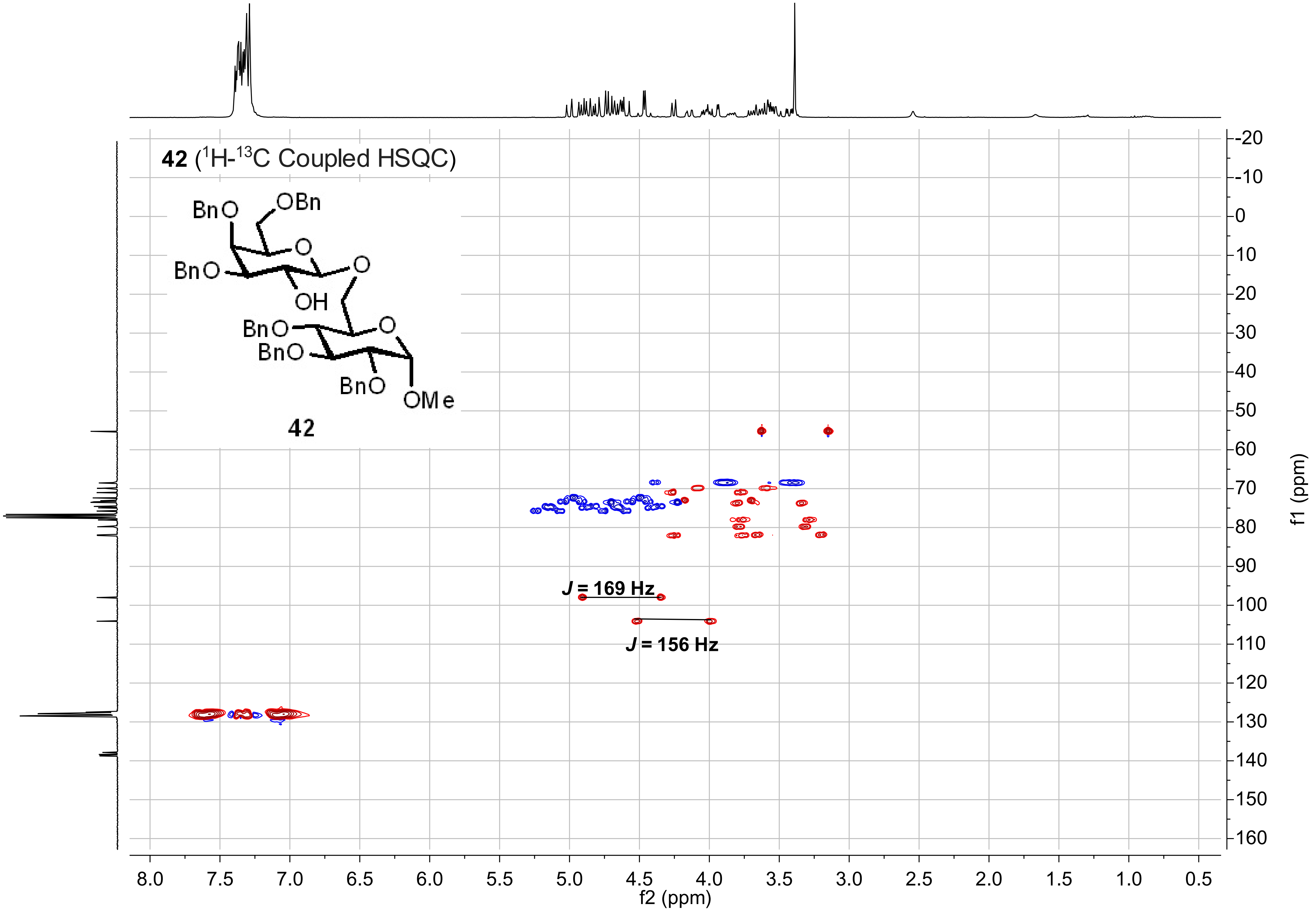

Supplementary Figure 100. ^{1}H-^{13}C **HSQC Coupled Spectrum for Compound 42**

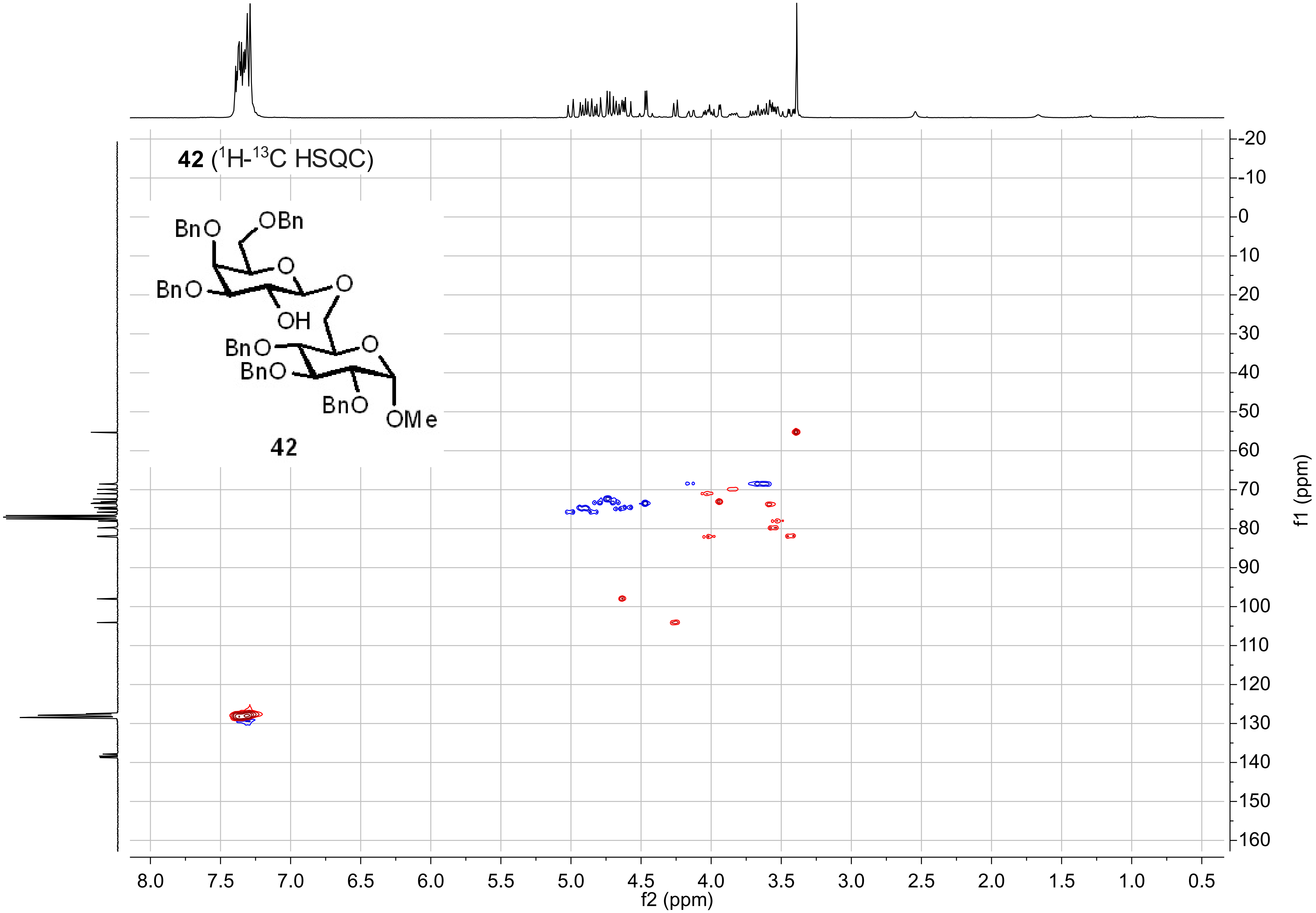

Supplementary Figure 101. **^{1}H-^{13}C HSQC Decoupled Spectrum for Compound 42**

43 (^{1}H NMR, 300 MHz, $CDCl_3$)

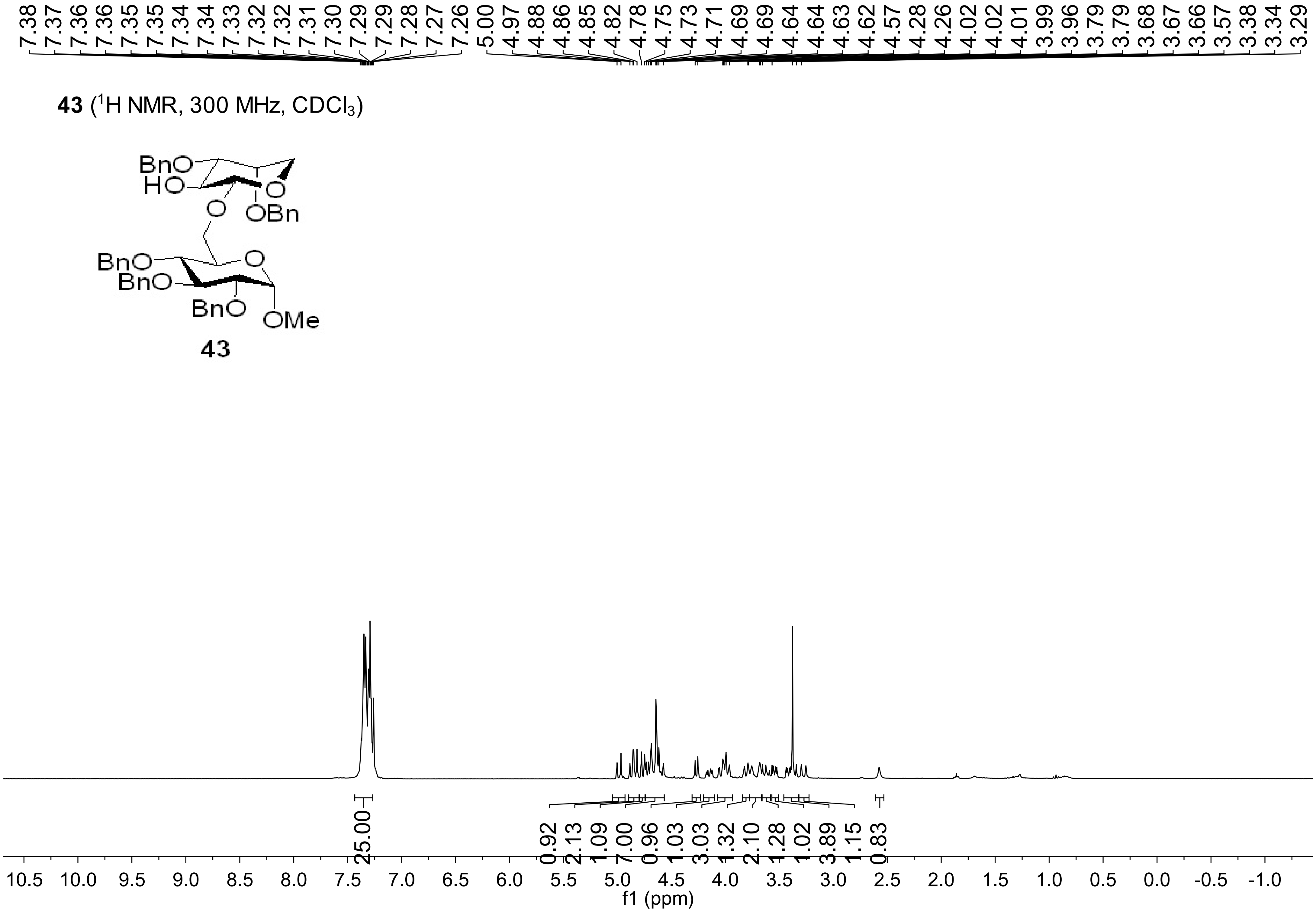

Supplementary Figure 102. **^{1}H NMR Spectrum for Compound 43**

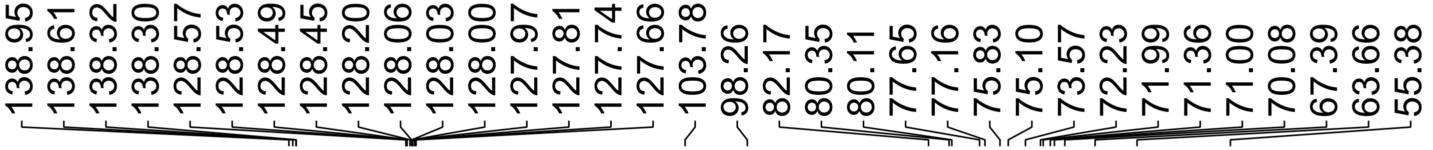

43 (^{13}C NMR, 75 MHz, $CDCl_3$)

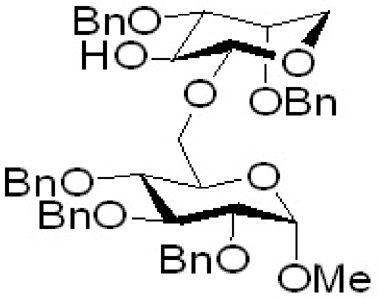

43

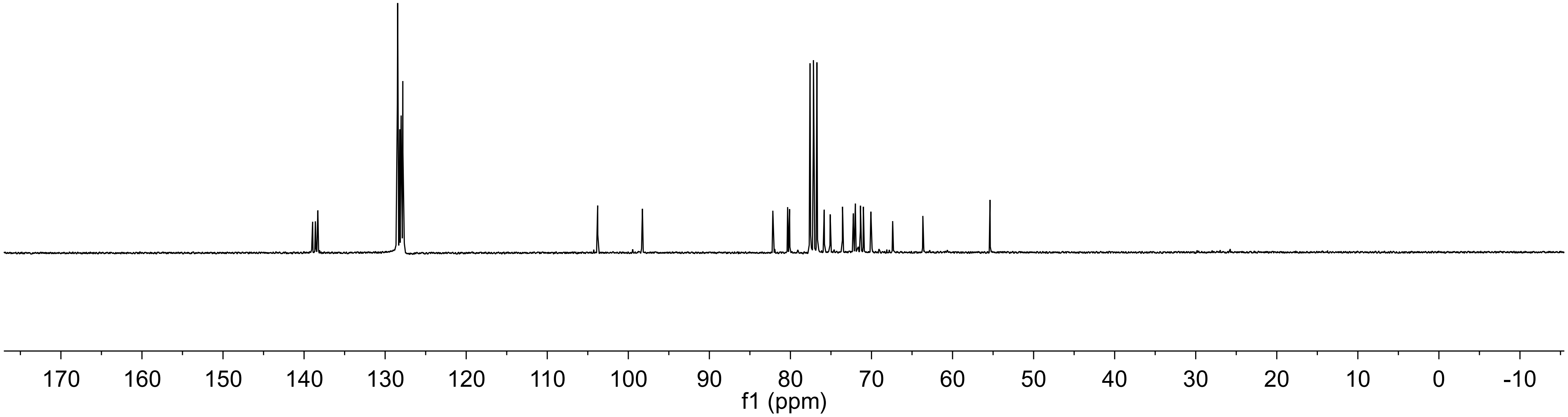

Supplementary Figure 103. ^{13}C NMR Spectrum for Compound 43

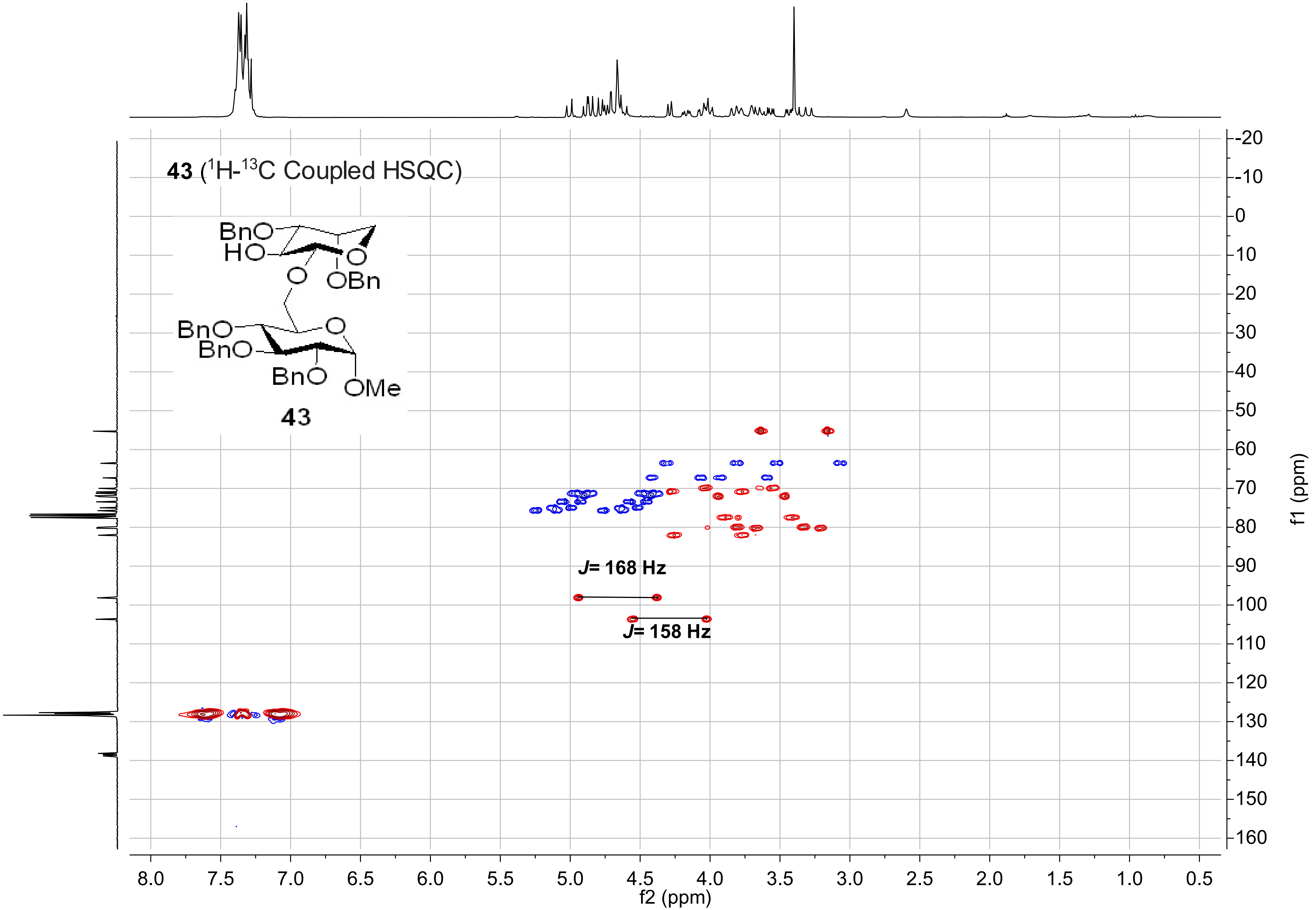

Supplementary Figure 104. ^{1}H-^{13}C HSQC Coupled Spectrum for Compound 43

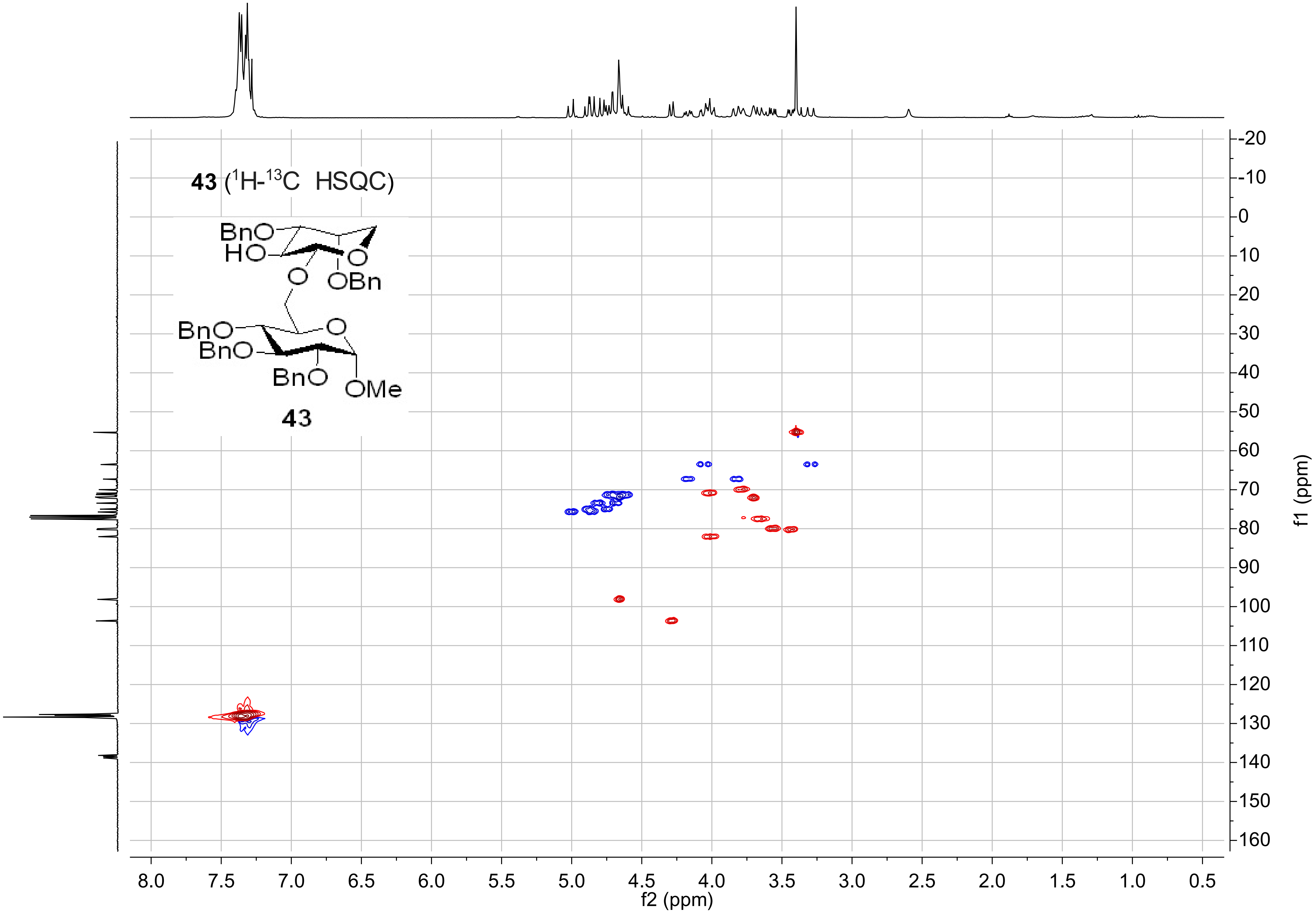

Supplementary Figure 105. ^{1}H-^{13}C **HSQC Decoupled Spectrum for Compound 43**

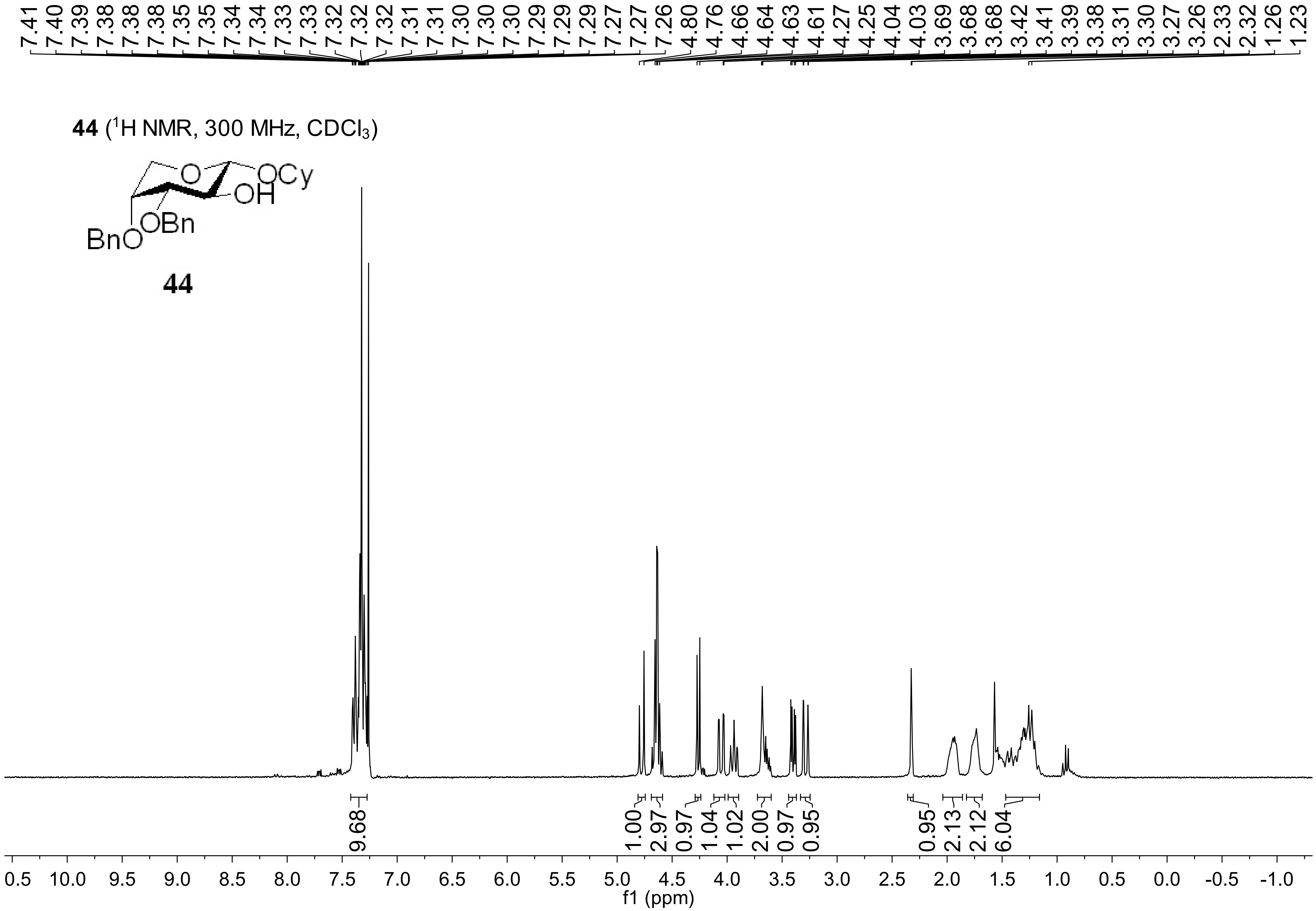

Supplementary Figure 106. **^{1}H NMR Spectrum for Compound 44**

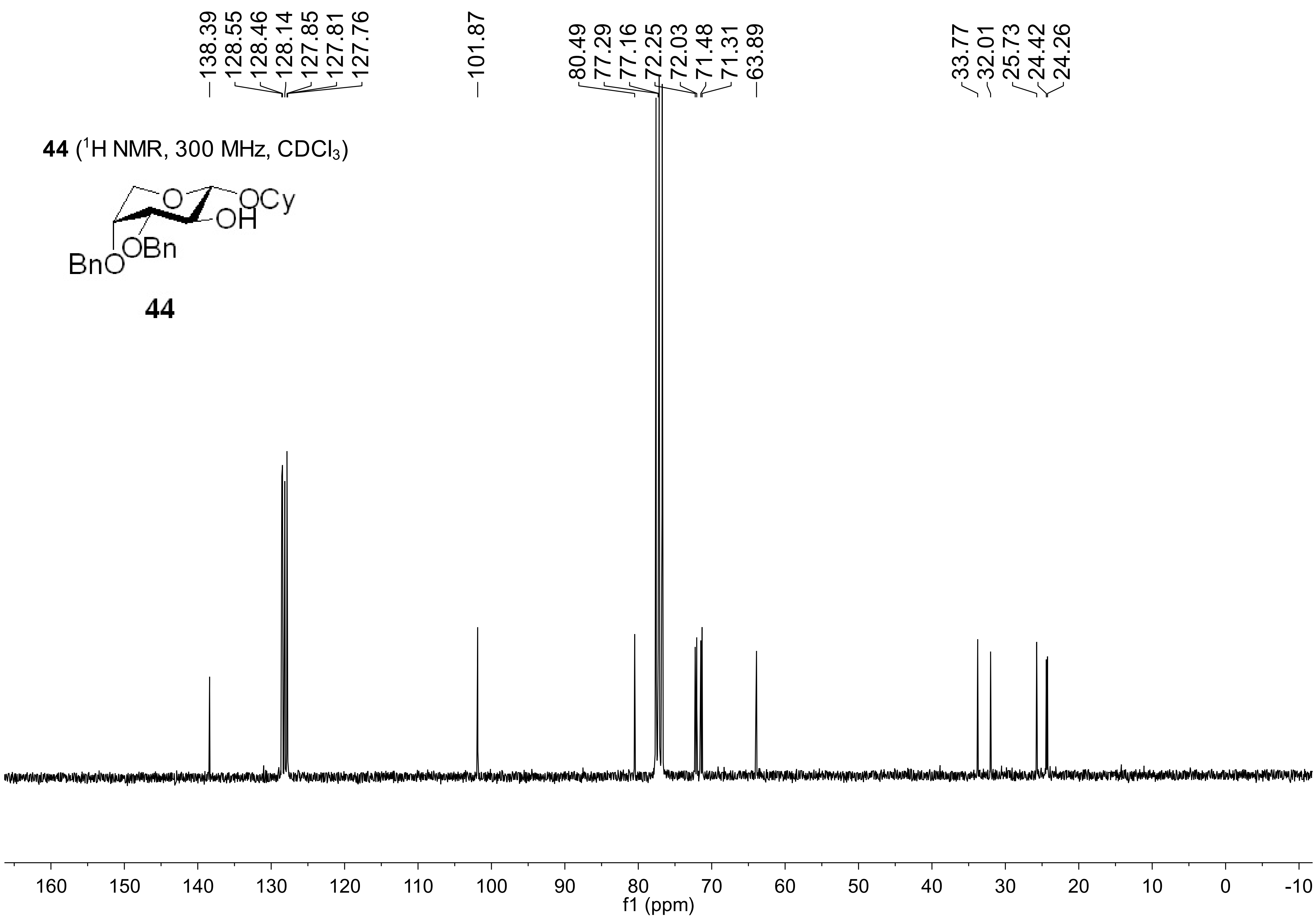

Supplementary Figure 107. ^{13}C NMR Spectrum for Compound 44

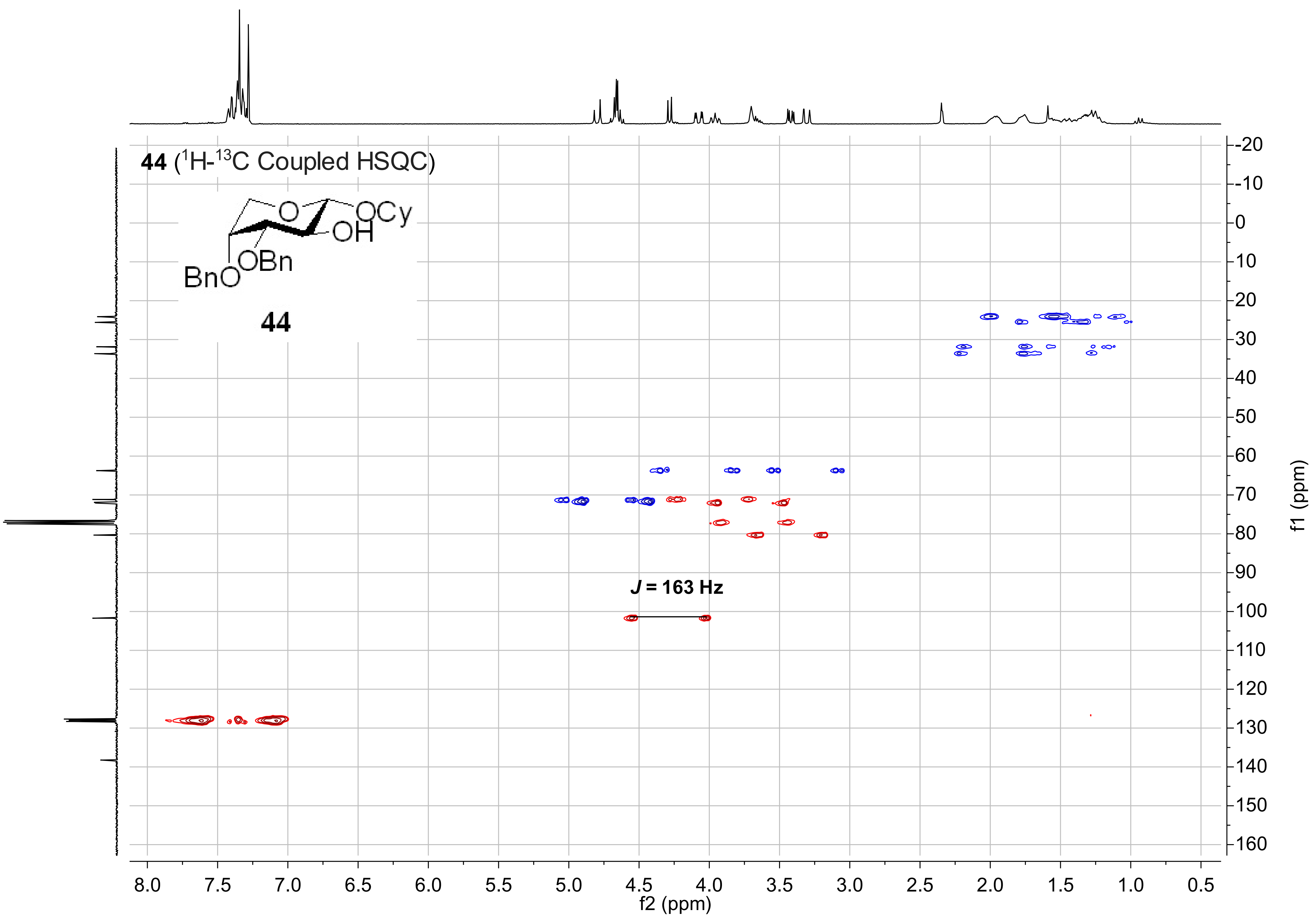

Supplementary Figure 108. **^{1}H-^{13}C HSQC Coupled Spectrum for Compound 44**

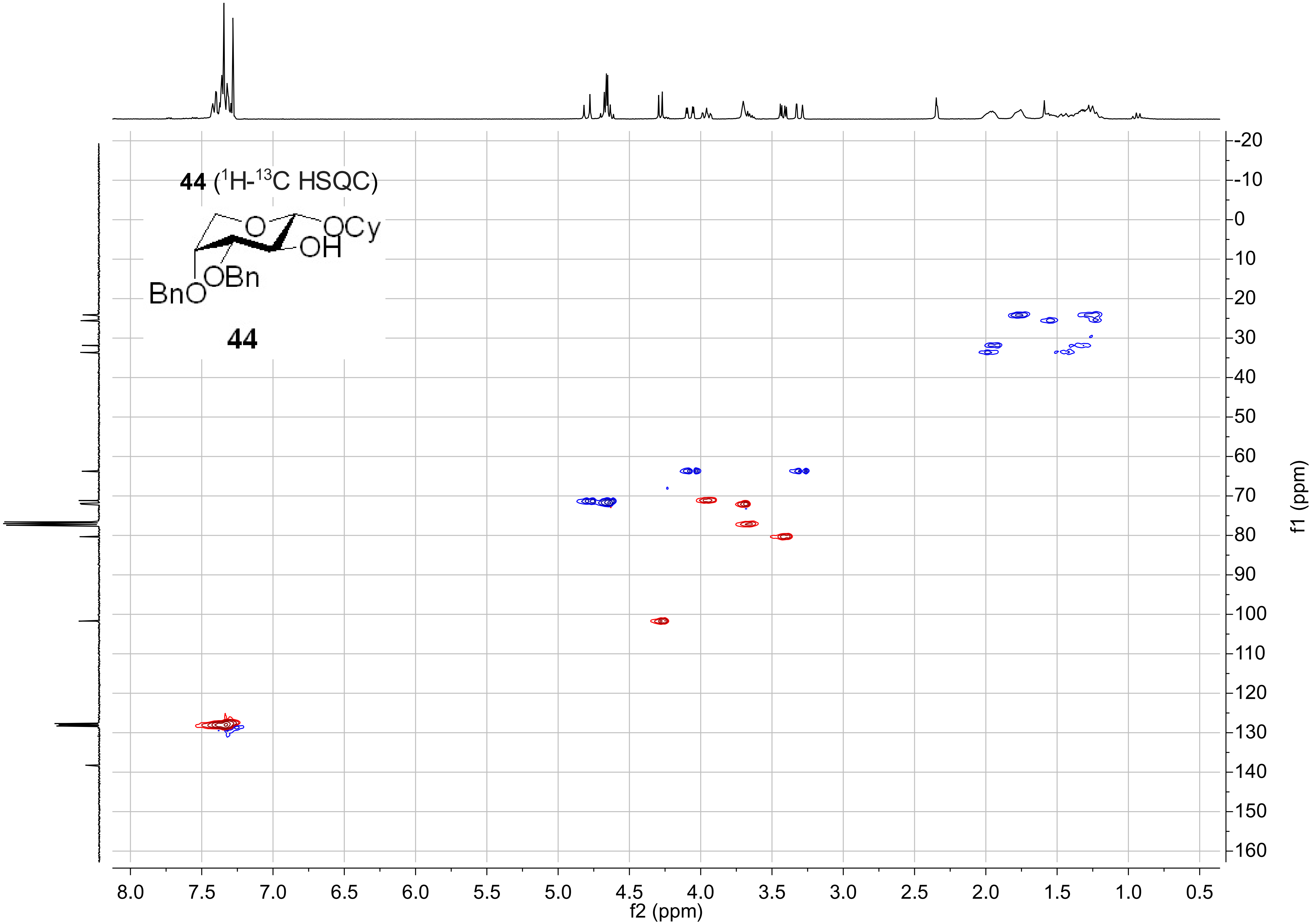

Supplementary Figure 109. ^{1}H-^{13}C **HSQC Decoupled Spectrum for Compound 44**

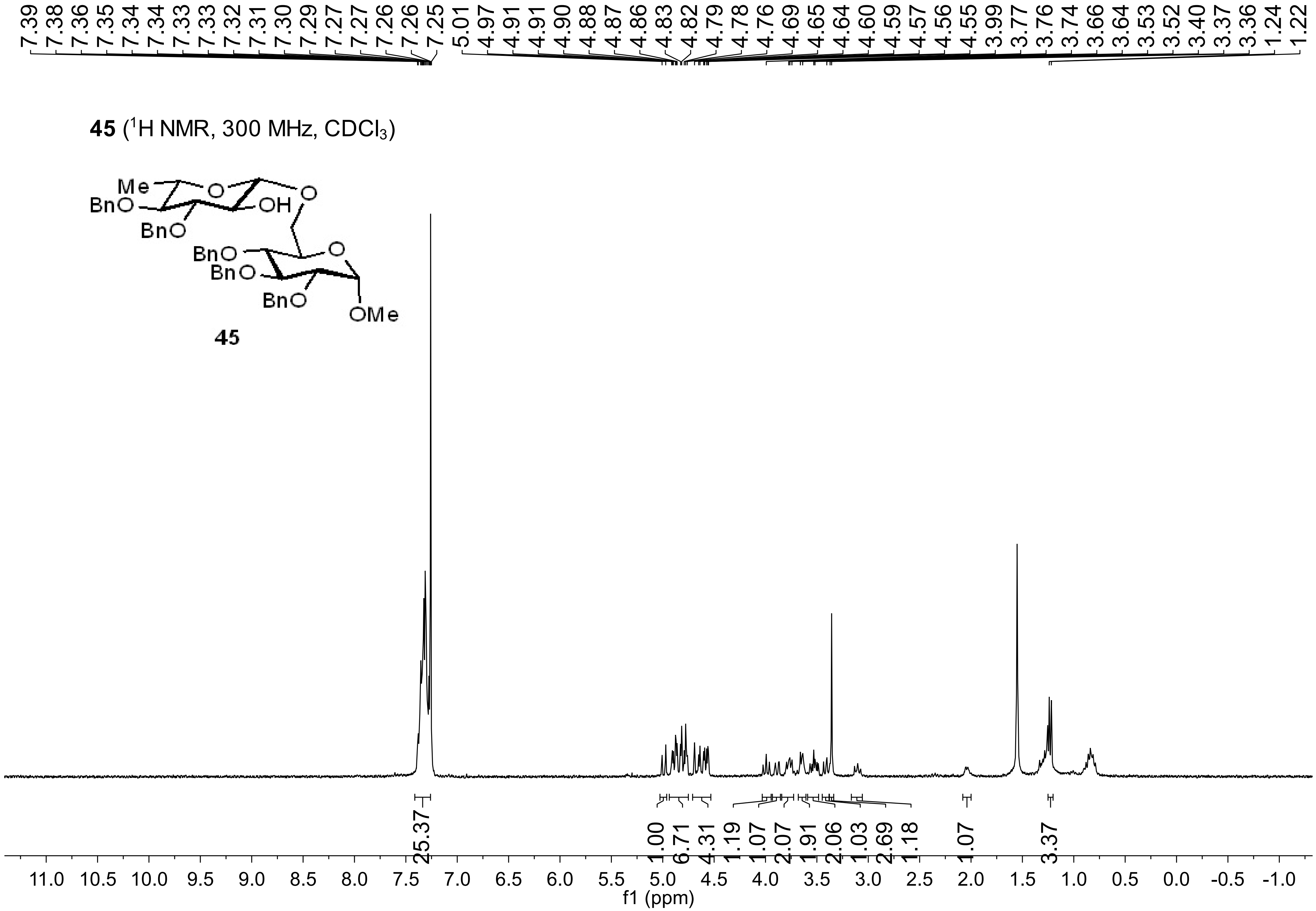

Supplementary Figure 110. ^{1}H NMR Spectrum for Compound 45

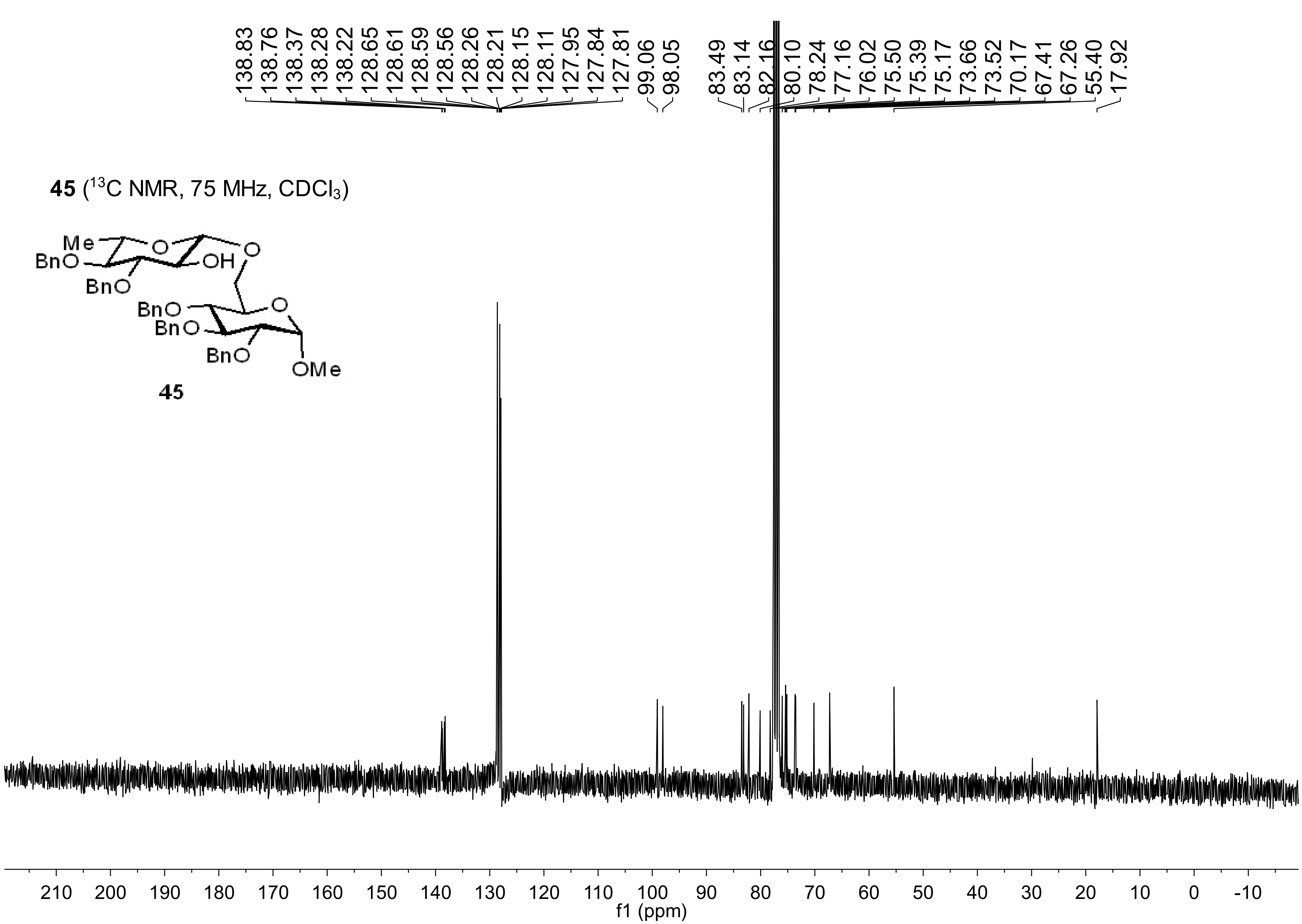

Supplementary Figure 111. ^{13}C NMR Spectrum for Compound 45

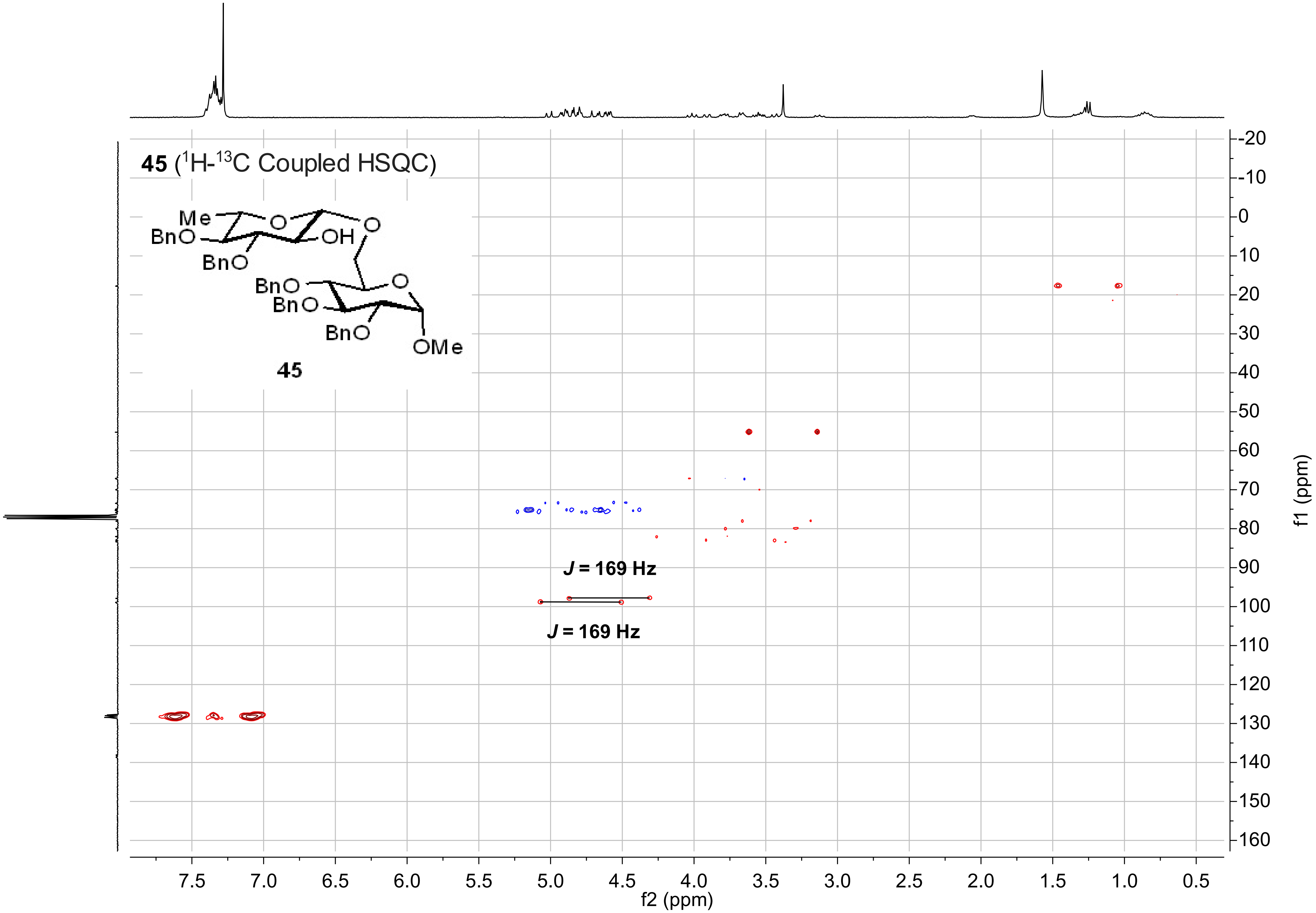

Supplementary Figure 112. ^{1}H-^{13}C HSQC Coupled Spectrum for Compound 45

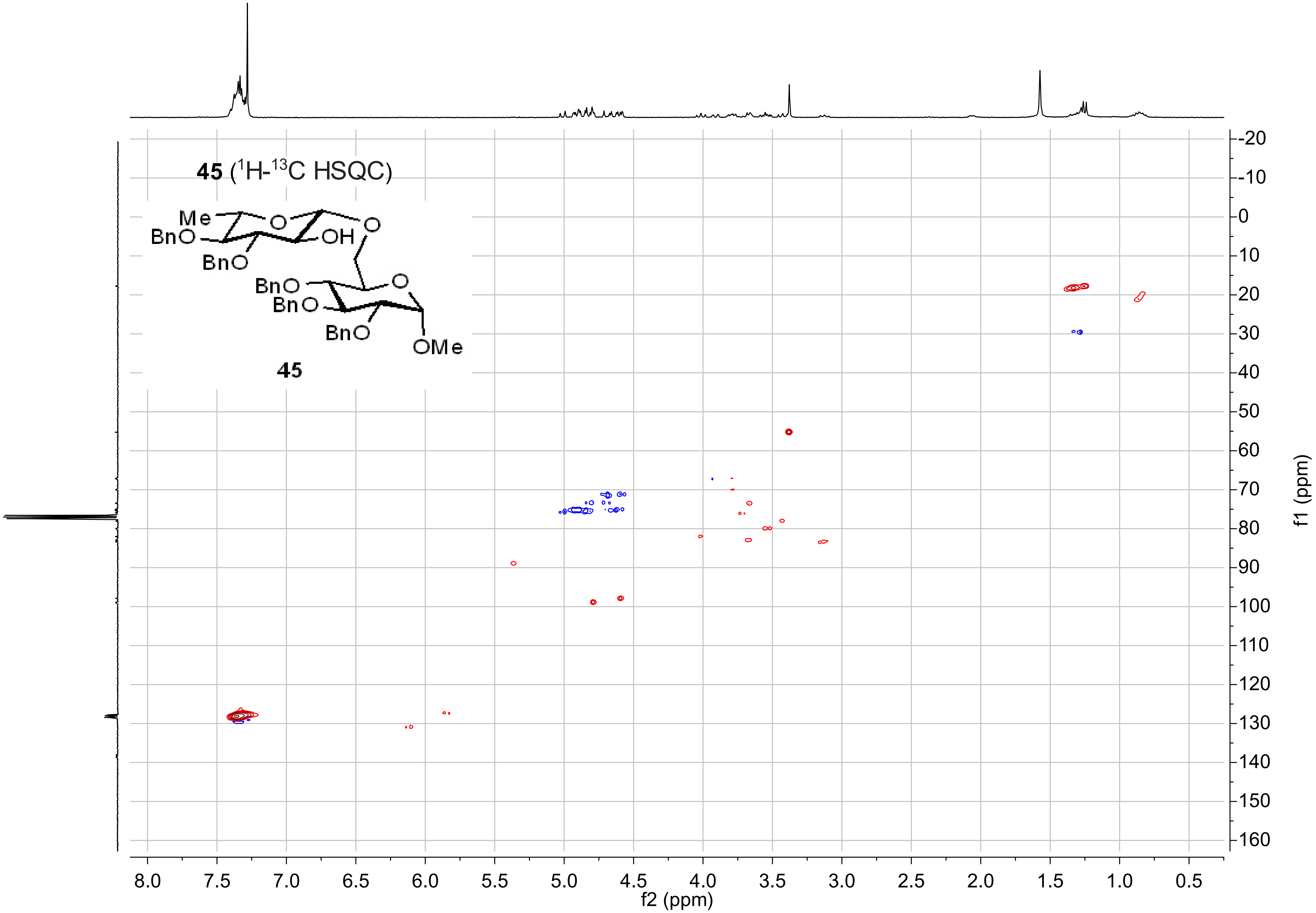

Supplementary Figure 113. ^{1}H-^{13}C HSQC Decoupled Spectrum for Compound 45

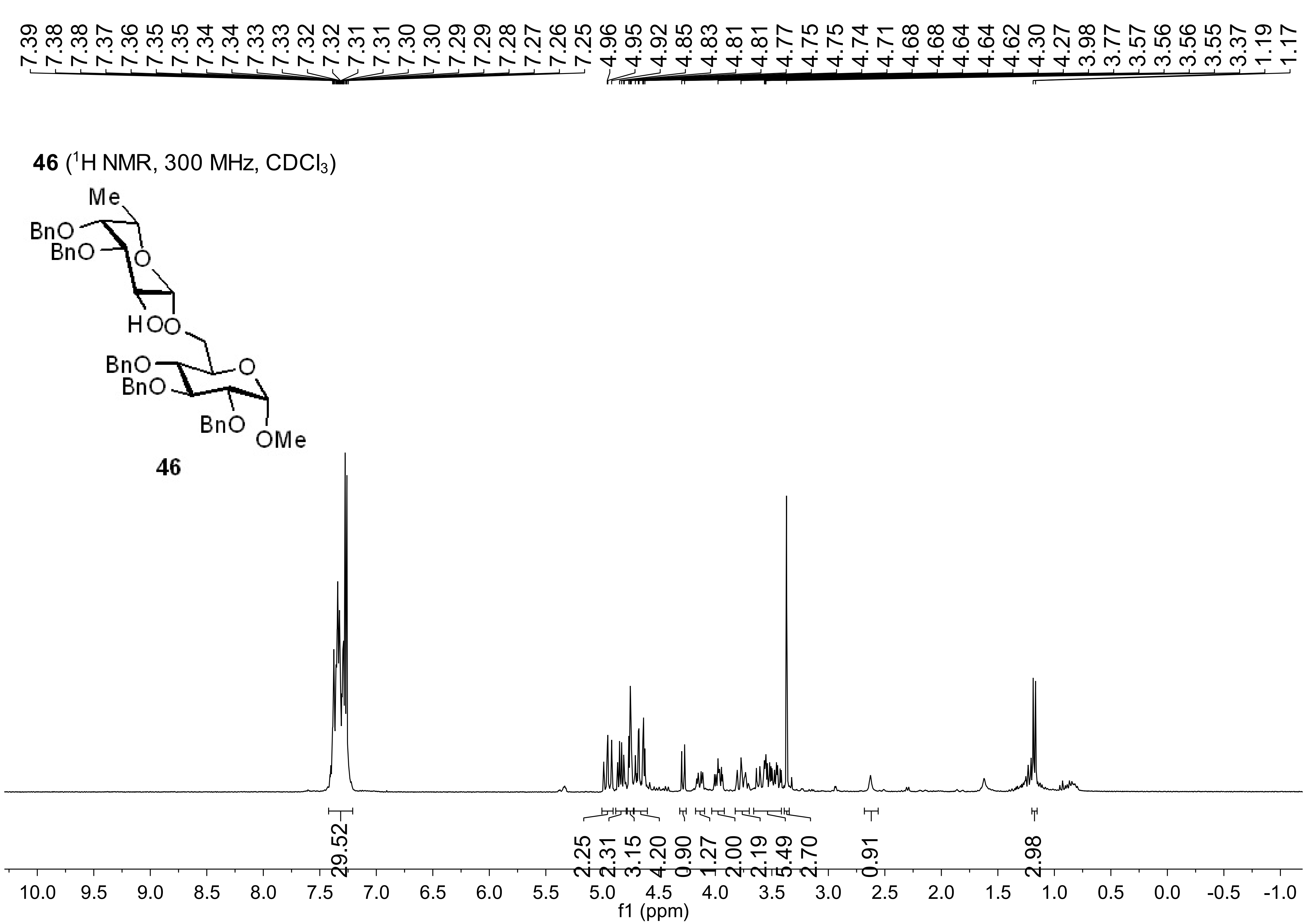

Supplementary Figure 114. ^{1}H NMR Spectrum for Compound 46

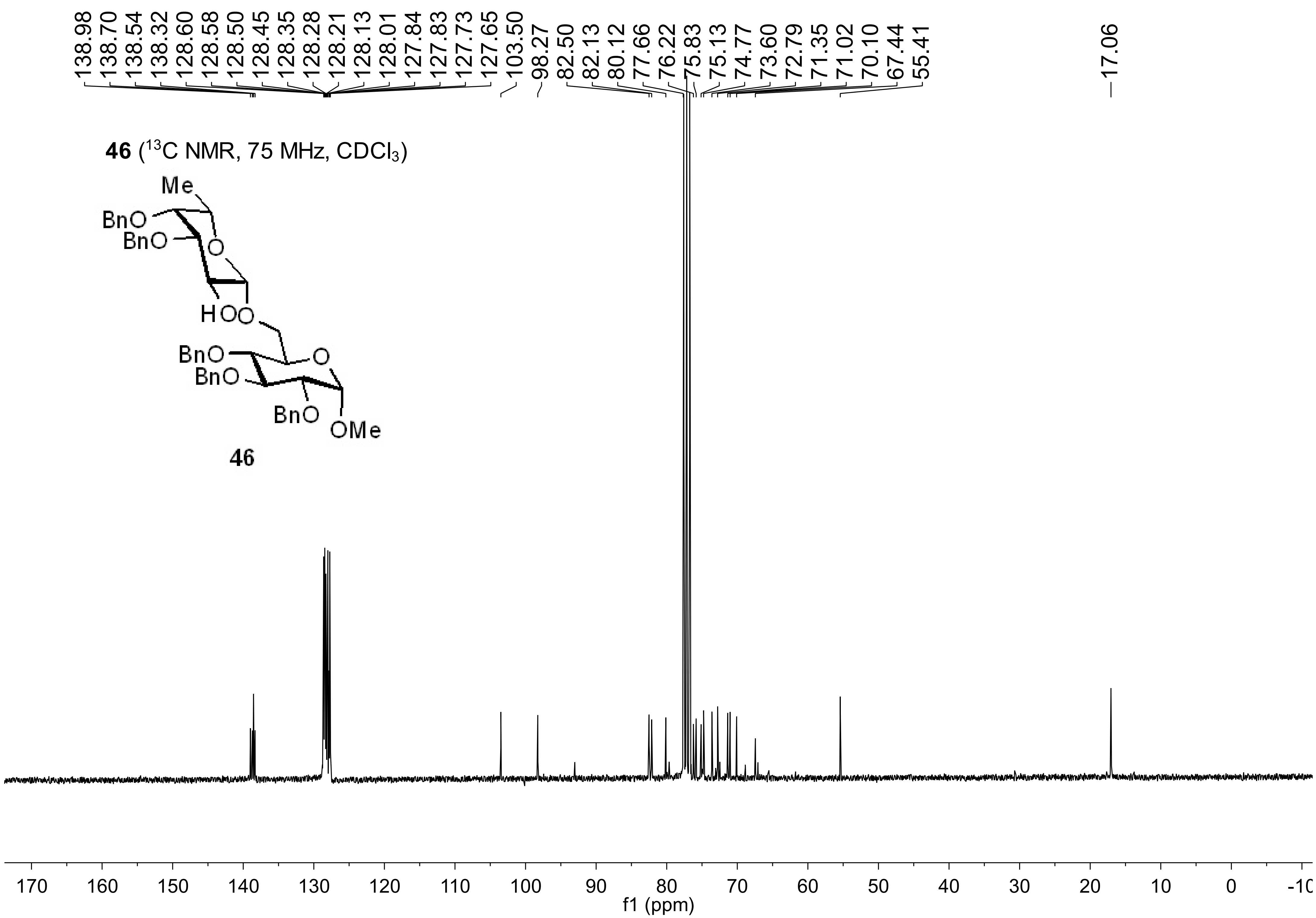

Supplementary Figure 115. ^{13}C NMR Spectrum for Compound 46

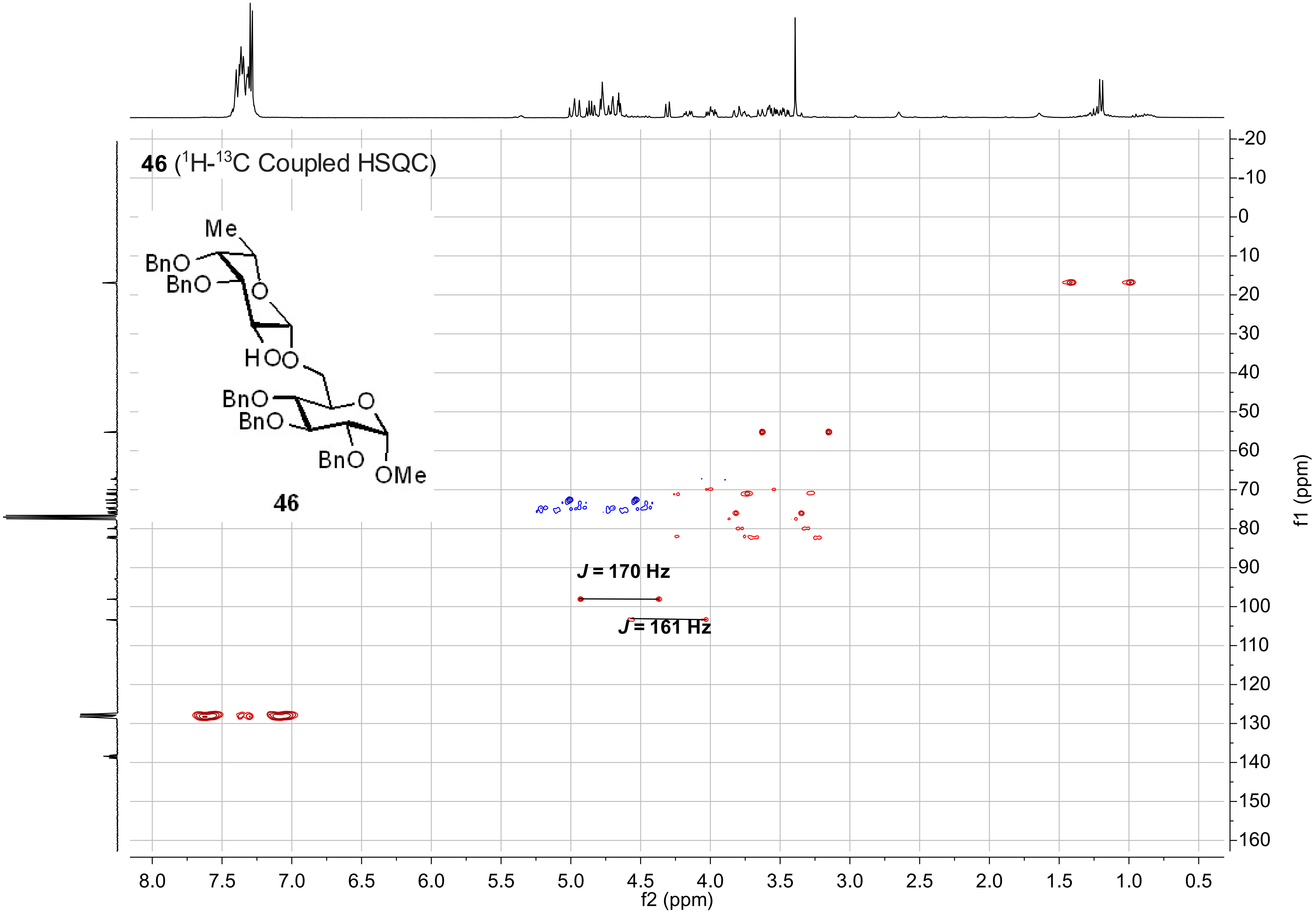

Supplementary Figure 116. ^{1}H-^{13}C HSQC Coupled Spectrum for Compound 46

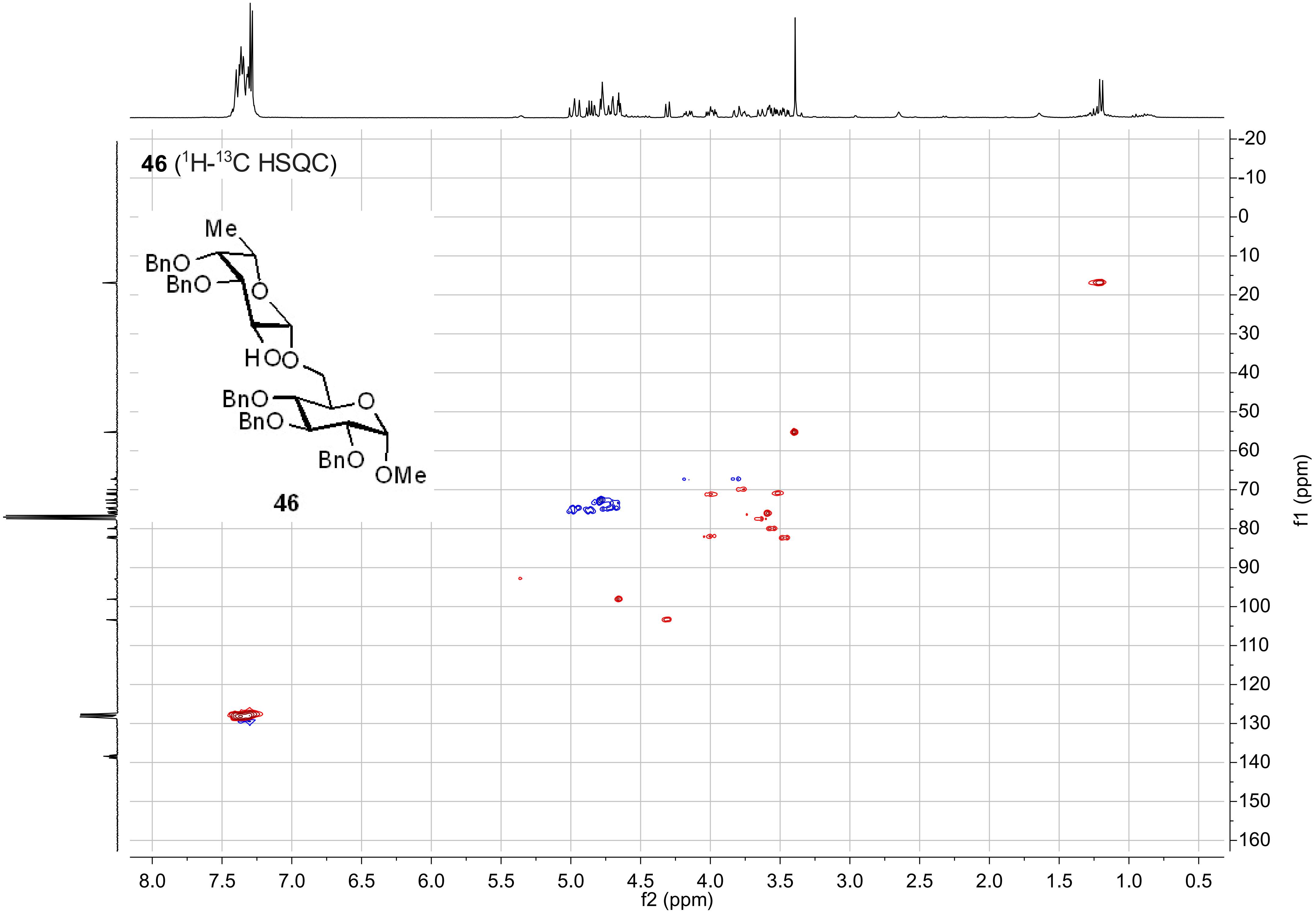

Supplementary Figure 117. **^{1}H-^{13}C HSQC Decoupled Spectrum for Compound 46**

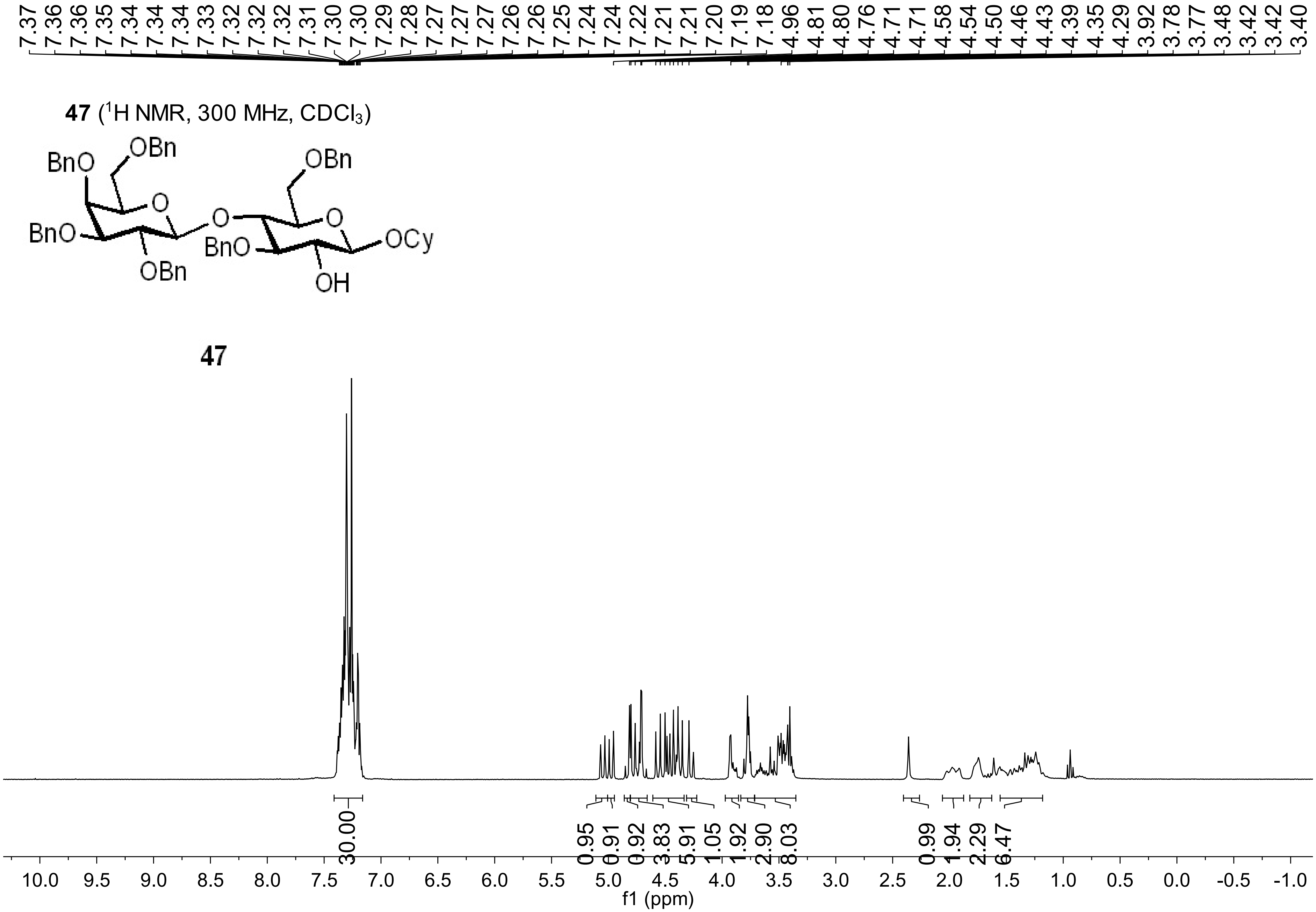

Supplementary Figure 118. ^{1}H NMR Spectrum for Compound 47

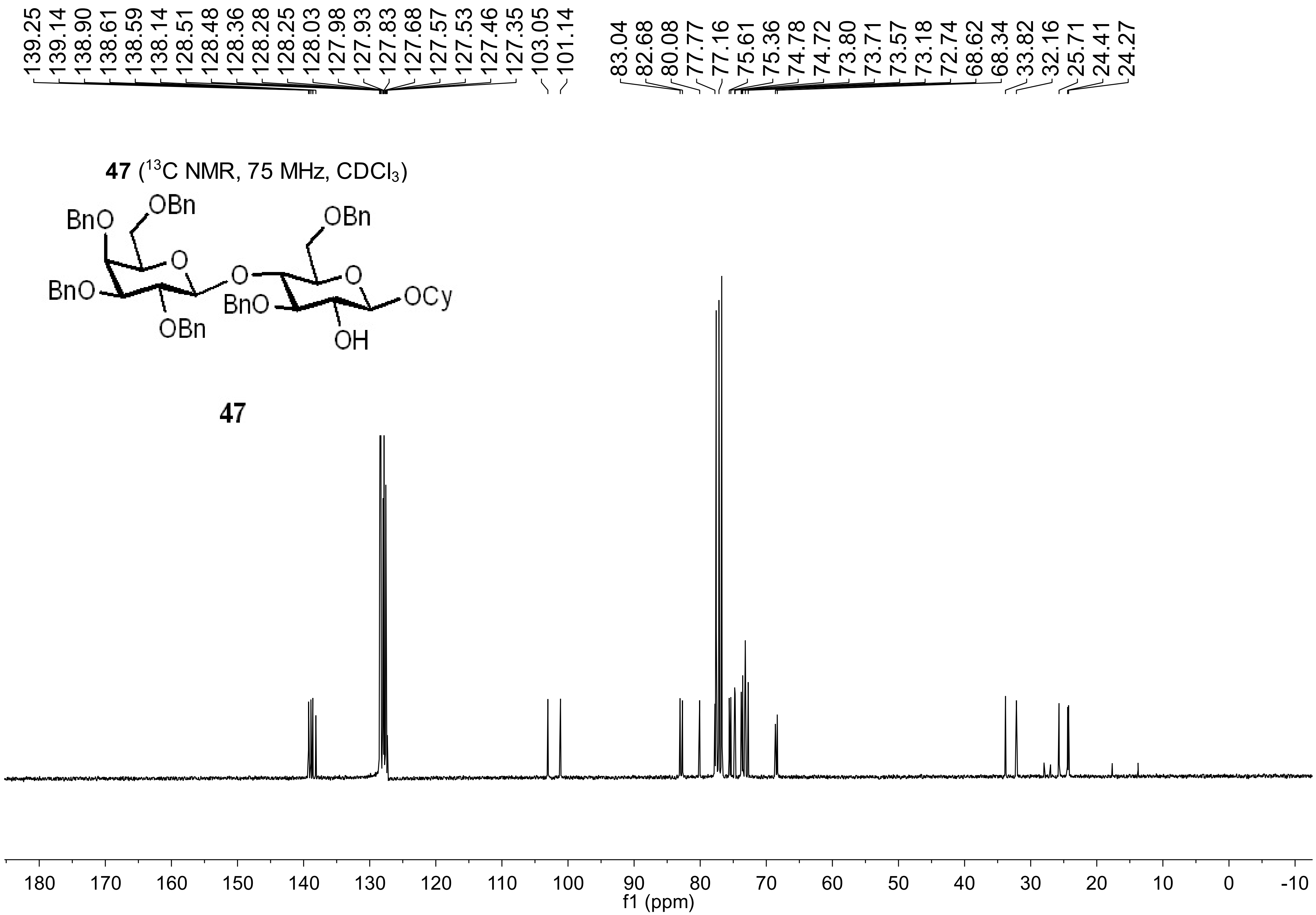

Supplementary Figure 119. ^{13}C NMR Spectrum for Compound 47

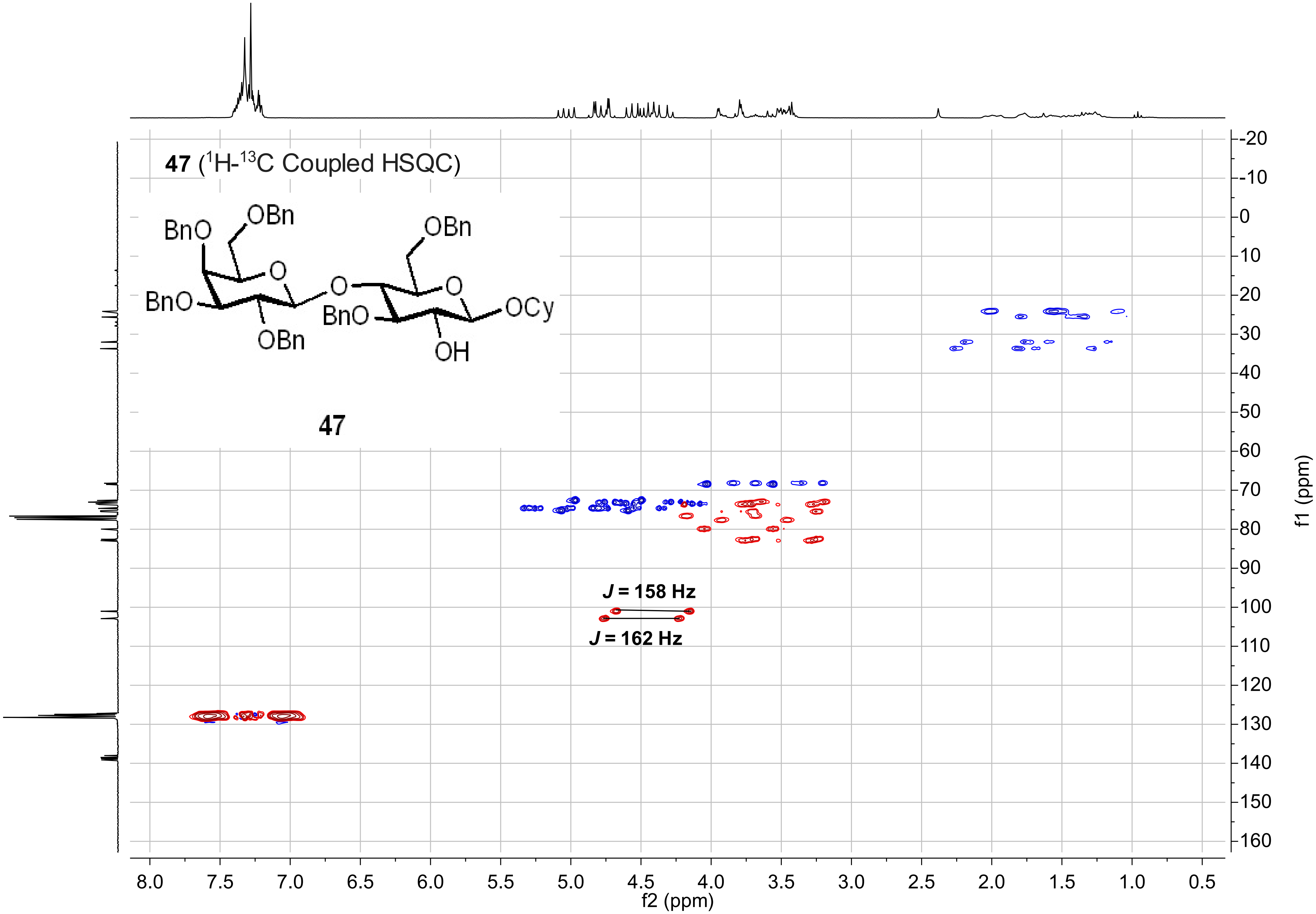

Supplementary Figure 120. ^{1}H-^{13}C **HSQC Coupled Spectrum for Compound 47**

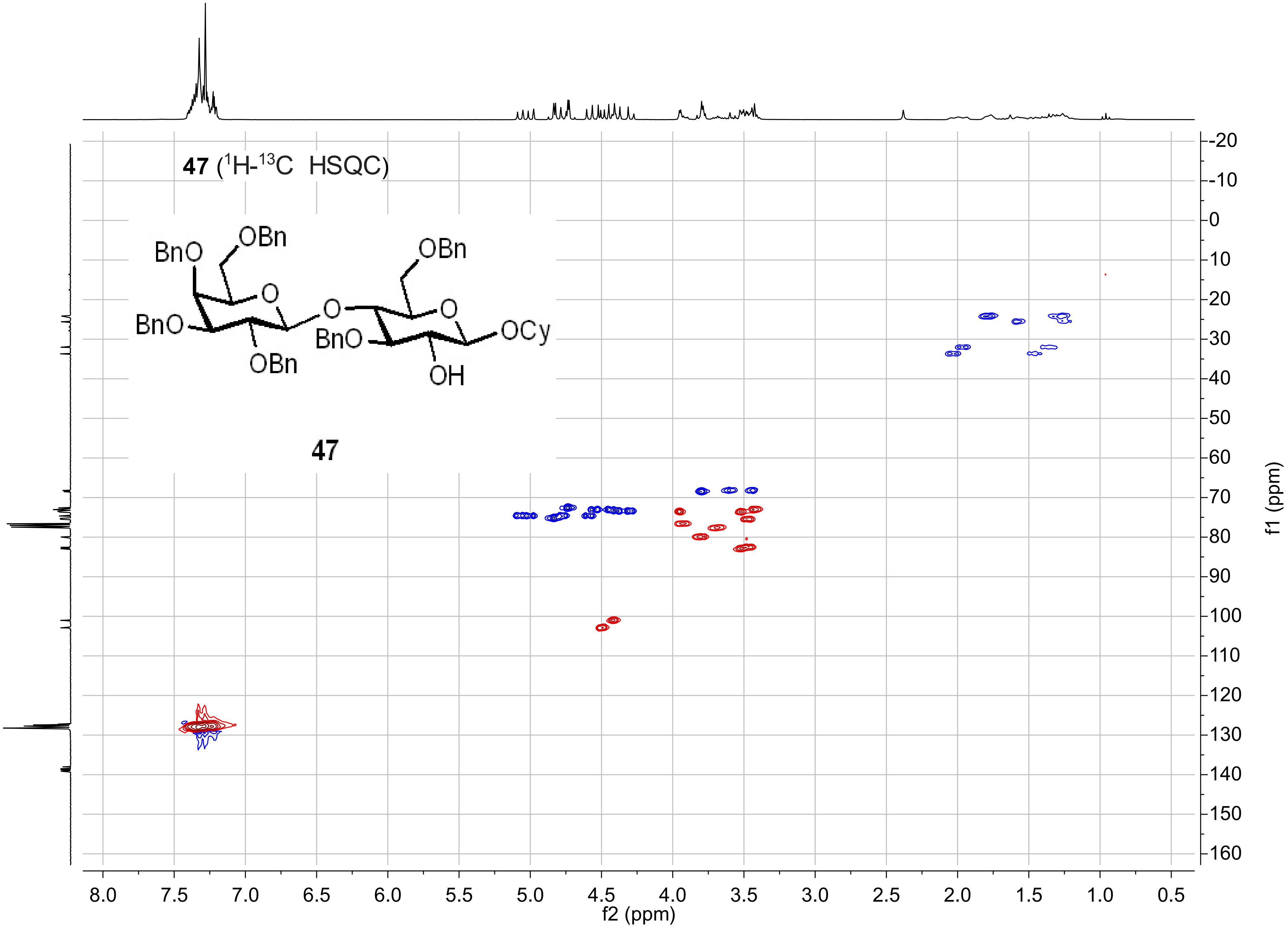

Supplementary Figure 121. ^{1}H-^{13}C **HSQC Decoupled Spectrum for Compound 47**

48 (^{1}H NMR, 300 MHz, $CDCl_3$)

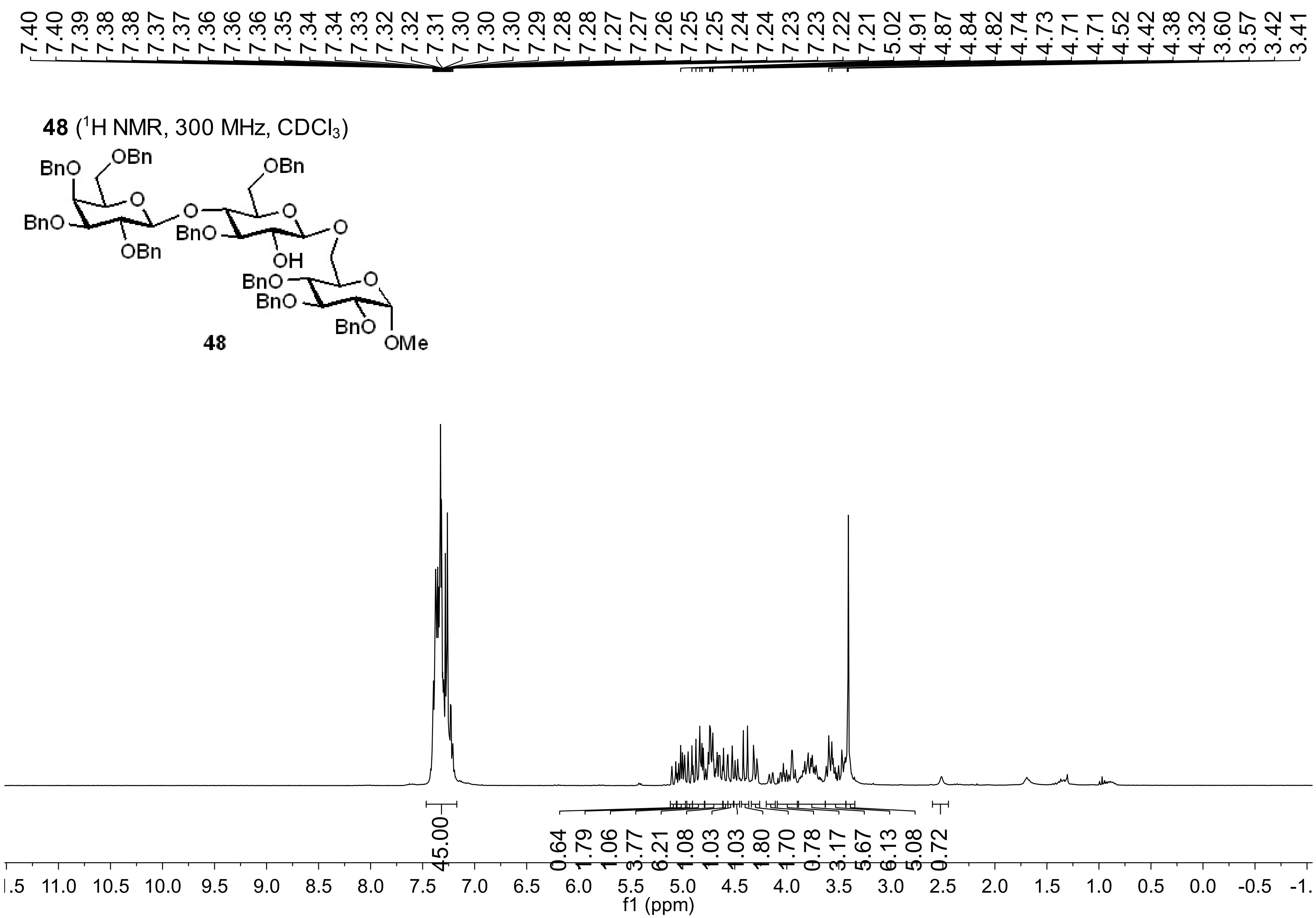

Supplementary Figure 122. ^{1}H NMR Spectrum for Compound 48

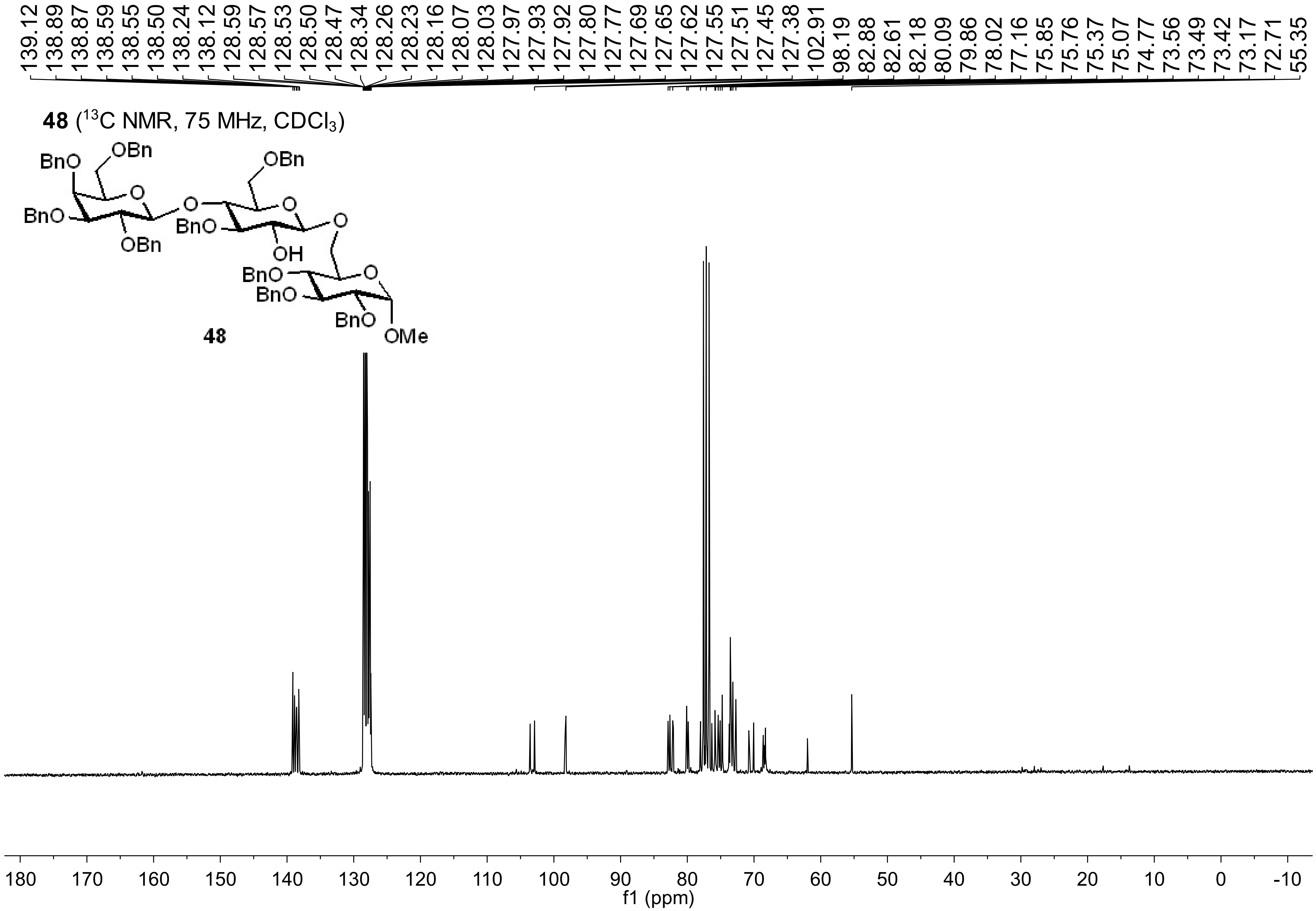

Supplementary Figure 123. ${}^{13}C$ NMR Spectrum for Compound 48

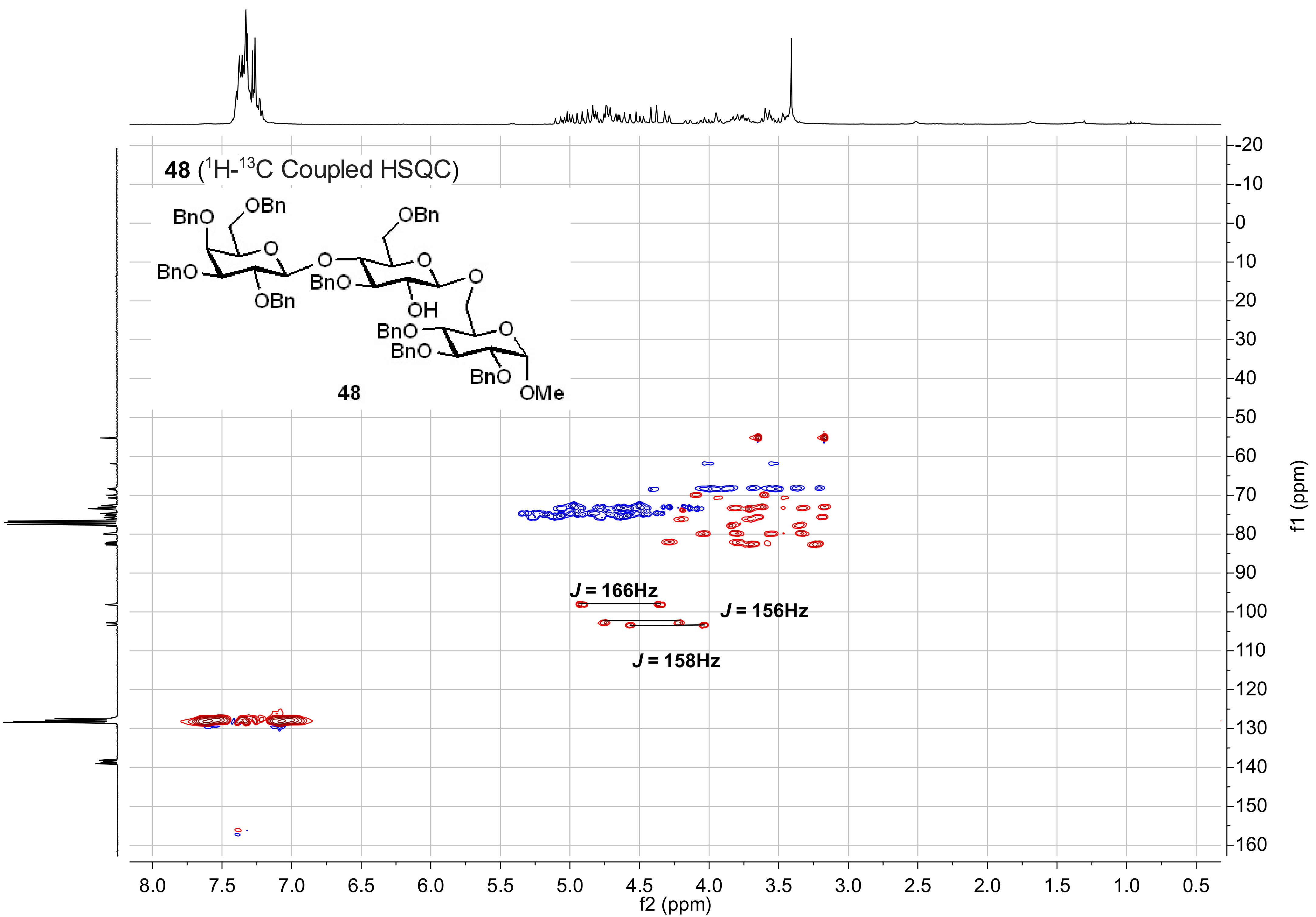

Supplementary Figure 124. ^{1}H-^{13}C HSQC Coupled Spectrum for Compound 48

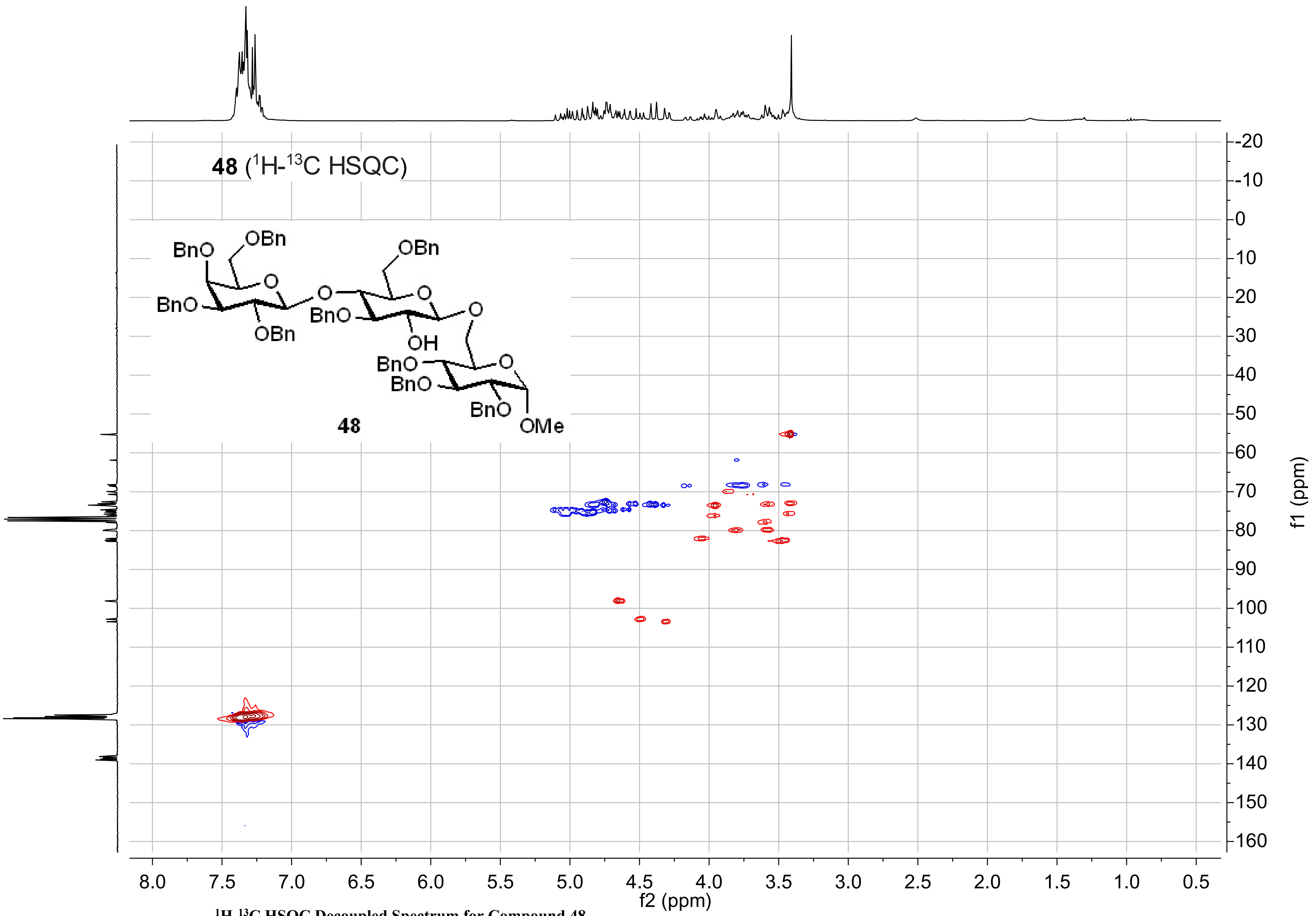

^{1}H-^{13}C HSQC Decoupled Spectrum for Compound 48

Supplementary Figure 125.

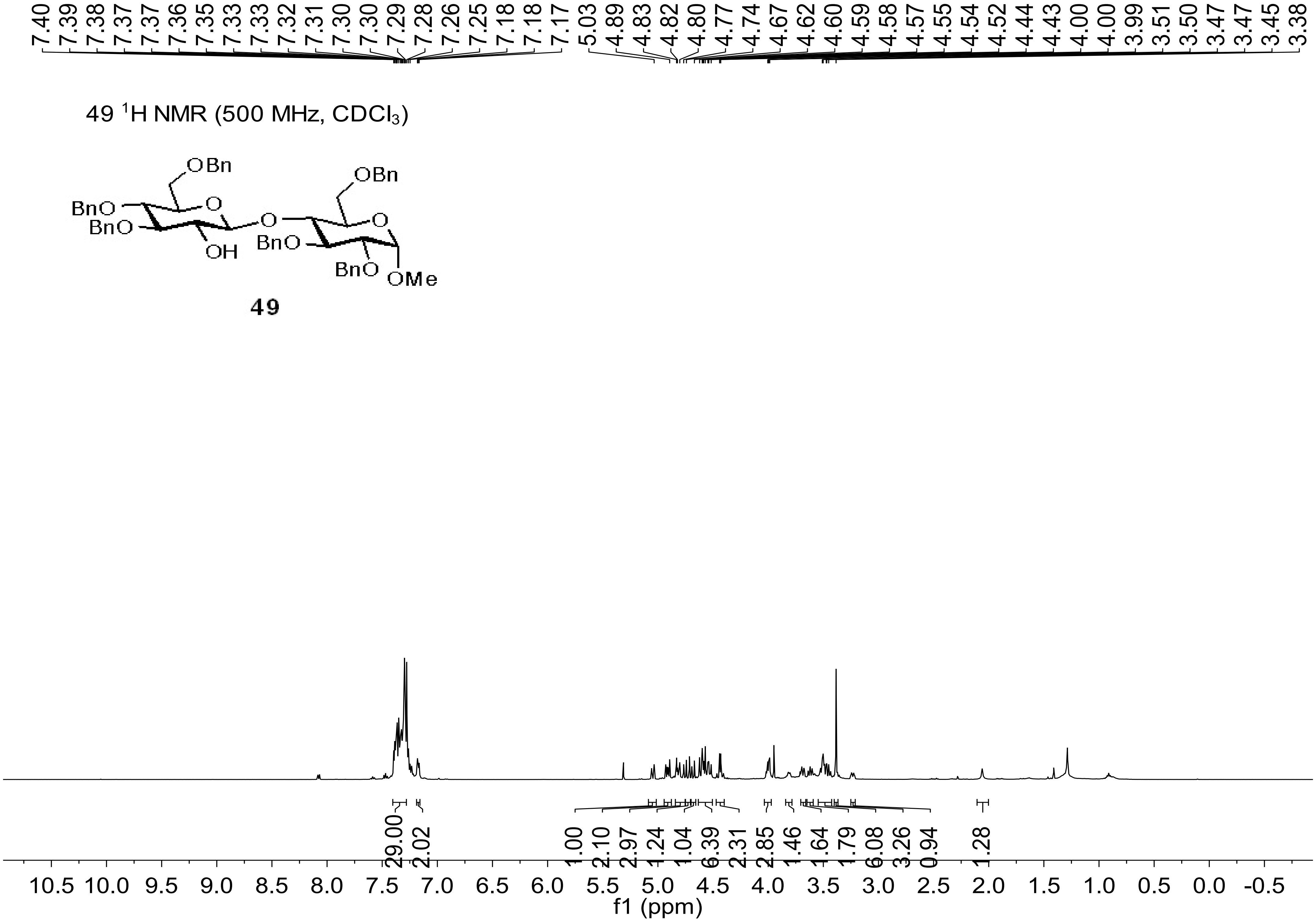

Supplementary Figure 126. **¹H NMR Spectrum for Compound 49**

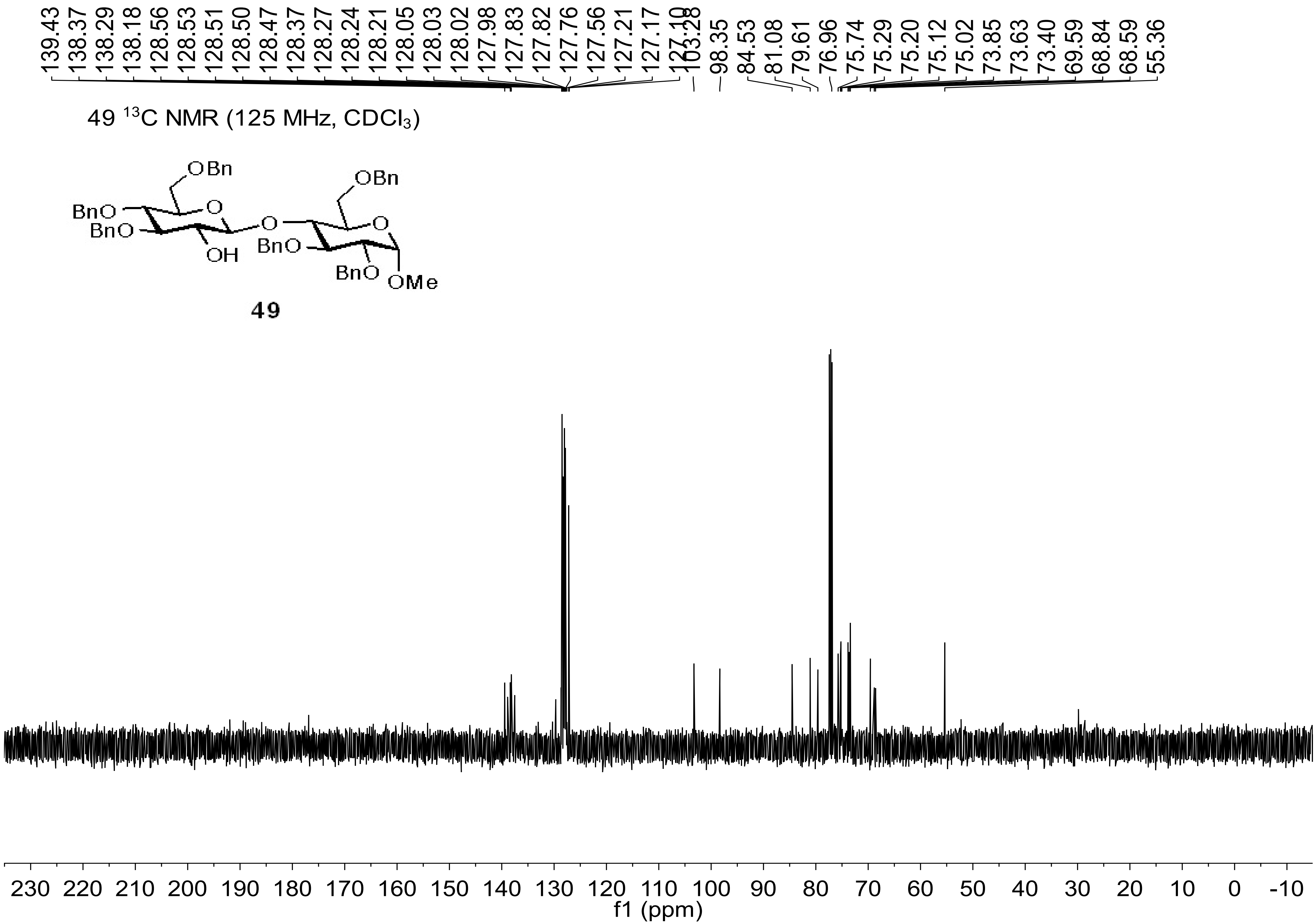

Supplementary Figure 127. ^{13}C NMR Spectrum for Compound 49

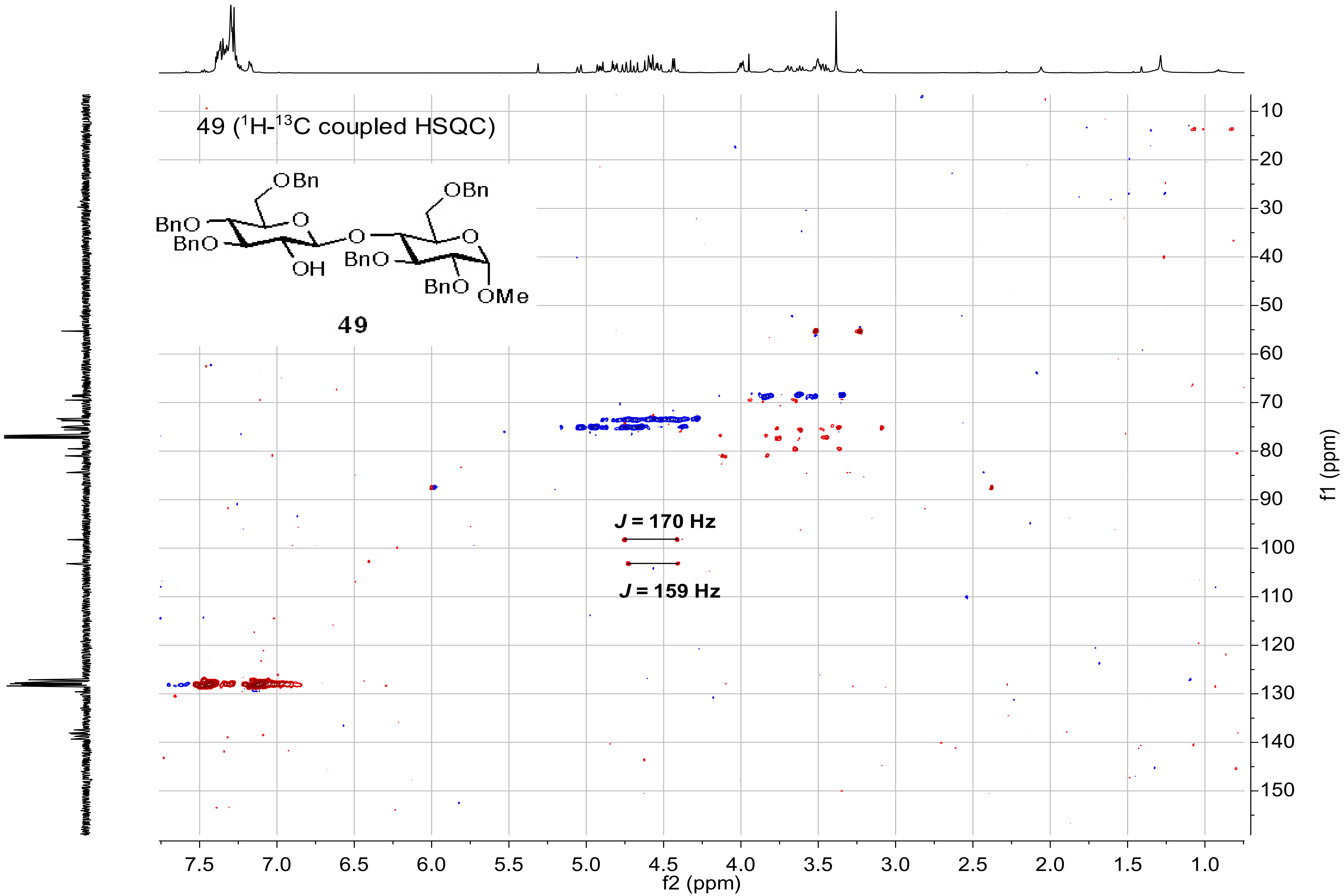

Supplementary Figure 128. ^{1}H-^{13}C **HSQC Coupled Spectrum for Compound 49**

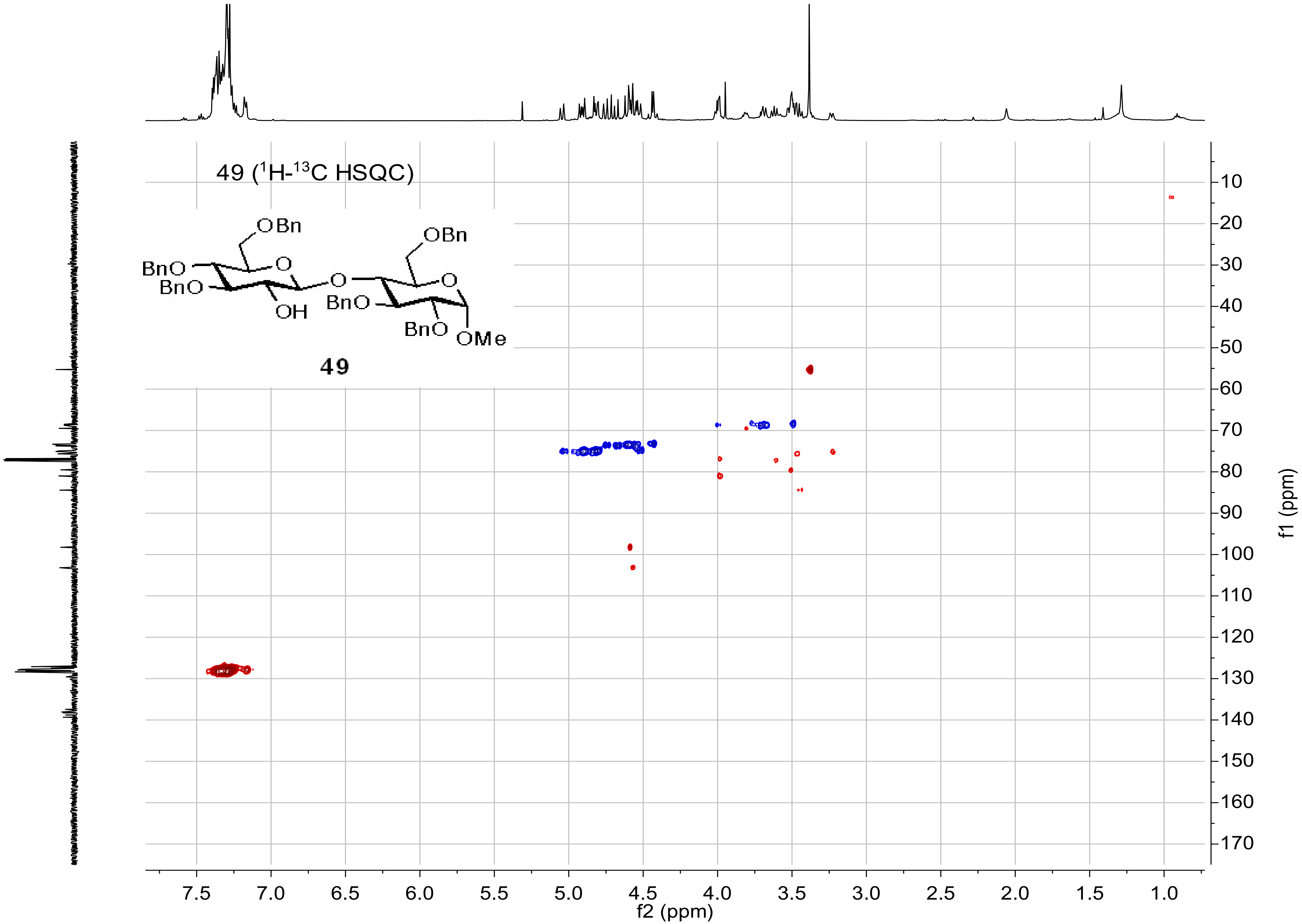

Supplementary Figure 129. ^{1}H-^{13}C **HSQC Decoupled Spectrum for Compound 49**

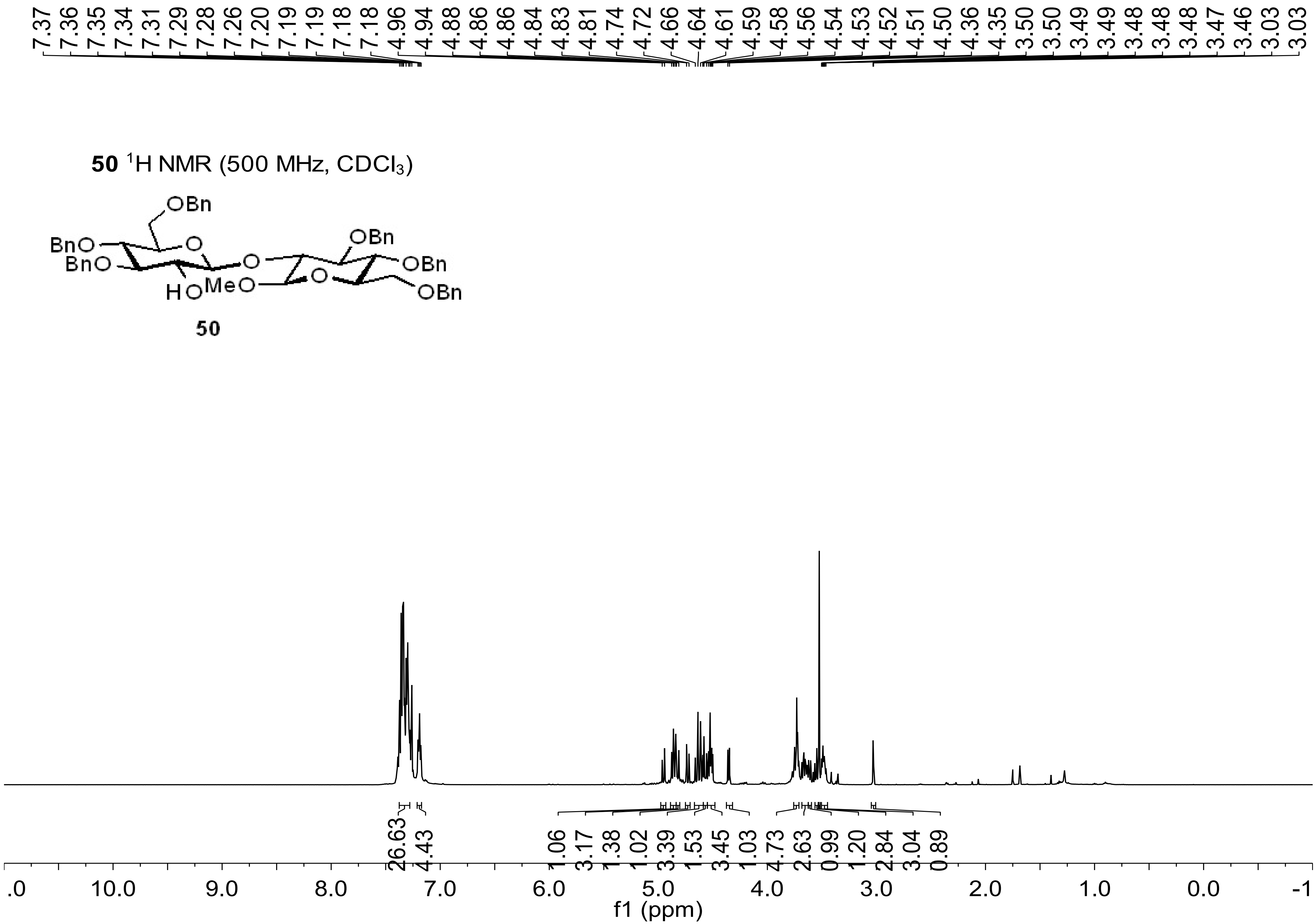

Supplementary Figure 130. ^{1}H NMR Spectrum for Compound 50

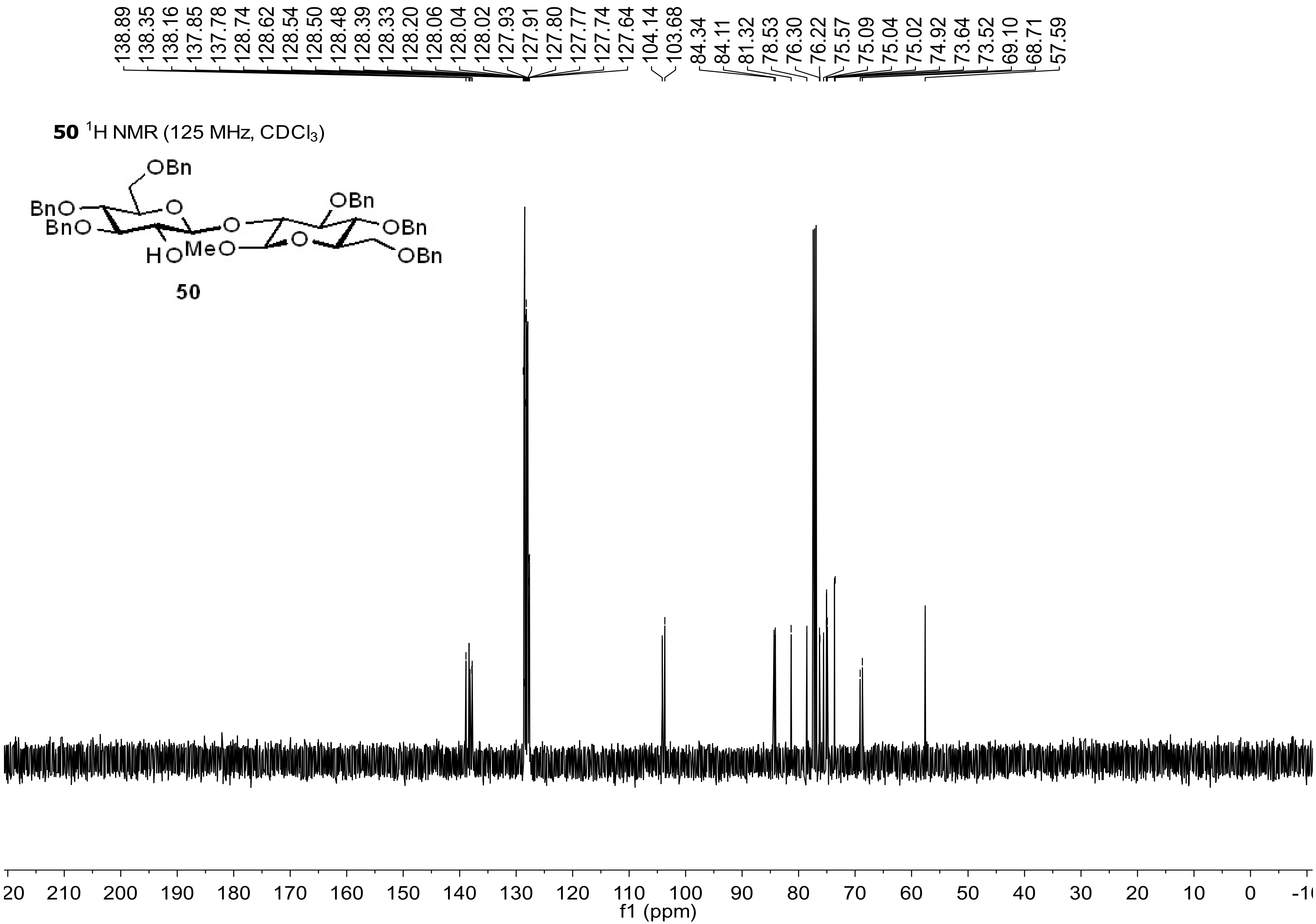

Supplementary Figure 131. ^{13}C NMR Spectrum for Compound 50

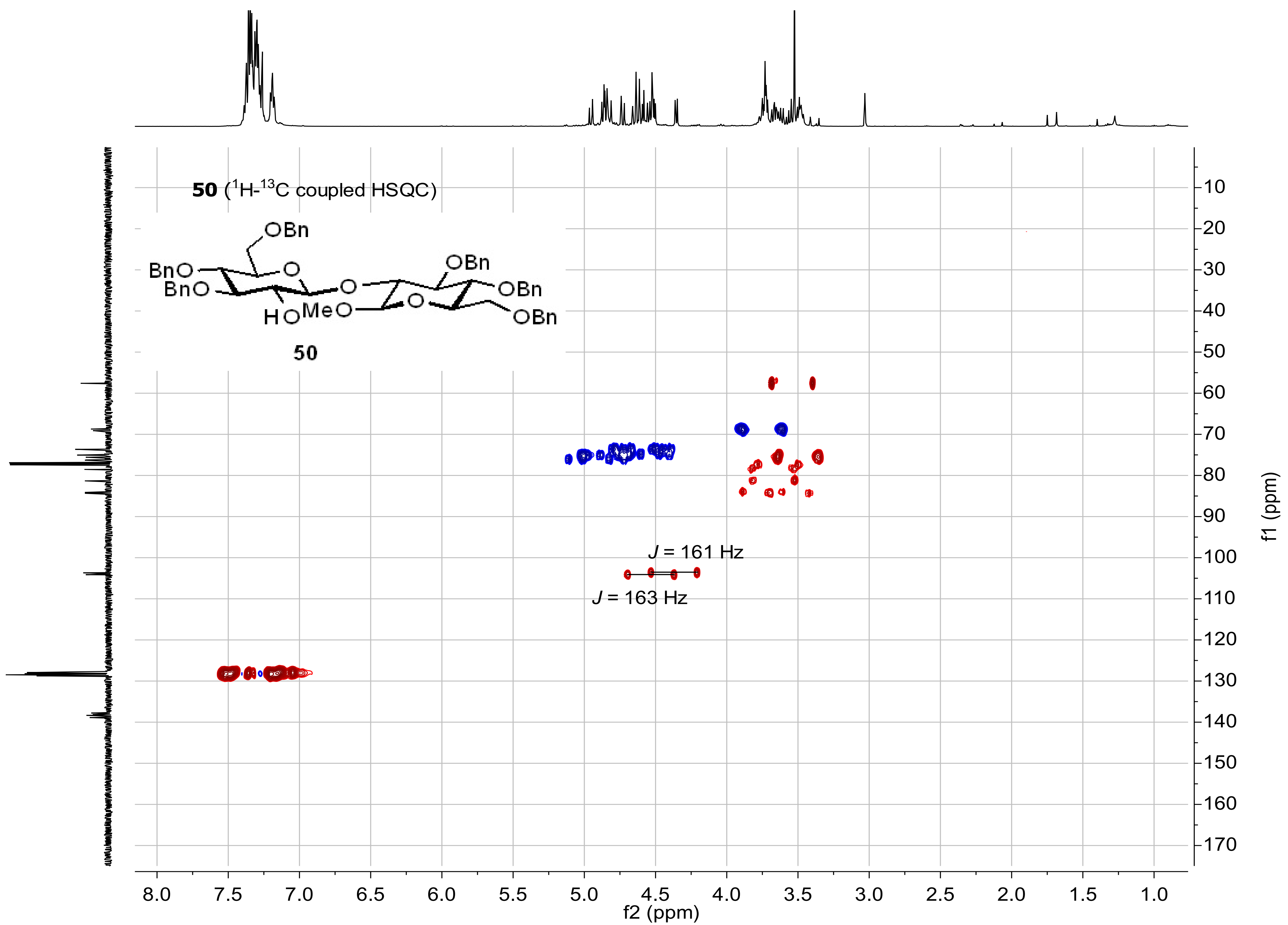

Supplementary Figure 132. ^{1}H-^{13}C **HSQC Coupled Spectrum for Compound 50**

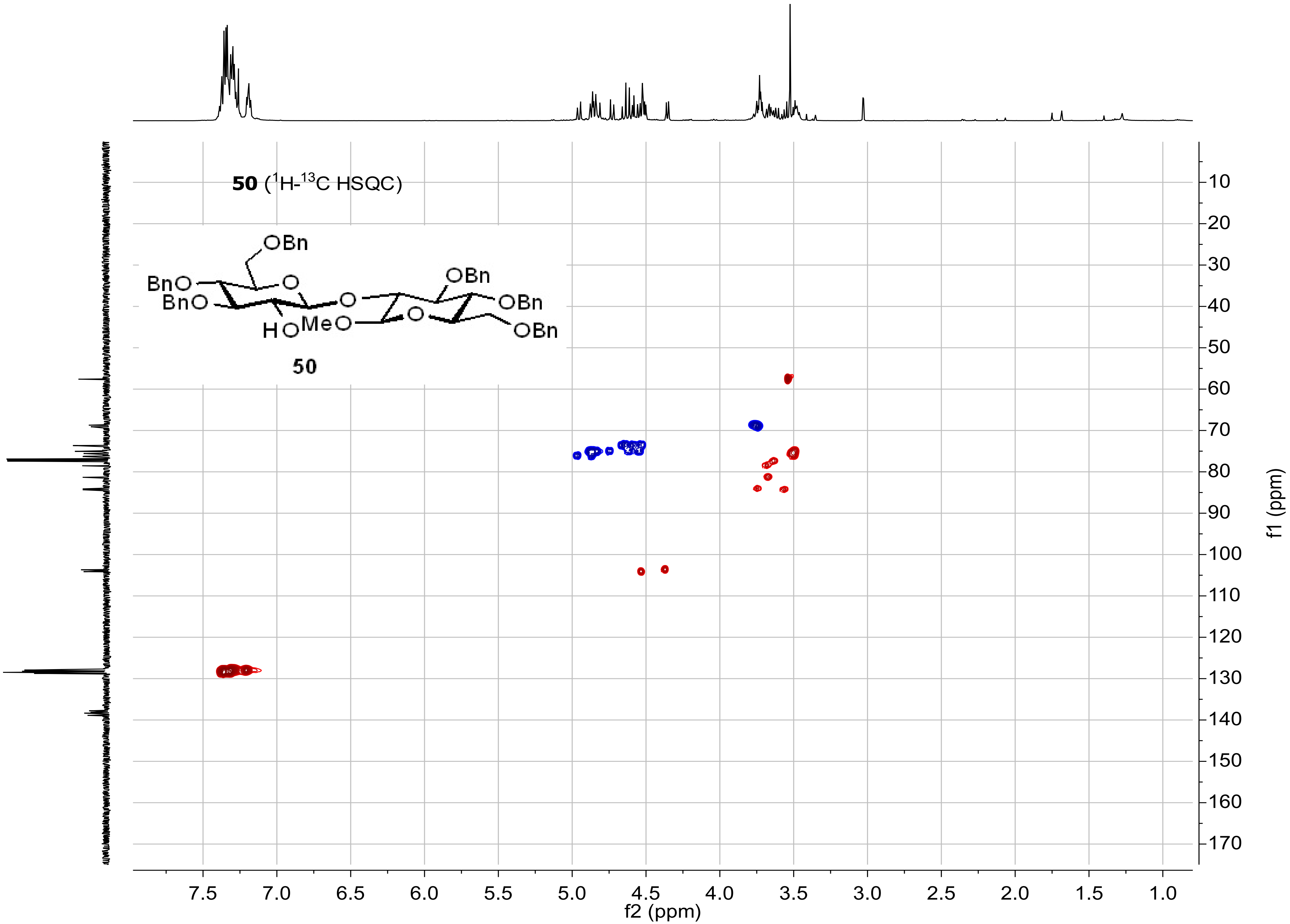

Supplementary Figure 133. **^{1}H-^{13}C HSQC Decoupled Spectrum for Compound 50**

55 (^{1}H NMR, 300MHz, $CDCl_3$)

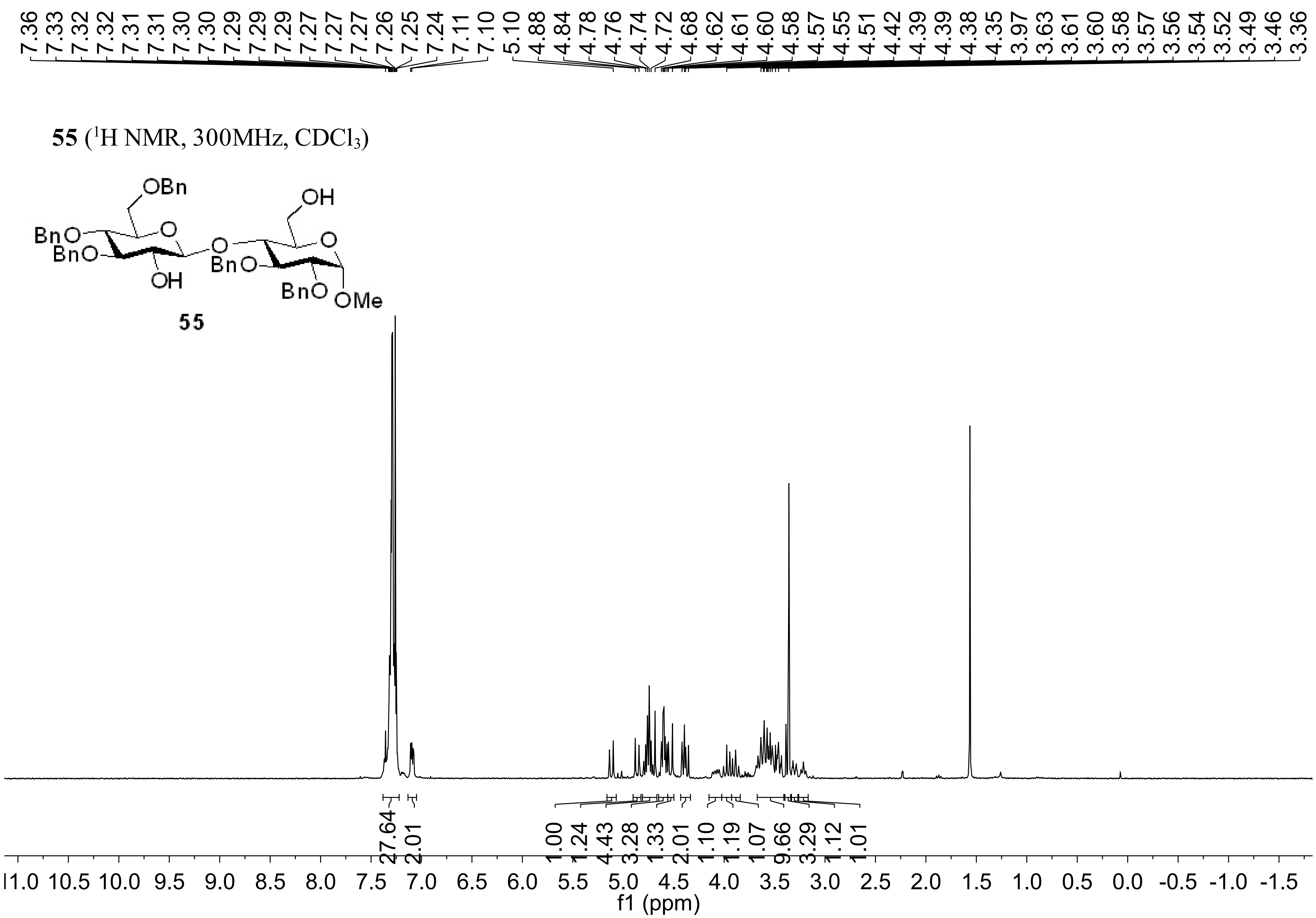

Supplementary Figure 134. ^{1}H NMR Spectrum for Compound 55

55 (^{13}C NMR, 75MHz, $CDCl_3$)

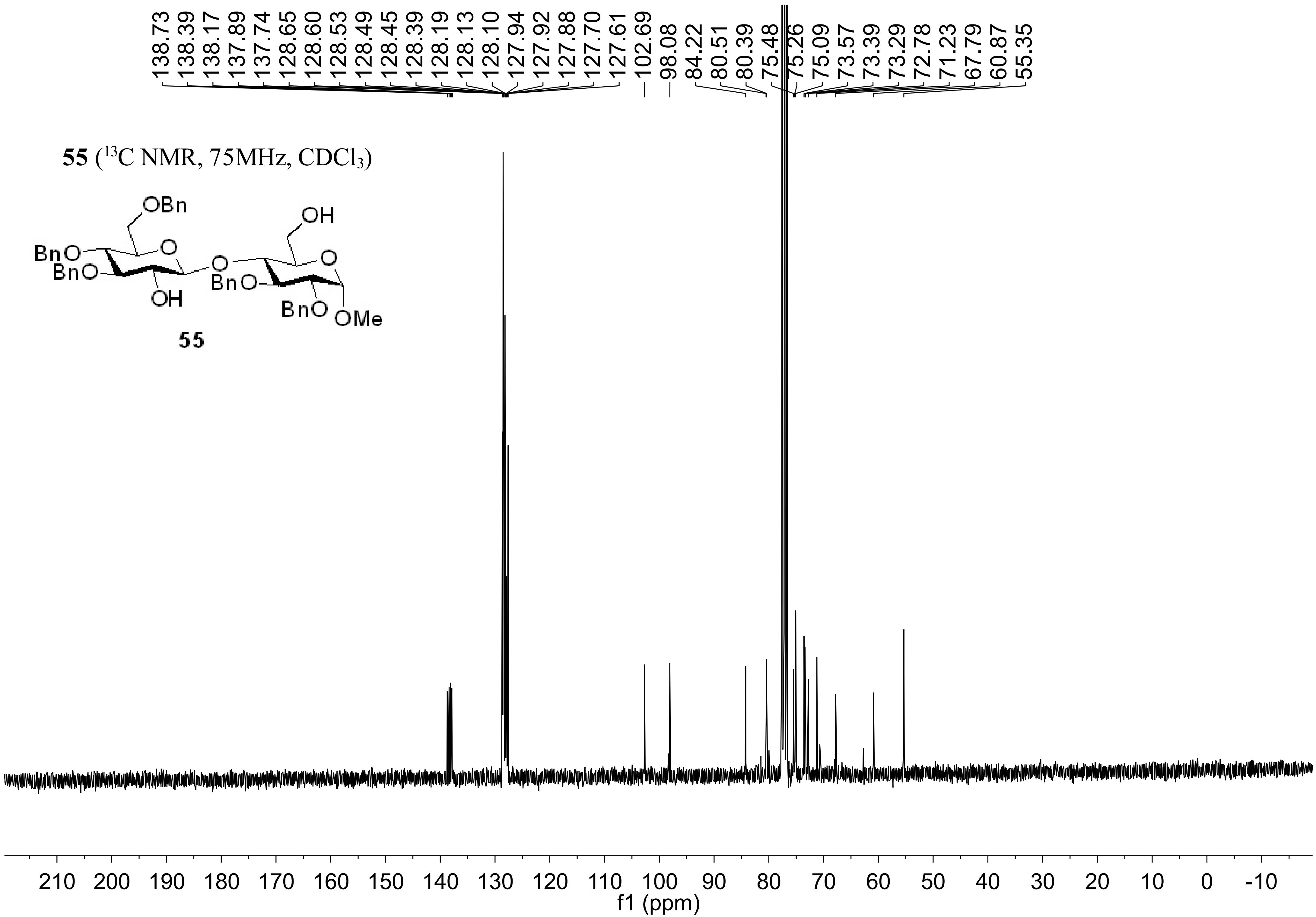

Supplementary Figure 135. ^{13}C NMR Spectrum for Compound 55

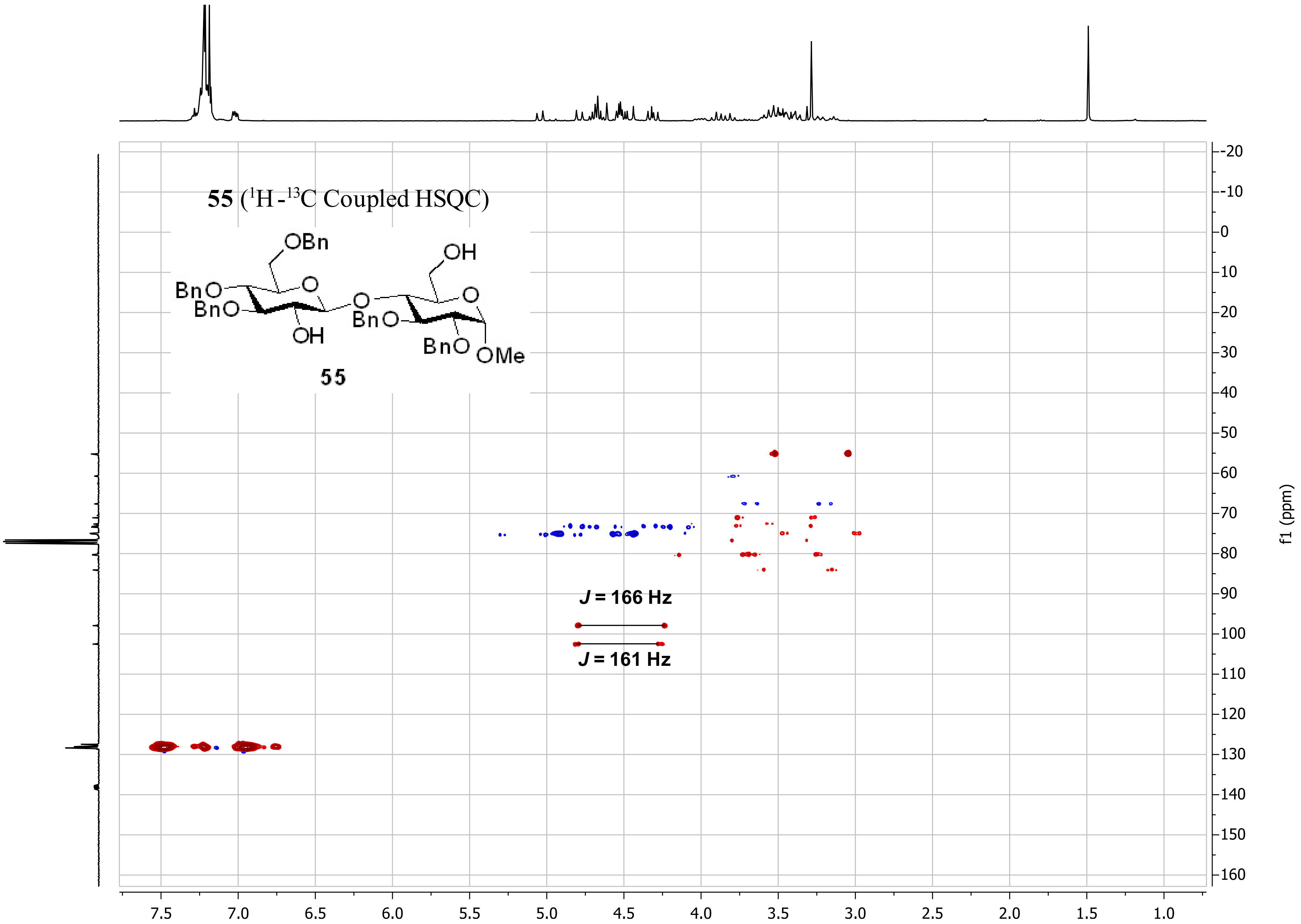

Supplementary Figure 136. ^{1}H-^{13}C **HSQC Coupled Spectrum for Compound 55**

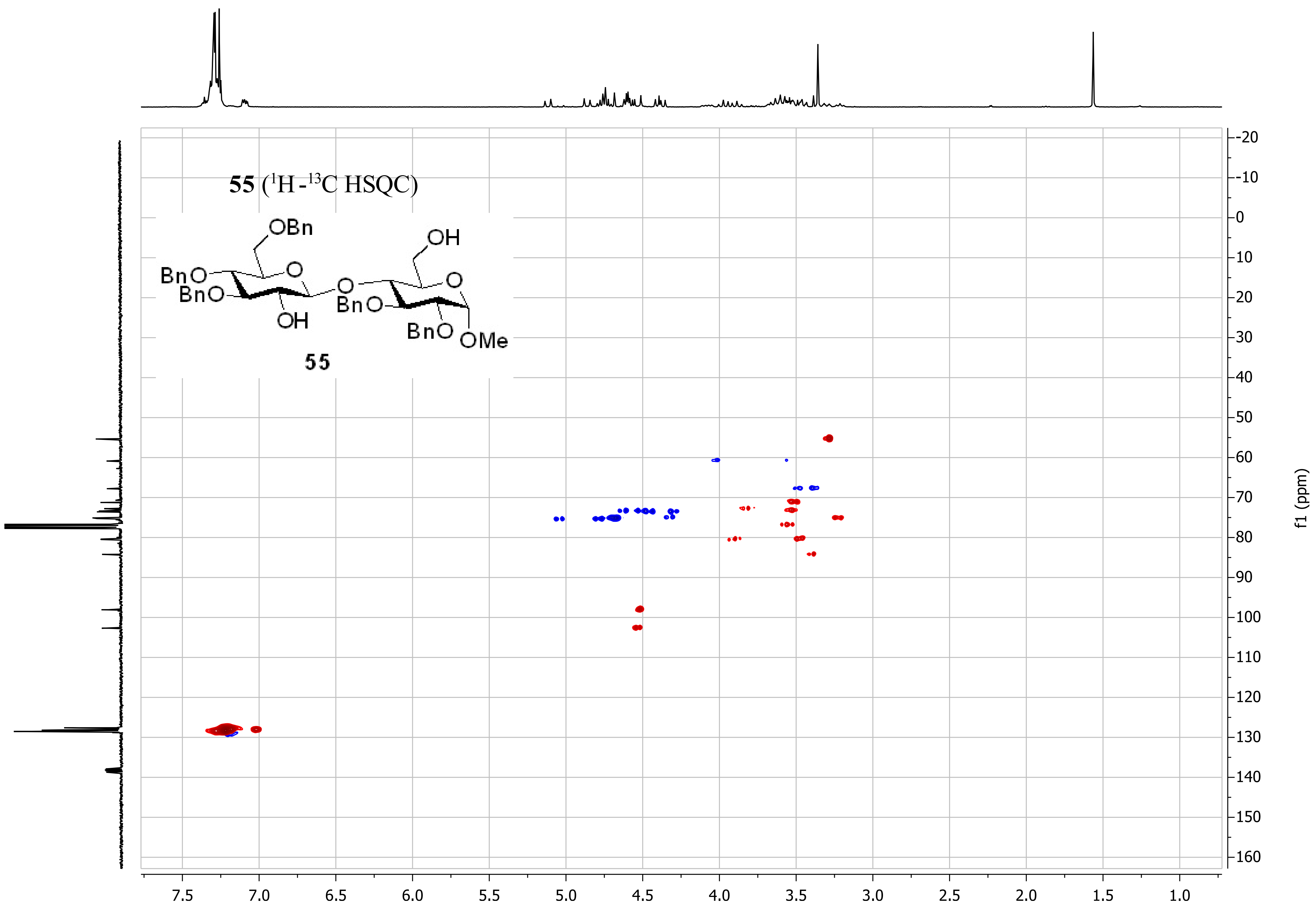

Supplementary Figure 137. ^{1}H-^{13}C **HSQC Decoupled Spectrum for Compound 55**

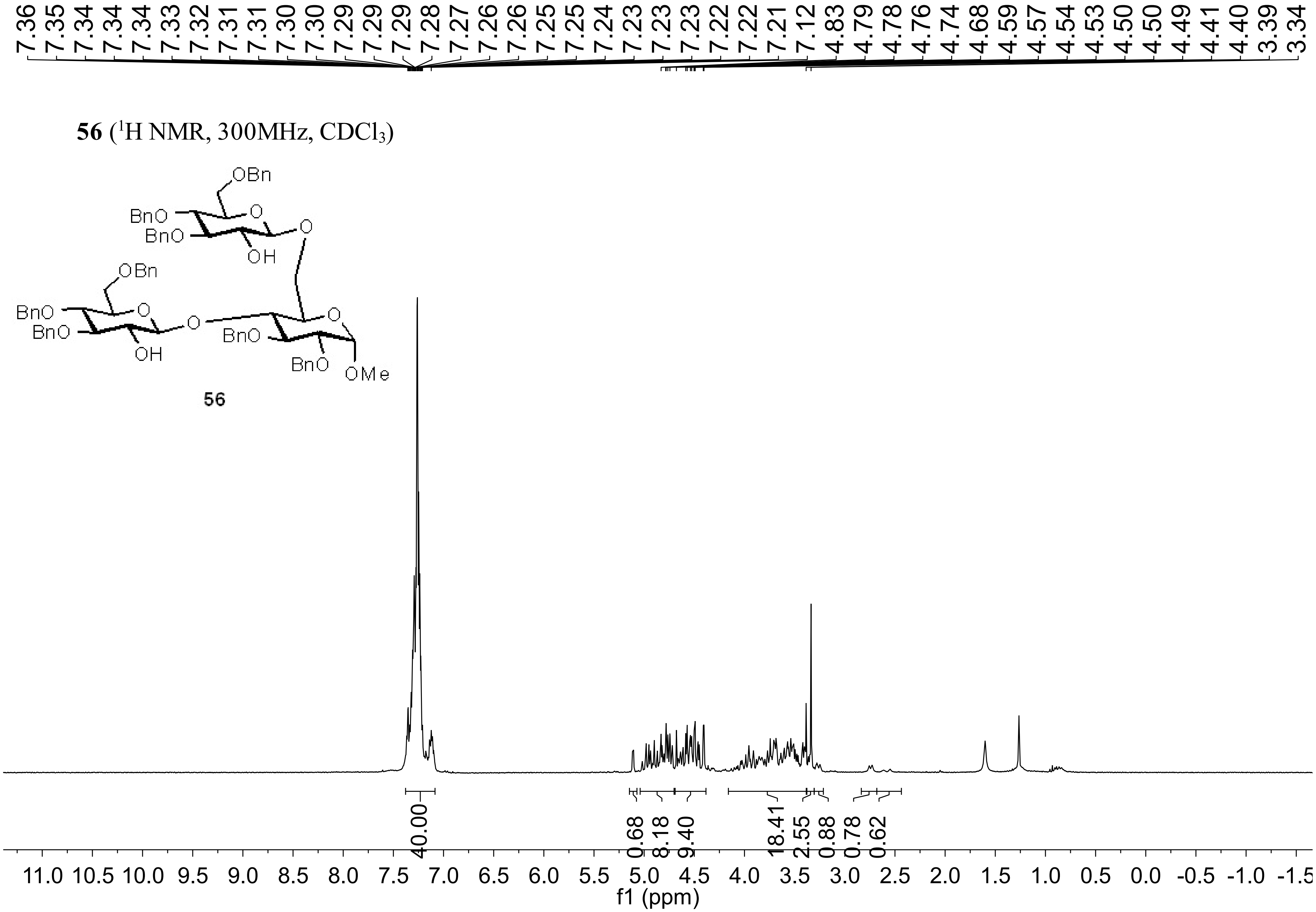

Supplementary Figure 138. ^{1}H NMR Spectrum for Compound 56

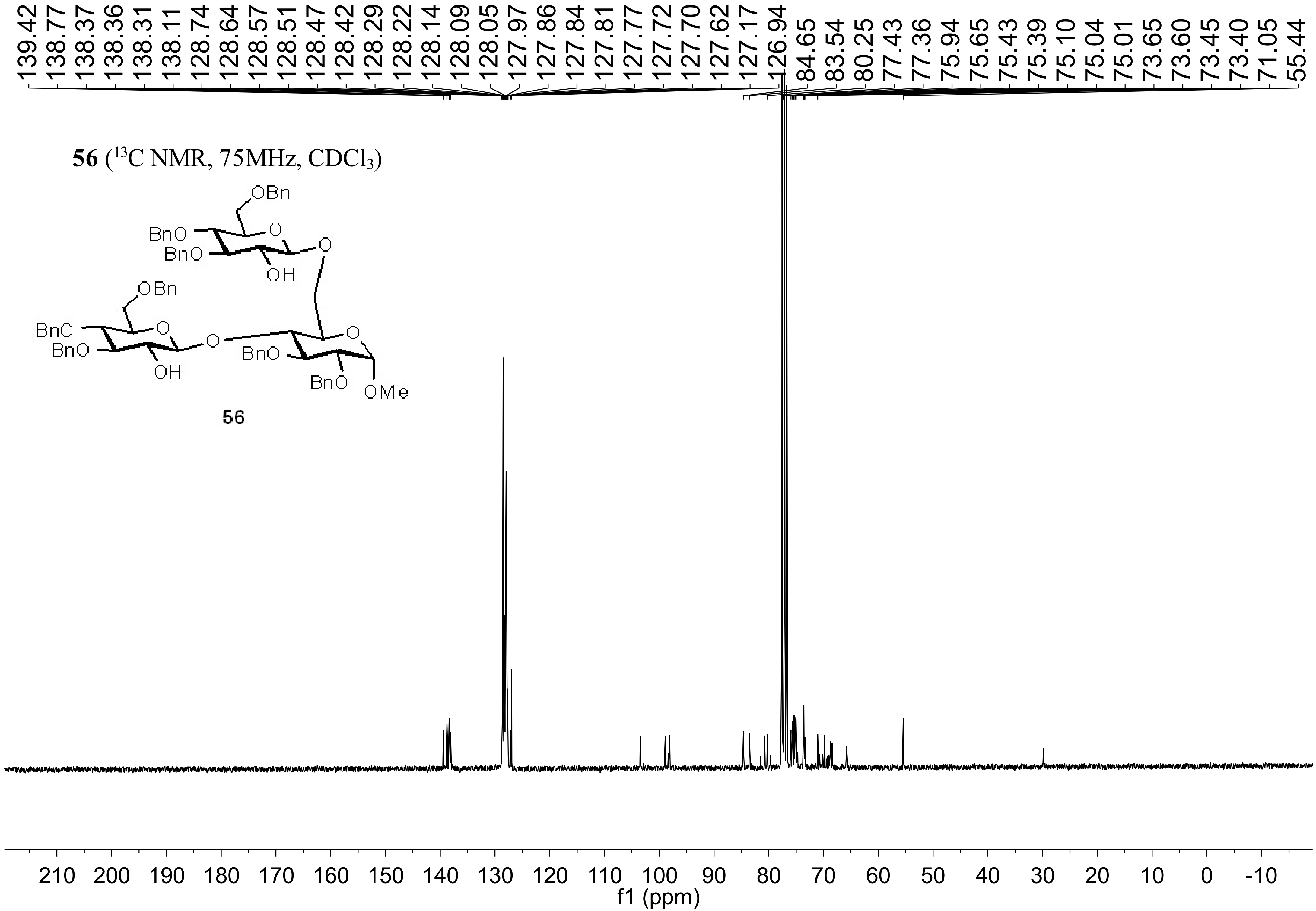

Supplementary Figure 139. **^{13}C NMR Spectrum for Compound 56**

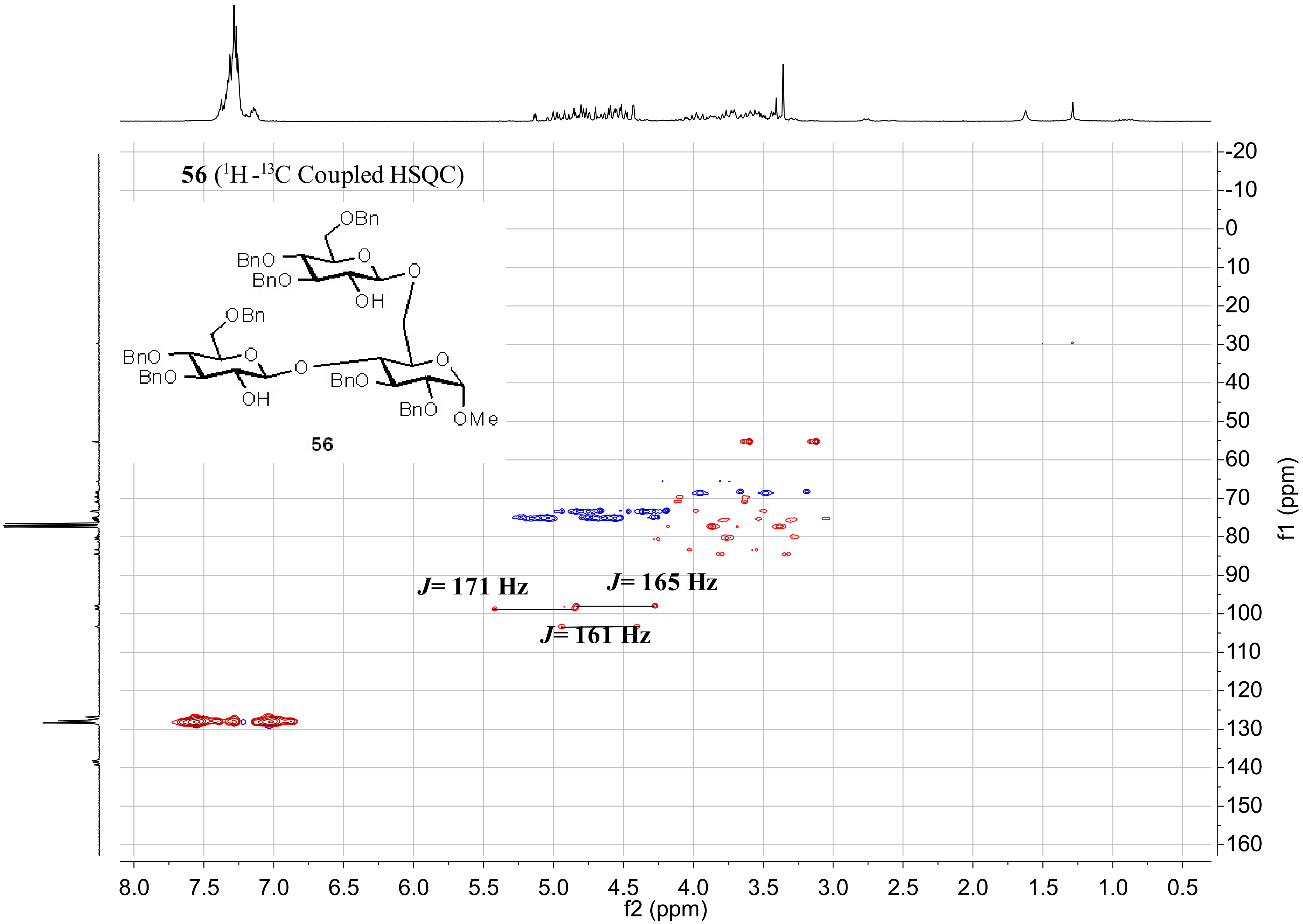

Supplementary Figure 140. ^{1}H-^{13}C **HSQC Coupled Spectrum for Compound 56**

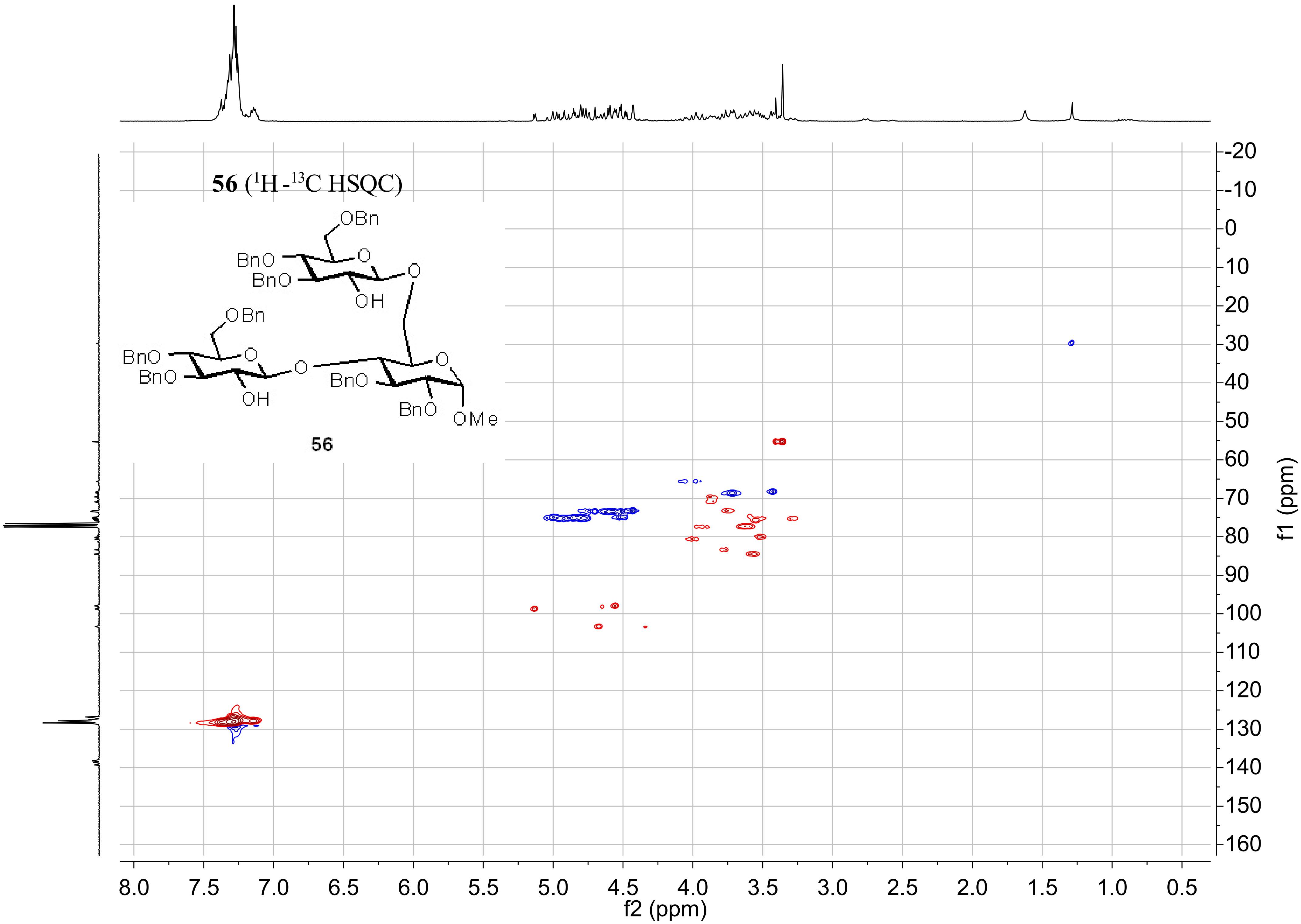

Supplementary Figure 141. ^{1}H-^{13}C HSQC Decoupled Spectrum for Compound 56

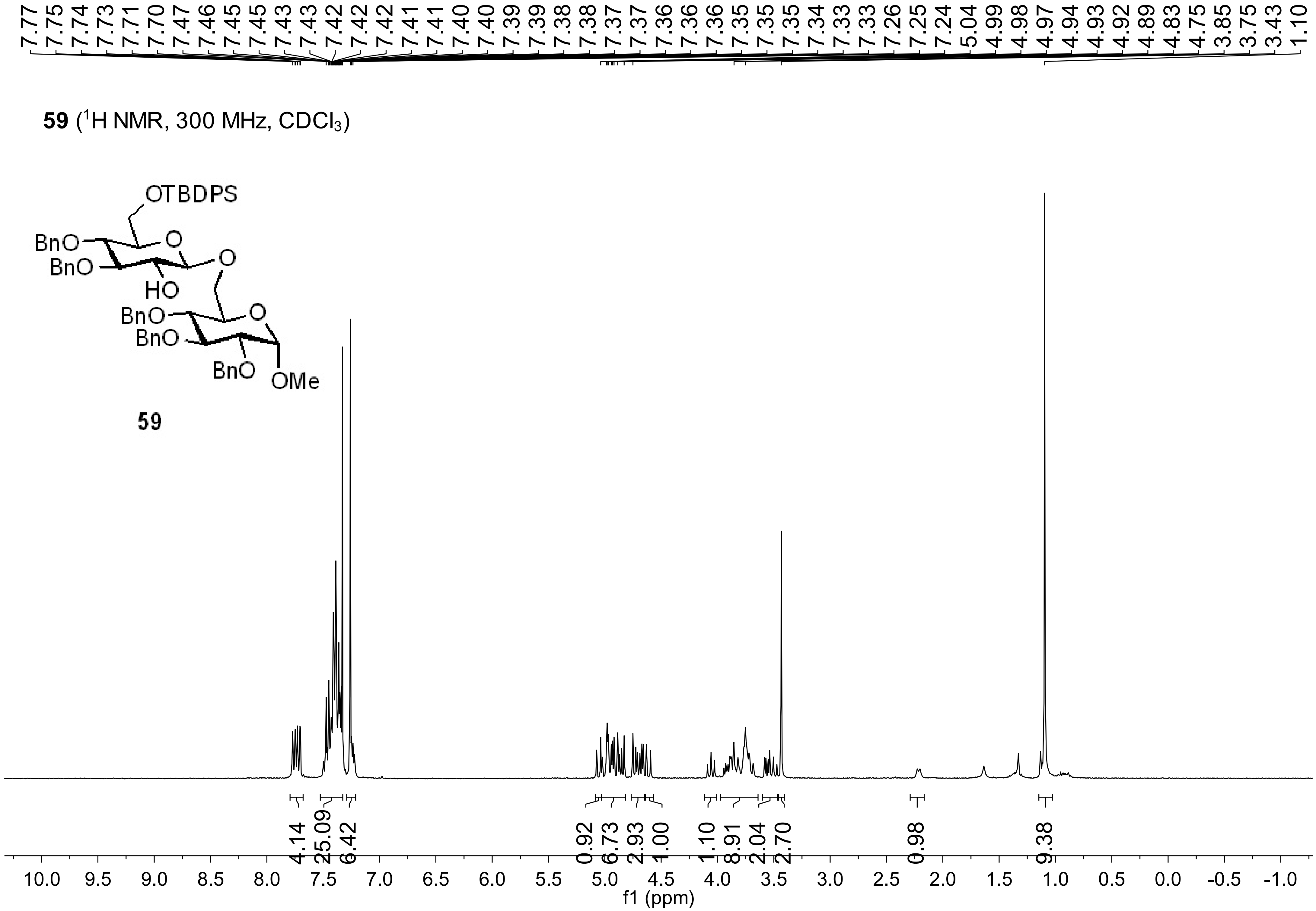

Supplementary Figure 142. ^{1}H NMR Spectrum for Compound 59

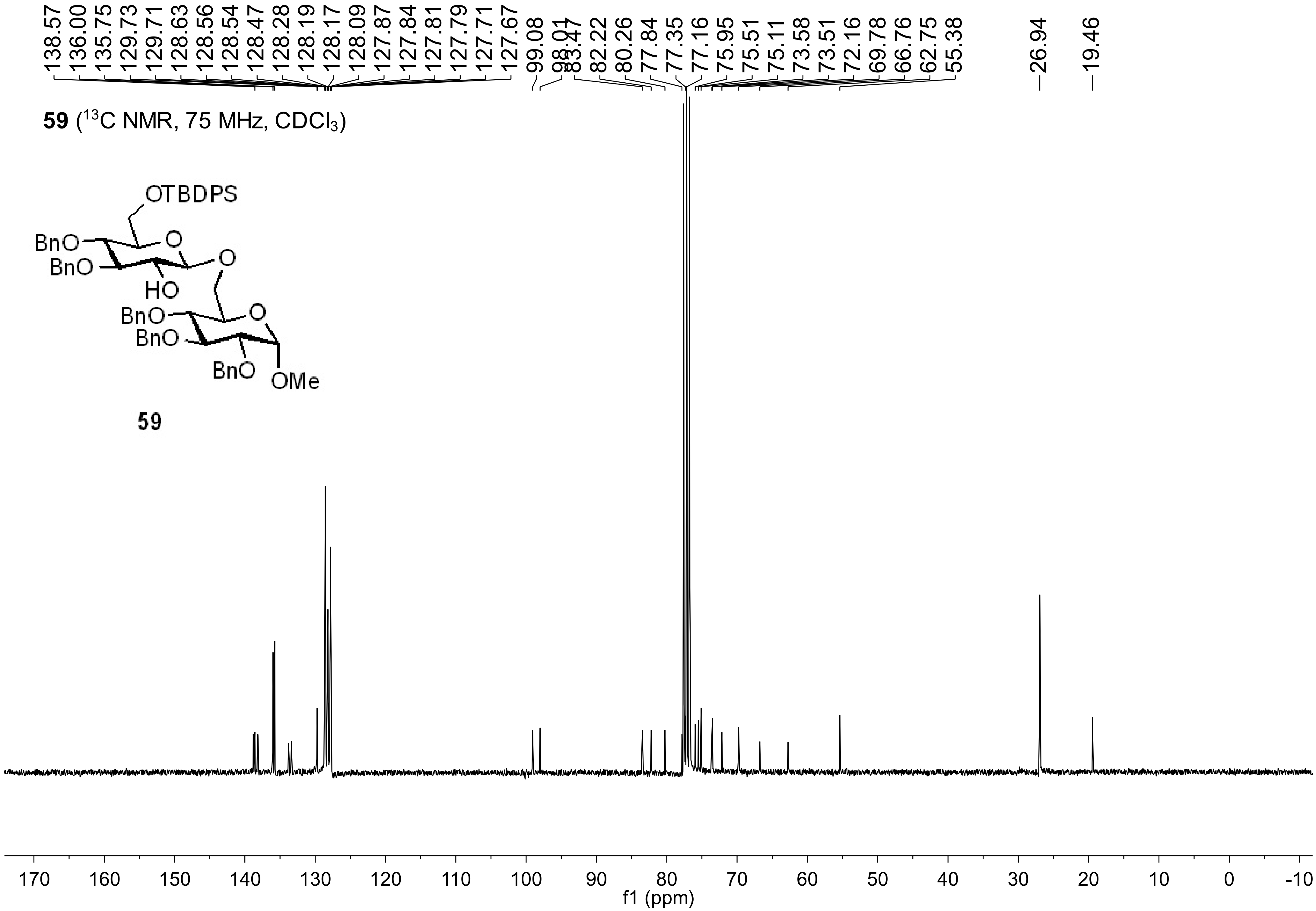

Supplementary Figure 143. ^{13}C NMR Spectrum for Compound 59

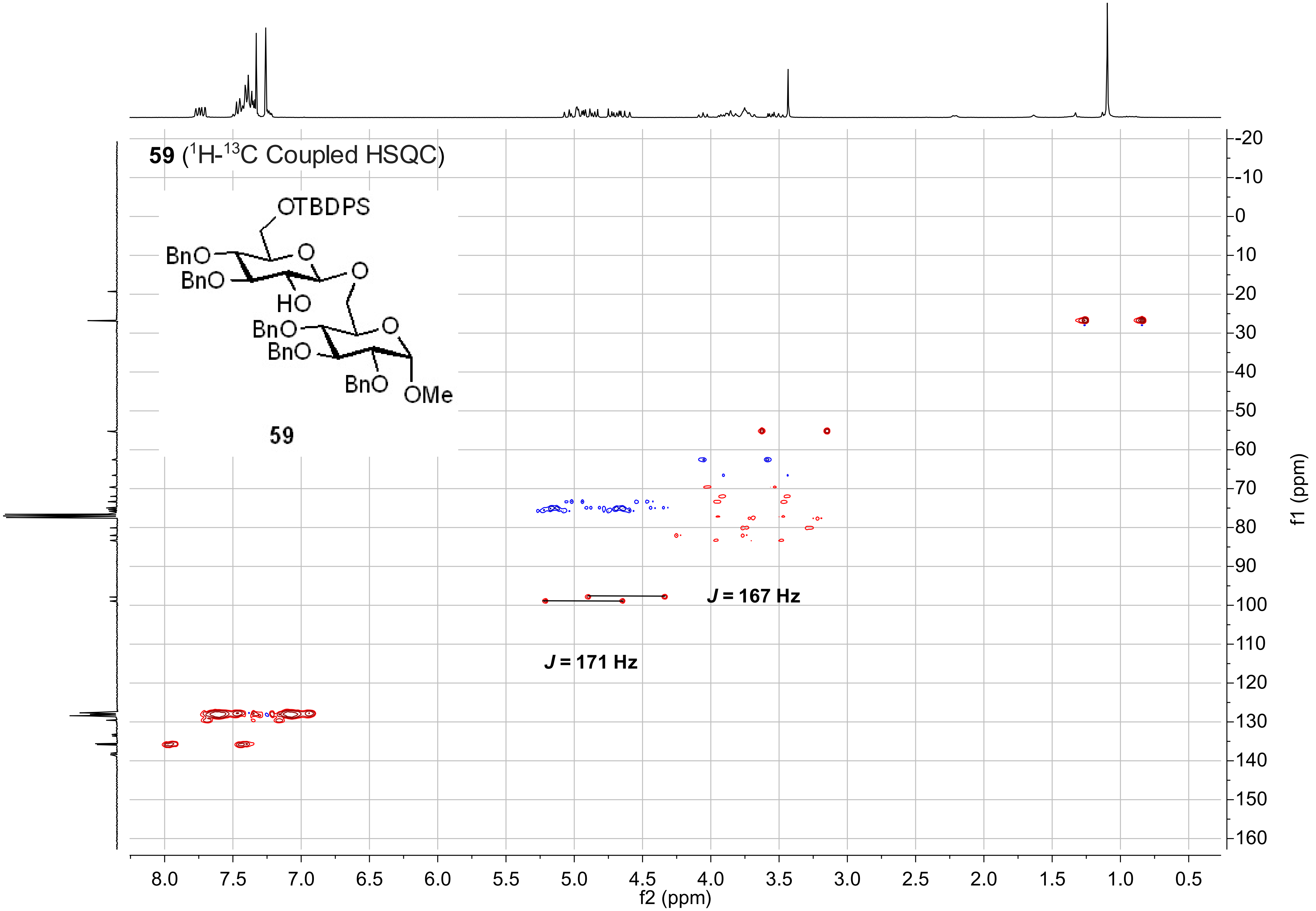

Supplementary Figure 144. ^{1}H-^{13}C HSQC Coupled Spectrum for Compound 59

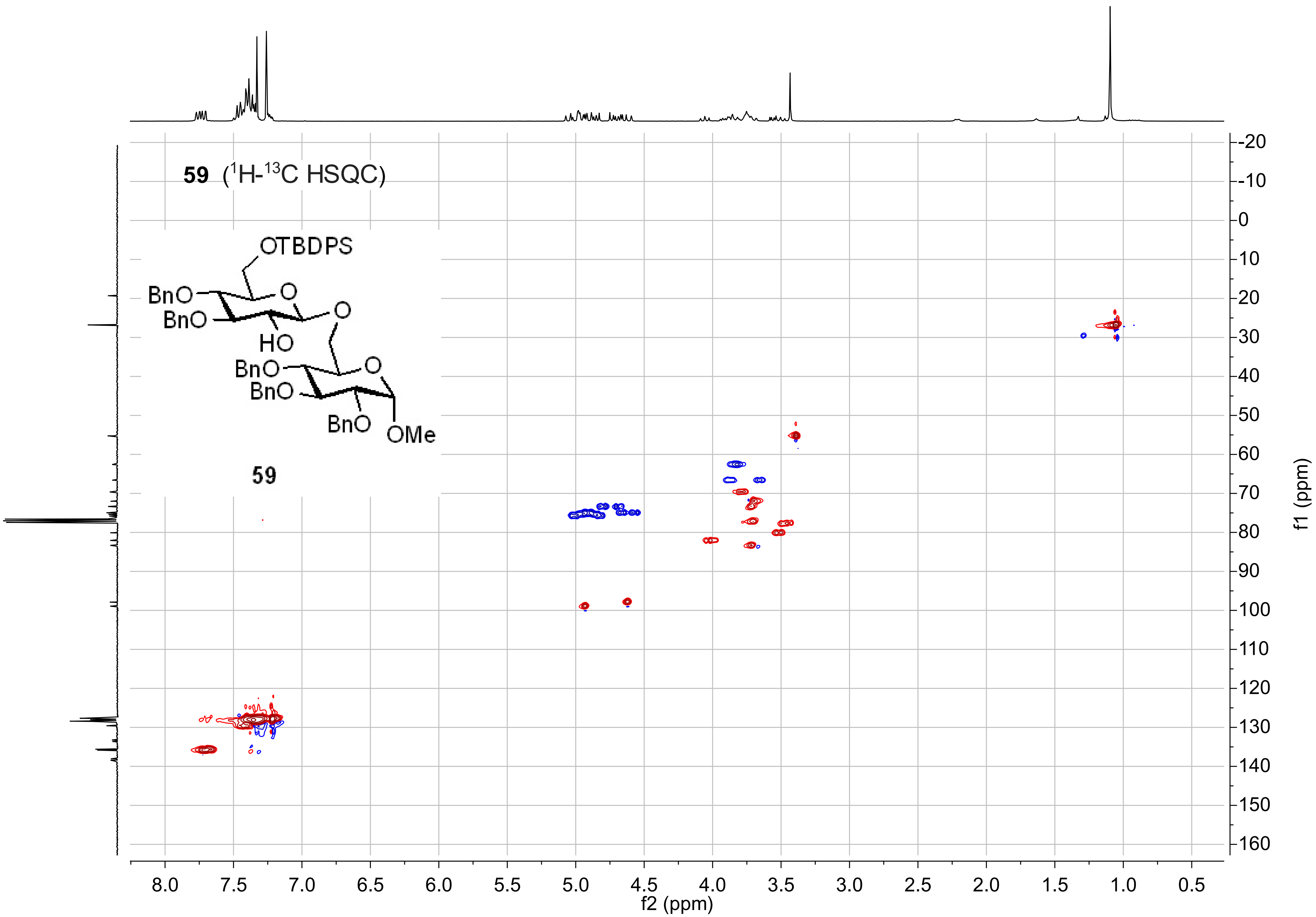

Supplementary Figure 145. ^{1}H-^{13}C HSQC Decoupled Spectrum for Compound 59

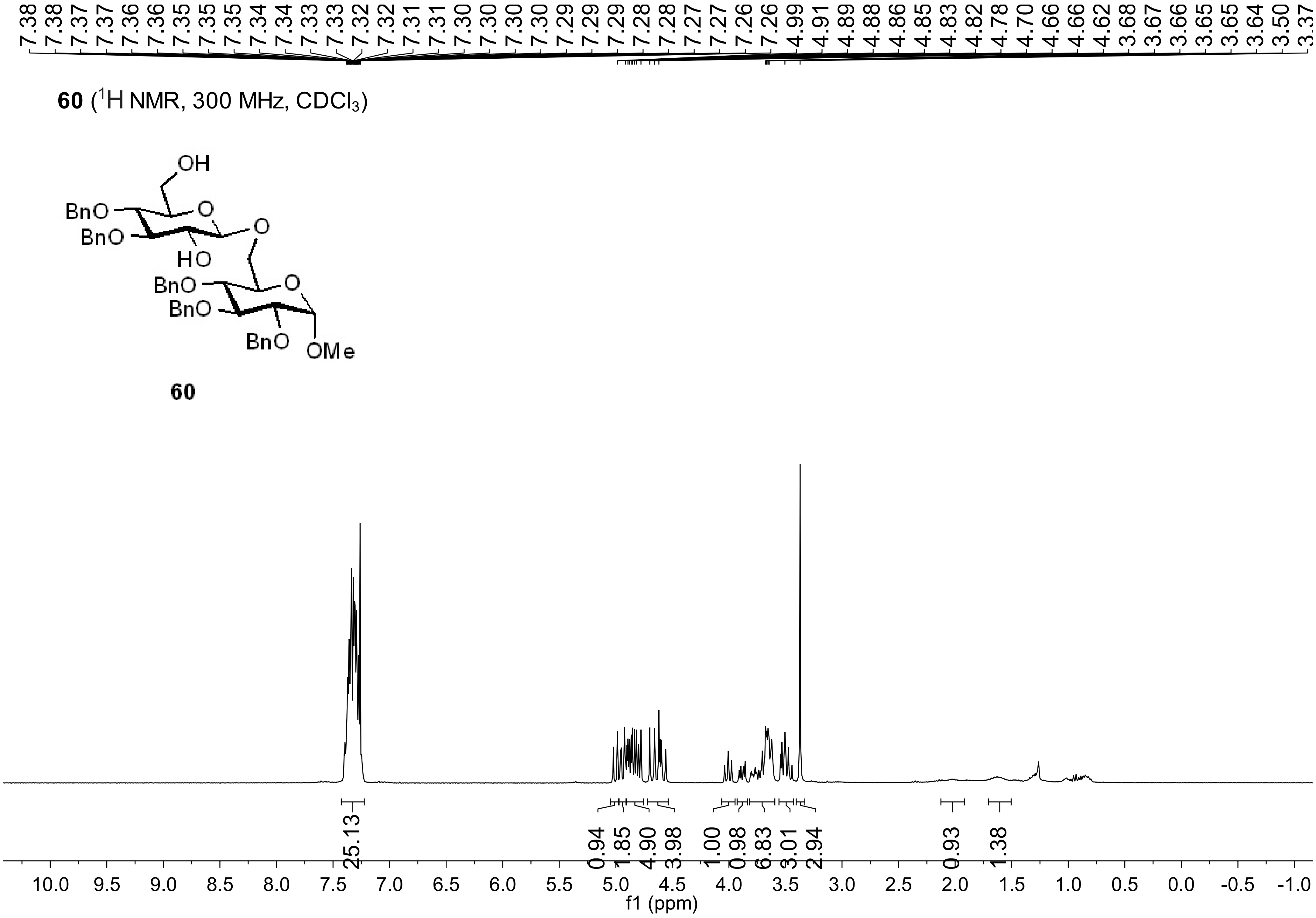

Supplementary Figure 146. ¹H NMR Spectrum for Compound 60

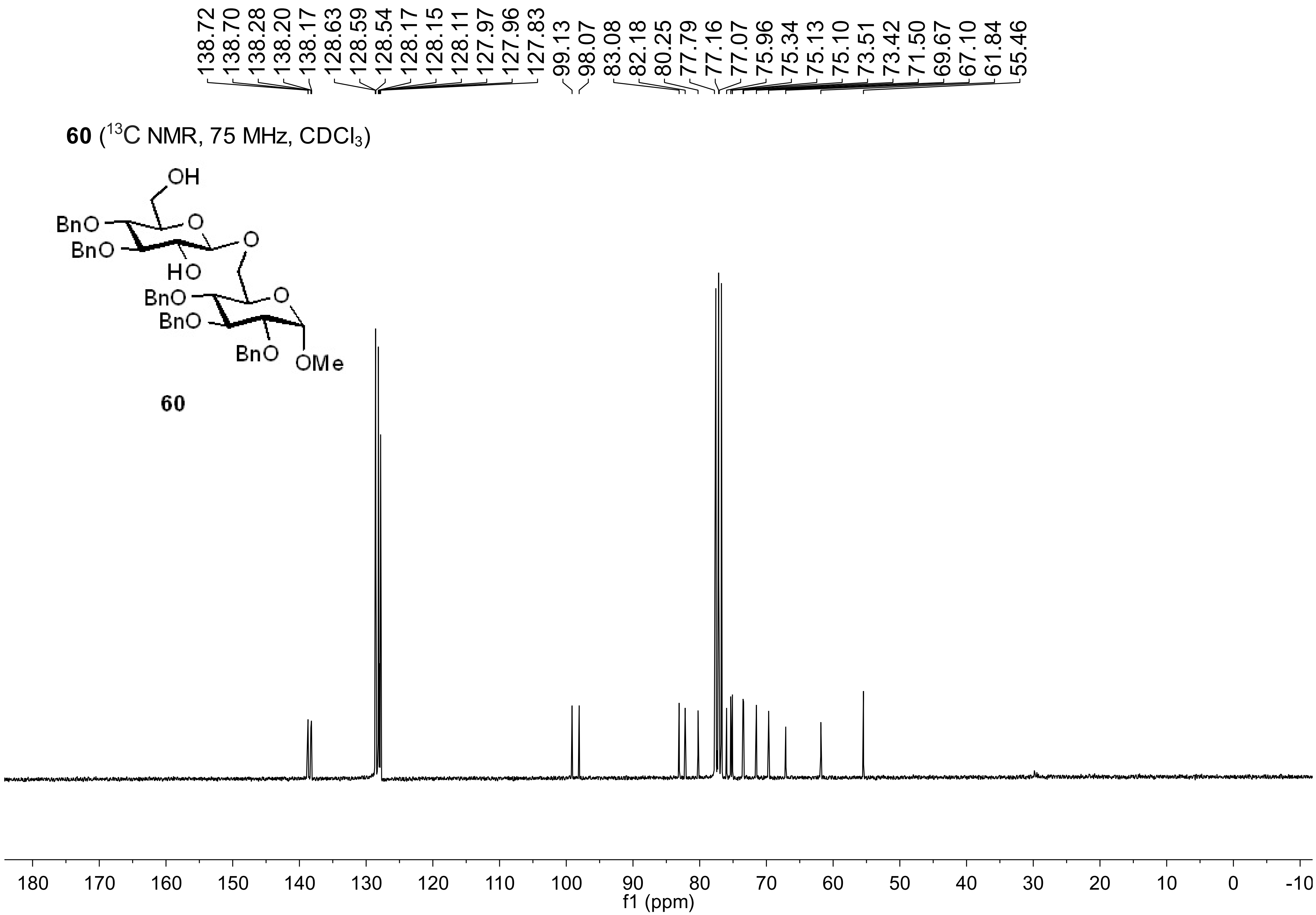

Supplementary Figure 147. ^{13}C NMR Spectrum for Compound 60

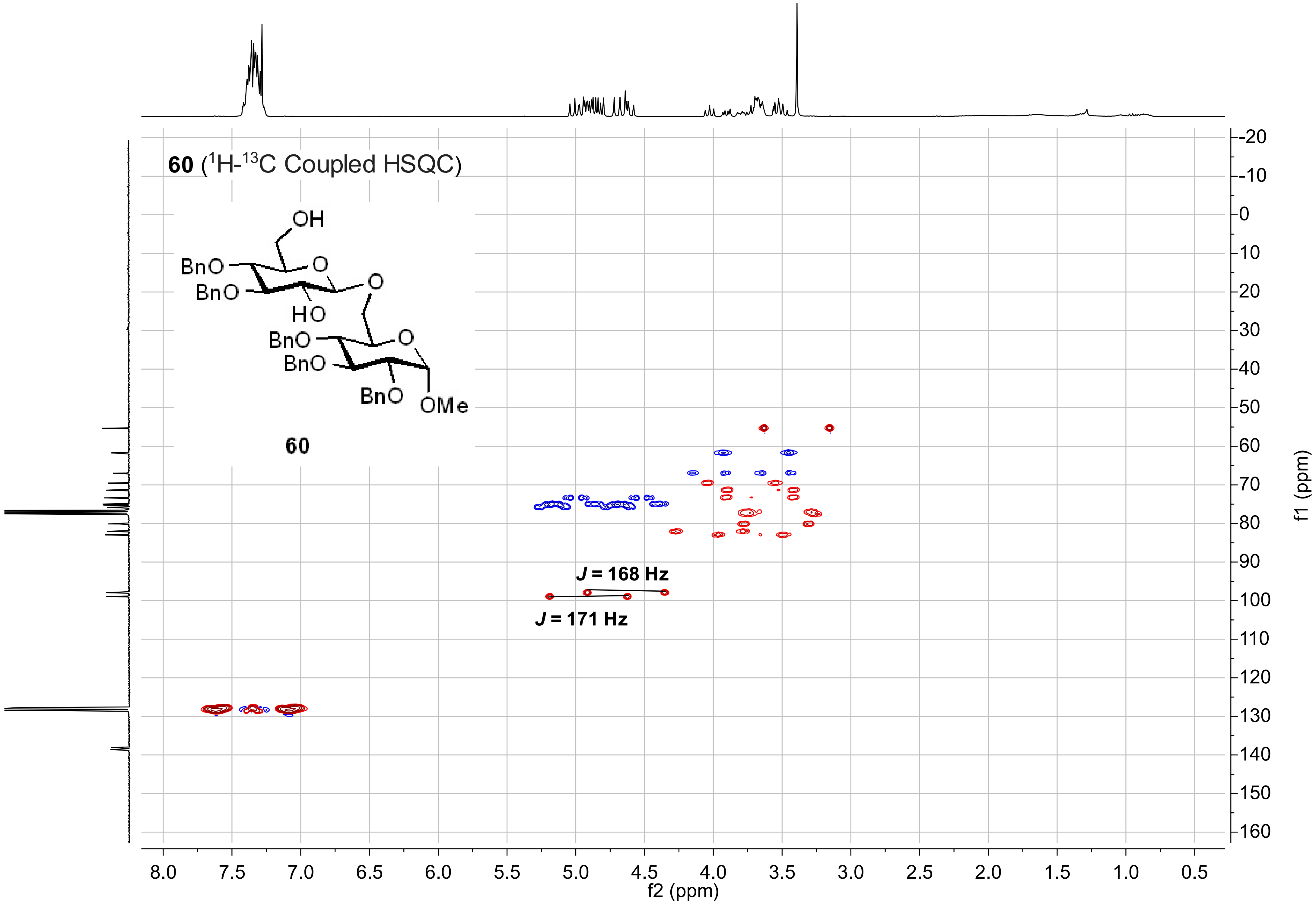

Supplementary Figure 148. ^{1}H-^{13}C HSQC Coupled Spectrum for Compound 60

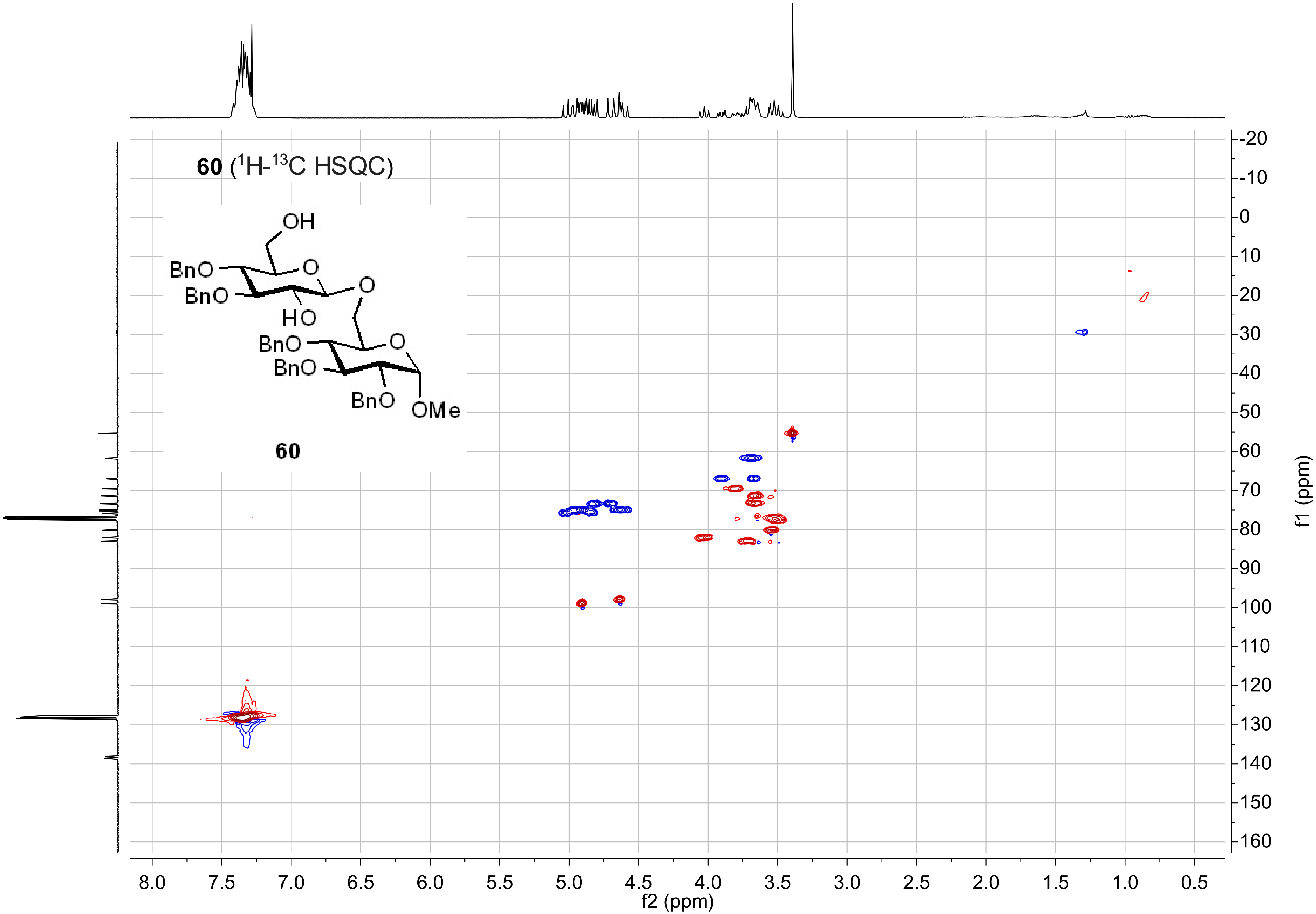

Supplementary Figure 149. **^{1}H-^{13}C HSQC Decoupled Spectrum for Compound 60**

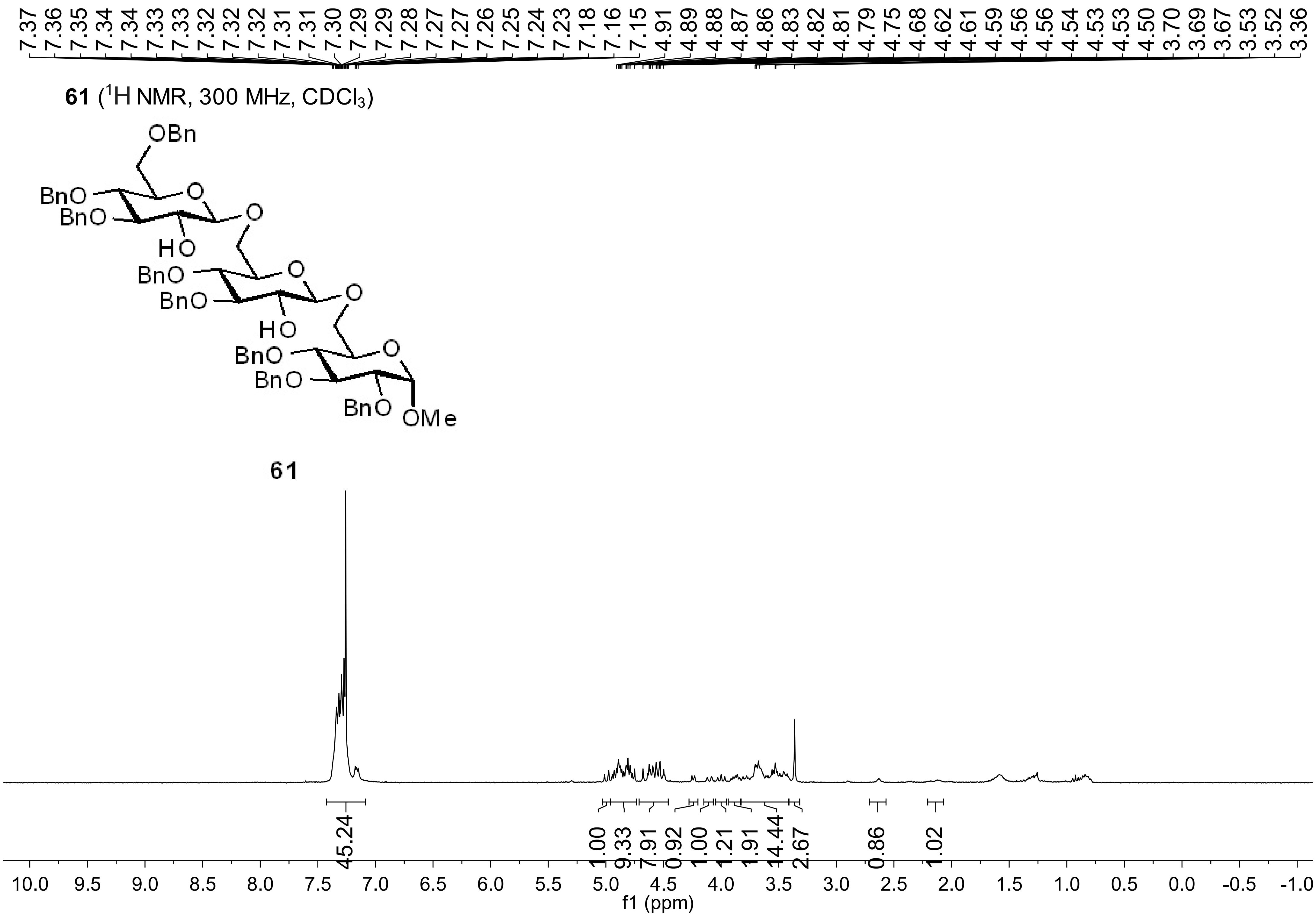

Supplementary Figure 150. ^{1}H NMR Spectrum for Compound 61

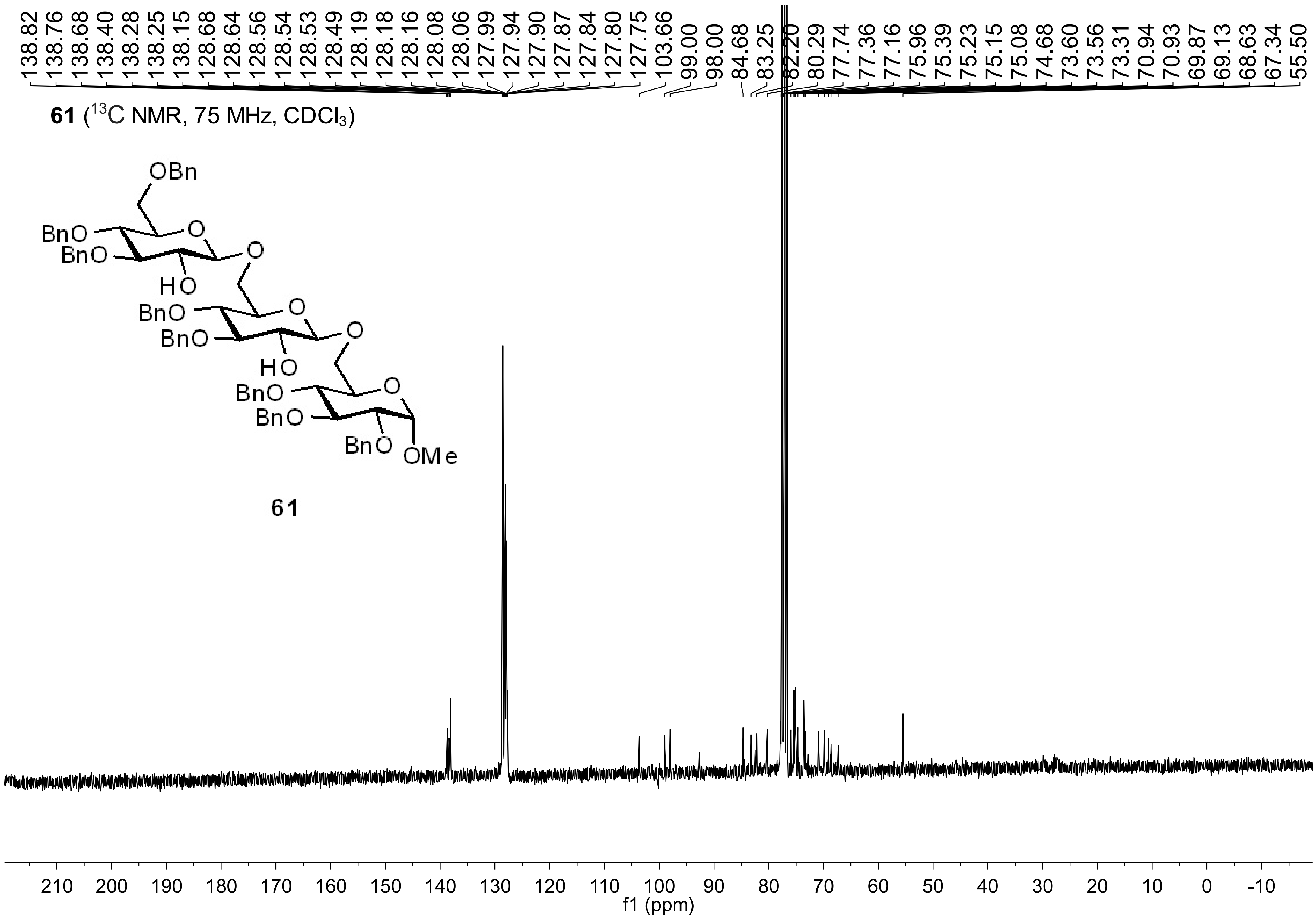

Supplementary Figure 151. **^{13}C NMR Spectrum for Compound 61**

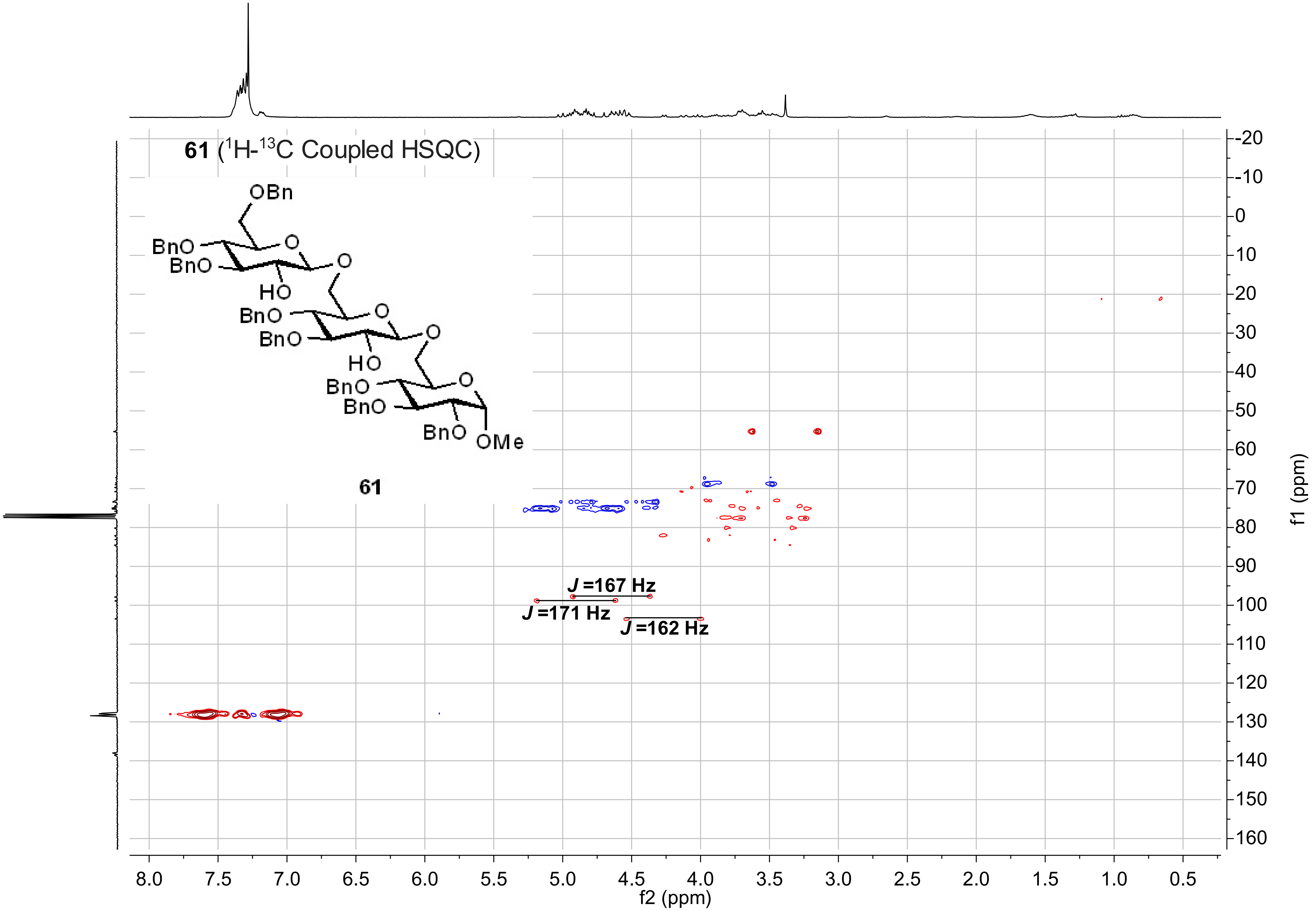

Supplementary Figure 152. ^1H-^{13}C HSQC Coupled Spectrum for Compound 61

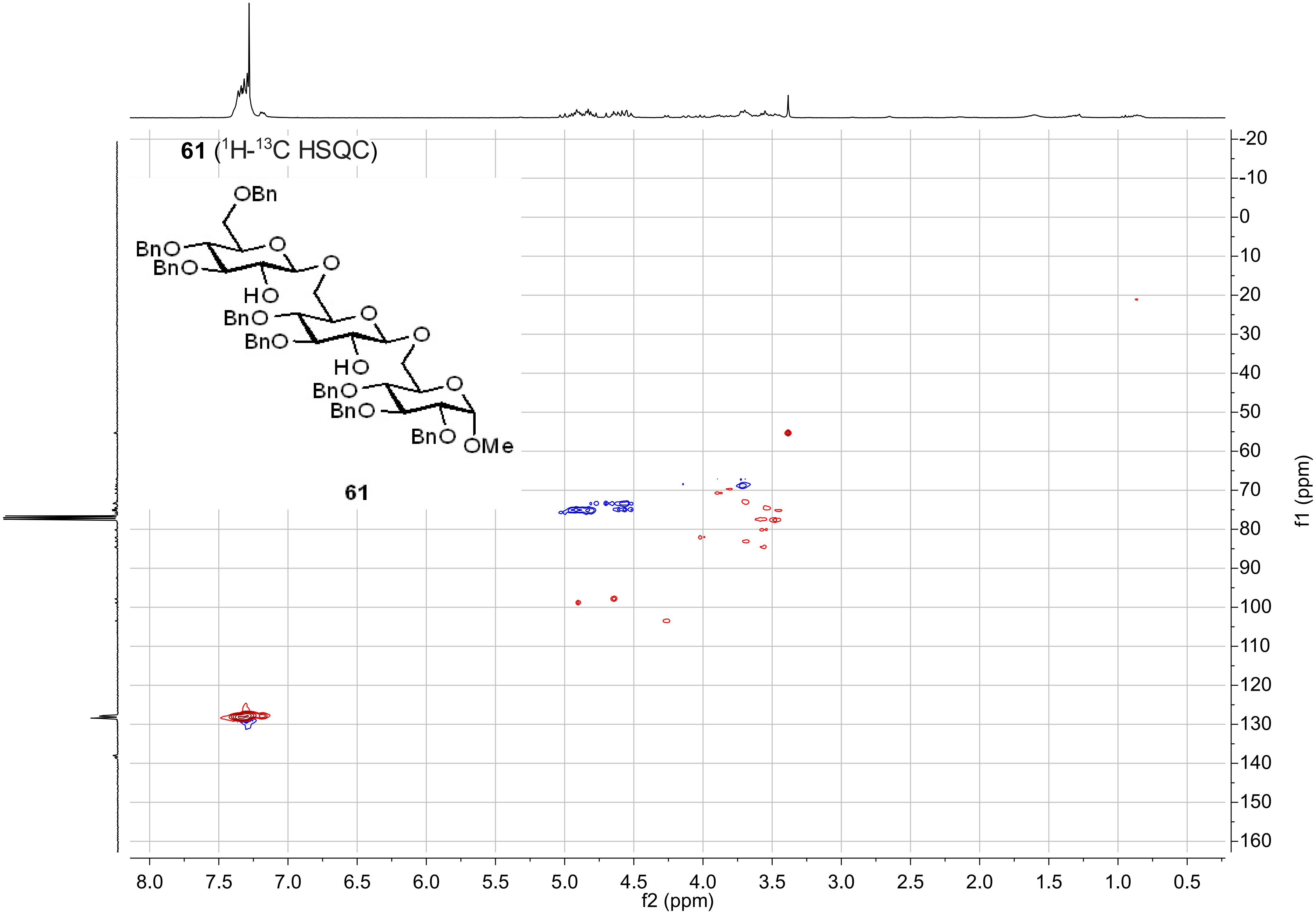

Supplementary Figure 153. ^{1}H-^{13}C HSQC Decoupled Spectrum for Compound 61

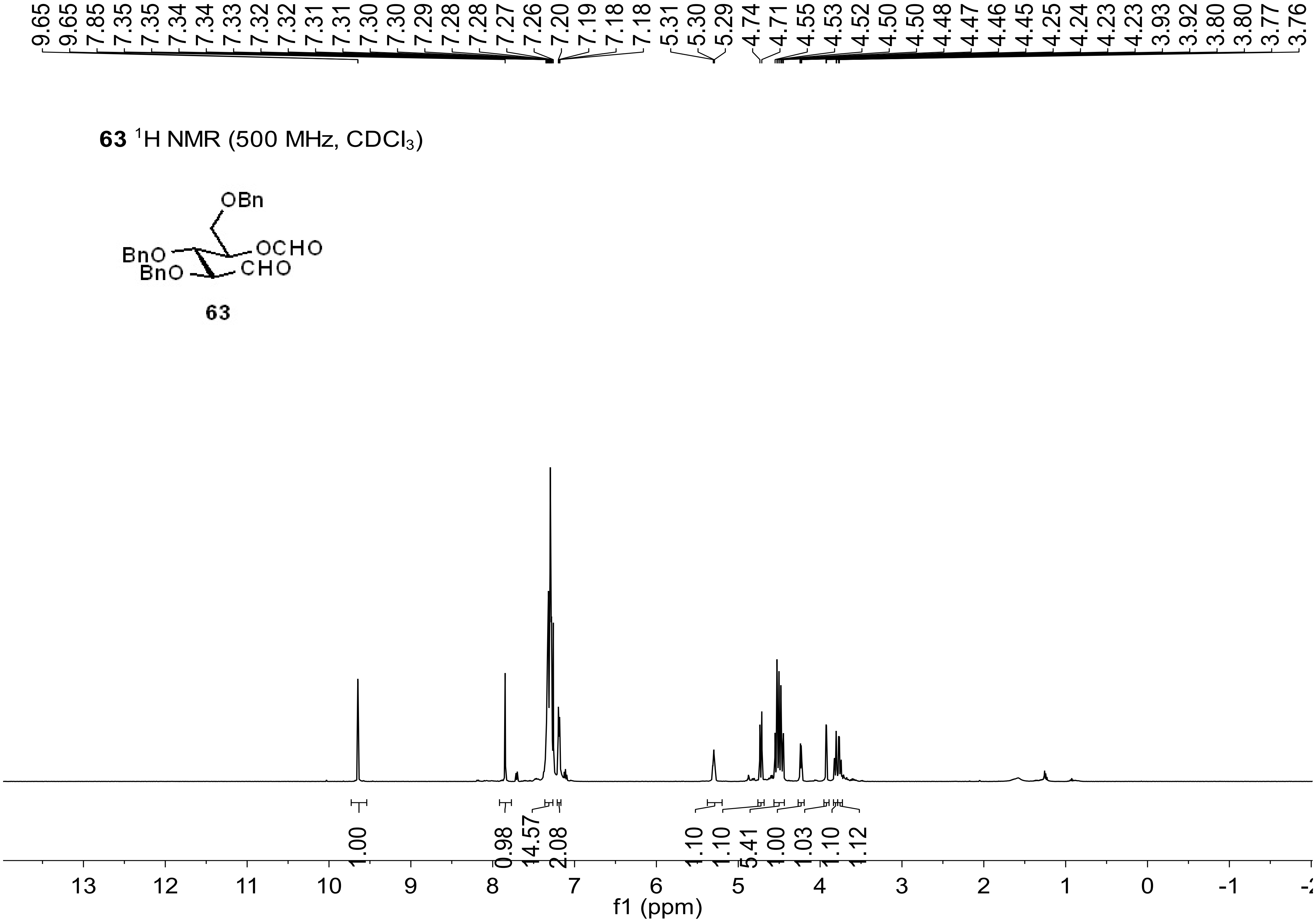

Supplementary Figure 154. ^{1}H NMR Spectrum for Compound 63

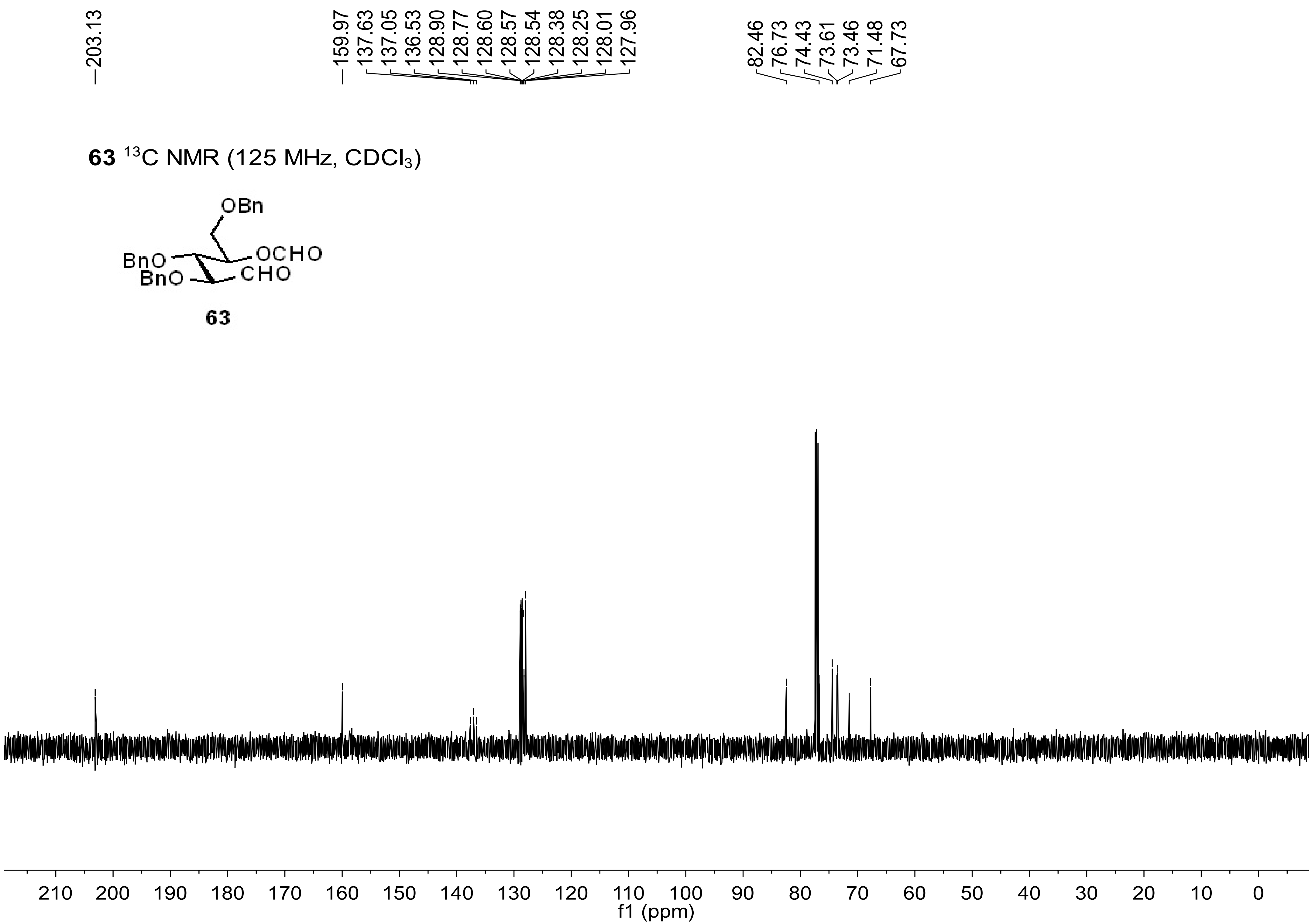

Supplementary Figure 155. ^{13}C NMR Spectrum for Compound 63

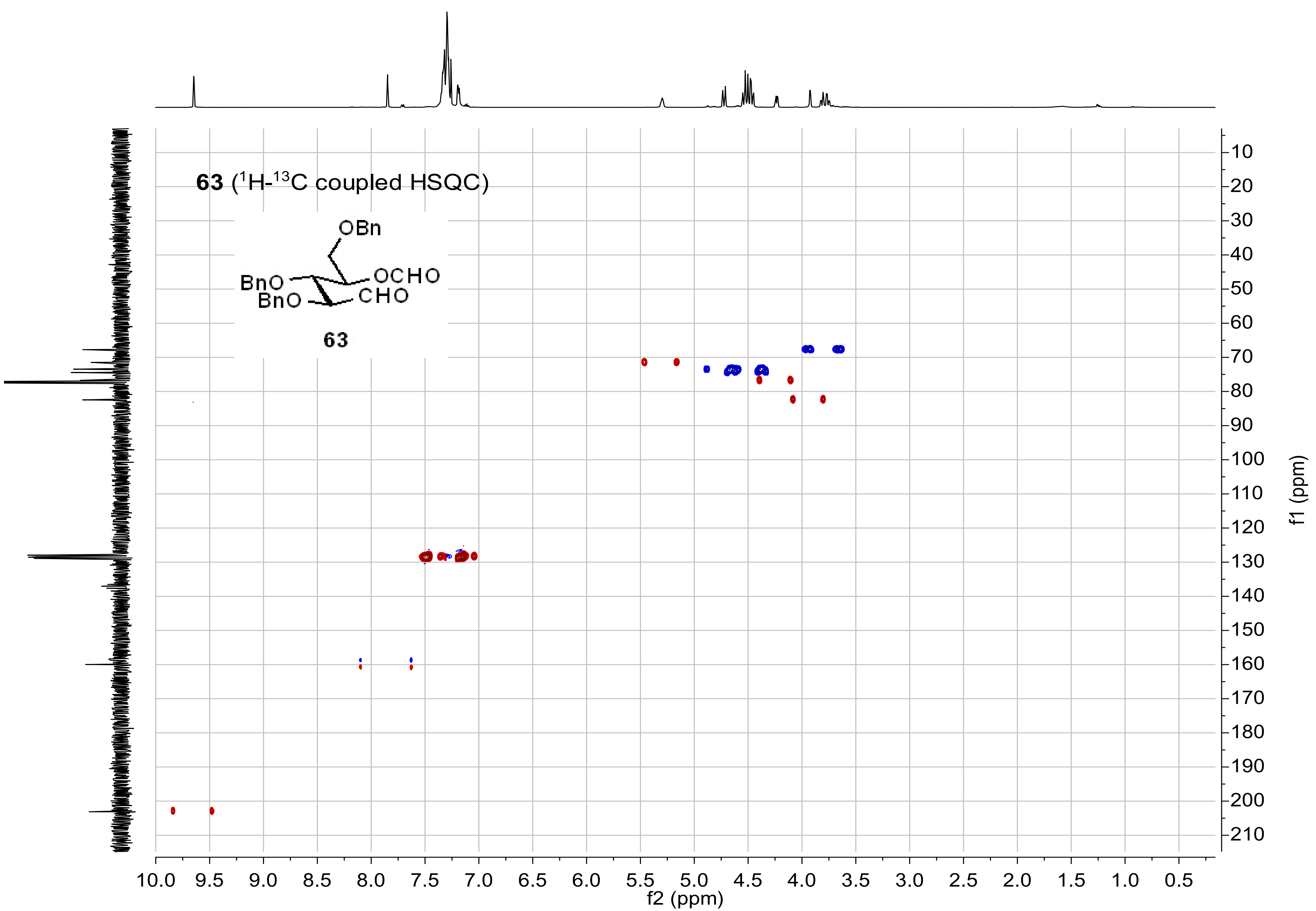

Supplementary Figure 156. ^{1}H-^{13}C HSQC Coupled Spectrum for Compound 63

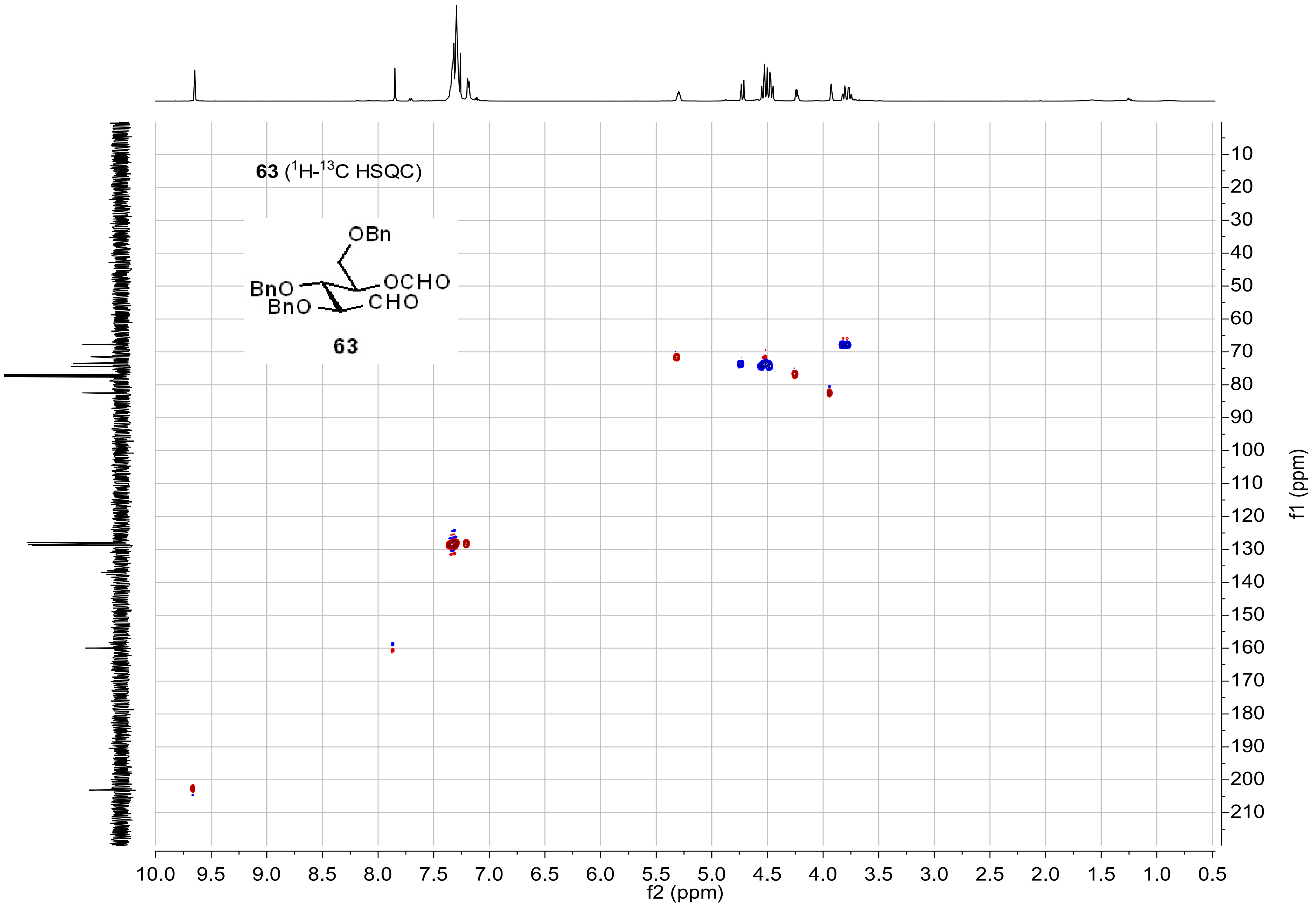

Supplementary Figure 157. ^{1}H-^{13}C **HSQC Decoupled Spectrum for Compound 63**

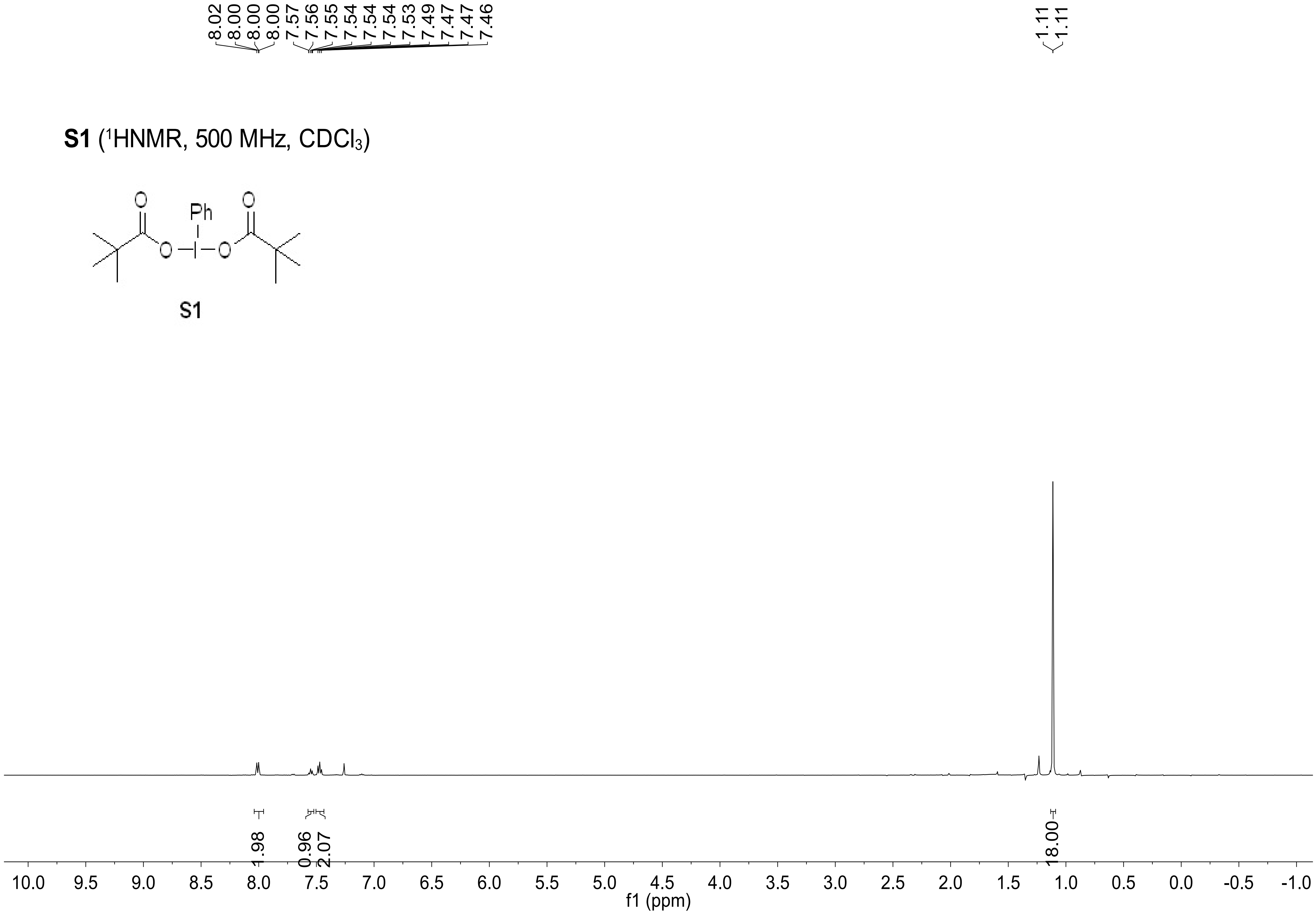

Supplementary Figure 158. ^{1}H NMR Spectrum for Compound S1

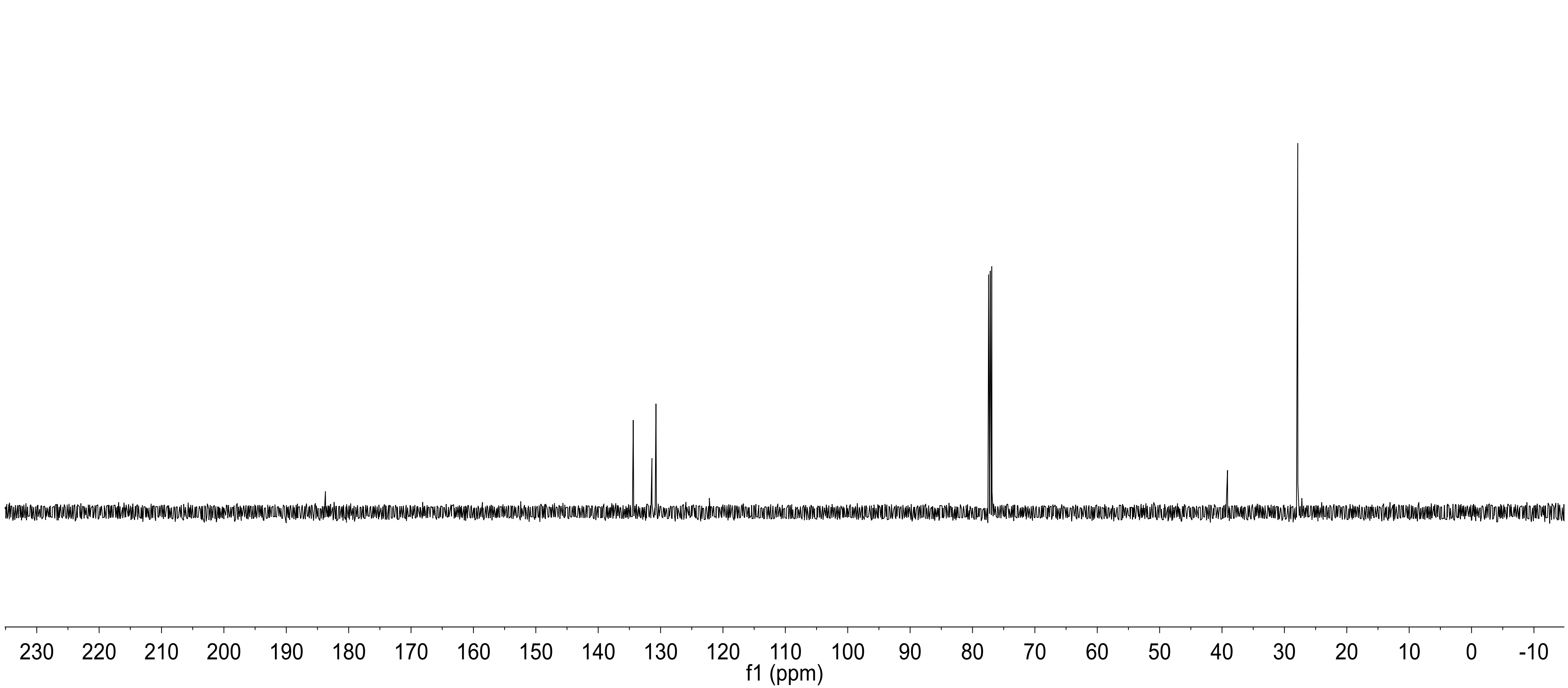

S1 (^{13}C NMR, 126 MHz, $CDCl_3$)

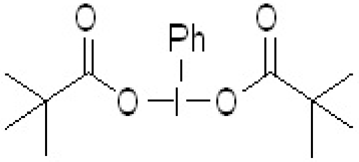

S1

Supplementary Figure 159. ^{13}C NMR Spectrum for Compound S1

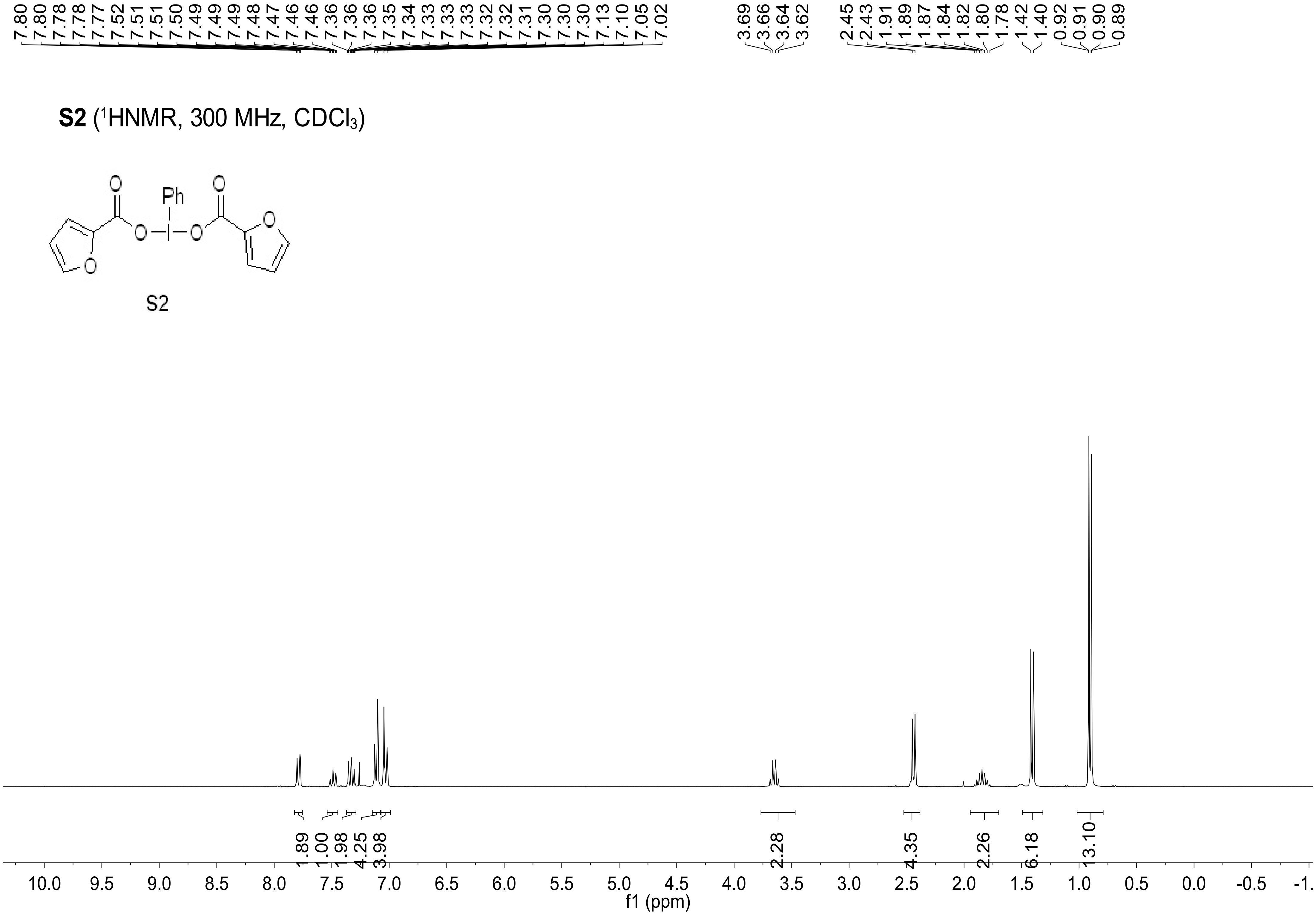

Supplementary Figure 160. ^{1}H NMR Spectrum for Compound S2

S2 (^{13}C NMR, 126 MHz, $CDCl_3$)

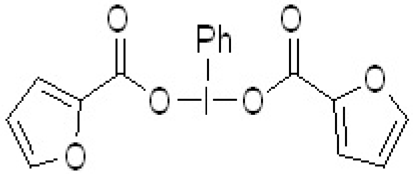

S2

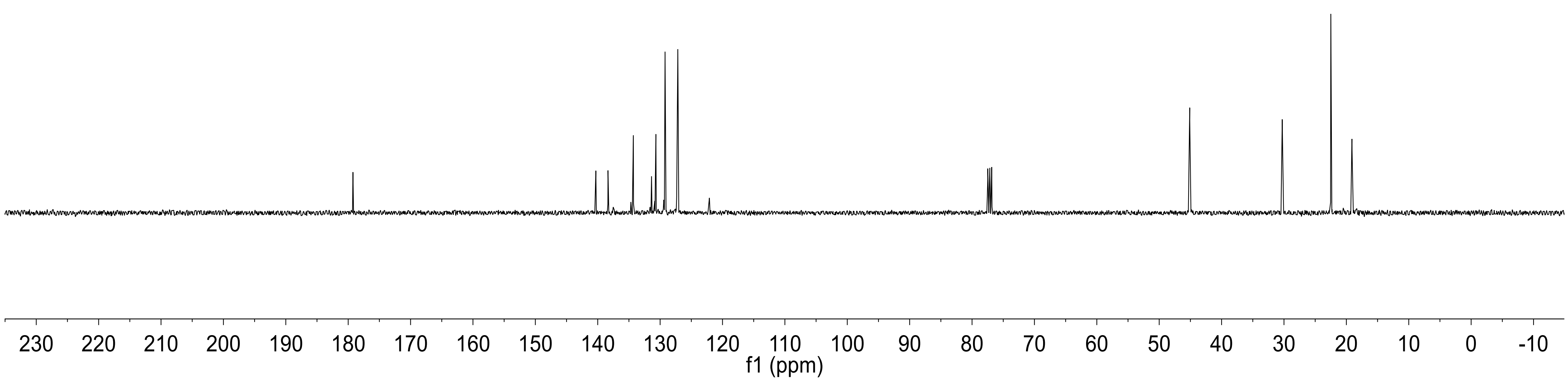

Supplementary Figure 161. ^{13}C NMR Spectrum for Compound S2

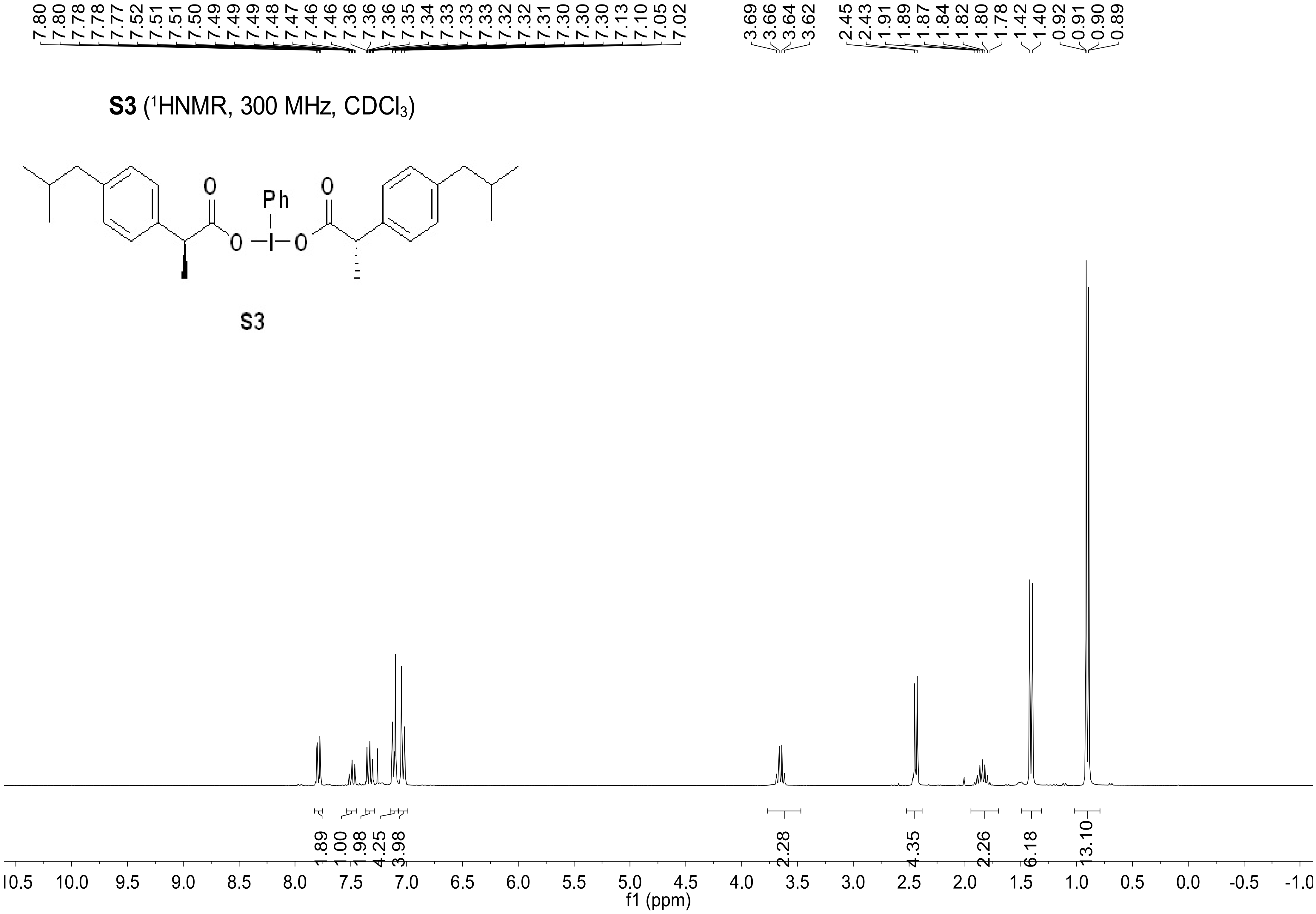

Supplementary Figure 162. ^{1}H NMR Spectrum for Compound S3

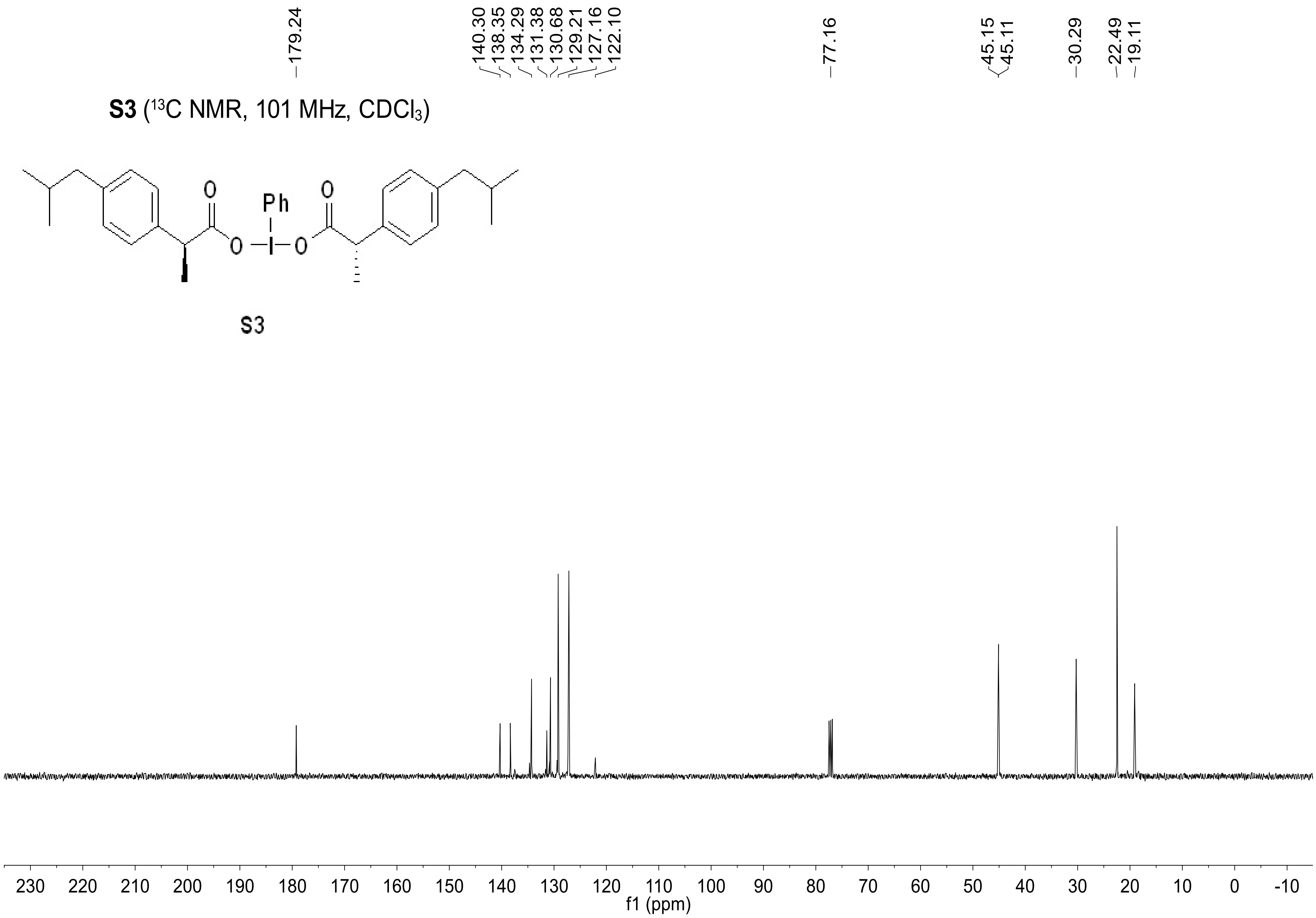

Supplementary Figure 163. ^{13}C NMR Spectrum for Compound S3

S4 (^{1}HNMR, 500 MHz, $CDCl_3$)

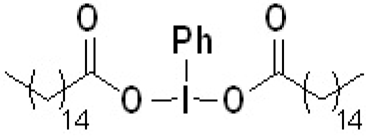
S4

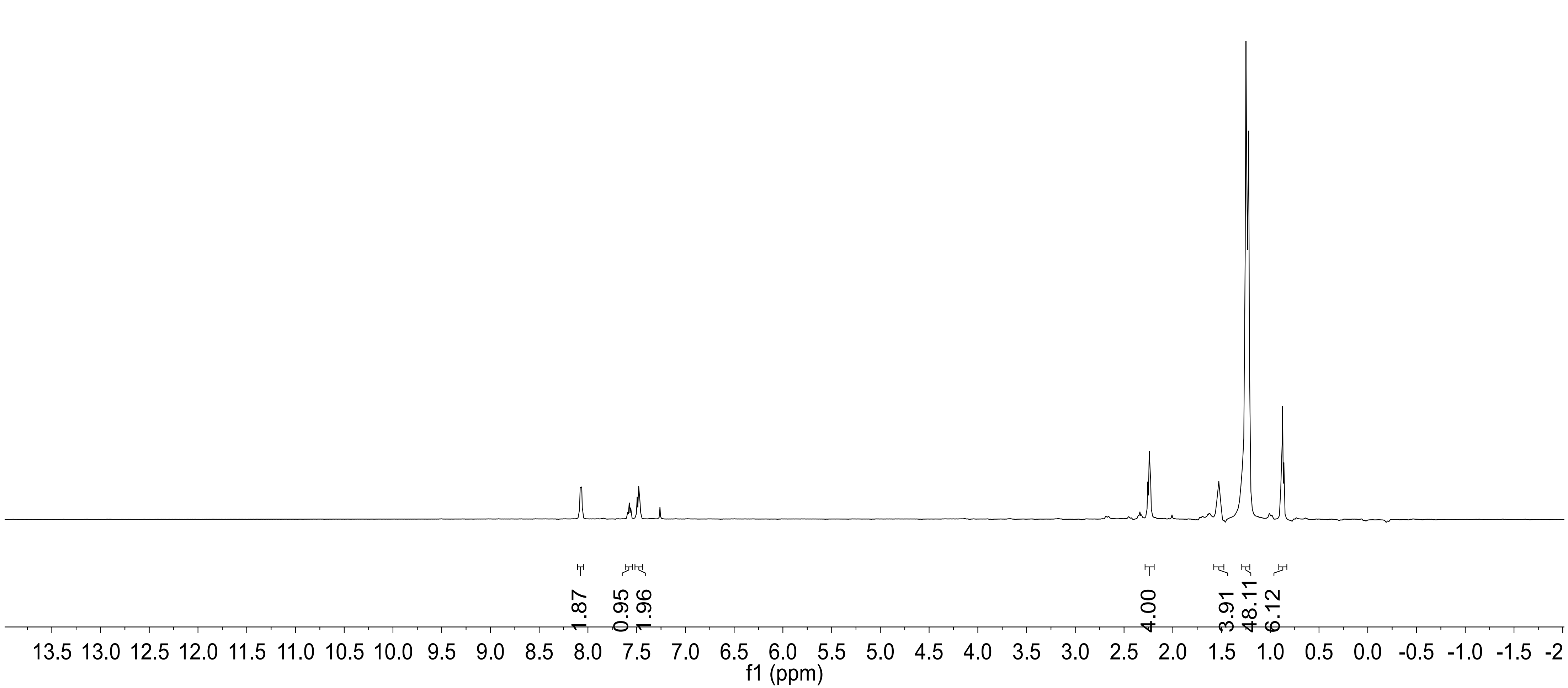

Supplementary Figure 164. ^{1}H NMR Spectrum for Compound S4

S4 (^{13}C NMR, 126 MHz, $CDCl_3$)

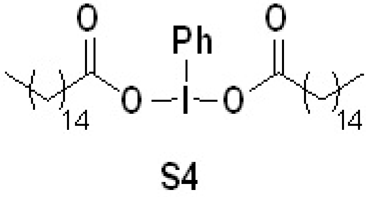

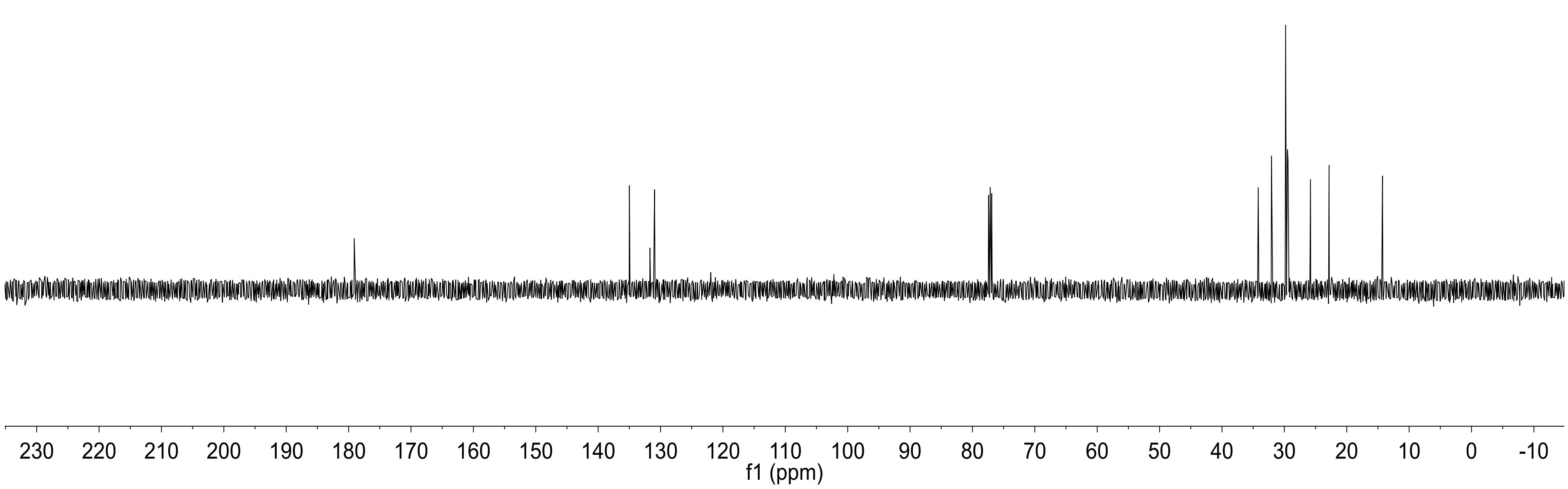

Supplementary Figure 165. ^{13}C NMR Spectrum for Compound S4

S5 (^{1}HNMR, 300 MHz, $CDCl_3$)

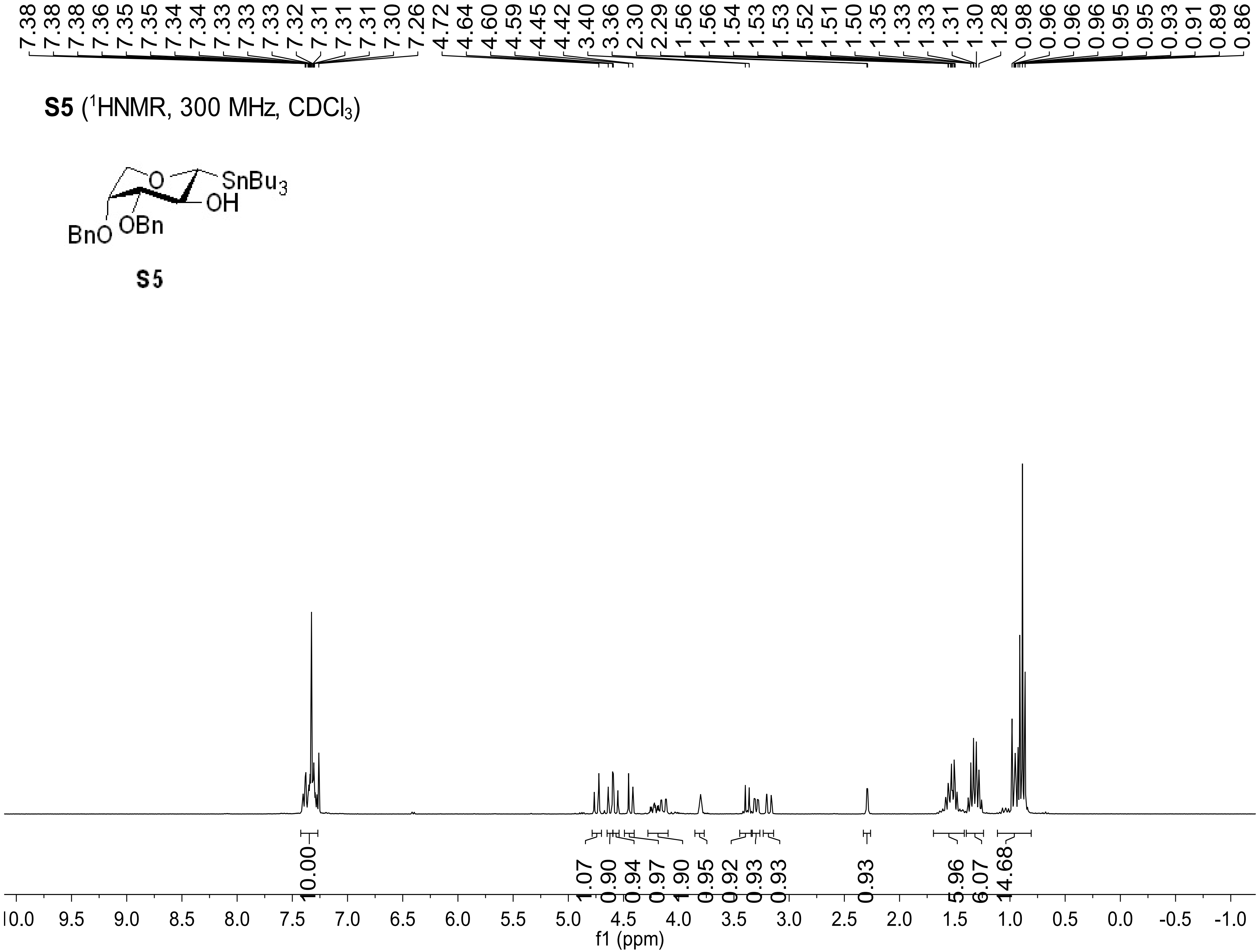

Supplementary Figure 166. ^{1}H NMR Spectrum for Compound S5

S5 (^{13}C NMR, 75 MHz, $CDCl_3$)

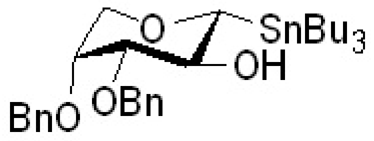

S5

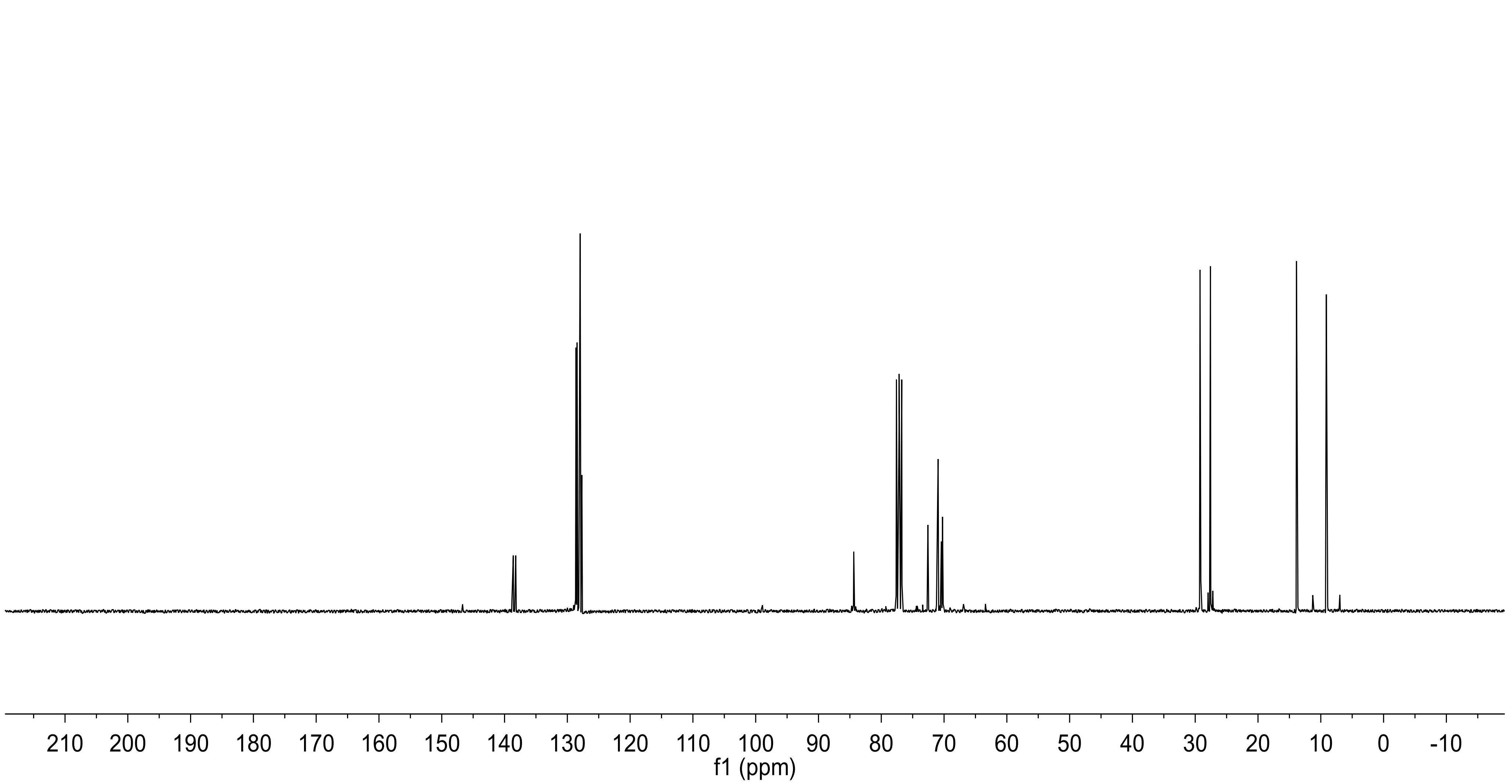

Supplementary Figure 167. ^{13}C NMR Spectrum for Compound S5

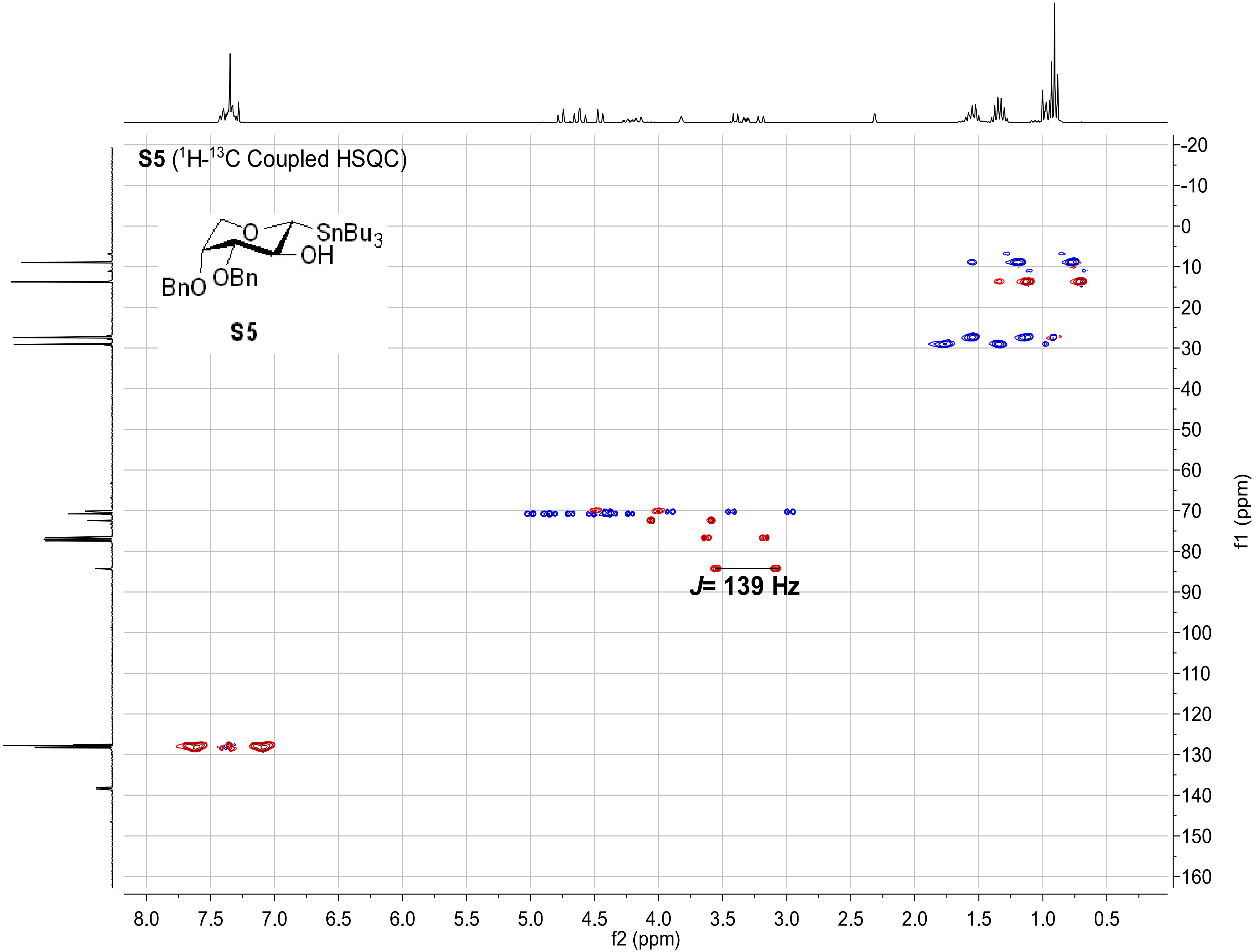

Supplementary Figure 168. ^{1}H-^{13}C HSQC Coupled Spectrum for Compound S5

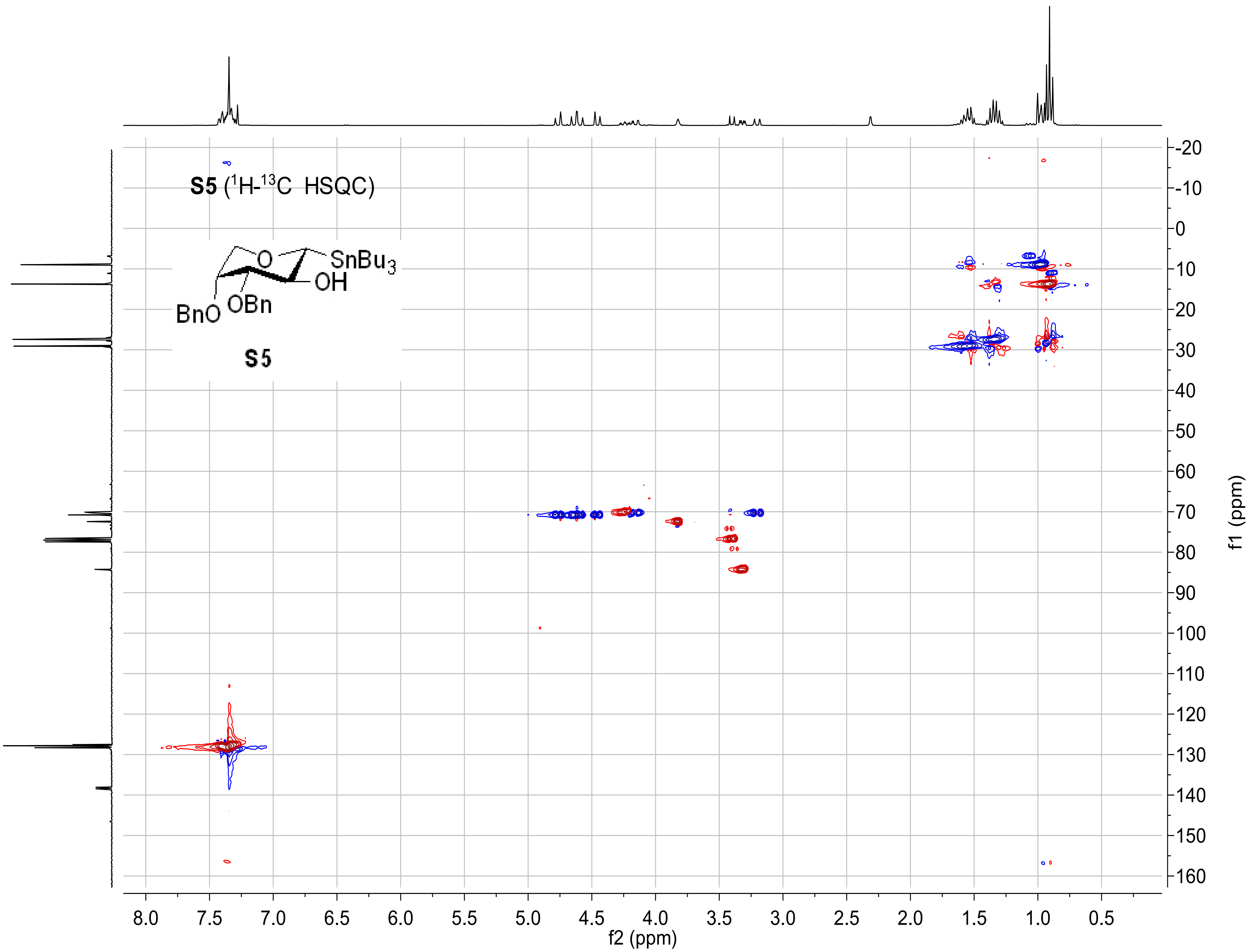

Supplementary Figure 169. **^{1}H-^{13}C HSQC Decoupled Spectrum for Compound S5**

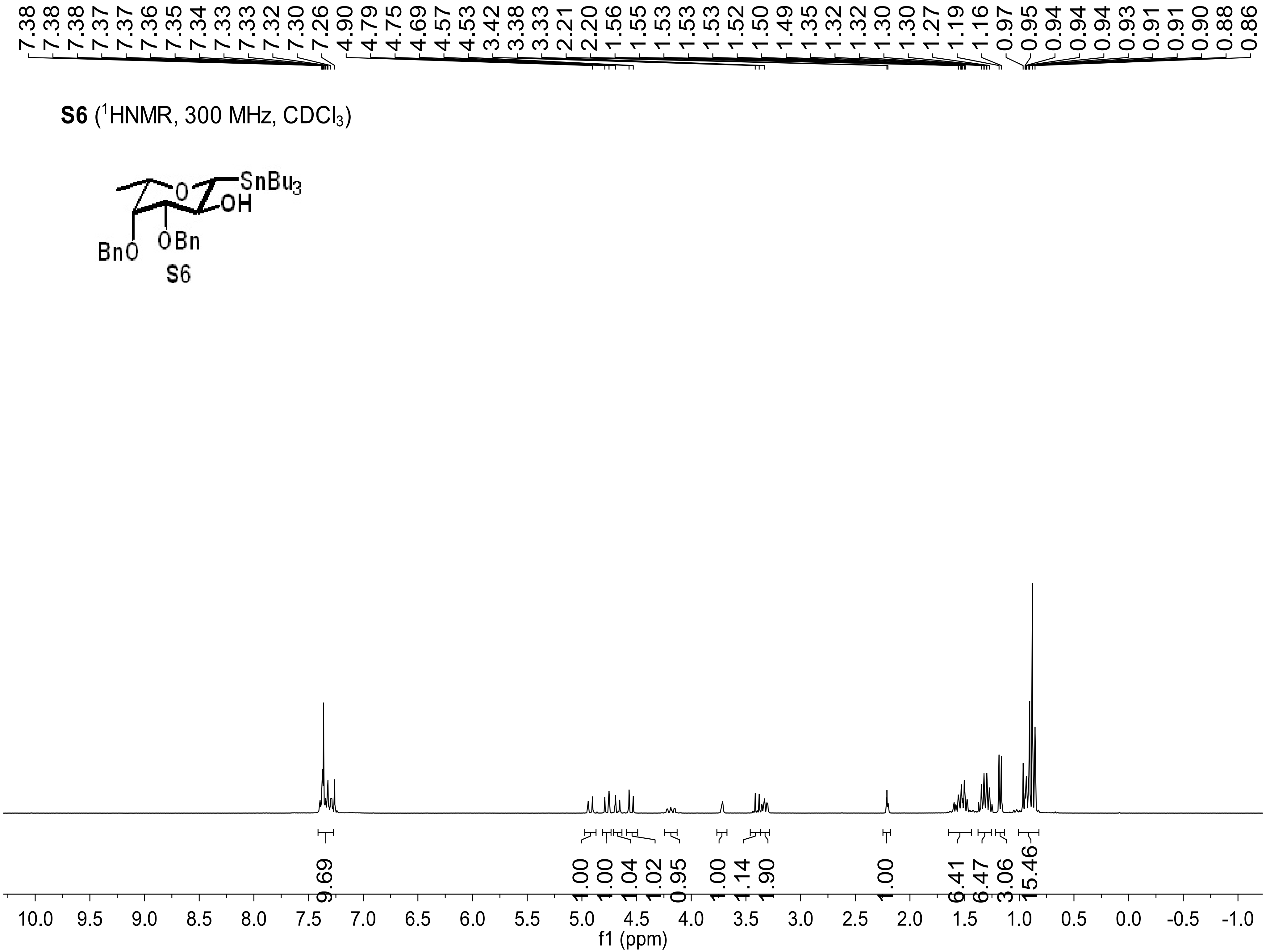

Supplementary Figure 170. ^{1}H NMR Spectrum for Compound S6

S6 (^{13}C NMR, 75 MHz, $CDCl_3$)

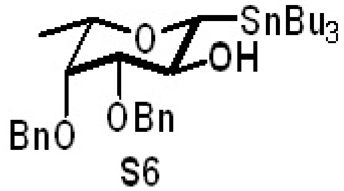

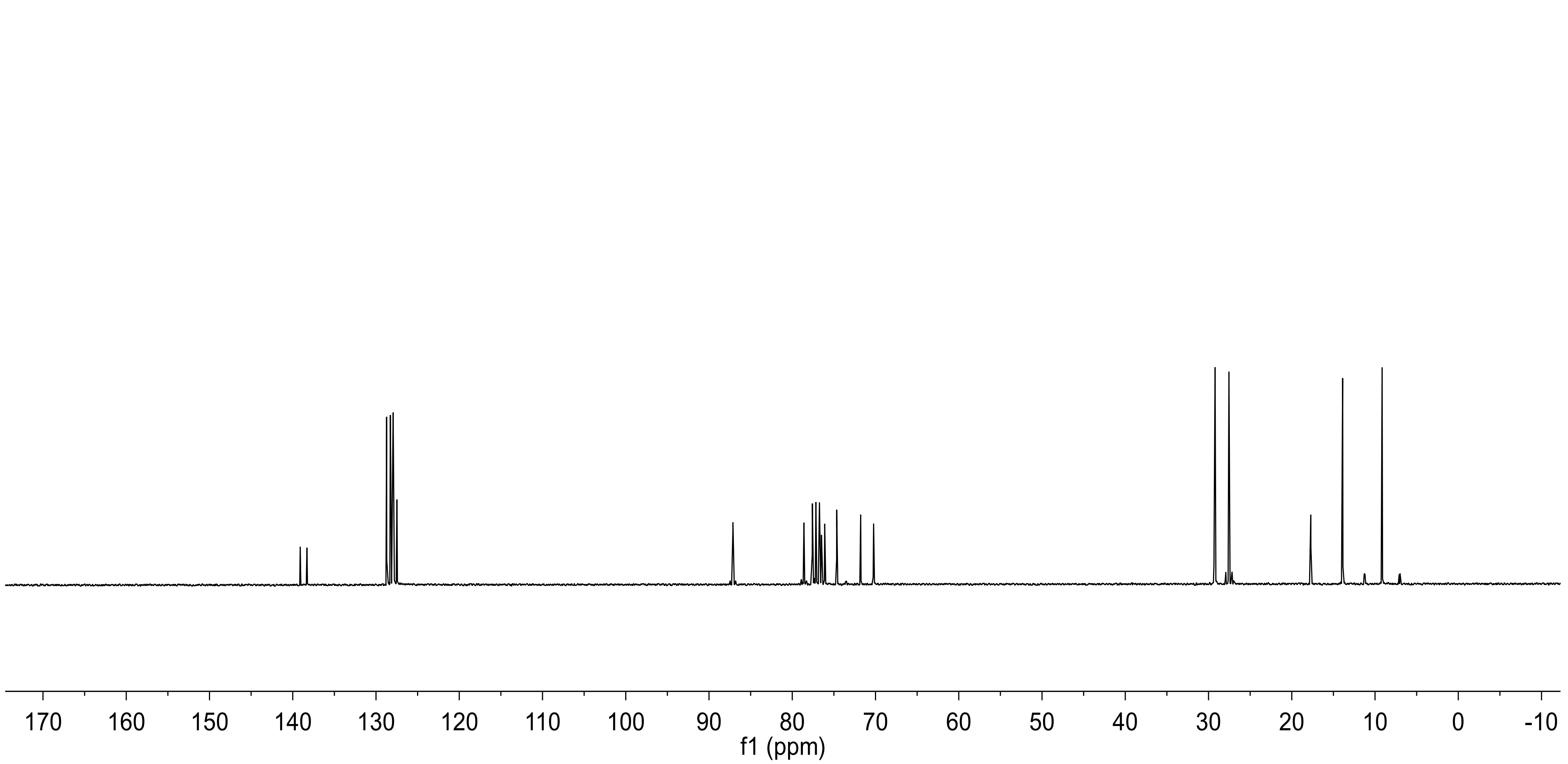

Supplementary Figure 171. ^{13}C NMR Spectrum for Compound S6

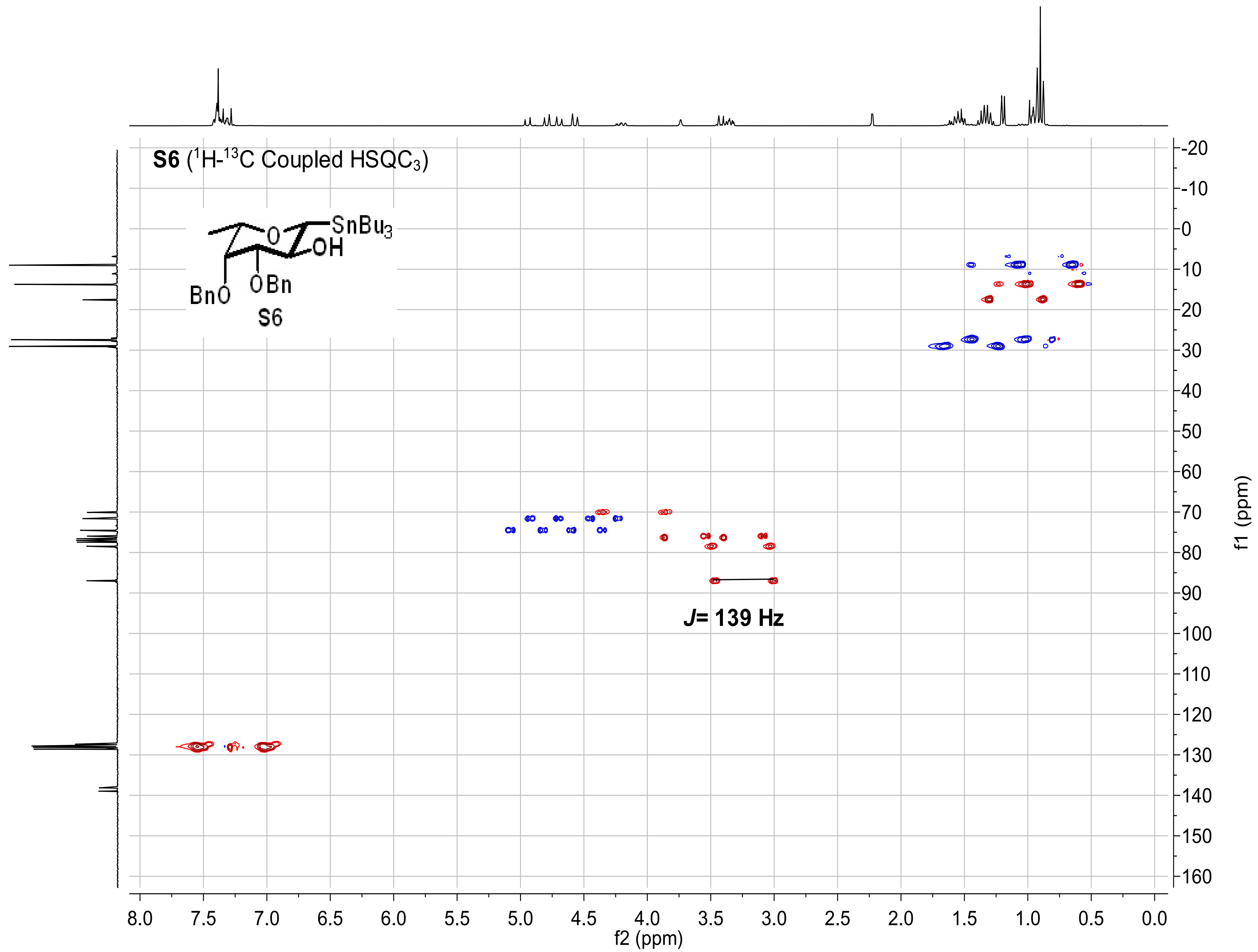

Supplementary Figure 172. **^{1}H-^{13}C HSQC Coupled Spectrum for Compound S6**

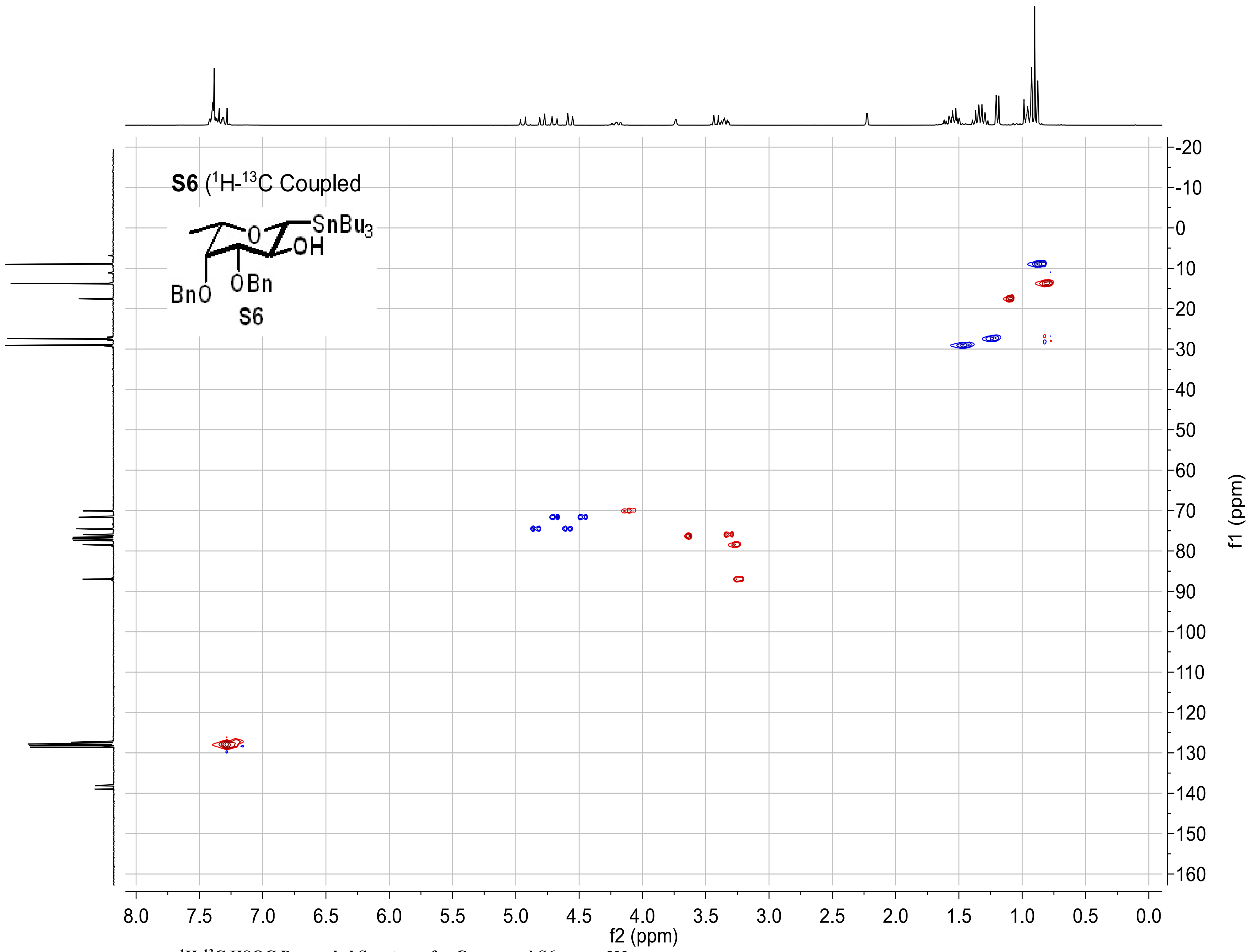

Supplementary Figure 173. **^{1}H-^{13}C HSQC Decoupled Spectrum for Compound S6**

Supplementary References

1. Zhu, F., Rourke, M.J., Yang, T., Rodriguez, J. & Walczak, M.A. Highly stereospecific cross-coupling reactions of anomeric stannanes for the synthesis of C-aryl glycosides. *J. Am. Chem. Soc.,* **138**, 12049-12051 (2016).

2. Wang, Y., Zhang, L., Yang, Y., Zhang, P., Du, Z. & Wang, C. Alkene oxyalkylation enabled by merging rhenium catalysis with hypervalent iodine (III) reagents via decarboxylation. *J. Am. Chem. Soc.,* **135**, 18048-18051 (2013).

3. Buda, S., Gołębiowska, P. & Mlynarski, J. Application of the 2-Nitrobenzyl Group in Glycosylation Reactions: A Valuable Example of an Arming Participating Group. *Eur. J. Org. Chem.*, **19**, 3988-3991 (2013)

4. Mayato, C., Dorta, R. & Vázquez, J. Experimental evidence on the hydroxymethyl group conformation in alkyl β-d-mannopyranosides. *Tetrahedron:Asymmetry*, **15**, 2385-2397 (2004).

5. Iwasaki, T., Agura, K., Maegawa, Y., Hayashi, Y., Ohshima, T. & Mashima, K. A tetranuclear-zinc-cluster-catalyzed practical and versatile deprotection of acetates and benzoates. *Chem. Eur. J.*, **16**, 11567-11571 (2010).

6. Dhar, S., La Clair, J. J., León, B., Hammons, J. C., Yu, Z., Kashyap, M. K., Castro, J. E. & Burkart, M. D. A carbohydrate-derived splice modulator. *J. Am. Chem. Soc.*, **138**, 5063-5068 (2016).

7. Hevey, R. & Ling, C. C. Studies on the 6-homologation of β-D-idopyranosides. *Carbohydr. Res.*,**445**, 65-74 (2017).

8. Kumar, V., Yadav, N. & Kartha, K. R. In (III) triflate-catalyzed detritylation and glycosylation by solvent-free ball milling. *Carbohydr. Res.*,**397**, 18-26 (2014).

9. Zhang, F., Zhang, W., Zhang, Y., Curran, D. P. & Liu, G. Synthesis and applications of a light-fluorous glycosyl donor. *J. Org. Chem.*, **74**, 2594-2597 (2009).

10. Zhu, F., Rodriguez, J., Yang, T., Kevlishvili, I., Miller, E., Yi, D., O'Neill, S., Rourke, M.J., Liu, P. & Walczak, M. A. Glycosyl Cross-Coupling of Anomeric Nucleophiles: Scope, Mechanism, and Applications in the Synthesis of Aryl C-Glycosides. *J. Am. Chem. Soc.*, **139**, 17908-17922 (2017).

11. Xia, M. J., Yao, W., Meng, X. B., Lou, Q. H. & Li, Z. J. $Co_2(CO)_6$-propargyl cation mediates glycosylation reaction by using thioglycoside. *Tetrahedron Lett.*, **58**, 2389-2392 (2017).

12. Mayato, C., Dorta, R. & Vazquez, J. Experimental evidence on the hydroxymethyl group conformation in alkyl β-d-mannopyranosides. *Tetrahedron:Asymmetry*, **15**, 2385-2397 (2017).

13. Wang, B., Xiong, D. C. & Ye, X. S. Direct C–H trifluoromethylation of glycals by photoredox catalysis. *Org. Lett.,***17**, 5698-5701 (2015).

14. Hanessian, S. & Rogel, O. Synthesis of glycophostones: cyclic phosphonate analogues of biologically relevant sugars. *J. Org. Chem.*, **65**, 2667-2674 (2000).
